# Mild and Efficient Synthesis of Diverse Organo‐Au^I^‐L Complexes in Green Solvents

**DOI:** 10.1002/cssc.201903415

**Published:** 2020-03-03

**Authors:** Fredric J. L. Ingner, Ann‐Cathrin Schmitt, Andreas Orthaber, Paul J. Gates, Lukasz T. Pilarski

**Affiliations:** ^1^ Department of Chemistry—BMC Uppsala University BOX 576 75-123 Uppsala Sweden; ^2^ Department of Chemistry—Ångström Uppsala University BOX 523 75-120 Uppsala Sweden; ^3^ School of Chemistry University of Bristol Cantock's Close, Clifton Bristol BS8 1TS UK

**Keywords:** boronates, gold, green solvents, NHCs, transmetalation

## Abstract

An exceptionally mild and efficient method was developed for the preparation of (hetero)aryl‐Au^I^‐L complexes using ethanol or water as the reaction medium at room temperature and Ar‐B(triol)K boronates as the transmetalation partner. The reaction does not need an exogeneous base or other additives, and quantitative yields can be achieved through a simple filtration as the only required purification method, which obviates considerable waste associated with alternative workup methods. A broad reaction scope was demonstrated with respect to both the L and (hetero)aryl ligands on product Au complexes. Despite the polar reaction medium, large polycyclic aromatic hydrocarbon units can be incorporated on the Au complexes in very good to excellent yields. The approach was demonstrated for the chemoselective manipulation of orthogonally protected aryl boronates to afford a new class of N‐heterocyclic carbene‐Au‐aryl complexes. A mechanistic rationale was proposed.

## Graphical abstract

**Triols and triturations**! High‐yield synthesis of diverse aryl‐Au^I^ complexes is achieved under mild conditions in green solvents (ethanol or water) by harnessing the unique properties of aryl‐B(triol)K boronates. The method permits transmetalation from aryl‐B(triol)K boronates while preserving aryl‐B(pin) boronates on the substrate. Pure products are obtained through a simple filtration.

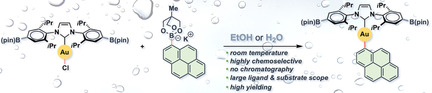

## Introduction

Synthetic chemistry is under increasing pressure to provide environmentally benign routes to functional molecules. Reducing the reliance on hazardous and/or toxic reagents and solvents has acquired increasing urgency. Several legislative measures have been taken across the world to restrict the future use of various toxic, carcinogenic, and/or environmentally damaging solvents.^1^ Their replacement with greener alternatives is of growing interest in various areas of synthesis,^2^ including organometallics and homogeneous catalysis,^3^ and across the pharmaceutical sector.^4^

**Figure 1:**
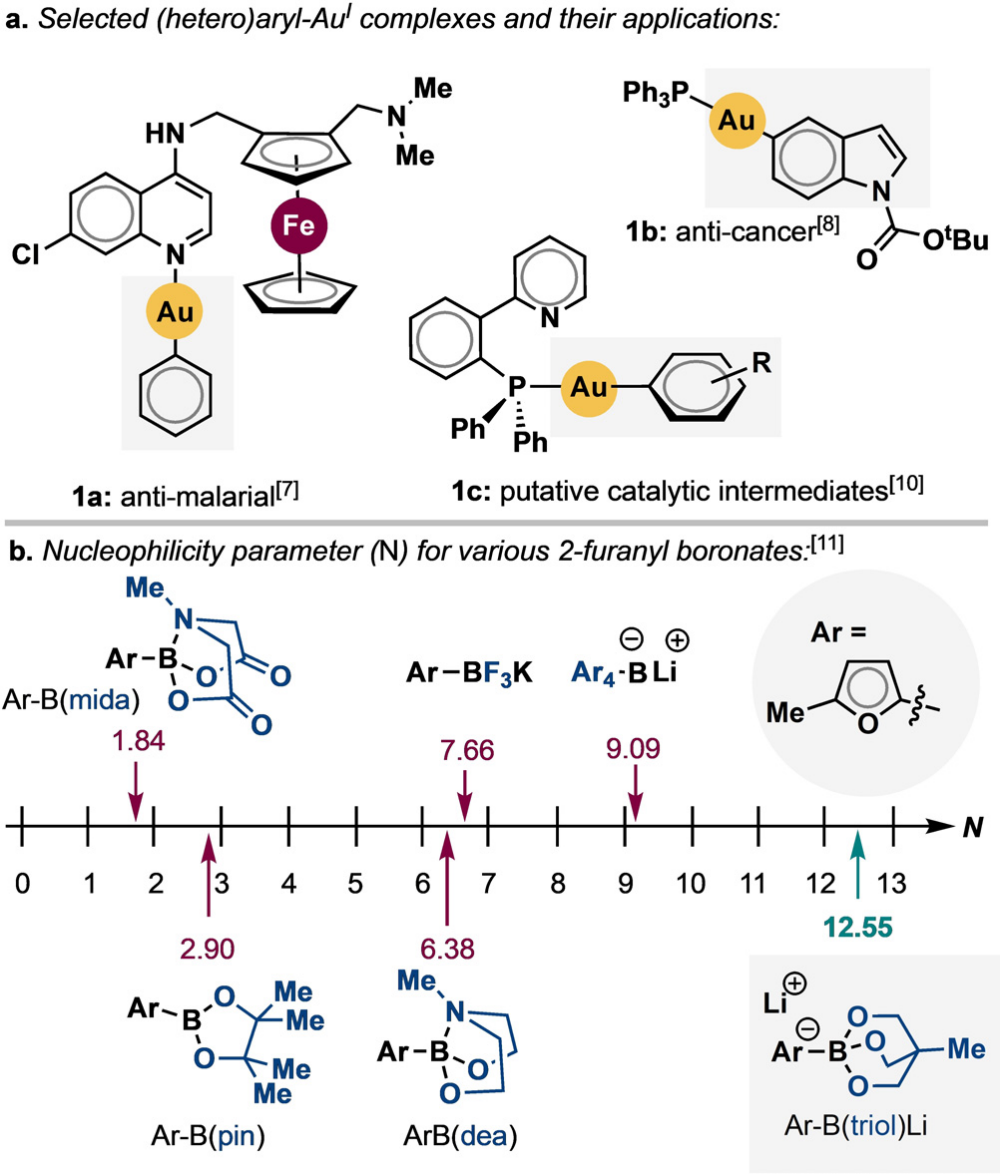
(a) Example organo‐Au^I^ complexes and their properties; (b) relative nucleophilicities of selected arylboronates reported by Mayr and co‐workers.^11^.

As part of a wide‐ranging project in our group to investigate new methods for the manipulation of aryl‐transition‐metal systems, we sought to develop a reliable and exceptionally mild approach for the synthesis of diverse L‐Au^I^‐(hetero)aryl complexes. In recent years, organo‐Au^I^ complexes have attracted considerable interest because of their various forms of biological activity^5^ and for their promise in materials applications.^6^ For example, complexes **1 a** 7 and **1 b** 8 (Figure 1) exhibit antimalarial and anticancer properties, respectively. L‐Au^I^‐(hetero)aryl complexes are also of great interest as intermediates in homogeneous catalysis,^9^ as exemplified by **1 c**.^10^ Herein, we report a uniquely mild, versatile, and efficient approach to L‐Au^I^‐(hetero)aryl complexes using green solvents and without any need for extraction, recrystallization, or chromatography to obtain analytically pure products.

We specifically set out to develop a protocol to prepare L‐Au^I^‐Ar complexes that can tolerate a wide variety of functional groups and ligands, require only environmentally benign/nontoxic solvent media, and, importantly, work efficiently without any other exogeneous additives or heating using well‐defined reagents. Although, conventionally, organoboronic acids^12^ and their pinacol (pin) esters9m, 12c, 13 have been used to generate organo‐Au^I^ complexes, the former are not well defined because they can contain varying amounts of boroxine species, and are often used in excess. Additionally, previously reported methods have often relied on unsustainable/hazardous solvents, with exogeneous additives and at elevated temperatures. With our aims in mind, we considered boronates of the type [ArB(triol)]M (Figure 1 b), which have never been used in this context as candidate sources of the aryl group.^14^ Originally developed by Yamamoto and co‐workers as effective reagents in various coupling reactions,^15^ such boronates have also been shown to be significantly more nucleophilic than several of their more commonly used congeners (selected variants are shown in Figure 1 b).^11^

The triol itself, 2‐(hydroxymethyl)‐2‐methylpropane‐1,3‐diol, has low toxicity, is significantly cheaper than pinacol,^16^ and is produced on a multi‐ton scale annually.^17^ We anticipated that the transmetalation of (hetero)aryl groups from such boronates to an Au^I^ center should be particularly efficient on account of their significantly enhanced nucleophilicity^11^, ^18^ and because they have been shown to benefit transmetalation to other transition metals.15e, 15f, 19 For example, Chan–Lam aminations using [PhB(triol)]K proceed at three times the rate of the equivalent reactions using PhB(OH)_2_ or PhBF_3_K as the arylating reagent.^20^ We envisaged the prospect of an alternative intramolecular transmetalation pathway through intermediate alkoxide adducts (see below).

## Results and Discussion

In initial experiments, we compared a range of 3‐tolyl boronates (**2 a**–**d**) in transmetalation to the Au^I^ center of Ph_3_PAuCl (**3 a**) in various solvents (Table 1, entries 1–8). Triolboronate **2 a** proved substantially more efficient in ethanol (EtOH; entry 7) and even H_2_O (entry 8) than in toluene12a, 12c, 21 or acetonitrile,9s, 22 both of which have served as solvents of choice in many previously reported protocols. Moreover, in contrast to many previous methods, the transmetalation occurred efficiently without any heating. Boronate **2 a** was the only one amongst several (**2 a**–**d**) to afford the desired products in H_2_O, even if elevated temperatures were used (entries 9–12).

**Table 1:** Selected results from the comparison of different boronates in the transmetalation to **3 a**.^[a]^

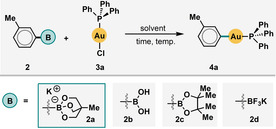					
Entry	Boronate	Solvent	*T* [°C]	*t* [h]	Yield [%]
1	**2 a**	toluene	23	24	43
2	**2 a**	dichloromethane	23	24	60
3	**2 a**	THF	23	24	46
4	**2 a**	ethyl acetate	23	24	39
5	**2 a**	acetonitrile	23	24	45
6	**2 a**	1‐propanol	23	24	88
7	**2 a**	EtOH	23	24	97
8	**2 a**	H_2_O	23	24	85
9	**2 b**	H_2_O	50	5	0
10	**2 c**	H_2_O	50	5	0
11	**2 d**	H_2_O	50	5	0
12	**2 a**	H_2_O	50	5	87

[a] Ph_3_PAuCl (0.05 mmol), boronate (1 equiv.), solvent (0.5 mL). Yields determined by ^31^P NMR spectroscopy.

**Scheme 1:**
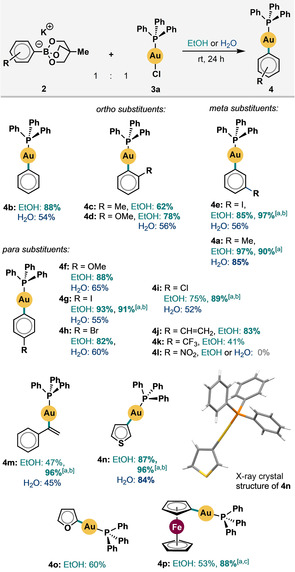
Boronate scope in EtOH and H_2_O solvents; Ph_3_PAuCl (0.1 mmol), boronate (1.0 equiv.); unless otherwise stated the yield was determined by ^31^P NMR spectroscopy; [a] isolated yield; [b] 3.0 equiv. of boronate used; [c] 1.2 equiv. of boronate used.

Subsequently, we extensively investigated the scope with respect to both five‐ and six‐membered (hetero)aryl boronates using **3 a** as the gold precursor (Scheme 1). The reactions proved very clean: only starting materials and products were observed when the reaction progress was monitored by ^31^P NMR spectroscopy. Very good to excellent conversions were obtained with almost all substrates in EtOH at room temperature using only a 1:1 ratio between **2** and **3 a**. Boronic acids bearing methyl (**4 a**, **c**), methoxy (**4 d**, **f**), iodo (**4 e**, **g**), bromo (**4 h**), chloro (**4 i**), vinyl (**4 j**), and trifluoromethyl (**4 k**) groups performed well. The use of 1.2–3.0 equivalents of the boronate invariably returned >99 % conversion and very good to near‐quantitative yields. For example, the vinyl‐Au^I^ complex **4 m** formed in up to 96 % yield, whereas the 2‐furanyl (**4 o**) and ferrocenyl (**4 p**) units returned 96 and 88 % yield, respectively. The identity of the product complex **4 n** was confirmed by X‐ray crystallography (see the Supporting Information for more details). Furthermore, no difficulties arising from the previously suggested S⋅⋅⋅Au coordination in thiophene substrates was observed.12c Generally, electron‐rich substrates performed slightly better, and only the very strongly electron‐withdrawing nitro group (**4 l**) prevented product formation. Steric hindrance from substituents in the *ortho*‐position relative to the C−Au bond imposed only a modest reduction in yield (e.g., **4 d** vs. **4 f**).

No recrystallization or chromatographic separation were required: although each reaction mixture remained heterogeneous throughout, analytically pure products could be obtained simply through a single filtration at the end of the reaction time. This is a marked advantage over previously reported procedures for the preparation of L‐Au^I^‐(hetero)aryl complexes, which have often relied on the use of halogenated solvents during the work‐up procedure. Even water proved to be a viable, albeit slightly less effective, solvent (Scheme 1; yields from reactions performed in water are shown in blue).2b, 3

**Scheme 2:**
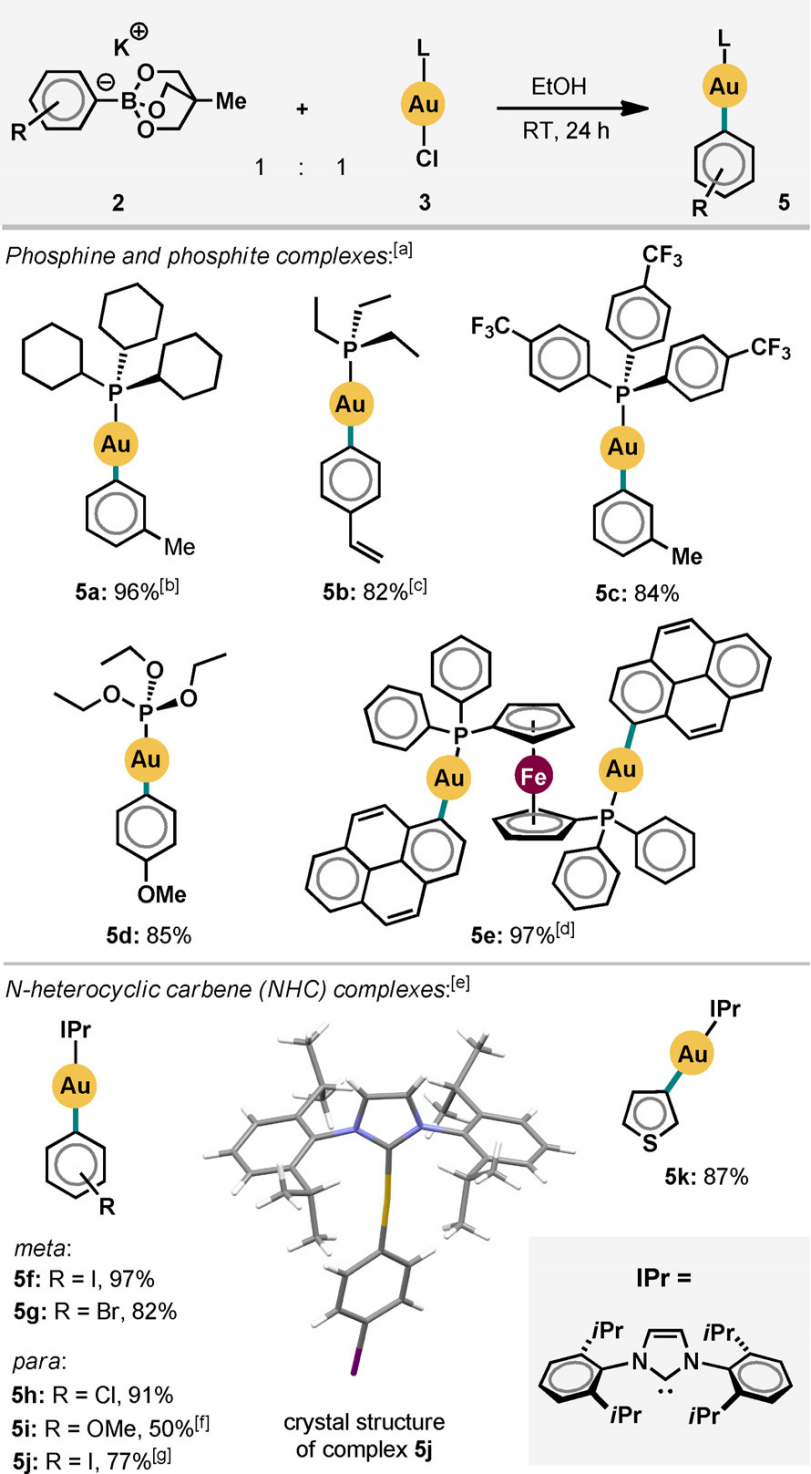
Scope with respect to the L ligand; isolated yields unless otherwise specified; 0.1 mmol‐scale of LAuCl [a] 1.0 equiv. of boronate used; [b] 1.5 equiv. of boronate used; [c] yield determined by ^31^P NMR spectroscopy; [d] ratio of reagents used: [(dppf)(AuCl)_2_]/boronate=1:2 [dppf=1,1′‐bis(diphenylphosphino)ferrocene]; [e] 1.2 equiv. of boronate used; [f] yield determined by ^1^H NMR spectroscopy using 1,3,5‐trimethoxybenzene as standard; [g] reaction performed on 0.4 mmol‐scale.

Next, we investigated the scope of the reaction with respect to a variety of L‐type ligands on the Au^I^ center. Trialkyl phosphine ligands with both large (**5 a**) and small (**5 b**) cone angles^23^ performed well. Weaker σ‐donor ligands, such as tris(4‐trifluoromethylphenyl)phosphine (**5 c**) and triethylphosphite (**5 d**), also gave very good yields (Scheme 2). Under our reaction conditions, the trimetallic ferrocene‐based complex **5 e** was obtained in 97 % isolated yield. The efficiency of this reaction is particularly notable given the presence of multiple planar π‐extended and apolar groups. Dinuclear Au^I^ complexes have attracted attention in various contexts, including as potential anticancer therapies^24^ and in catalysis.^25^ Additionally, N‐heterocyclic carbene (NHC)‐Au‐aryl complexes bearing a variety of functional groups formed efficiently (**5 f**–**k**). The only exception was the 4‐methoxyphenyl variant (**5 i**), presumably owing to protodeauration, which is accelerated by polar protic solvents for NHC‐Au^I^ complexes with electron‐rich arene ligands^26^ (compare **4 f**, Scheme 1). The reaction scaled well: compound **5 g** was prepared on a 0.4 mmol‐scale, which is 8 times the scale used in our initial optimization.

**Scheme 3:**
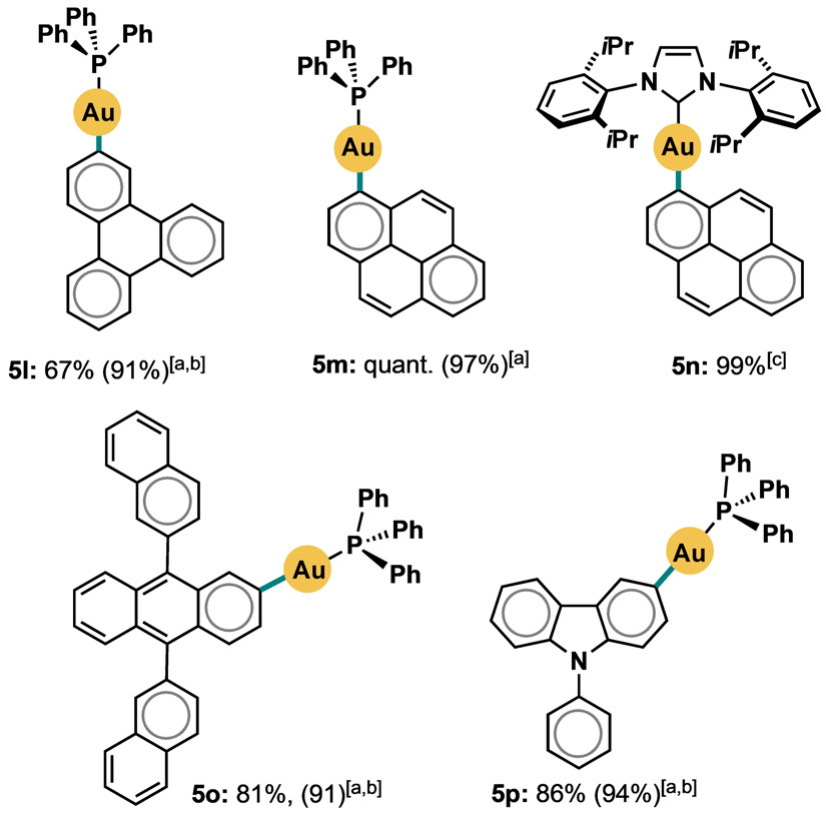
Synthesis of PAH‐containing complexes; general conditions as in Scheme 2; PPh_3_AuCl complex (0.1 mmol), boronate (1.0 equiv.); yields determined by ^31^P NMR spectroscopy unless otherwise stated; [a] isolated yield; [b] 3.0 equiv. of boronate used; [c] 1.2 equiv. of boronate used.

Various organometallic complexes incorporating polycyclic aromatic hydrocarbons (PAHs), including those of Au^I^, have attracted attention because of their interesting photophysical properties and potential applications in molecular recognition.13b, 27 Encouraged by the excellent yield of **5 e**, we turned our attention to the synthesis of complexes based on several PAH units (Scheme 3). Products **5 l**–**p** were isolated in very good to quantitative yield: up to 97 % yield (**5 m**) using only one equivalent of the appropriate [aryl‐B(triol)]K reagent and up to 99 % yield (**5 n**) using only 1.2 equivalents. This was a significant improvement over several previous reports of the synthesis of PAH‐containing Au^I^ complexes, for which poor to moderate yields have been common until now.13b, 22c, 28 In all our examples, analytically pure products could be obtained through a simple filtration, obviating the need for additional extraction, recrystallization, or chromatography. In previous reports, purification has relied on the use of chlorinated solvents, which are increasingly the subject of stringent legislative regulation.^1^

N‐aryl carbazole units feature in a range of organic electronic applications as easily tunable charge transporters.^29^ Under our conditions, the auration of *N*‐phenyl carbazole (**5 p**) occurred with 86 % conversion using only 1 equivalent of the corresponding triol boronate, and in 94 % isolated yield using 3 equivalents.

**Figure 2:**
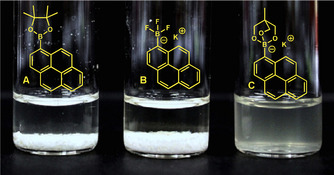
Photograph of pyrenyl boronates (2 mmol) in EtOH (2 mL).

In a more general context, the manipulation of PAHs can often suffer from poor solubility in many solvents. In contrast to their −B(pin) and −BF_3_K congeners, each of the starting material [ArB(triol)]K substrates dissolved easily in EtOH at room temperature (Figure 2). This should open new opportunities for the manipulation of PAHs.^30^

To address a related environmental aspect, and to mitigate concerns that the overall method is reliant on unrenewable solvents, we synthesized a representative range of the parent [ArB(triol)]K reagents in 2‐methyltetrahydrofuran, a solvent obtained from renewable resources.^31^ These syntheses returned comparable or higher yields than the more commonly used THF or toluene solvents (see the Supporting Information for further details).

**Scheme 4:**
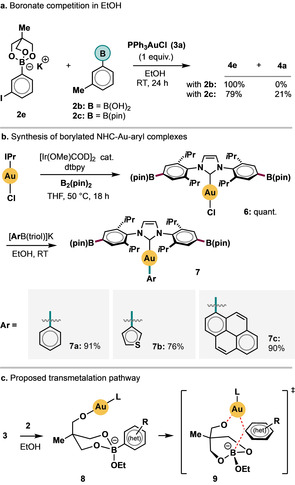
(a) Competition study: Ph_3_PAuCl (0.1 mmol) and boronates (1 equiv. each) were used; (b) use of differentiated boronate reactivity to generate novel organogold complexes; (c) tentative intramolecular transmetalation pathway based on preliminary mechanistic data (see the Supporting Information for further information).

Species bearing multiple, differentiated boronate groups can impart various unique advantages in synthesis.^32^ As part of a wider program of studies into Au^I^ complexes, we sought to exploit the differentiated reactivity of **2 a**, **2 b**, and **2 c** to generate a new class of decorated NHC‐Au^I^‐aryl complexes that retained versatile boronate functionality for further manipulation. In a competition experiment, significantly faster transmetalation to PPh_3_AuCl (**3 a**) in EtOH was achieved with boronate **2 e** compared with **2 b** or **2 c** (Scheme 4 a). As shown in Scheme 4 b, the Ir‐catalyzed borylation^33^ of IPr‐Au‐Cl gave the diborylated complex **6** in quantitative yield. To the best of our knowledge, this marks the first reported use of C−H functionalization methodology to derivatize coordinated NHC ligands.^34^

Subsequently, several aryl groups were introduced on the Au^I^ center using [ArB(triol)]K boronates in good to excellent yields (products **7 a**–**c**) under our mild conditions without any evidence of C−B bond cleavage on the boryl NHC moiety in **6**. In contrast, attempts to generate complexes **7** from the corresponding (hetero)arylboronic acids invariably led to intractable mixtures.

We propose that, in addition to their significant nucleophilicity, boronates **2** benefit from the lability of an alkoxide residue, leading to the formation of complexes **8**. Thereafter, fast intramolecular transmetalation (transition states **9**, Scheme 4 c) presumably occurs; hydroxide and alkoxide organo‐Au^I^ complexes are known to participate in transmetalation processes.12c, 35 Experiments in support of this proposed pathway are presented in the Supporting Information.

## Conclusions

Triol‐based boronates afford a mild, convenient, high‐yielding, and versatile pathway to L‐Au^I^‐(hetero)aryl complexes. Our method works efficiently in green solvents at room temperature, often without an excess of the boronate reagent. No extraction, recrystallization, or chromatography methods were required: product isolation was achieved solely by filtration. Uniquely, the reactant scope includes phosphine, phosphite, and N‐heterocyclic carbene (NHC) L‐type ligands, as well as diversely substituted (hetero)aryl and polycyclic aromatic hydrocarbon (PAH)‐based aryl groups transferred to the Au^I^ center. The significantly higher efficiency of transmetalation from triol‐based boronates compared with their congeners permits the diversification of C−H borylated NHC‐Au complexes. We envisage that this can be leveraged to construct new Au^I^ complexes with valuable bioactivity profiles and properties useful in organic electronics applications. Work on this continues in our laboratory.

## Experimental Section

### Representative procedure

Ph_3_PAuCl (0.1 mmol, 1 equiv.) and the desired triol‐based aryl boronate (0.3 mmol, 3 equiv.) were added to a 12 mL microwave vial equipped with a magnetic stirrer bar. EtOH (1.0 mL) was added, and the resulting heterogeneous mixture was stirred at room temperature for the appropriate time. After stirring, the precipitate was washed with EtOH (2×4 mL) and dried under reduced pressure to afford the corresponding analytically pure aryl Au^I^ complex.

## Conflict of interest

*The authors declare no conflict of interest*.

## Supporting information

As a service to our authors and readers, this journal provides supporting information supplied by the authors. Such materials are peer reviewed and may be re‐organized for online delivery, but are not copy‐edited or typeset. Technical support issues arising from supporting information (other than missing files) should be addressed to the authors.

Supplementary
