# Supplementary material for: Mild and Efficient Synthesis of Diverse Organo‐AuI‐L Complexes in Green Solvents
Source: ChemSusChem. 2020 Mar 3;13(8):2032–7. doi: 10.1002/cssc.201903415 (PMC7277043; doi:10.1002/cssc.201903415)
Supplement: Supplementary file 1 — Supplementary [file CSSC-13-2032-s001.pdf]

ChemSusChem

Supporting Information

Mild and Efficient Synthesis of Diverse Organo-Au^{I}-L Complexes in Green Solvents

Fredric J. L. Ingner,[a] Ann-Cathrin Schmitt,[a] Andreas Orthaber,[b] Paul J. Gates,[c] and Lukasz T. Pilarski*[a]

Mild and Efficient Synthesis of Diverse Organo-Au(I)-L Complexes in Green Solvents

Supporting Information

Content

General Remarks 2
Preparation potassium triolboronate salts 3
Procedure 1 – From boronic acids 3
Procedure 2 – From pinacol esters 3
Procedure 3 – From boronic acids using 2-Me-THF 4
Potassium triolboronates: characterization data 4
Ph_3PAuAr complexes 10
Procedure A - preparation of Ph_3PAuAr complexes for spectroscopic yield determination 10
Procedure B - preparation of new Ph_3PAuAr complexes for characterisation 10
PPh_3AuAr complexes: characterization data 11
Phosphine and phosphite complexes 15
Procedure C - preparation of R_3P complexes. 15
R_3PAuAr complexes: characterization data 15
NHC-AuAr complexes 18
Procedure D - preparation of NHC complexes for spectroscopic quantification. 18
NHC-AuAr complexes: characterization data 18
Polyclic aromatic hydrocarbon-based Au(I) complexes 20
Polycyclic aromatic hydrocarbon-based Au(I) complexes: characterization data 20
$B(pin)_2NHC$-AuX complexes 22
Procedure E - preparation of $B(pin)_2NHC$-AuX complexes 22
$B(pin)_2NHC$-AuX complexes: characterization data 23
Mechanistic investigation 24
NMR Experiments 24
Competition experiment 28
References 31
Copies of NMR Spectra 32
***Potassium triolboronates* 33**
***Phosphine/Phosphite Au complexes* 71**
***NHC Au complexes* 103**
***Polycyclic aromatic hydrocarbon-based Au(I) complexes* 112**
***NHC-Bpin Au complexes* 123**

General Remarks

Unless otherwise stated, all reactions were performed under air with magnetic stirring. Thin layer chromatography (TLC) was carried out using aluminium-backed plates coated with Kieselgel 60 (0.20 mm, UV 254) and visualized under ultraviolet light (λ = 254 nm) or by thermal decomposition. Purification by column chromatography was performed using Kiesel gel 60 H silica gel (particle size 0.063-0.100 mm).

^{1}H, ^{11}B, ^{13}C, ^{19}F and ^{31}P NMR spectra were recorded on an Agilent 400-MR spectrometer (^{1}H: 399.97 MHz, ^{11}B: 128.33 MHz, ^{13}C: 100.58 MHz, ^{19}F: 376.32 MHz, ^{31}P: 161.92 MHz) at room temperature (25 °C). ^{1}H and ^{13}C shifts were referenced indirectly to tetramethylsilane using the residual solvent peaks of DMSO-*d6* (^{1}H: 2.50 ppm, ^{13}C: 39.52), C_6D_6 (^{1}H: 7.16 ppm, ^{13}C: 128.06 ppm) and chloroform-*d* (^{1}H: 7.26 ppm, ^{13}C: 77.16 ppm). ^{11}B, ^{19}F and ^{31}P chemical shifts were referenced to an external standard (^{11}B: 15% $BF_3.OEt_2$ ^{19}F: $CFCl_3$, ^{31}P: 85% H_3PO_4). Chemical shifts (δ) were denoted in ppm (parts per milion) and coupling constants (J) in Hz. For describing signal multiplicities, the following abbreviations were used: s (singlet), d (doublet), t (triplet), q (quartet), h (heptet) and m (multiplet). Unless otherwise stated, ^{31}P NMR spectra used for quantification was recorded with 30s relaxation delay and 32 scans (or equivalent to give a S/N > 250:1).

THF was freshly distilled from Na^0/benzophenone and stored over 4Å molecular sieves under Argon. Toluene was obtained from VWR as analytical grade Analar NORMAPUR. Ethanol was obtained from Solveco as 99.5% analytical grade.

Unless otherwise stated, all the other reagents were obtained commercially and used without further purification.

High-resolution electrospray ionisation mass spectrometry was performed on a micrOTOF II Focus instrument (Bruker Daltonics, Coventry, UK). High resolution nanospray ionisation was performed on a Synpat G2S instrument (Waters, Manchester, UK) using a Triversa chip based nanospray source (Advion Biosciences, Norwich, UK).

X-ray crystallography: All the measurements performed using graphite-monochromatized Mo Kα radiation using a Bruker D8 APEX-II equipped with a CCD camera. Data reduction was performed with SAINT.[1] Absorption corrections for the area detector were performed using SADABS.[2,3] The structure was solved by direct methods (SHELXT) and refined by full-matrix least-squares techniques against F2 using all data (SHELXL) using the OLEX2 suit of programs.[4] All non-hydrogen atoms were refined with anisotropic displacement parameters if not stated otherwise. Hydrogen atoms constrained in geometric positions to their parent atoms. The disorder of the thienyl group in 4n was modelled as a positional disorder restraining the two groups to be identical (site occupation factors 0.354:0.646).

Preparation potassium triolboronate salts

Procedure 1 – From boronic acids

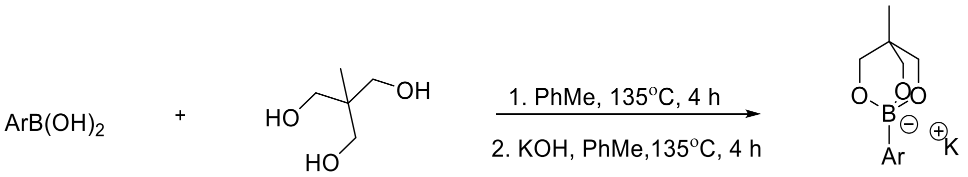

The cyclic triolboronates **2a**-**2u** were prepared according to a modified literature procedure.[5] Boronic acid (1.0 mmol) and trimethylolethane (1 mmol) were added to toluene (25 mL) in a round bottomed flask (50 mL). Water was removed by azeotropic distillation for 4 h at 135 °C (oil bath). Subsequently, the solution was cooled to rt. Powdered KOH (0.9 mmol) was added to the solution in one portion. Water was again removed by azeotropic distillation for 4 h at 135 °C (oil bath). The reaction was cooled to rt and the resulting precipitate was collected by filtration and washed with small amounts of acetone (5-10 mL). The potassium triolboronate products (**2a**-**2u**) were afforded as a bench-stable powders.

Procedure 2 – From pinacol esters

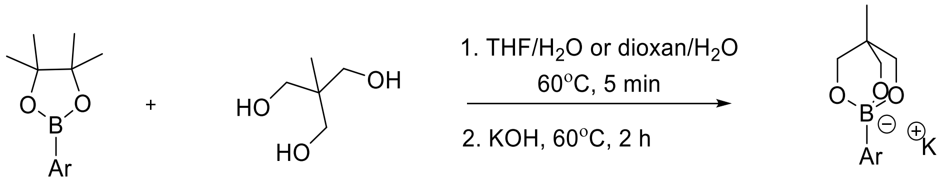

The cyclic triolboronates **2s-2t** were prepared according to a modified literature procedure.[6] Arylboronic acid pinacol ester (0.7 mmol, 1 equiv.) and trimethylolethane (0.7 mmol, 1 equiv.) were stirred in water/THF or water/dioxane (1:10, 7.0 mL, 0.1 M) at 60 ˚C for 5 min until the mixture turned homogeneous. Powdered KOH (0.6 mmol, 0.9 equiv) was added in one portion and the reaction was left to stir for 2 h at 60 ˚C. The resulting precipitate was collected and rinsed with acetone to afford the potassium triolboronate product as a bench-stable powder. (Products that were soluble in acetone were obtained via pentane trituration instead.)

Procedure 3 – From boronic acids using 2-Me-THF

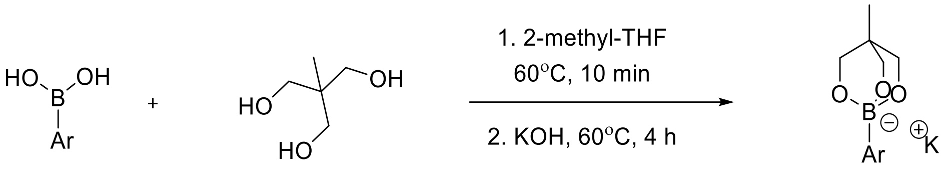

The cyclic triolboronates **2b, 2n and 2u** were prepared according to a modified literature procedure.[6] Arylboronic acid (1.0 mmol, 1 equiv.) and trimethylolethane (1.0 mmol, 1 equiv.) were stirred in 2-methyltetrahydrofuran (5.0 mL) at 60 ˚C for 10 minutes until the mixture turned homogeneous. Powdered KOH (0.9 mmol, 0.9 equiv) was added in one portion and the walls of the flask were rinsed with additional 2-methyltetrahydrofuran (2.0 mL). The reaction was left to stir for 4 h at 60 ˚C upon which a flocculent precipitate formed. The precipitate was collected and rinsed with acetone to afford the potassium triolboronate product as a bench-stable powder.

Potassium triolboronates: characterization data

2a) 3-tolyl potassium triolboronate

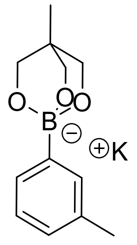

2a was synthesized according to Procedure 1.
Isolated yield: 72% (1.370 g, based on 7.35 mmol of boronic acid), colourless solid.
^{1}H NMR (400 MHz, DMSO-d_6) δ 7.14 – 7.11 (m, 1H), 7.09 – 7.05 (m, 1H), 6.85 (dd, J = 7.2 Hz, 1H), 6.74 – 6.69 (m, 1H), 3.55 (s, 6H), 2.16 (s, 3H), 0.46 (s, 3H). ^{13}C NMR (101 MHz, DMSO-d_6) δ 133.9, 133.7, 129.6, 125.9, 125.1, 74.0, 34.9, 21.9, 16.7. (C-B is not observed). HRMS: calcd for $C_{12}H_{16}O_3B$ [M]-: 219.1200; found: 219.1208

2b) Phenyl potassium triolboronate

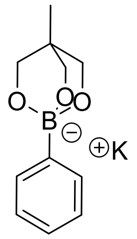

2b was synthesized according to Procedure 1 or Procedure 3.
Isolated yield: 75% (0.920 g, based on 5.00 mmol of boronic acid using Procedure 1) or 73% (0.160 g, based on 1.0 mmol of boronic acid using Procedure 3), colourless solid.
Spectral data is in accordance with previously reported values.[5]

2c) 2-tolyl potassium triolboronate

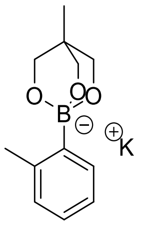

2c was synthesized according to Procedure 1.
Isolated yield: 14 % (0.070 g, based on 2.10 mmol of boronic acid), colourless solid. ^{1}H NMR (400 MHz, DMSO-d_6) δ 7.35 (d, J = 7.0 Hz, 1H), 6.85 – 6.64 (m, 3H), 3.57 (s, 6H), 2.33 (s, 3H), 0.47 (s, 3H). ^{13}C NMR (101 MHz, DMSO-d_6) δ 142.8, 134.1, 129.4, 128.1, 124.2, 69.4, 36.0, 22.6, 17.3. (C-B is not observed). HRMS: calcd for $C_{12}H_{16}O_3B$ [M]-: 219.1200, found: 219.1207

2d) 2-methoxyphenyl) potassium triolboronate

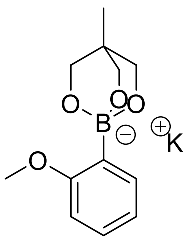

2d was synthesized according to Procedure 1.
Isolated yield: 56% (0.138 g, based on 1.00 mmol of boronic acid), colourless solid. ^{1}H NMR (400 MHz, DMSO-d_6) δ 7.40 (d, J = 7.6 Hz, 1H), 6.89 (dd, J = 7.6 Hz, 7.7 Hz, 1H), 6.63 – 6.51 (m, 2H), 3.57 (s, 3H), 3.53 (s, 6H), 0.45 (s, 3H). ^{13}C NMR (101 MHz, DMSO-d_6) δ 163.0, 135.6, 126.3, 119.4, 110.8, 73.7, 55.8, 34.8, 16.7. (C-B is not observed). HRMS: calcd for $C_{12}H_{16}O_4B$ [M]-: 235.1149; found: 235.1155

2e) (3-iodophenyl) potassium triolboronate

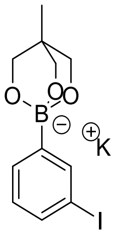

2e was synthesized according to Procedure 1.
Isolated yield: 76% (1.474 g, based on 5.80 mmol of boronic acid), colourless solid. ^{1}H NMR (400 MHz, DMSO-d_6) δ 7.62 (dd, J = 2.1, 1.1 Hz 1H), 7.28 (ddd, J = 7.2, 1.1, 1.1 Hz, 1H), 7.24 (ddd, 7.7, 2.1, 1.1 Hz, 1H), 6.79 (dd, 7.7, 7.2 Hz, 1H), 3.55 (s, 6H), 0.46 (s, 3H). ^{13}C NMR (101 MHz, DMSO-d_6) δ 141.6, 132.8, 131.5, 128.7, 94.8, 74.1, 34.9, 16.5. (C-B is not observed). HRMS: calcd for $C_{11}H_{13}O_3BI$ [M]-: 331.0010, found: 331.0150

2f) (4-methoxyphenyl) potassium triolboronate

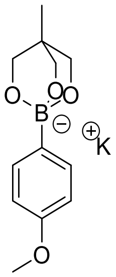

2f was synthesized according to Procedure 1.
Isolated yield: 95% (0.178 g, based on 1.00 mmol of boronic acid), colourless solid. Spectral data is in accordance with previously reported values.[7]

2g) (4-iodophenyl) potassium triolboronate

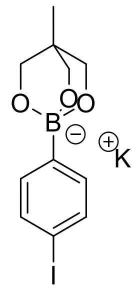

2g was synthesized according to Procedure 1.
Isolated yield: 96 % (0.142 g, based on 0.500 mmol of boronic acid), colourless solid. ^{1}H NMR (400 MHz, DMSO-d_6) δ 7.46 (d, J = 8.0 Hz, 2H), 7.23 (d, J = 8.0 Hz, 2H), 3.58 (s, 6H), 0.61 (s, 3H). ^{13}C NMR (101 MHz, DMSO-d_6) δ 135.5 (br, C_{Ar} signals overlap), 94.1, 70.9, 35.7, 17.0. (C-B is not observed). HRMS: calcd for $C_{11}H_{13}O_3BI$ [M]-: 331.0010; found: 331.0016

2h) (4-chlorophenyl) potassium triolboronate

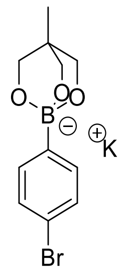

2h was synthesized according to Procedure 1.
Isolated yield: 73% (0.212 g, based on 1.00 mmol of boronic acid), colourless solid. ^{1}H NMR (400 MHz, DMSO-d_6) δ 7.23 (d, J = 8.0 Hz, 2H), 7.10 (d, J = 8.0 Hz, 2H), 3.54 (s, 6H), 0.45 (s, 3H). ^{13}C NMR (101 MHz, DMSO-d_6) δ 134.9, 128.6, 118.0, 74.1, 34.9, 16.6. (C-B is not observed); HRMS: calcd for $C_{11}H_{13}O_3BBr$ [M]-: 283.0149; found: 283.0142

2i) (4-chlorophenyl) potassium triolboronate

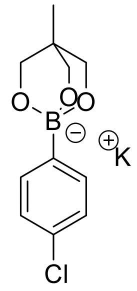

2i was synthesized according to Procedure 1.
Isolated yield: 44% (0.111 g, based on 1.00 mmol of boronic acid), colourless solid. ^{1}H NMR (400 MHz, DMSO-d_6) δ 7.28 (d, J = 8.2 Hz, 2H), 6.96 (d, J = 8.2 Hz, 2H), 3.54 (s, 6H), 0.45 (s, 3H). ^{13}C NMR (101 MHz, DMSO-d_6) δ 134.3, 129.1, 125.7, 74.0, 34.9, 16.6. (C-B is not observed). HRMS: calcd for $C_{11}H_{13}O_3BCl$ [M]-: 239.0654; found: 239.0662

2j) (4-vinylphenyl) potassium triolboronate

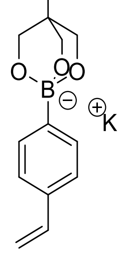

2j was synthesized according to Procedure 1.
Isolated yield: 77% (0.186 g, based on 1.00 mmol of boronic acid), colourless solid. ^{1}H NMR (400 MHz, DMSO-d_6) δ 7.26 (d, J = 7.5 Hz, 1H), 7.06 (d, J = 7.6 Hz, 1H), 6.58 (dd, J = 18.8, 11.5 Hz, 1H), 5.61 (d, J = 18.8 Hz, 1H), 5.00 (d, J = 11.5 Hz, 1H), 3.54 (s, 6H), 0.45 (s, 3H). ^{13}C NMR (101 MHz, DMSO-d_6) δ 138.5, 132.8, 124.0, 111.0, 74.0, 35.0 16.7. (C-B is not observed).; HRMS: calcd for $C_{13}H_{16}O_3B$ [M]-: 231.1200, found: 231.1209

2k) (4-trifluoromethylphenyl) potassium triolboronate

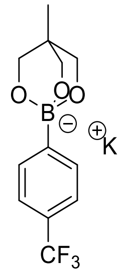

2k was synthesized according to Procedure 1.
Isolated yield: 46% (0.130 g, based on 1.00 mmol of boronic acid), colourless solid.
^{1}H NMR (400 MHz, DMSO-d_6) δ 7.50 (d, J = 7.4 Hz, 2H), 7.28 (d, J = 7.4 Hz, 2H), 3.57 (s, 6H), 0.47 (s, 3H). ^{13}C NMR (101 MHz, DMSO-d_6) δ 132.8, 125.8 (q, J_{C-F} = 271 Hz), 125.2 (q, J_{C-F} = 31 Hz), 122.2 (q, J_{C-F} = 4 Hz), 74.1, 35.0, 16.6. (C-B is not observed); ^{19}F (376 MHz, DMSO-d_6): = -60.10 ppm. HRMS: calcd for $C_{12}H_{13}O_3BF_3$ [M]-: 273.0918, found: 273.0929

2l) (3-pyridyl) potassium triolboronate

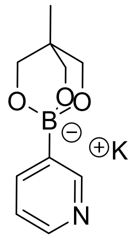

2l was synthesized according to Procedure 1.
Isolated yield: 63% (0.463 g, based on 3.00 mmol of boronic acid), colourless solid.
Spectral data is in accordance with previously reported values.[8]

2m) (1-phenylvinyl) potassium triolboronate

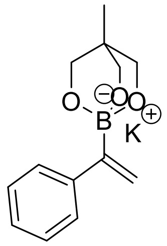

2m was synthesized according to Procedure 1.
Isolated yield: 48% (0.117 g, based on 1.00 mmol of boronic acid), colourless solid.
^{1}H NMR (400 MHz, DMSO-d_6) δ 7.48 – 7.44 (m, 2H), 7.08 – 7.03 (m, 2H), 6.98 – 6.92 (m, 1H), 5.09 (d, J = 5.8 Hz, 1H), 5.01 (d, J = 5.8 Hz, 1H), 3.49 (s, 6H), 0.42 (s, 3H). ^{13}C NMR (101 MHz, DMSO-d_6) δ 127.4, 126.5, 124.0, 73.4, 64.1, 34.4, 16.2. ppm (C-B is not observed). HRMS: calcd for $C_{13}H_{16}O_3B$ [M]-: 231.1200, found: 231.1206

2n) (3-thiophenyl) potassium triolboronate

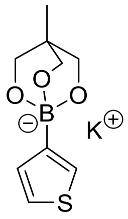

2n was synthesized according to Procedure 1 or Procedure 3.
Isolated yield: 87% (0.166 g, based on 1.00 mmol of boronic acid, using Procedure 3) or 70% (0.158 g, based on 1.00 mmol of boronic acid using Procedure 1), colourless solid.
^{1}H NMR (400 MHz, DMSO-d_6) δ 7.00 (dd, J = 4.7, 2.7 Hz, 1H), 6.92 (dd, J = 4.7, 1.0 Hz, 1H), 6.83 (dd, J = 2.7, 1.0 Hz, 1H), 3.52 (s, 6H), 0.44 (s, 3H). ^{13}C NMR (101 MHz, DMSO-d_6) δ 133.3, 124.3, 121.4, 73.9, 34.8, 16.7. ppm (C-B is not observed). HRMS: calcd for $C_9H_{12}O_3BS$ [M]-: 211.0607; found: 211.0615

2o) (2-furanyl) potassium triolboronate

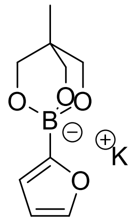

2o was synthesized according to Procedure 1.
Isolated yield: 78% (0.164 g, based on 1.00 mmol of boronic acid), colourless solid.
^{1}H NMR (400 MHz, DMSO-d_6) δ 7.34 – 7.22 (dd, J = 1.6, 0.8 Hz, 1H), 6.07 (dd, J = 3.0, 1.6 Hz, 1H), 5.88 (d, J = 3.0, 0.8 Hz, 1H), 3.51 (s, 6H), 0.44 (s, 3H). ^{13}C NMR (101 MHz, DMSO-d_6) δ 140.6, 110.1, 108.8, 73.7, 34.8, 16.5. (C-B is not observed). HRMS: calcd for $C_9H_{12}O_4B$ [M]-: 195.0838, found: 195.0838

2p) ferrocenyl potassium triolboronate

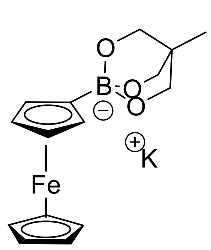

2pa was synthesized according to Procedure 1.
Isolated yield: 54% (0.173 g, based on 1.00 mmol of boronic acid), orange solid.
^{1}H NMR (400 MHz, DMSO-d_6) δ 3.96 (s, 5H), 3.82 (br s, 2H), 3.78 (br s, 2H), 3.53 (s, 6H), 0.46 (s, 3H). ^{13}C (100 MHz, DMSO-d_6) δ 73.6, 72.0, 67.8, 67.0, 34.9, 16.8. (C-B is not observed). HRMS: calcd for $C_{15}H_{18}O_3BFe$ [M]-: 313.0707, found: 313.0715

2q) (4-nitrophenyl) potassium triolboronate

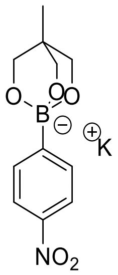

2q was synthesized according to Procedure 1.
Isolated yield: 44% (0.056 g, based on 0.500 mmol of boronic acid), brown solid.
^{1}H NMR (400 MHz, DMSO-d_6) δ 7.95 (d, J = 8.3 Hz, 2H), 7.67 (d, J = 8.3 Hz, 2H), 3.61 (s, 6H), 0.61 (s, 3H).^{13}C NMR (101 MHz, DMSO-d_6) δ 146.5, 133.5, 121.1, 70.6, 35.4, 16.5. (C-B is not observed). HRMS: calcd for $C_{11}H_{13}O_5BN$ [M]-: 250.0894, found: 250.0889

2r) (N-phenyl-3-carbazolyl) potassium triolboronate

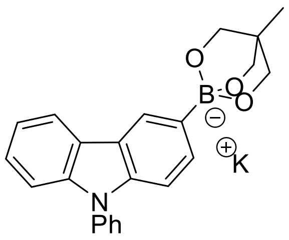

2r was synthesized according to Procedure 1.
Isolated yield: 79% (0.562 g, based on 2.00 mmol of boronic acid). Colourless solid. ^{1}H NMR (400 MHz, DMSO-d_6) δ 8.12 – 8.07 (m, 2H), 7.64 (dd, J = 7.7, 7.7 Hz, 2H), 7.57 (d, J = 7.7 Hz, 2H), 7.45 (dd, J = 7.7, 7.7 Hz, 2H), 7.37 – 7.25 (m, 2H), 7.18 (dd, J = 7.2, 7.2 Hz, 1H), 7.07 (d, J = 8.2 Hz, 1H), 3.62 (s, 6H), 0.49 (s, 3H). ^{13}C (100 MHz, DMSO-d_6): δ 139.9, 139.0, 138.3, 131.8, 130.4, 127.1, 126.7, 125.0, 124.5, 124.0, 121.7, 120.1, 119.7, 109.4, 107.0, 74.2, 31.1, 16.8. (C-B is not observed). HRMS: calcd for $C_{23}H_{21}O_3BN$ [M]-: 370.1624, found: 370.1637

2s) (9,10-Bis(2-naphthyl)anthracene-2-yl) potassium triolboronate

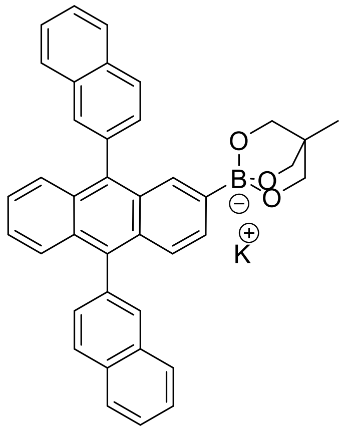

2s was synthesized according to Procedure 2.
Isolated yield: 56% (0.212 g, based on 0.700 mmol of (Ar-B(pin)).
Orange solid. ^{1}H NMR (400 MHz, DMSO-d_6) δ 8.16 (d, J = 8.2 Hz, 2H), 8.14 – 8.07 (m, 2H), 8.05 – 7.98 (m, 3H), 7.98 – 7.93 (m, 1H), 7.72 – 7.49 (m, 9H), 7.45 – 7.39 (m, 1H), 7.31 (d, J = 8.8 Hz, 1H), 7.25 – 7.19 (m, 2H), 3.46 (s, 6H), 0.40 (s, 3H). ^{13}C NMR (101 MHz, DMSO-d_6) δ 137.7, 137.3, 135.7, 135.6, 133.5, 133.2, 132.7, 130.3, 130.1, 130.1, 130.0, 129.9, 129.6, 129.3, 128.5, 128.5, 128.4, 128.4, 128.3, 128.2, 128.2, 126.9, 126.9, 126.7, 126.6, 126.5, 124.6, 124.5, 122.9, 71.1, 34.9, 16.6. (C-B is not observed). HRMS: calcd for $C_{39}H_{30}O_3B$ [M]-: 557.2300, found: 557.2309

2t) (2-triphenyl) potassium triolboronate

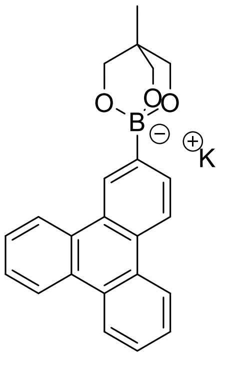

2t was synthesized according to Procedure 2.
Isolated yield: 67% (0.053 g, based on 0.200 mmol of Ar-B(pin)), colourless solid. ^{1}H NMR (400 MHz, DMSO-d_6) δ 8.75 – 8.63 (m, 5H), 8.39 (d, J = 8.0 Hz, 1H), 7.72 (d, J = 8.0 Hz, 1H), 7.69 – 7.54 (m, 4H), 3.66 (s, 6H), 0.53 (s, 3H).^{13}C (100 MHz, DMSO-d_6): δ 133.3, 130.9, 130.7, 129.1, 128.8, 127.5, 127.4, 126.7, 126.6, 126.6, 123.7, 123.7, 123.4, 123.4, 120.6, 74.1, 35.1, 16.7. (C-B is not observed). HRMS: calcd for $C_{23}H_{20}O_3B$ [M]-: 355.1515, found: 355.1528

2u) (1-pyrenyl) potassium triolboronate

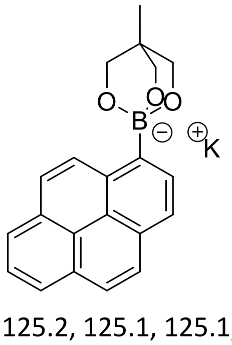

2u was synthesized according to Procedure 1 or Procedure 3.
Isolated yield: 91% (0.301 g, based on 0.100 mmol of boronic acid, using Procedure 3) or 55% (0.182 g, based on 0.100 mmol of boronic acid, using Procedure 1), colourless solid.
^{1}H NMR (400 MHz, DMSO-d_6) δ 9.21 (d, J = 9.2 Hz, 1H), 8.26 (d, J = 7.6 Hz, 1H), 8.04 (dd, J = 7.6, 1.9 Hz, 2H), 8.02 – 7.89 (m, 3H), 7.87 (dd, J = 7.6, 7.6 Hz, 1H), 7.83 (d, J = 9.2 Hz, 1H), 3.77 (s, 6H), 0.58 (s, 3H).
^{13}C (100 MHz, DMSO-d_6) δ 134.3, 133.3, 131.6, 131.3, 131.3, 128.6, 128.2, 125.2, 125.1, 125.1, 124.1, 123.5, 123.4, 123.1, 123.1, 74.3, 35.2, 16.8. (C-B is not observed). HRMS: calcd for $C_{21}H_{18}O_3B$ [M]-: 329.1358, found: 329.1366

Ph_3PAuAr complexes

Procedure A - preparation of Ph_3PAuAr complexes for spectroscopic yield determination

Ph_3PAuCl (0.1 mmol, 1 equiv.) and potassium triolboronate (0.1 mmol, 1 equiv.) were added to a microwave vial fitted with a stirring bar. Solvent (1 mL) was added and the mixture was stirred at room temperature for 24 h. After stirring, the suspension was diluted in H_2O (5 mL) and extracted with DCM* (3 x 5 mL). The combined organic phases were collected, dried over Na_2SO_4 and filtered. Solvent was removed by rotary evaporation to afford the arylgold complex. Spectroscopic yields were determined using ^{31}P NMR spectroscopy.

Yield determination was readily performed by ^{31}P NMR as the reactions proved very clean; only expected products and starting materials were present at the end of the reaction. R_3PAuCl and R_3PAuAr species proved insoluble in H_2O and phosphorus-containing species were recovered by extraction in DCM.* Yield determination was performed by comparing integral ratios of R_3PAuCl and R_3PAuAr. ^{31}P NMR spectra used for quantification were recorded using 30 s relaxation delay and 32 scans (or equivalent to give a S/N > 250:1).

**DCM was used only to ensure full recovery of R_3PAu species for accurate quantification as part of the method development. For synthetic applications, halogenated solvents can be omitted (See Procedure B).*

Procedure B - preparation of new Ph_3PAuAr complexes for characterisation

Ph_3PAuCl (0.1 mmol, 1 equiv.) and potassium triolboronate (0.3 mmol, 3 equiv.) were added to a microwave vial fitted with a stirring bar. EtOH (1 mL) was added and the heterogeneous mixture was stirred at room temperature for 24 h. After stirring, the precipitate was washed with EtOH (2x4 mL) and dried under reduced pressure to afford the arylgold complex.

PPh_3AuAr complexes: characterization data

4a) 3-tolyl(triphenylphosphine)gold

4a was synthesized according to Procedure A. Reference sample for characterization was prepared according to Procedure B.

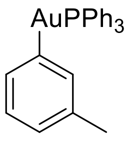

Spectroscopic yield: 97 % (EtOH). 85% (H_2O) Isolated yield: 90% (50 mg, 0.1 mmol scale, 1 equiv. Ar-B(triol)K, colourless solid). ^{1}H NMR (400 MHz, Chloroform-*d*) δ 7.66 – 7.57 (m, 6H), 7.54 – 7.44 (m, 10H), 7.39 (d, J = 7.4 Hz, 1H), 7.22 (dd, J = 7.4, 7.4 Hz, 1H), 6.93 (d, J = 7.4 Hz, 1H), 2.32 (s, 3H). ^{13}C NMR (101 MHz, Chloroform-*d*) ^{13}C NMR (101 MHz, Chloroform-*d*) δ 171.6 (d, J_{C-P} = 116 Hz), 140.2, 136.5 (d, J_{C-P} = 5 Hz), 136.4, 134.4 (d, J_{C-P} = 14 Hz), 131.1 (d, J_{C-P} = 49 Hz), 131.1, 129.0 (d, J_{C-P} = 11 Hz), 127.4 (d, J_{C-P}= 5 Hz), 126.7, 21.8. ^{31}P NMR (162 MHz, Chloroform-*d*) δ 43.51. HRMS-Nanospray calcd for $C_{25}H_{22}AuP$ $[M+K]^+$: 589.0762; found: 589.0754

4b) Phenyl(triphenylphosphine)gold

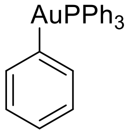

4b was synthesized according to Procedure A. Spectroscopic data is in accordance with literature values.[9] Spectroscopic yield: 88 % (EtOH); 54% (H_2O)

4c) 2-tolyl(triphenylphosphine)gold

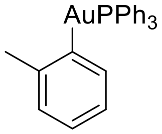

4c was synthesized according to Procedure A. Spectroscopic data is in accordance with literature values.[10] Spectroscopic yield: 62 % (EtOH).

4d) 2-Methoxyphenyl(triphenylphosphine)gold

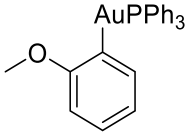

4d was synthesized according to Procedure A. Spectroscopic data is in accordance with literature values.[11] Spectroscopic yield: 78% (EtOH), 56% (H_2O);

4e) 3-Iodophenyl(triphenylphosphine)gold

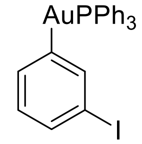

4e was synthesized according to Procedure A. Reference sample for characterization was prepared according to Procedure B.

Spectroscopic yield: 85% (EtOH). 56% (H_2O) Isolated yield: 97% (32 mg, colourless solid, 3 equiv. Ar-B(triol)K). ^{1}H NMR (400 MHz, Chloroform-*d*) δ 7.94 (d, J = 5.3 Hz, 1H), 7.62 – 7.44 (m, 16H), 7.41 (d, J = 7.6 Hz, 1H), 7.04 (dd, J = 7.6, 7.6 Hz, 1H). ^{13}C NMR (101 MHz, Chloroform-*d*) δ 175.6 (d, $J_{C\text{-}P}$ = 117 Hz), 147.7, 138.1, 134.6, 134.3 (d, $J_{C\text{-}P}$ = 14 Hz), 131.2 (d, $J_{C\text{-}P}$ = 2 Hz), 130.7 (d, $J_{C\text{-}P}$ = 50 Hz), 129.5 (d, $J_{C\text{-}P}$ = 7 Hz), 129.3, 129.1 (d, $J_{C\text{-}P}$ = 11 Hz). ^{31}P NMR (162 MHz, Chloroform-*d*) δ 42.63. HRMS- Nanospray calcd for $C_{24}H_{19}AuPI$ [M-H]-: 660.9862; found: 660.9871

4f) 4-Methoxyphenyl(triphenylphosphine)gold

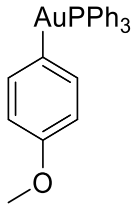

4f was synthesized according to Procedure A. Spectroscopic data is in accordance with literature values.[12] Spectroscopic yield: 88 % (EtOH); 65% (H_2O)

4g) 4-Iodophenyl(triphenylphosphine)gold

4g was synthesized according to Procedure A. Reference sample for characterization was prepared according to Procedure B.

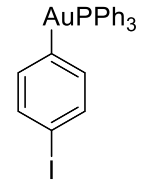

Spectroscopic yield: 93 % (EtOH). 55% (H_2O) Isolated yield: 91% (30 mg, colourless solid, 3 equiv Ar-B(triol)K). ^{1}H NMR (400 MHz, Chloroform-*d*) δ 7.62 – 7.54 (m, 8H), 7.52 – 7.42 (m, 9H), 7.37 – 7.30 (m, 2H). ^{13}C NMR (101 MHz, Chloroform-*d*) δ 171.2 (d, $J_{C\text{-}P}$ = 117 Hz), 141.1, 137.5, 136.1 (d, $J_{C\text{-}P}$ = 6 Hz), 134.3 (d, $J_{C\text{-}P}$ = 14 Hz), 131.2, 130.8 (d, $J_{C\text{-}P}$ = 49 Hz), 129.1 (d, $J_{C\text{-}P}$ = 11 Hz). ^{31}P NMR (162 MHz, Chloroform-*d*) δ 43.61. HRMS-Nanospray calcd for $C_{24}H_{19}AuIP$ [M-H]-: 660.9862; found: 660.9869

4h) 4-Bromophenyl(triphenylphosphine)gold

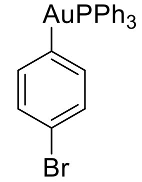

4h was synthesized according to Procedure A. Spectroscopic data is in accordance with literature values.[13] Spectroscopic yield: 82% (EtOH); 60% (H_2O)

4i) 4-Chlorophenyl(triphenylphosphine)gold

4i was synthesized according to Procedure A. Reference sample for characterization was prepared according to Procedure B.

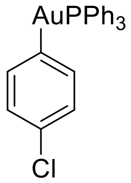

Spectroscopic yield: 75% (EtOH). 52% (H_2O) Isolated yield: 89% (25 mg, colourless solid, 3 equiv Ar-B(triol)K). ^{1}H NMR (400 MHz, Chloroform-*d*) δ 7.65 – 7.54 (m, 6H), 7.54 – 7.44 (m, 13H).; ^{13}C NMR (101 MHz, Chloroform-*d*) 140.3, 134.3 (d, *J* = 14 Hz), 131.2 (d, *J* = 2 Hz), 129.0 (d, *J* = 11 Hz), 127.3 (d, *J* = 6 Hz). (Au-C not observed) ^{31}P (162 MHz, Chloroform-*d*): = 43.46 ppm. HRMS- Nanospray calcd for $C_{24}H_{19}AuCl$ [M-H]-: 569.0506; found: 569.0513

4j) 4-Vinylphenyl(triphenylphosphine)gold

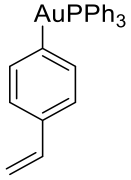

4j was synthesized according to Procedure A. Spectroscopic data is in accordance with literature values.[12] Spectroscopic yield: 83 % (EtOH)

4k) 4-(Trifluoromethyl)phenyl(triphenylphosphine)gold

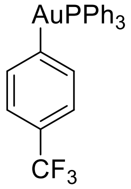

4k was synthesized according to Procedure A. Spectroscopic data is in accordance with literature values.[9] Spectroscopic yield: 41% (EtOH)

4m) (1-Vinyl)phenyl(triphenylphosphine)gold

4m was synthesized according to Procedure A. Reference sample for characterization was prepared according to Procedure B. Rapid degradation of **4m** in is observed in $CDCl_3$.

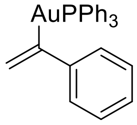

Spectroscopic yield: 47 % (EtOH). 45% (H_2O). Isolated yield: 96% (27 mg, colourless solid, 3 equiv Ar-B(triol)K). ^{1}H NMR (400 MHz, Chloroform-*d*) δ 7.61 – 7.40 (m, 18H), 7.33 – 7.28 (m, 1H), 7.12 (dd, *J* = 7.4 Hz, 1H), 6.12 (dd, *J* = 15.8, 3.6 Hz, 1H), 5.36 (dd, *J* = 7.4, 3.6 Hz, 1H). ^{13}C NMR (101 MHz, Chloroform-*d*) δ 134.2 (d, *J* = 14 Hz), 131.9 (d, *J* = 3 Hz), 129.2 (d, *J* = 12 Hz), 128.4, 127.9, 127.47, 122.7, 110.0. (Au-C not observed). ^{31}P NMR (162 MHz, Chloroform-*d*) δ 43.08. HRMS- Nanospray calcd for $C_{26}H_{22}AuP$ [M-H]-: 561.1052 ; found: 561.1064

4n) 3-Thiophenyl(triphenylphosphine)gold

4n was synthesized according to Procedure A. Reference sample for characterization was prepared according to Procedure B.

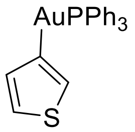

Spectroscopic yield: 87 % (EtOH), 84% (H2O). Isolated yield: 96% (26 mg, colourless solid, 3 equiv Ar-B(triol)K). ^{1}H NMR (400 MHz, Chloroform-*d*) δ 7.66 – 7.54 (m, 6H), 7.54 – 7.41 (m, 10H), 7.34 (d, *J* = 4.8 Hz, 1H), 7.32 – 7.28 (m, 1H). ^{13}C NMR (101 MHz, Chloroform-*d*) δ 135.2 (d, *J* = 4 Hz), 134.4 (d, *J* = 14 Hz), 131.2 (d, *J* = 3 Hz), 130.6, 130.0 (d, *J* = 6 Hz), 129.1 (d, *J* = 11 Hz), 122.7 (d, *J* = 8 Hz). (Au-C not observed). ^{31}P NMR (162 MHz, Chloroform-*d*) δ 44.05. HRMS- Nanospray calcd for $C_{22}H_{18}AuPS$ [M-H]-: 541.0460; found: 541.0453

4o) 2-Furanyl(triphenylphosphine)gold

4o was synthesized according to Procedure A. Spectroscopic data is in accordance with literature

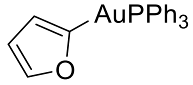

values.[9] Spectroscopic yield: 60 % (EtOH).

4p) Ferrocenyl(triphenylphosphine)gold

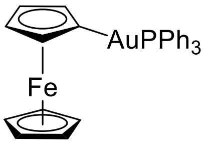

4p was synthesized according to Procedure A. Spectroscopic data is in accordance with literature values.[14] Spectroscopic yield: 53 % (EtOH); Isolated yield (28mg, pale orange solid, 3.0 equiv. boronate used).

Phosphine and phosphite complexes

Procedure C - preparation of R_3P complexes.

R_3PAuCl (0.1 mmol, 1.0 equiv.) and potassium triolboronate (0.1 mmol, 1.0 equiv.) were added to a microwave vial fitted with a stirring bar. EtOH (1 mL, 99.5%) was added and the mixture was stirred at room temperature for 24 h. After stirring, the solid was washed with EtOH (2 mL) and left to settle. The remaining solution was decanted and the solid was dried under reduced pressure. Unless otherwise stated, the target complex was afforded as a colourless solid without further purification. Spectroscopic yields were determined using ^{31}P NMR spectroscopy.

R_3PAuAr complexes: characterization data

5a´) Chloro(triethylphosphite)gold

Synthesis of **5a’** was performed according to a modified literature procedure.[15]

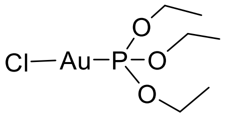

Me_2SAuCl (59 mg, 0.2 mmol) was dissolved in anhydrous DCM in an oven dried Schlenk tube under argon atmosphere. A solution of triethyl phosphite (0.5 M in DCM, 33 mg, 0.2 mmol) was added dropwise under stirring and the reaction was stirred in darkness for 14h. The reaction mixture was concentrated *in vacuo* and the product **5a’**was obtained as a colourless oil. Isolated yield 93% (80mg, colourless oil). ^1H NMR (400 MHz, Chloroform-*d*) δ 4.13 (dq, $^3J_{P\text{-}H}$ = 10.0 Hz, $^3J_{H\text{-}H}$ 7.1 Hz, 6H), 1.33 (dd, J = 7.1, 7.1 Hz, 9H). ^{13}C NMR (101 MHz, Chloroform-*d*) δ 63.1, 16.1 (d, $J_{C\text{-}P}$ = 7 Hz). ^{31}P NMR (162 MHz, Chloroform-*d*) δ 117.8. HRMS-Nanospray calcd for $C_6H_{15}AuClO_3P$ [M-Cl]+: 363.0424; found: 363.0428

5a) 3-Tolyl(tricyclohexylphosphine)gold

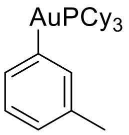

Synthesis of **5a** was performed according to Procedure C. Spectroscopic data is in accordance with literature values.[16] Isolated yield 96% (55 mg, 1.2 equiv. triolboronate).

5b) 4-Vinylphenyl(triethylphosphine)gold

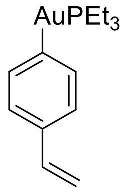

Synthesis of **5b** was performed according to Procedure C. Spectroscopic data is in accordance with literature values.[17] Spectroscopic yield 82%.

5c) 3-Tolyl(tri(4-trifluoromethylphenyl)phosphine)gold

Synthesis of **5c** was performed according to Procedure C.

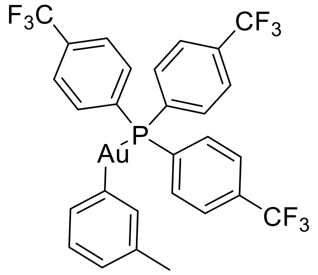

Isolated yield 84% (63 mg, pale yellow solid).
^{1}H NMR (400 MHz, Chloroform-*d*) δ 7.82 – 7.66 (m, 12H), 7.40 – 7.36 (m, 1H), 7.34 (d, *J* = 7.2 Hz, 1H), 7.21 (dd, *J* = 7.2, 7.2 Hz, 1H), 6.94 (d, *J* = 7.2 Hz, 1H), 2.30 (s, 3H). ^{13}C NMR (101 MHz, Chloroform-*d*) δ 139.9, 136.8, 136.1, 134.8, 134.7, 133.9 (qd, J_{C-F} = 32, 2 Hz), 128.6 (d, J_{C-P} = 82 Hz), 127.5, 127.2, 126.3 (dq, *J* = 11, 4 Hz), 123.3 (d, J_{C-F} = 273 Hz), 21.7. ^{19}F NMR (376 MHz, Chloroform-*d*) δ -63.26. ^{31}P NMR (162 MHz, Chloroform-*d*) δ 43.68.
HRMS-Nanospray calcd for $C_{28}H_{19}AuF_9P$ [M+K]+: 793.0383; found: 793.0395

5d) 4-Methoxyphenyl(triethylphosphite)gold

Synthesis of **5d** was performed according to Procedure C.

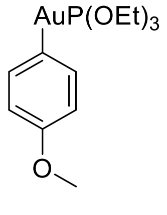

Isolated yield 85% (72 mg, 0.15 mmol scale, colourless oil). ^{1}H NMR (400 MHz, Chloroform-*d*) δ 7.40 (d, *J* = 8.5 Hz, 2H), 6.88 (d, *J* = 8.5 Hz, 2H), 4.18 (dq, $^2J_{P-H}$ = 9.8 Hz, $^3J_{H-H}$ 7.1 Hz, 6H), 3.77 (s, 3H), 1.37 (t, *J* = 7.1 Hz, 9H). ^{13}C NMR (126 MHz, Chloroform-*d*) δ 158.1, 140.1, 113.2, 69.0, 61.9, 54.9, 16.5 (d, J_{C-P} = 7 Hz). ^{31}P NMR (162 MHz, Chloroform-*d*) δ 156.96. HRMS-Nanospray calcd for $C_{13}H_{22}AuO_4P$ [M+K]+: 509.0558; found: 509.0556

5e) [1,1′-Bis(1-pyrenyl(diphenylphosphino)gold)ferrocene]

Synthesis of **5e** was performed according to Procedure C.

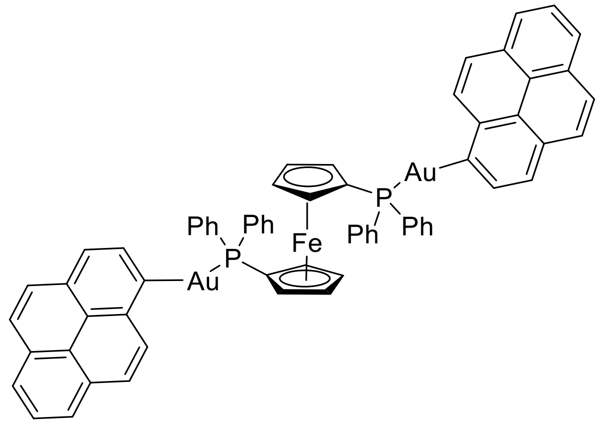

Isolated yield 97% (66 mg, orange solid, 0.05 mmol scale[(dppf)(AuCl)$_2$], 2 equiv. triolboronate). ^{1}H NMR (400 MHz, Chloroform-*d*) δ 8.80 (d, J = 9.0 Hz, 2H), 8.34 (d, J = 7.5 Hz, 2H), 8.16 – 8.09 (m, 4H), 8.09 – 8.02 (m, 4H), 8.02 – 7.90 (m, 6H), 7.73 – 7.61 (m, 8H), 7.48 – 7.36 (m, 12H), 4.95 (br s, 4H), 4.51 (br s, 4H). ^{13}C NMR (101 MHz, Chloroform-*d*) δ 139.9, 137.3, 134.1, 133.7 (d, J_{C-P} = 14 Hz), 132.3 (d, J_{C-P} = 51 Hz), 131.6 (d, J_{C-P} = 5 Hz), 131.2, 129.2, 128.9 (d, J_{C-P} = 11 Hz), 127.9, 125.9, 125.6 (d, J_{C-P} = 9 Hz), 125.1, 124.1, 123.6, 123.4, 74.8 (d, J_{C-P} = 12 Hz), 73.0 (d, J_{C-P} = 59 Hz). HRMS-Nanospray calcd for $C_{66}H_{46}Au_2FeP_2$ [M-$C_{16}H_9$]+: 1149.1062; found: 1149.1

NHC-AuAr complexes

Procedure D - preparation of NHC complexes for spectroscopic quantification.

IPrAuCl (0.1 mmol, 1.0 equiv.) and potassium triolboronate (0.12 mmol, 1.2 equiv.) were added to a microwave vial fitted with a stirring bar. EtOH (1 mL, 99.5%) was added and the mixture was stirred at room temperature for 24 h. After stirring, the solid was washed with EtOH (2 mL) and left to settle. Remaining solution was decanted and solid was dried under vacuum. The target complex was afforded as a colourless solid without further purification.

NHC-AuAr complexes: characterization data

5f) [N,N-Bis(2,6-diisopropylphenyl)imidazol-2-yl](3-iodophenyl)gold(I)

Synthesis of **5f** was performed according to Procedure D.

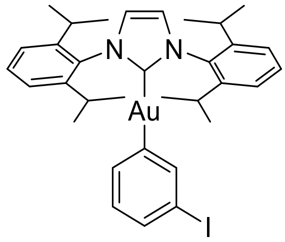

Isolated yield 97% (76 mg, colourless solid). ^{1}H NMR (400 MHz, Chloroform-*d*) *δ* 7.48 (dd, *J* = 7.8, 7.8 Hz, 2H), 7.41 (ddd, *J* = 2.0, 1.1, 0.5 Hz, 1H), 7.29 (d, *J* = 7.8 Hz, 4H), 7.20 (ddd, *J* = 7.7, 2.0, 1.1 Hz, 1H), 7.15 (s, 2H), 7.00 (ddd, *J* = 7.1, 1.1, 0.5 Hz, 1H), 6.75 (ddd, *J* = 7.7, 7.1, 0.5 Hz, 1H), 2.65 (hept, *J* = 6.9 Hz, 4H), 1.39 (d, *J* = 6.9 Hz, 12H), 1.25 (d, *J* = 6.9 Hz, 12H). ^{13}C NMR (101 MHz, Chloroform-*d*) *δ* 195.3, 173.6, 148.3, 145.7, 139.0, 134.4, 132.9, 130.2, 128.6, 123.9, 122.84, 96.8, 28.7, 24.5, 23.9. HRMS-Nanospray calcd for $C_{33}H_{40}AuIN_2$ [M-H]-: 787.1829; found: 787.1836

5g) [N,N-Bis(2,6-diisopropylphenyl)imidazol-2-yl](3-bromophenyl)gold(I)

Synthesis of **5g** was performed according to Procedure D.

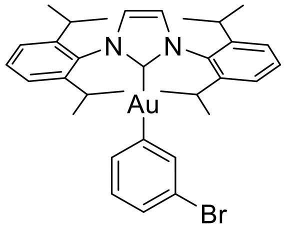

Isolated yield 82% (61 mg, colourless solid). ^{1}H NMR (400 MHz, Chloroform-*d*) *δ* 7.49 (dd, *J* = 7.8, 7.8 Hz, 2H), 7.30 (d, *J* = 7.8 Hz, 4H), 7.20 (d, *J* = 2.3 Hz, 1H), 7.16 (s, 2H), 7.00 – 6.93 (m, 2H), 6.89 (dd, *J* = 7.5, 7.5 Hz, 1H), 2.66 (hept, *J* = 7.0 Hz, 4H), 1.40 (d, *J* = 6.9 Hz, 12H), 1.26 (d, *J* = 6.9 Hz, 12H). ^{13}C NMR (101 MHz, Chloroform-*d*) *δ* 195.5, 173.0, 145.7, 142.2, 138.5, 134.4, 130.2, 128.1, 126.9, 123.9, 122.8, 122.8, 28.7, 24.5, 23.9. HRMS-Nanospray calcd for $C_{33}H_{40}AuBrN_2$ [M-H]-: 739.1968; found: 739.1979

5h) [N,N-Bis(2,6-diisopropylphenyl)imidazol-2-yl](4-chlorophenyl)gold(I)

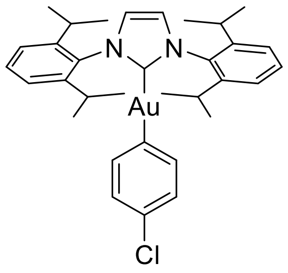

Synthesis of **5h** was performed according to Procedure D. Spectroscopic data is in accordance with literature values.[18]
Isolated yield 91% (64 mg, colourless solid)

5i) [N,N-Bis(2,6-diisopropylphenyl)imidazol-2-yl](4-methoxyhenyl)gold(I)

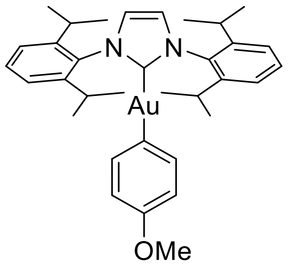

Synthesis of **5i** was performed according to Procedure D. Spectroscopic data is in accordance with literature values.[18]
Spectroscopic yield 50% (Quantified using ^{1}H NMR with 1,3,5-trimethoxybenzene as internal standard).

5j) [N,N-Bis(2,6-diisopropylphenyl)imidazol-2-yl](4-iodophenyl)gold(I)

Synthesis of **5j** was performed according to Procedure D.

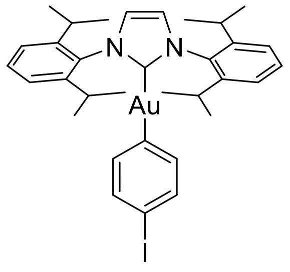

Isolated yield 77% (222 mg, 0.4 mmol, colourless solid). ^{1}H NMR (400 MHz, Chloroform-*d*) δ 7.47 (dd, *J* = 7.8, 7.8 Hz, 2H), 7.31 (d, *J* = 7.9 Hz, 2H), 7.28 (d, *J* = 7.8 Hz, 4H), 7.15 (s, 2H), 6.81 (d, *J* = 7.9 Hz, 2H), 2.64 (hept, *J* = 7.1 Hz, 4H), 1.38 (d, *J* = 6.9 Hz, 12H), 1.24 (d, *J* = 6.9 Hz, 12H). ^{13}C NMR (126 MHz, Chloroform-*d*) δ 196.4, 168.6, 145.7, 142.1, 135.3, 134.5, 130.2, 124.0, 122.8, 90.2, 28.8, 24.5, 23.9. HRMS-Nanospray calcd for $C_{33}H_{40}AuIN_2$ [M-H]-: 787.1829; found: 787.1836

5k) [N,N-Bis(2,6-diisopropylphenyl)imidazol-2-yl](3-thiophenyl)gold(I)

Synthesis of **5k** was performed according to Procedure D.

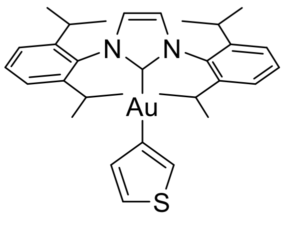

Isolated yield 87% (58 mg, colourless solid).^{1}H NMR (400 MHz, Chloroform-*d*) δ 7.46 (dd, *J* = 7.8, 7.8 Hz, 2H), 7.27 (d, *J* = 7.8 Hz, 4H), 7.24 (dd, *J* = 4.5, 2.6 Hz, 1H), 7.14 (s, 2H), 6.86 (dd, *J* = 4.5, 0.9 Hz, 1H), 6.71 (dd, *J* = 2.6, 0.9 Hz, 1H), 2.67 (hept, *J* = 6.9 Hz, 4H), 1.40 (d, *J* = 6.9 Hz, 12H), 1.24 (d, *J* = 6.9 Hz, 12H). ^{13}C NMR (101 MHz, Chloroform-*d*) δ 196.1, 164.4, 145.7, 136.5, 134.5, 130.1, 128.4, 123.9, 122.7, 121.4, 28.7, 24.4, 23.9. HRMS-Nanospray calcd for $C_{31}H_{39}AuN_2S$ [M-H]-: 667.2427; found: 667.2439

Polyclic aromatic hydrocarbon-based Au(I) complexes

Polycyclic aromatic hydrocarbon-based Au(I) complexes: characterization data

5l) 2-Triphenylene(triphenylphosphine)gold

5l was synthesized according to Procedure A. Reference sample for characterization was prepared according to Procedure B.

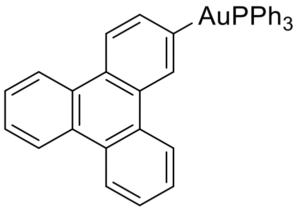

Spectroscopic yield: 67% (EtOH). Isolated yield: 91% (31 mg, colourless solid, 3 equiv B(triol)K). ^{1}H NMR (400 MHz, Chloroform-*d*) δ 8.96 (m, 1H), 8.86 – 8.78 (m, 1H), 8.72 – 8.62 (m, 3H), 8.58 (d, J = 8.1 Hz, 1H), 7.94 (d, J = 8.1 Hz, 1H), 7.73 – 7.56 (m, 10H), 7.51 (m, 9H). ^{13}C NMR (101 MHz, Chloroform-*d*) δ 171.6 (d, J_{C-P} = 118 Hz), 139.0, 134.4 (d, J_{C-P} = 14 Hz), 133.9, 131.2, 130.8 (d, J_{C-P} = 23 Hz), 129.8, 129.5 (d, J_{C-P} = 24 Hz), 129.1 (d, J_{C-P} = 11 Hz), 128.8, 128.0, 127.2, 126.8 (d, J_{C-P} = 2 Hz), 126.3, 123.5, 123.3, 123.1 (d, J_{C-P} = 2 Hz), 121.5. ^{31}P NMR (162 MHz, Chloroform-*d*) δ 43.50. HRMS- Nanospray calcd for $C_{36}H_{26}AuP$ [M-H]-: 685.1365; found: 685.1375

5m) 1-Pyrenyl(triphenylphosphine)gold

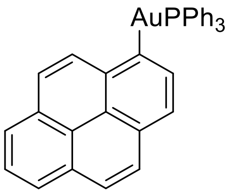

5m was synthesized according to Procedure A. Spectroscopic data is in accordance with literature values.[19] Spectroscopic yield: >99% (EtOH), Isolated yield: 97% (64 mg, colourless solid, 1 eq Ar-B(triol)K)

5n) [N,N-Bis(2,6-diisopropylphenyl)imidazol-2-yl](1-pyrenyl)gold(I)

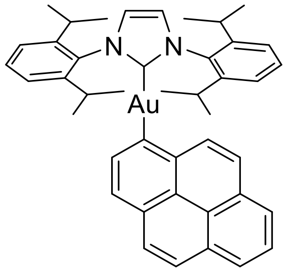

Synthesis of **5n** was performed according to Procedure D. Spectroscopic data is in accordance with literature values.[20]
Isolated yield 99% (78 mg, colourless solid)

5o) 9,10-Bis(2-naphthyl)anthracene-2-yl(triphenylphosphine)gold

5o was synthesized according to Procedure A. Reference sample for characterization was prepared according to Procedure B.

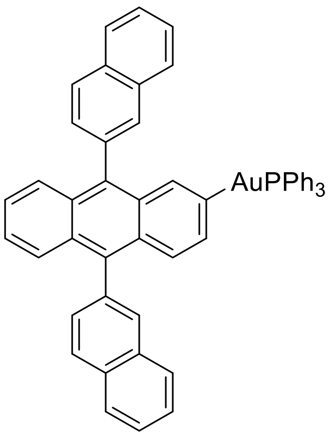

Spectroscopic yield: 81% (EtOH). Isolated yield: 91% (49 mg, yellow solid, 3 equiv B(triol)K). ^{1}H NMR (400 MHz, Chloroform-*d*) *δ* 8.13 – 7.91 (m, 9H), 7.77 – 7.65 (m, 6H), 7.64 – 7.50 (m, 10H), 7.48 – 7.35 (m, 8H), 7.29 – 7.22 (m, 3H). ^{13}C NMR (101 MHz, Chloroform-*d*) *δ* 170.1 (d, *J* = 118 Hz), 137.7, 137.5, 137.3, 136.8, 136.3, 135.9, 134.4 (d, $J_{C\text{-}P}$ = 14 Hz), 133.5 (d, $J_{C\text{-}P}$ = 8 Hz), 132.7 (d, $J_{C\text{-}P}$ = 2 Hz), 131.2, 131.1 (d, $J_{C\text{-}P}$ = 2.3 Hz), 130.7, 130.5, 130.3, 130.0, 129.9, 129.8, 129.0 (d, $J_{C\text{-}P}$ = 11Hz), 128.3, 128.2, 127.9 (d, $J_{C\text{-}P}$ = 2 Hz), 127.7, 127.1, 126.9, 126.2, 126.0 (d, $J_{C\text{-}P}$ = 7 Hz), 125.8, 124.5 (d, $J_{C\text{-}P}$ = 6 Hz), 124.2 (d, $J_{C\text{-}P}$ = 6 Hz). ^{31}P NMR (162 MHz, Chloroform-*d*) *δ* 43.26. HRMS-Nanospray calcd for $C_{52}H_{36}AuP$ [M-H]-: 887.2148; found: 887.2166

5p) 3-(N-phenyl)-carbazolyl(triphenylphosphine)gold

5p was synthesized according to Procedure A. Reference sample for characterization was prepared according to Procedure B.

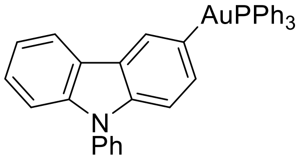

Spectroscopic yield: 86% (EtOH). Isolated yield: 94% (33 mg, colourless solid, 3 equiv Ar-B(triol)K). ^{1}H NMR (400 MHz, Chloroform-*d*) *δ* 8.41 (s, 1H), 8.15 (d, *J* = 7.7 Hz, 1H), 7.72 – 7.56 (m, 10H), 7.54 – 7.45 (m, 10H), 7.44 – 7.38 (m, 3H), 7.35 (dd, *J* = 7.5, 7.5 Hz, 1H), 7.23 (dd, *J* = 7.5, 7.5 Hz, 1H). ^{13}C NMR (101 MHz, Chloroform-*d*) *δ* 140.3, 139.9, 138.3, 137.1, 134.4 (d, $J_{C\text{-}P}$ = 14 Hz), 130.6, 129.9 (d, $J_{C\text{-}P}$ = 13 Hz), 129.6, 129.1 (d, $J_{C\text{-}P}$ = 11 Hz), 127.1 (d, $J_{C\text{-}P}$ = 9 Hz), 127.0, 126.8, 125.9, 124.9, 124.3, 123.2, 120.3, 120.1, 119.9, 119.3, 109.7, 109.3, 108.9. (Au-C not observed). ^{31}P NMR (162 MHz, Chloroform-*d*) *δ* 43.75. HRMS-Nanospray calcd for $C_{36}H_{27}AuPN$ [M-H]-: 700.1474; found: 700.1482

$B(pin)_2$NHC-AuX complexes

7) [N,N-Bis(2,6-diisopropylphenyl(4-pinacolboryl)imidazol-2-yl](chloro)gold(I)

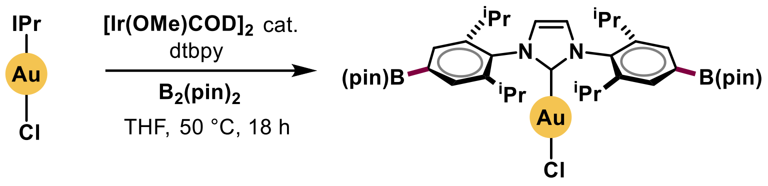

7´

Complex **7** was prepared according to a modified literature procedure.[21]

To a dried Youngs-tube under argon was added B_2Pin_2 (5 mg, 0.02 mmol, 0.2 equiv.), $[Ir(OMe)COD]_2$ (2 mg, 2.5 mol%), dtbpy (4,4′-Di-tert-butyl-2,2′-dipyridyl, 2 mg, 5.0 mol%) and dry THF (0.1 mL). The mixture was stirred in RT for 30 min. B_2Pin_2 (52 mg, 0.20 mmol, 2.0 equiv.) and IPr-Au-Cl (62 mg, 0.10 mmol, 1.0 equiv.) was added in one portion along with dry THF (0.3 mL). The mixture was stirred at 50°C oil bath for 14 h. The resulting mixture was diluted with DCM (15 mL), filtered through Celite and solution was reduced under vacuum. The solid was re-dissolved in DCM (0.1 mL) and triturated with pentane to afford a beige solid. The solid was washed with additional pentane and dried over vacuum to yield the target complex **7** as a beige solid (83 mg, 95%).

^{1}H NMR (400 MHz, Chloroform-*d*) δ 7.72 (s, 4H), 7.14 (s, 2H), 2.55 (hept, J = 6.91 Hz, 4H), 1.39 (s, 24H), 1.36 (d, J = 6.92 Hz, 12H), 1.24 (d, J = 6.91 Hz, 12H). ^{13}C NMR (101 MHz, Chloroform-*d*) δ 175.1, 144.6, 136.4, 130.7, 122.9, 84.1, 28.8, 24.9, 24.4, 24.0. HRMS-Nanospray calcd for $C_{39}H_{58}AuN_2O_4B_2Cl$ [M-H]-: 871.3878; found 871.3871.

Procedure E - preparation of $B(pin)_2$NHC-AuX complexes

7 (0.05 mmol, 1 equiv.) and potassium triolboronate (0.15 mmol, 3.0 equiv.) were added to a microwave vial fitted with a stirring bar. EtOH (1 mL, 99.5%) was added and the mixture was stirred at room temperature for 24 h. After stirring, the solid was washed with EtOH (2 mL) and left to settle. Remaining solution was decanted and solid was dried under vacuum. The target complex was afforded as a colourless solid without further purification.

$B(pin)_2NHC$-AuX complexes: characterization data

7a) [N,N-Bis(2,6-diisopropylphenyl(4-pinacolboryl)imidazol-2-yl](phenyl)gold(I)

Synthesis of **7a** was performed according to procedure E.

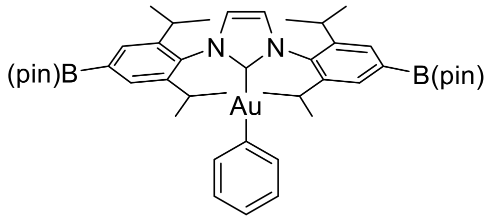

Isolated yield 91% (42 mg, 0.05 mmol scale, colourless solid). ^{1}H NMR (500 MHz, Chloroform-*d*) δ 7.62 (s, 4H), 7.03 (s, 2H), 7.00 (dd, *J* = 7.9, 1.6 Hz, 2H), 6.91 (dd, *J* = 7.9, 7.3 Hz, 2H), 6.73 (dd, *J* = 7.3, 1.6 Hz, 1H), 2.59 (hept, *J* = 6.9 Hz, 4H), 1.35 (d, *J* = 6.8 Hz, 12H), 1.30 (s, 24H), 1.19 (d, *J* = 6.9 Hz, 12H). ^{13}C NMR (126 MHz, Chloroform-*d*) δ 196.6, 169.6, 144.7, 140.5, 137.1, 130.4, 126.6, 124.1, 122.6, 84.0, 28.8, 24.9, 24.4, 23.9. HRMS-Nanospray calcd for $C_{45}H_{63}AuB_2KN_2O_4$ [M+K]+: 953.4277; found: 953.4281

7c) [N,N-Bis(2,6-diisopropylphenyl(4-pinacolboryl)imidazol-2-yl](3-thiophenyl)gold(I)

Synthesis of **7c** was performed according to procedure E.

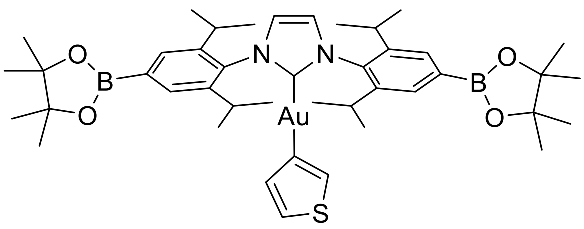

Isolated yield 76% (35 mg, 0.05 mmol scale, colourless solid). ^{1}H NMR (400 MHz, Chloroform-*d*) δ 7.70 (s, 4H), 7.23 (dd, *J* = 4.6, 2.5 Hz, 1H), 7.11 (s, 2H), 6.87 (dd, *J* = 4.6, 0.8 Hz, 1H), 6.72 (dd, *J* = 2.5, 0.8 Hz, 1H), 2.66 (hept, *J* = 7.3 Hz, 4H), 1.42 (d, *J* = 6.9 Hz, 12H), 1.38 (s, 22H), 1.26 (d, *J* = 6.9 Hz, 12H). ^{13}C NMR (101 MHz, Chloroform-*d*) δ 195.7, 164.4, 144.7, 137.0, 136.6, 130.5, 128.5, 122.6, 121.4, 84.0, 28.8, 24.9, 24.4, 23.9. HRMS-Nanospray calcd for $C_{43}H_{61}AuB_2KN_2O_4S$ [M+K]+: 959.3841; found: 959.3847

7c) [N,N-Bis(2,6-diisopropylphenyl(4-pinacolboryl)imidazol-2-yl](1-pyrenyl)gold(I)

Synthesis of **7c** was performed according to procedure E.

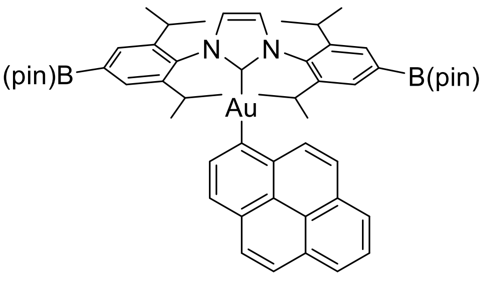

Isolated yield 90% (47 mg, 0.05 mmol scale, colourless solid). ^{1}H NMR (400 MHz, Chloroform-*d*) δ 7.94 – 7.84 (m, 4H), 7.83 (s, 4H), 7.81 – 7.75 (m, 3H), 7.68 (d, *J* = 9.0 Hz, 1H), 7.58 (d, *J* = 9.0 Hz, 1H), 2.78 (hept, *J* = 6.9 Hz, 4H), 1.46 (d, *J* = 6.9 Hz, 12H), 1.42 (s, 24H), 1.30 (d, *J* = 6.9 Hz, 12H). ^{13}C NMR (101 MHz, Chloroform-*d*) δ 197.2, 172.6, 145.1, 140.6, 138.1, 137.2, 135.2, 131.7, 131.6, 130.6, 128.1, 127.9, 125.5, 124.8, 124.8, 124.5, 123.4, 123.3, 122.8, 122.5, 122.3, 84.1, 28.9, 25.0, 24.4, 24.1. HRMS-Nanospray calcd for $C_{55}H_{67}AuB_2KN_2O_4$ [M+K]+: 1077.4590; found: 1077.4631

Mechanistic investigation

The mechanism is proposed to proceed through one of two suggested pathways (Scheme S1). Initially, the organoboronate is proposed to exchange with chloride and afford the Au(I) alkoxide complex **I**. We postulated that intermediate **I** could be detected by NMR spectroscopy.
The mechanism could subsequently proceed via two different pathways, A and B. Pathway A proceeds via intramolecular transmetalation which eliminates the neutral boronate **IIa**. Pathway B involves pre-activation of **I** via the coordination of a ROH species. Subsequent intramolecular transmetalation affords the expected product and eliminates the monocyclic boronate **IIb**.

Scheme S1: Proposed transmetalation mechanism.

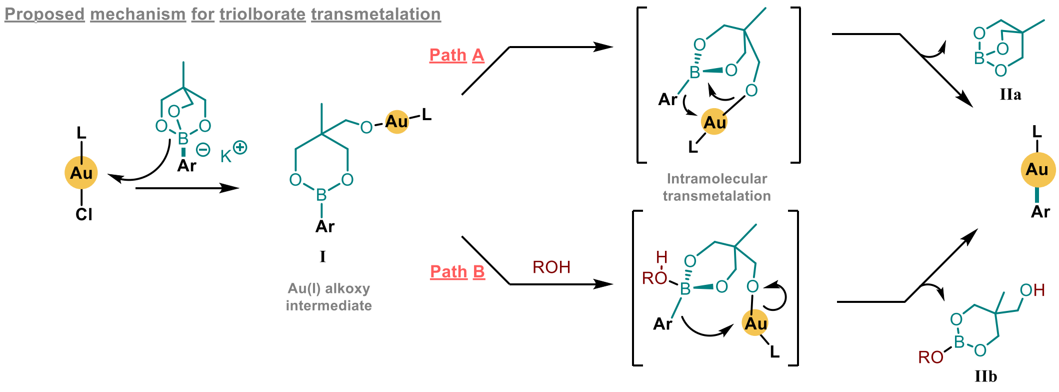

NMR Experiments

We performed ^{31}P and ^{11}B NMR experiments in an attempt to gain insight into the reaction mechanism. We postulated that observation of the Au(I) alkoxide intermediate **I** could be a feasible starting point.

We monitored the reaction between $Au(PPh_3)NTf_2$ and 3-tolyl B(triol)K via ^{31}P and ^{11}B NMR spectroscopy (^{1}H NMR spectroscopy was impractical due to the insolubility of the triolboronate salt, which led to signal broadening).

Method:

$Ph_3PAuNTf_2$ (37mg, 0.05 mmol) was loaded into an oven dried NMR tube under argon atmosphere. Chloroform-*d* (rigorously dried over K_2CO_3 and 4Å molecular sieves) was added under argon and the ^{31}P NMR spectrum of $Ph_3PAuNTf_2$ was recorded (Scheme S2, spectrum A). 3-Tolyl B(triol)K (0.05 mmol) was added, the reaction mixture was sonicated for 5 minutes and ^{31}P NMR spectrum was recorded (Scheme S2, spectrum B). The ^{11}B NMR spectrum was also recorded (spectrum E).

At this stage, in the case of the ^{31}P NMR experiment, EtOH (99.5%, 0.1 mL) was added, the sample was sonicated 5 min and the ^{31}P NMR spectrum was recorded (spectrum C).

Alternatively, in the case of the ^{11}B NMR experiment, EtOH (0.5 equiv.) was added and the ^{11}B NMR spectrum was recorded (Scheme S2, spectrum F). Thereafter, a second addition of EtOH (0.5 equiv.) was made, and the ^{11}B NMR spectrum recorded (spectrum G).

Results:

In the ^{31}P NMR study, upon triolboronate addition to $Ph_3PAuNTf_2$, a new signal appears downfield with respect to the starting cationic complex (δ = 38 ppm, Scheme S2, spectrum B). This signal is proposed to belong to the alkoxide Au(I) intermediate **I**. This species remains stable up to at least 4 h in solution, which counts against proposed pathway A, as intramolecular transmetalation would presumably occur quickly. The fact that EtOH addition leads to immediate transmetalation (compare spectra C and D) further suggests that Pathway B is likely.

In the ^{11}B NMR study, upon triolboronate addition to $Ph_3PAuNTf_2$ in $CDCl_3$, a signal is observed in the ^{11}B NMR spectrum (δ = 27 ppm, spectrum E) which is proposed to correspond to intermediate **I**. The observed signal does not correspond to the triolboronate, as the latter is insoluble in $CDCl_3$, but instead coincides with the expected shift of a Ar-$B(OR)_2$ species.

Upon addition of 0.5 equivalents of EtOH, a signal representing a new species appears upfield in the ^{11}B NMR spectrum (δ = 18 ppm, spectrum F). This new species forms in an equimolar ratio (1:1) to intermediate **I**, and is proposed to be the $B(OR)_3$ species formed a by-product of the transmetalation. Upon the second addition of EtOH (0.5 equiv.), full conversion of intermediate **I** to $B(OR)_3$ is observed. These observations are consistent with the process observed to occur via 31P NMR spectroscopy (see above) and further support the evidence for Pathway B.

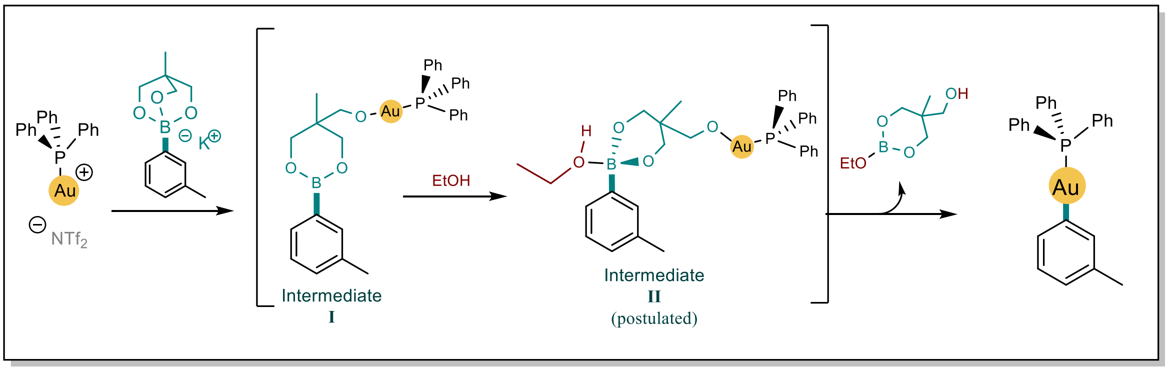

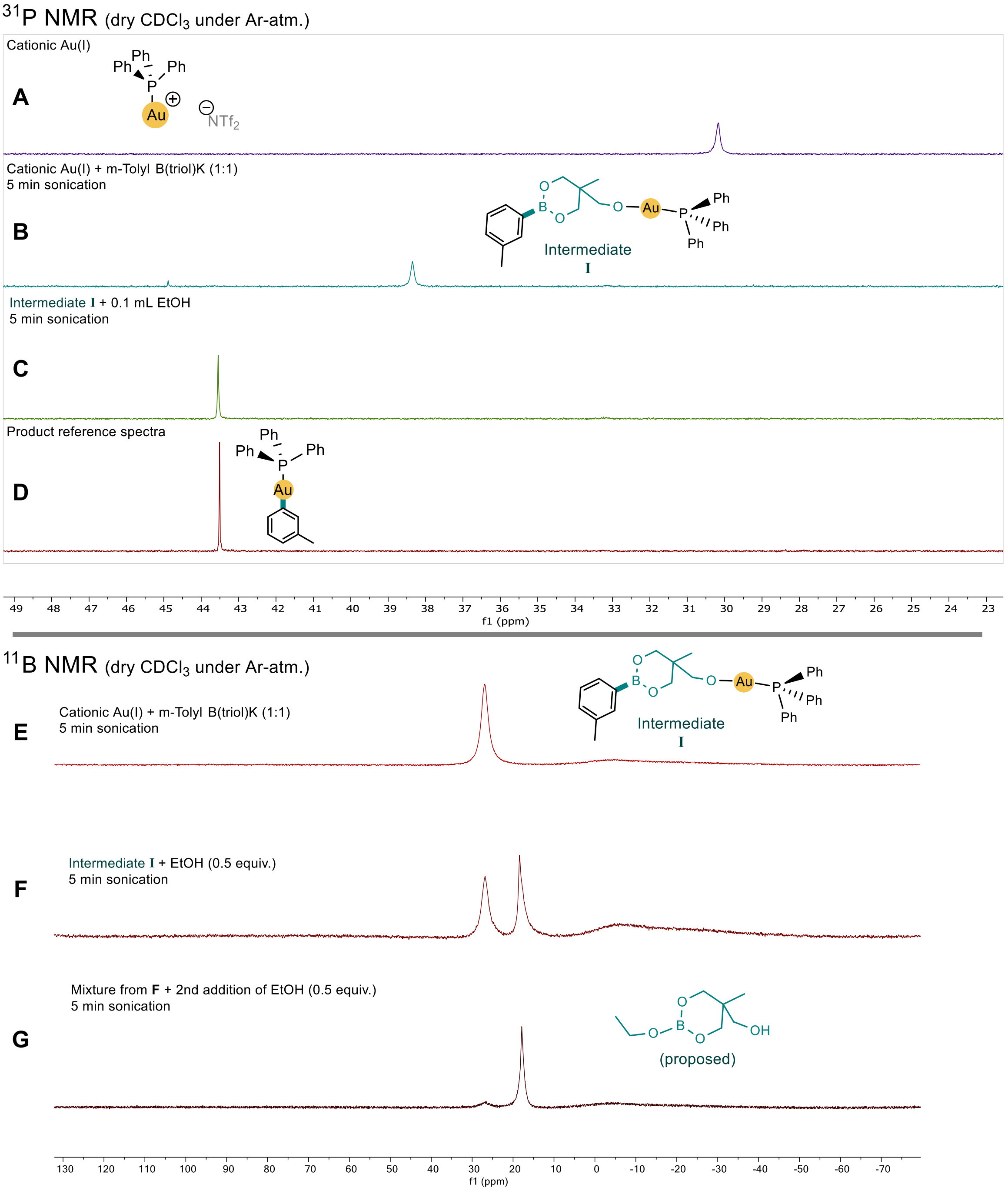

Scheme S2. Proposed mechanism for transmetalation from potassium triolboronates and ^{31}P (top) and ^{11}B NMR (bottom) spectroscopic investigation of intermediates.

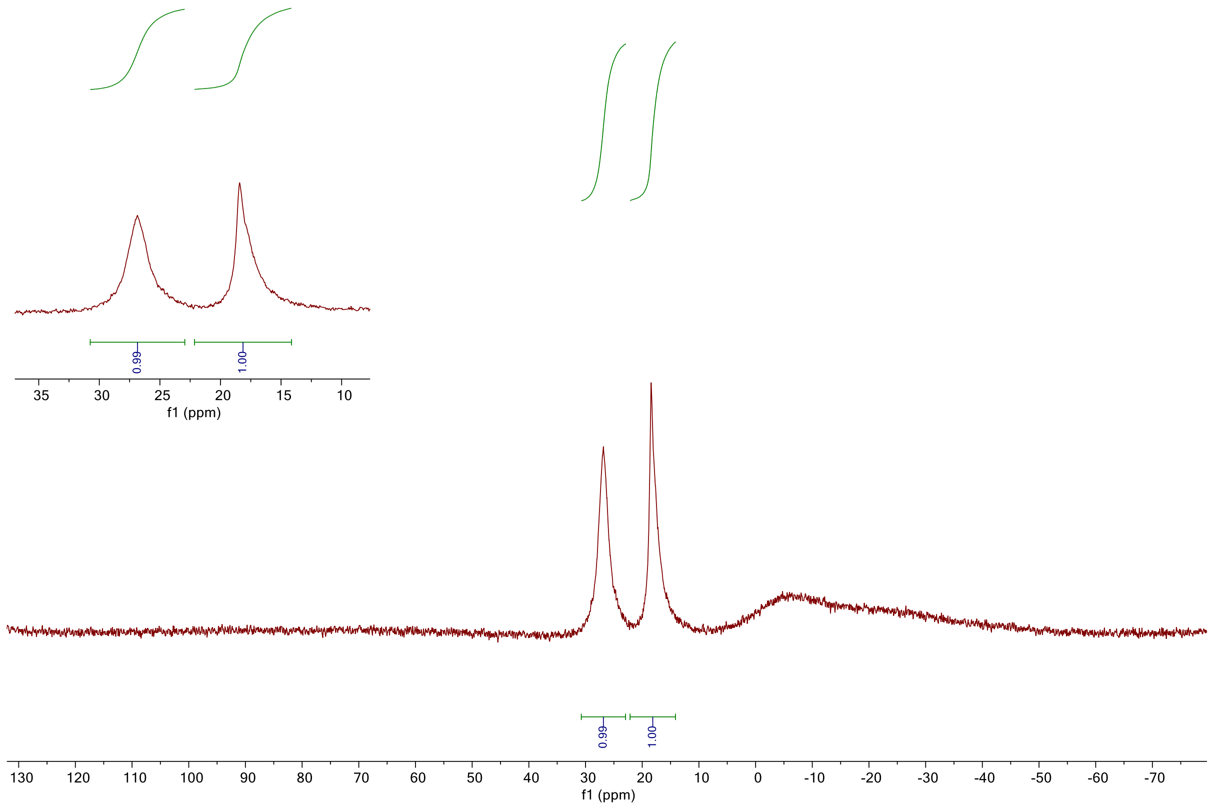

Scheme S3. ^{11}B NMR spectrum of $Ph_3PAuNTf_2$ and m-tolyl B(triol)K (1:1) in $CDCl_3$ after 0.5 equiv. EtOH addition (enhancement of bottom spectrum from Scheme S2).

Competition experiment

A competition study was performed in which the rate of transmetalation from the triol-based organoboronate to the Au(I) center was directly compared to that of other boronates commonly used in similar transmetalations (Scheme S4). 3-Iodophenyl B(triol)K (**2e**) was compared against two different 3-tolyl boronates (**2b**,**c**) in the transmetalation to PPh_3AuCl (**3**) in EtOH (Scheme S3).

Method:

Equimolar amounts of PPh_3AuCl (**3**), **2e** and either one of **2b** or **2c** (0.1 mmol) were added to a 5 mL microwave vial. EtOH (99.5%, 1.0 mL) was added and the reaction was stirred at 23 °C for 24 h. The reaction was extracted with DCM (2 x 20 mL) and washed with water (20 mL). Organic fractions were dried over Na_2SO_4 and concentrated under reduced pressure. The resulting ratio of **4e**:**4a** was determined using ^{31}P NMR spectroscopy in chloroform-*d*.

Results:

In both competition experiments, complete consumption of **3** was observed and **4e** was the major product. In competition experiments using boronic acid **2b**, the product of the transmetalation from **2e** was the only observed product (**4e**). The product **4a**, which would have resulted from transmetalation from the boronic acid starting material **2b**, was not observed.

Competition between **2e** and **2c** also resulted in the full conversion of **3**, and afforded triolboronate transmetalation product **4e** as the major product in 79% spectroscopic yield. Partial conversion of **2c** was observed: the product **4a**, resulting from transmetalation from the pinacol boronate **2c**, formed in 21% spectroscopic yield.

Transmetalation from aryl triolboronates under ambient conditions thus occurs more readily than from the corresponding pinacol-protected boronate or boronic acid. We have also shown that transmetalation from aryl triolboronates proceeds with exceptional chemoselectivity, even in the presence of aryl boronic acids.

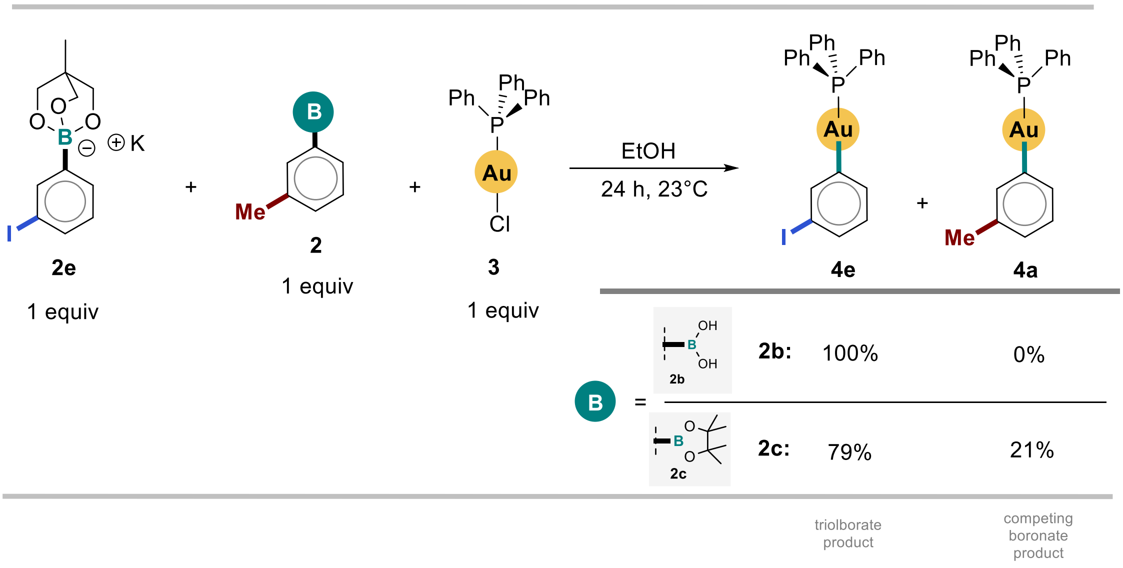

Scheme S4. Competition experiment.

Crystallographic data

4n

Table 1 Crystal data and structure refinement for 4n.

CCDC No.	1960515
Empirical formula	$C_{22}H_{18}AuPS$
Formula weight	542.36
Temperature/K	123.15(2)
Crystal system	triclinic
Space group	P-1
a/Å	7.918(9)
b/Å	11.158(12)
c/Å	11.794(13)
α/°	93.22(4)
β/°	105.43(4)
γ/°	95.71(4)
Volume/Å^3	995.7(19)
Z	2
ρ_{calc}g/cm^3	1.809
μ/mm^{-1}	7.573
F(000)	520.0
Crystal size/mm^3	0.12 × 0.12 × 0.1
Radiation	MoKα (λ = 0.71073)
2Θ range for data collection/°	4.92 to 57.27
Index ranges	$-10 \le h \le 10$, $-14 \le k \le 14$, $-15 \le l \le 15$
Reflections collected	10412
Independent reflections	4955 [R_{int} = 0.0197, R_{sigma} = 0.0289]
Data/restraints/parameters	4955/148/260
Goodness-of-fit on F^2	1.044
Final R indexes [I>=2σ (I)]	R_1 = 0.0185, wR_2 = 0.0412
Final R indexes [all data]	R_1 = 0.0206, wR_2 = 0.0419
Largest diff. peak/hole / e Å^{-3}	1.05/-0.59

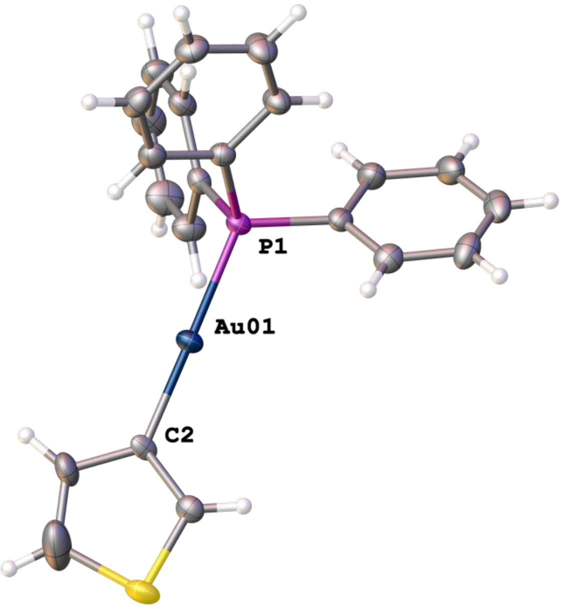

Crystal Data for $C_{22}H_{18}AuPS$ (**4n**) (*M* =542.36 g/mol): triclinic, space group P-1 (no. 2), *a* = 7.918(9) Å, *b* = 11.158(12) Å, *c* = 11.794(13) Å, *α* = 93.22(4)°, *β* = 105.43(4)°, *γ* = 95.71(4)°, *V* = 995.7(19) Å^3, *Z* = 2, *T* = 123.15(2) K, μ(MoKα) = 7.573 mm^{-1}, *Dcalc* = 1.809 g/cm^3, 10412 reflections measured (4.92° ≤ 2Θ ≤ 57.27°), 4955 unique (R_{int} = 0.0197, R_{sigma} = 0.0289) which were used in all calculations. The final R_1 was 0.0185 (I > 2σ(I)) and wR_2 was 0.0419 (all data).

5j

Table 2 Crystal data and structure refinement for 5j.

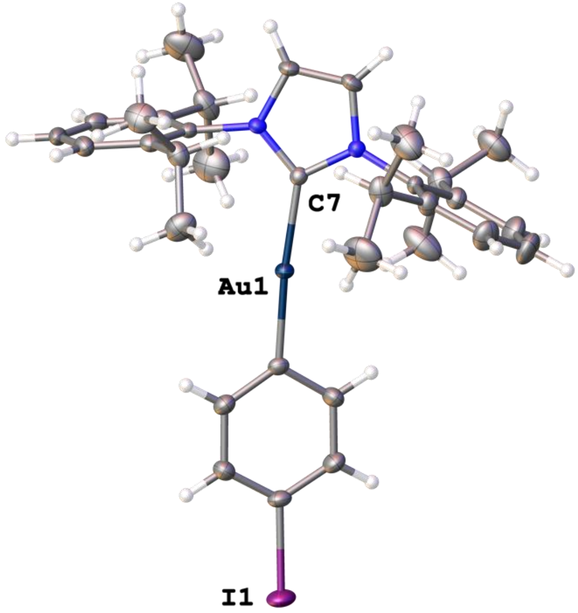

CCDC No.	1878065
Empirical formula	$C_{33}H_{40}AuIN_2$
Formula weight	788.53
Temperature/K	173.15(2)
Crystal system	monoclinic
Space group	$P2_1/c$
a/Å	15.7517(3)
b/Å	13.6363(2)
c/Å	15.1693(2)
α/°	90
β/°	104.7820(10)
γ/°	90
Volume/Å^3	3150.45(9)
Z	4
ρ_{calc}g/cm^3	1.662
μ/mm^{-1}	5.672
F(000)	1536.0
Crystal size/mm^3	0.2 × 0.2 × 0.11
Radiation	MoKα (λ = 0.71073)
2Θ range for data collection/°	4.01 to 61.218
Index ranges	$-22 \le h \le 22$, $-19 \le k \le 19$, $-21 \le l \le 21$
Reflections collected	66820
Independent reflections	9679 [R_{int} = 0.0494, R_{sigma} = 0.0330]
Data/restraints/parameters	9679/0/342
Goodness-of-fit on F^2	1.022
Final R indexes [I>=2σ (I)]	R_1 = 0.0230, wR_2 = 0.0437
Final R indexes [all data]	R_1 = 0.0323, wR_2 = 0.0462
Largest diff. peak/hole / e Å^{-3}	0.58/-0.88

Crystal Data for $C_{33}H_{40}AuIN_2$ (**5j**) (*M* =788.53 g/mol): monoclinic, space group $P2_1/c$ (no. 14), *a* = 15.7517(3) Å, *b* = 13.6363(2) Å, *c* = 15.1693(2) Å, *β* = 104.7820(10)°, *V* = 3150.45(9) Å^3, *Z* = 4, *T* = 173.15(2) K, μ(MoKα) = 5.672 mm^{-1}, *Dcalc* = 1.662 g/cm^3, 66820 reflections measured (4.01° ≤ 2Θ ≤ 61.218°), 9679 unique (R_{int} = 0.0494, R_{sigma} = 0.0330) which were used in all calculations. The final R_1 was 0.0230 (I > 2σ(I)) and wR_2 was 0.0462 (all data).

References

(1) SAINT, Bruker, Bruker AXS Inc., Madison, Wisconsin, U. *SAINT*; 2007.

(2) Sheldrick, G. M. *Acta Crystallogr. Sect. C Struct. Chem.* **2015**, *71* (1), 3–8.

(3) Sheldrick, G. M.; IUCr. *Acta Crystallogr. Sect. A Found. Crystallogr.* **2008**, *64* (1), 112–122.

(4) Dolomanov, O. V.; Bourhis, L. J.; Gildea, R. J.; Howard, J. A. K.; Puschmann, H. *J. Appl. Crystallogr.* **2009**, *42* (2), 339–341.

(5) Yamamoto, Y.; Takizawa, M.; Yu, X.-Q.; Miyaura, N. *Angew. Chemie Int. Ed.* **2008**, *47* (5), 928–931.

(6) Li, G.-Q.; Kiyomura, S.; Yamamoto, Y.; Miyaura, N. *Chem. Lett.* **2011**, *40* (7), 702–704.

(7) Maslak, V.; Tokic-Vujosevic, Z.; Saicic, R. N. *Tetrahedron Lett.* **2009**, *50* (16), 1858–1860.

(8) Miyaura, N.; Yamamoto, Y.; Takizawa, M.; Yu, X.-Q. *Heterocycles* **2010**, *80* (1), 359.

(9) Peña-López, M.; Sarandeses, L. A.; Pérez Sestelo, J. *European J. Org. Chem.* **2013**, *2013* (13), 2545–2554.

(10) Partyka, D. V.; Zeller, M.; Hunter, A. D.; Gray, T. G. *Angew. Chemie Int. Ed.* **2006**, *45* (48), 8188–8191.

(11) Barnes, N. A.; Flower, K. R.; Fyyaz, S. A.; Godfrey, S. M.; McGown, A. T.; Miles, P. J.; Pritchard, R. G.; Warren, J. E. *CrystEngComm* **2010**, *12* (3), 784–794.

(12) Croix, C.; Balland-Longeau, A.; Allouchi, H.; Giorgi, M.; Duchêne, A.; Thibonnet, J. *J. Organomet. Chem.* **2005**, *690* (21–22), 4835–4843.

(13) Flower, K. R.; McGown, A. T.; Miles, P. J.; Pritchard, R. G.; Warren, J. E. *Dalt. Trans.* **2010**, *39* (14), 3509.

(14) Meyer, N.; Schucht, H.; Lehmann, C. W.; Weibert, B.; Winter, R. F.; Mohr, F. *Eur. J. Inorg. Chem.* **2017**, *2017* (2), 521–526.

(15) Romanov, A. S.; Bochmann, M. *Organometallics* **2015**, *34* (11), 2439–2454.

(16) V. Partyka, D.; Zeller, M.; D. Hunter, A.; G. Gray, T.; Partyka, D. V.; Zeller, M.; Hunter, A. D.; Gray, T. G. *Inorg. Chem.* **2012**, *51* (15), 8394–8401.

(17) Croix, C.; Sauvage, C.-E.; Balland-Longeau, A.; Duchêne, A.; Thibonnet, J. *J. Inorg. Organomet. Polym. Mater.* **2008**, *18* (3), 334–343.

(18) Dupuy, S. S.; Crawford, L.; Bühl, M.; Slawin, A. M. Z.; Nolan, S. P. *Adv. Synth. Catal.* **2012**, *354* (13), 2380–2386.

(19) Pankajakshan, S.; Loh, T.-P. *Chem. - An Asian J.* **2011**, *6* (9), 2291–2295.

(20) Partyka, D.; Esswein, A.; Zeller, M.; Hunter, A.; Gray, T.; Partyka, D.; Esswein, A.; Zeller, M.; Hunter, A.; Gray, T. *Organometallics* **2007**, *26* (14), 3279–3282.

(21) Ishiyama, T.; Takagi, J.; Ishida, K.; Miyaura, N.; R. Anastasi, N.; F. Hartwig, J. *J. Am. Chem. Soc.* **2001**, *124* (3), 390–391.

Copies of NMR Spectra

Potassium triolboronates

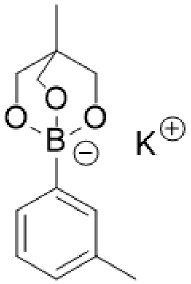

2a

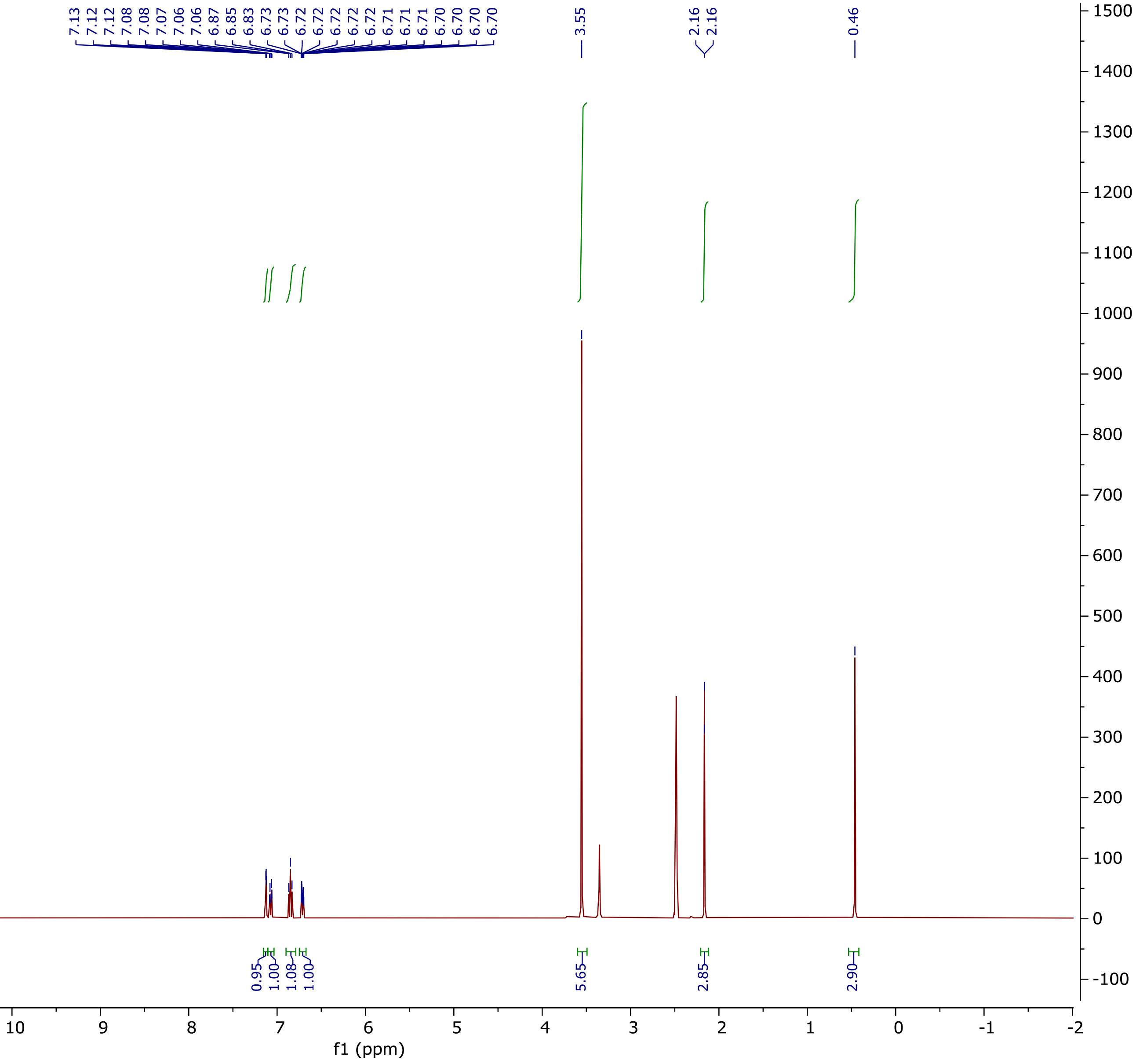

7.13
7.12
7.12
7.08
7.08
7.07
7.06
7.06
6.87
6.85
6.83
6.73
6.73
6.72
6.72
6.72
6.72
6.72
6.71
6.71
6.71
6.70
6.70
6.70
6.70
3.55
2.16
2.16
0.46
0.95
1.00
1.08
1.00
5.65
2.85
2.90
f1 (ppm)

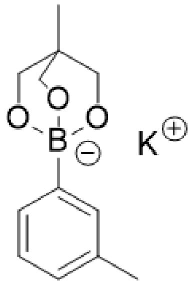

2a

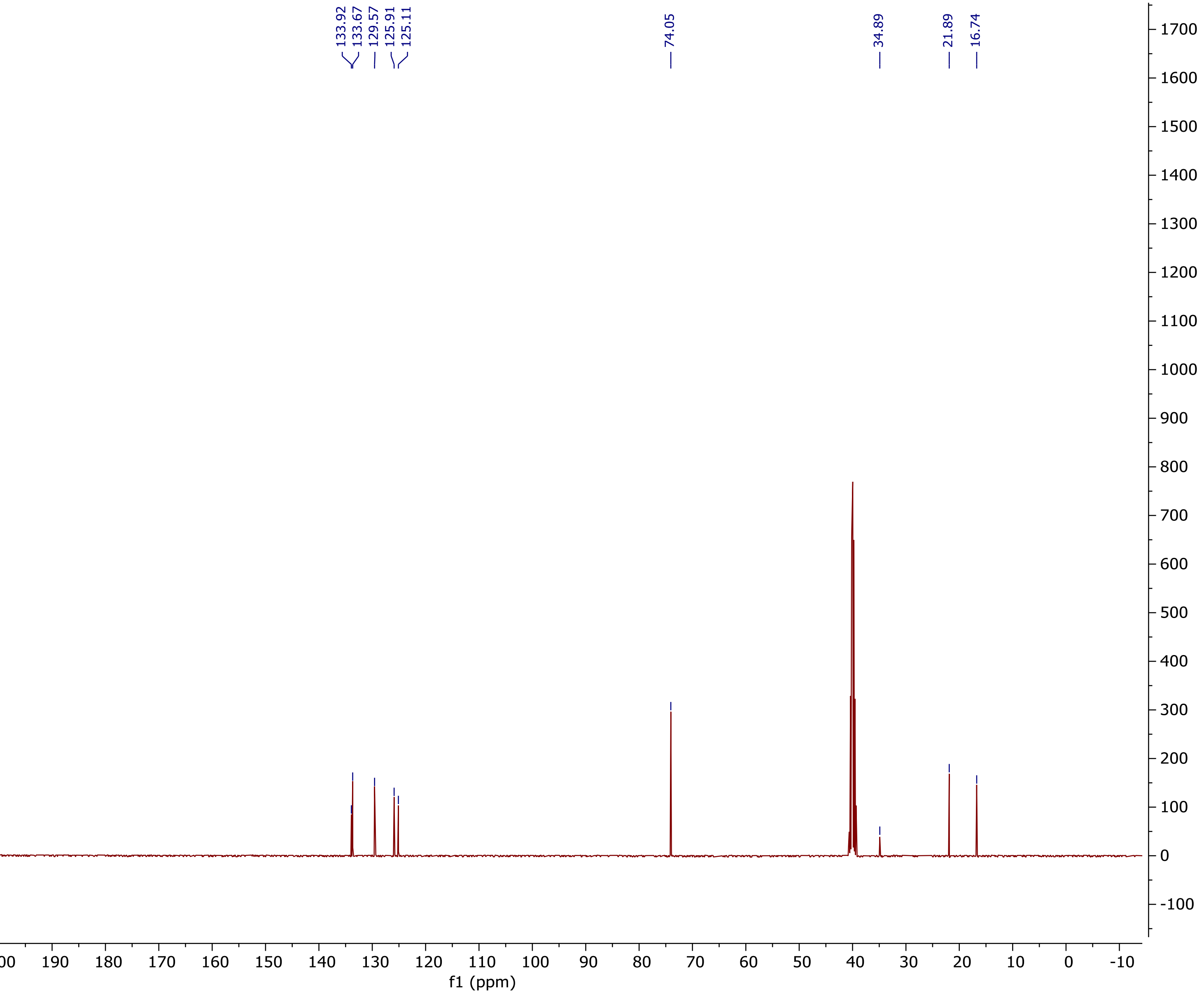

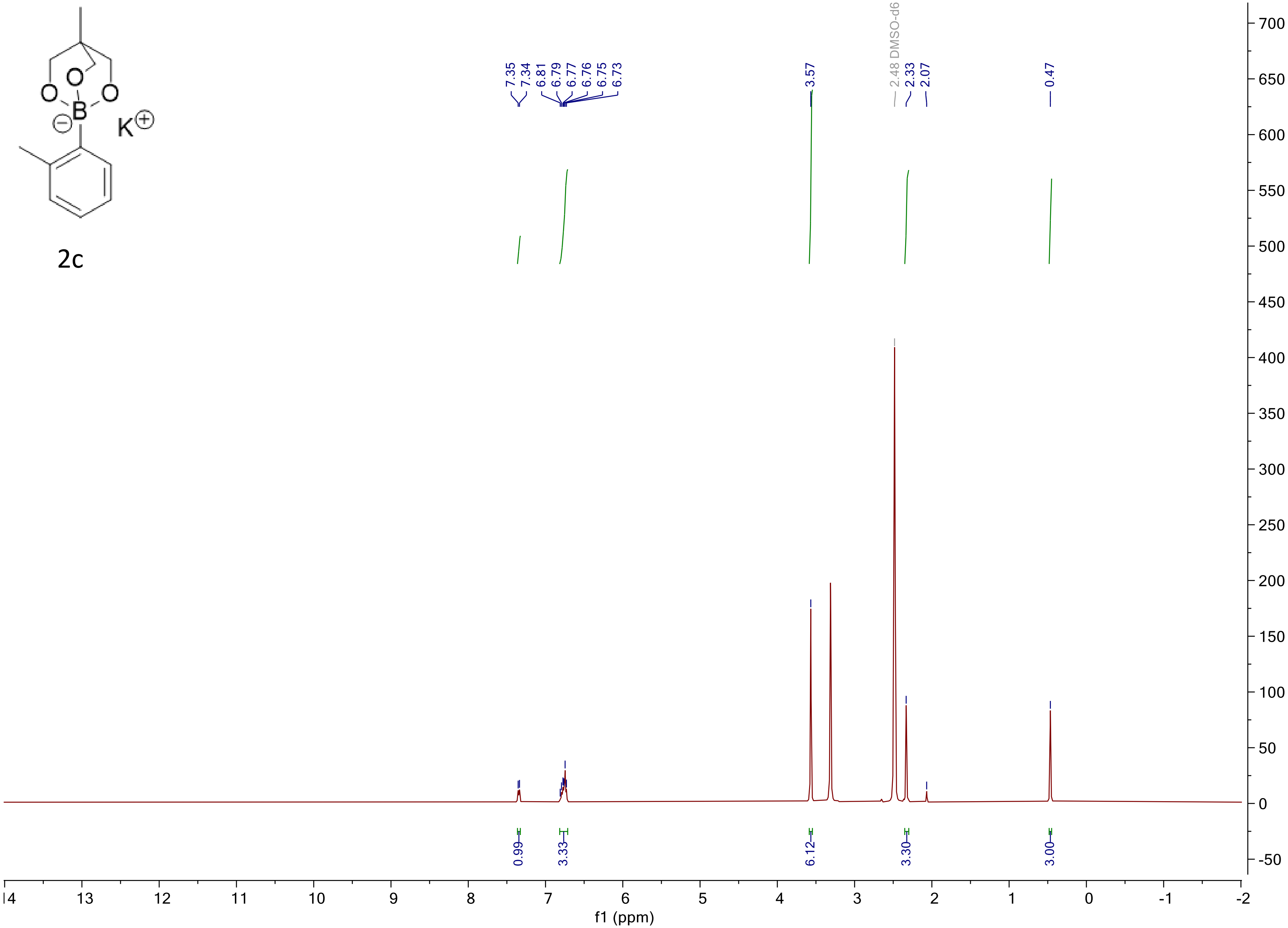

O
O
O
B
K
7.35
7.34
6.81
6.79
6.77
6.76
6.75
6.73
3.57
2.48 DMSO-d6
2.33
2.07
0.47
0.99
3.33
6.12
3.30
3.00
f1 (ppm)

2c

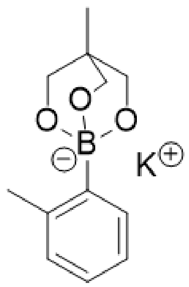

2c

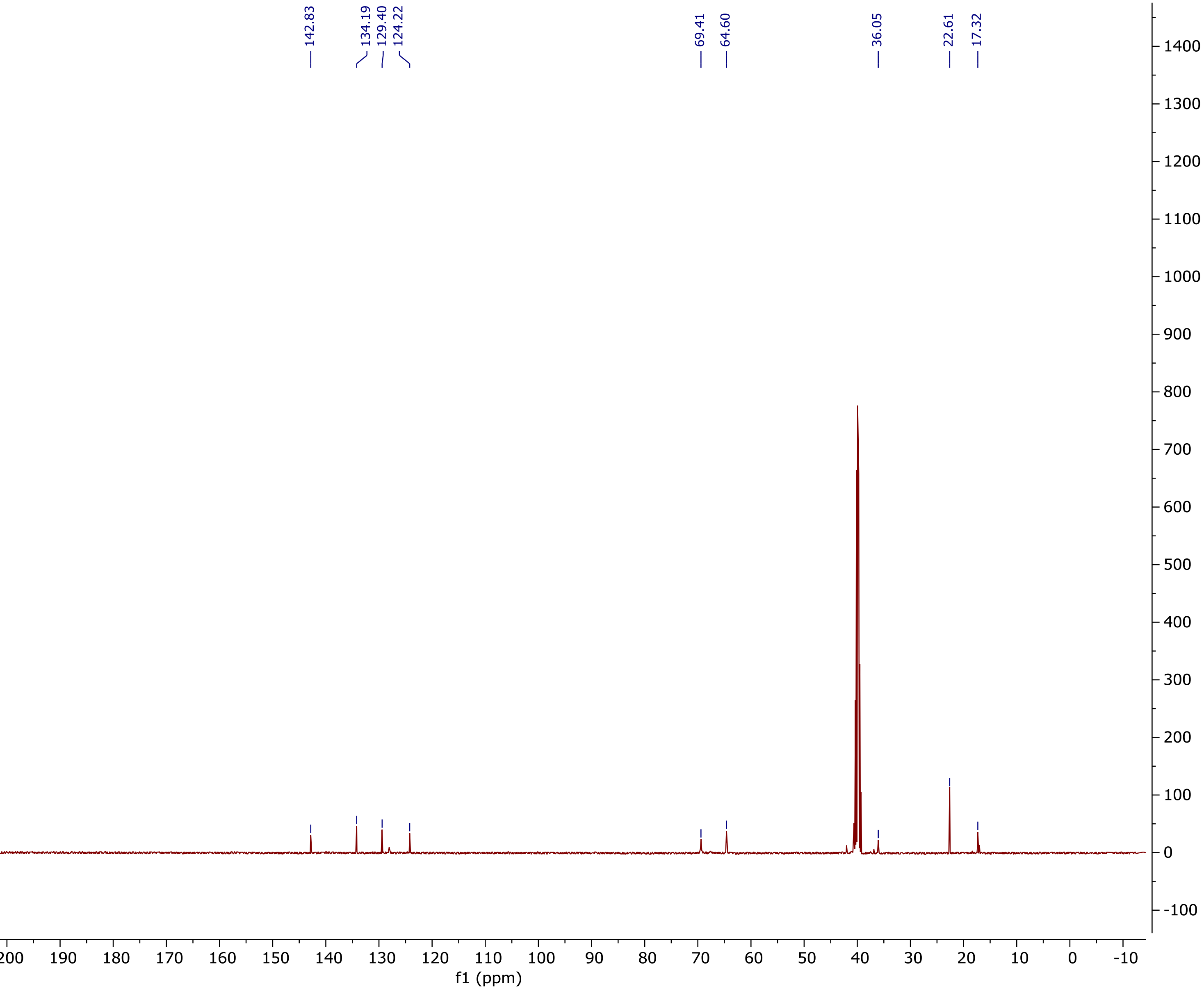
142.83
134.19
129.40
124.22
69.41
64.60
36.05
22.61
17.32
1400
1300
1200
1100
1000
900
800
700
600
500
400
300
200
100
0
-100
230 220 210 200 190 180 170 160 150 140 130 120 110 100 90 80 70 60 50 40 30 20 10 0 -10
f1 (ppm)

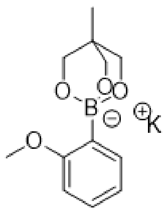

2d

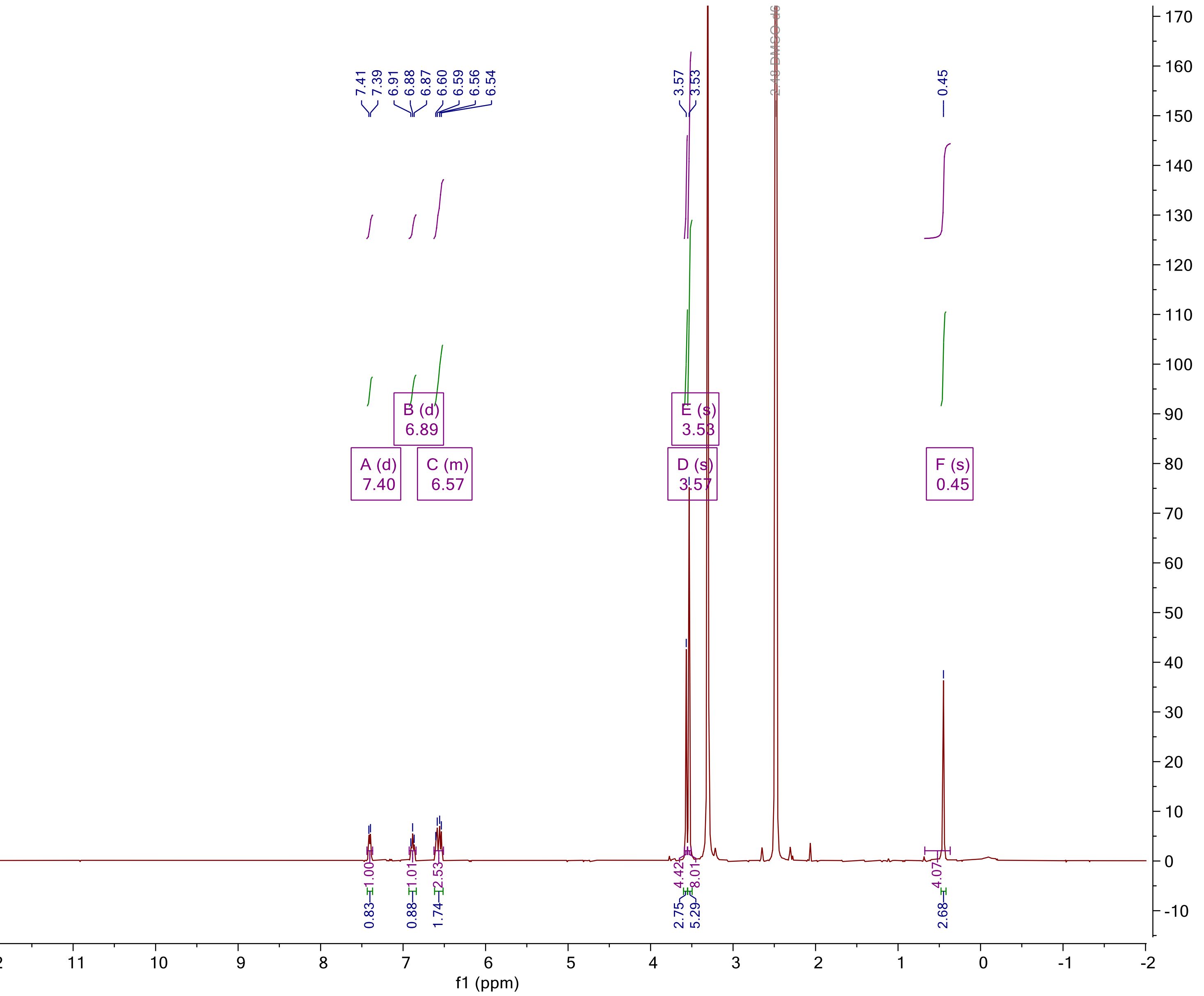

7.41
7.39
6.91
6.88
6.87
6.60
6.59
6.56
6.54
3.57
3.53
2.49 DMSO-d6
0.45
A (d)
7.40
B (d)
6.89
C (m)
6.57
D (s)
3.57
E (s)
3.53
F (s)
0.45
0.83
1.00
0.88
1.01
1.74
2.53
2.75
4.42
5.29
8.01
4.07
2.68
f1 (ppm)

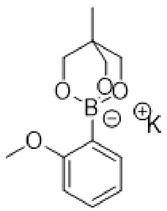
O
O
O
B
K
O

2d

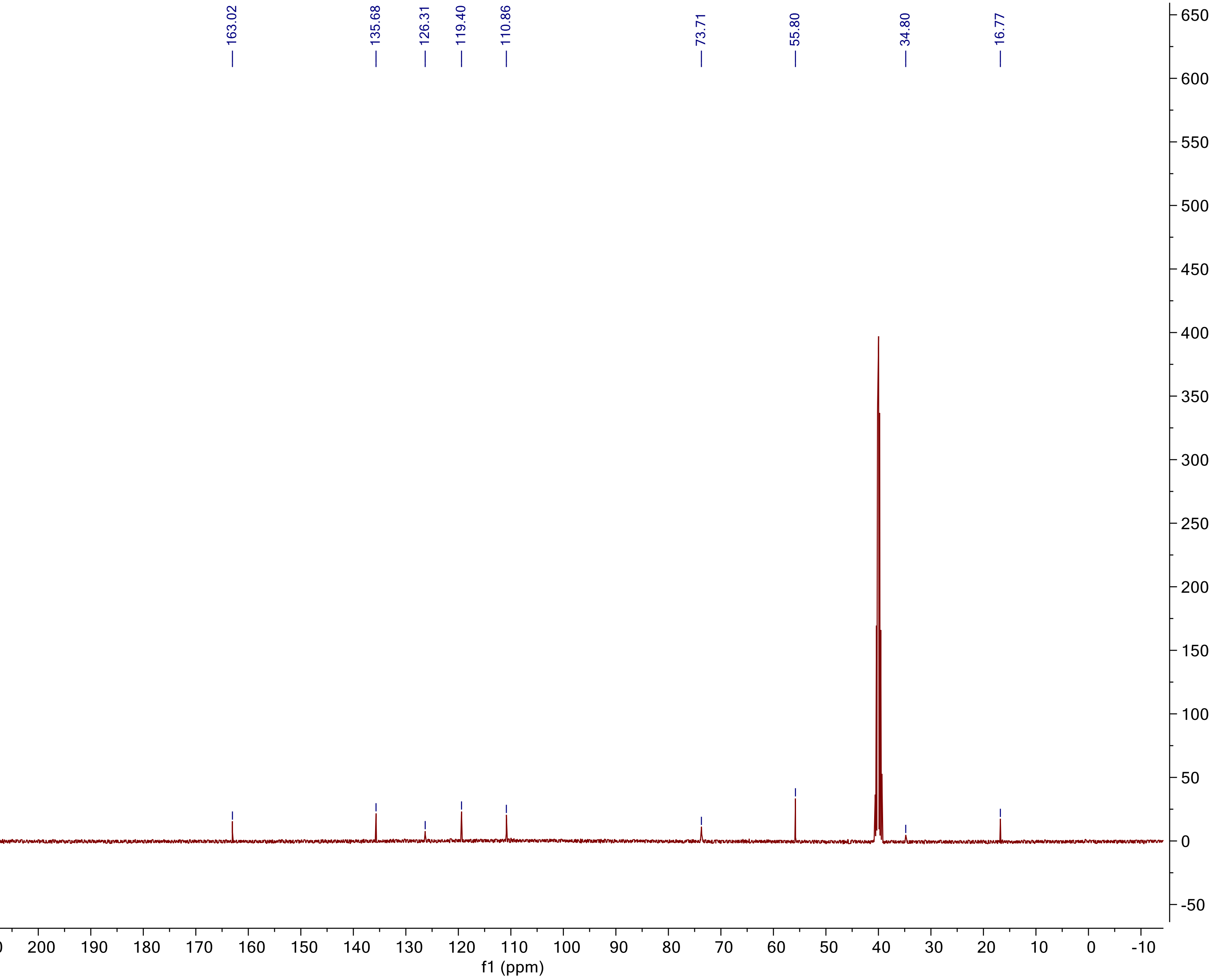
163.02
135.68
126.31
119.40
110.86
73.71
55.80
34.80
16.77
f1 (ppm)

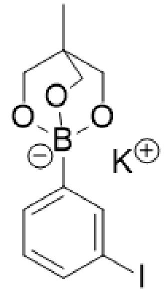
O
O
O
B
K
I

2e

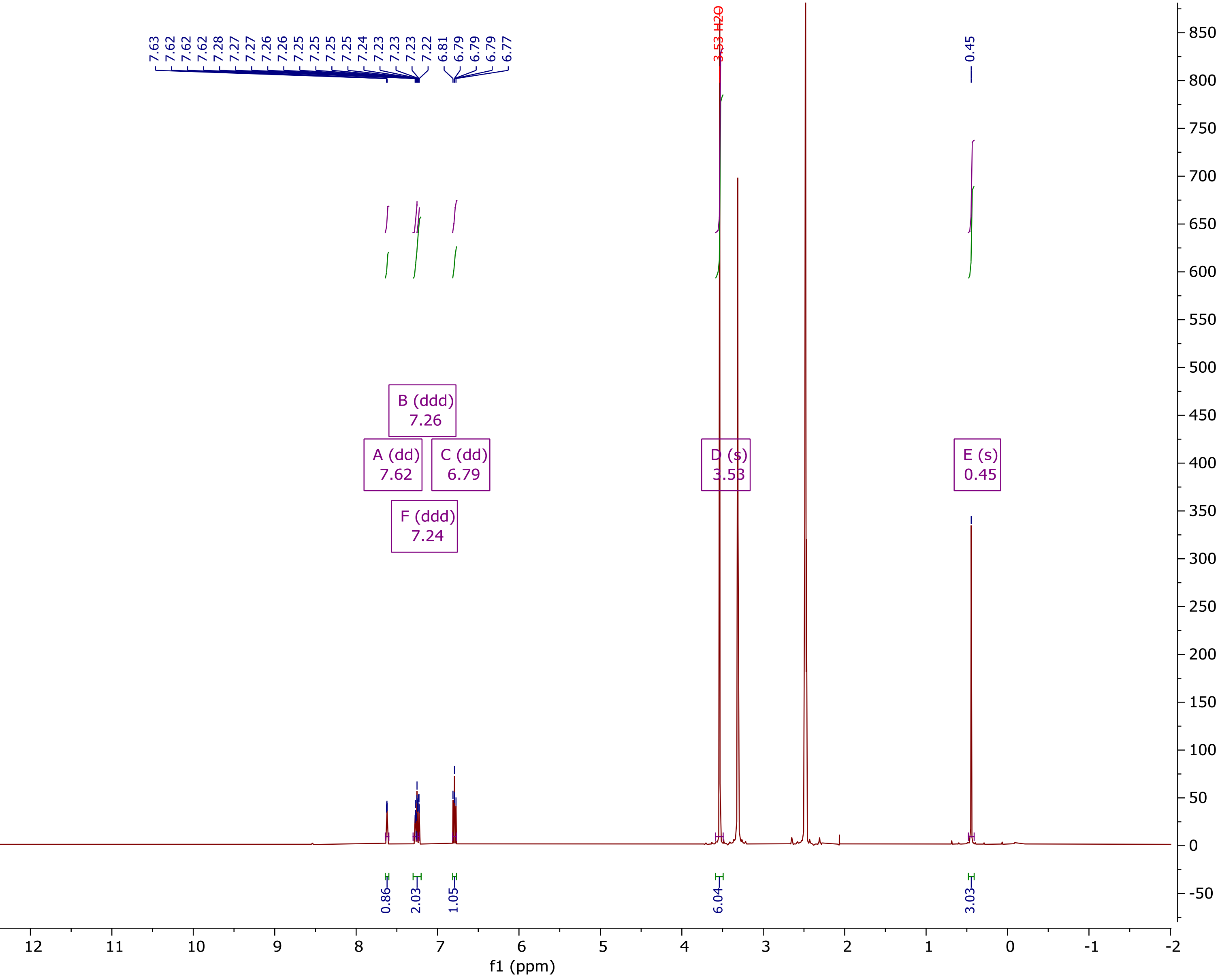
7.63
7.62
7.62
7.62
7.28
7.27
7.27
7.26
7.26
7.25
7.25
7.25
7.25
7.24
7.23
7.23
7.23
7.22
6.81
6.79
6.79
6.79
6.77
3.53 H2O
0.45
B (ddd)
7.26
A (dd)
7.62
C (dd)
6.79
F (ddd)
7.24
D (s)
3.53
E (s)
0.45
0.86
2.03
1.05
6.04
3.03
f1 (ppm)

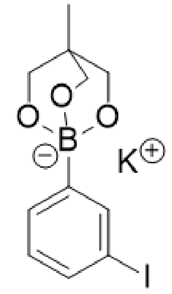

2e

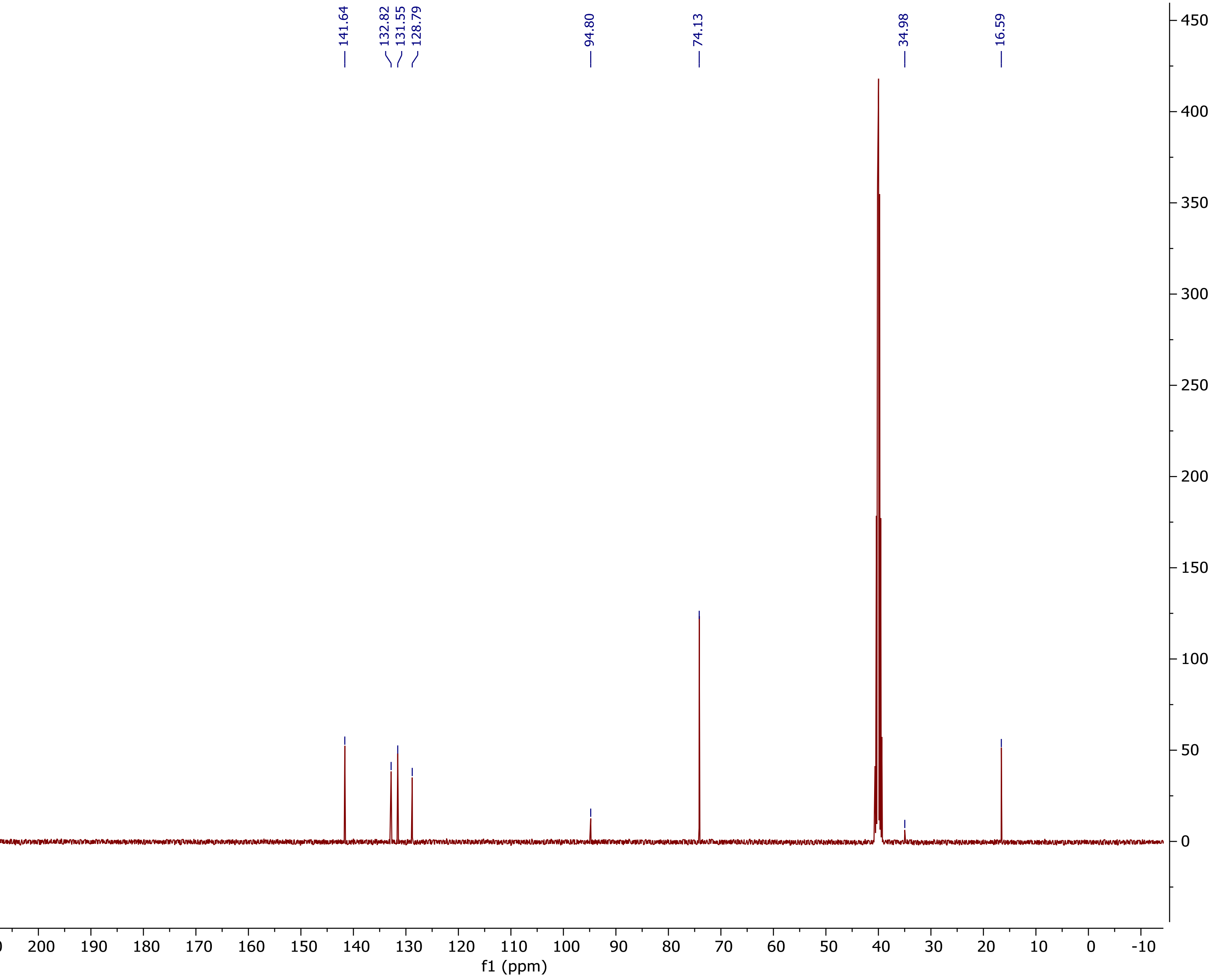

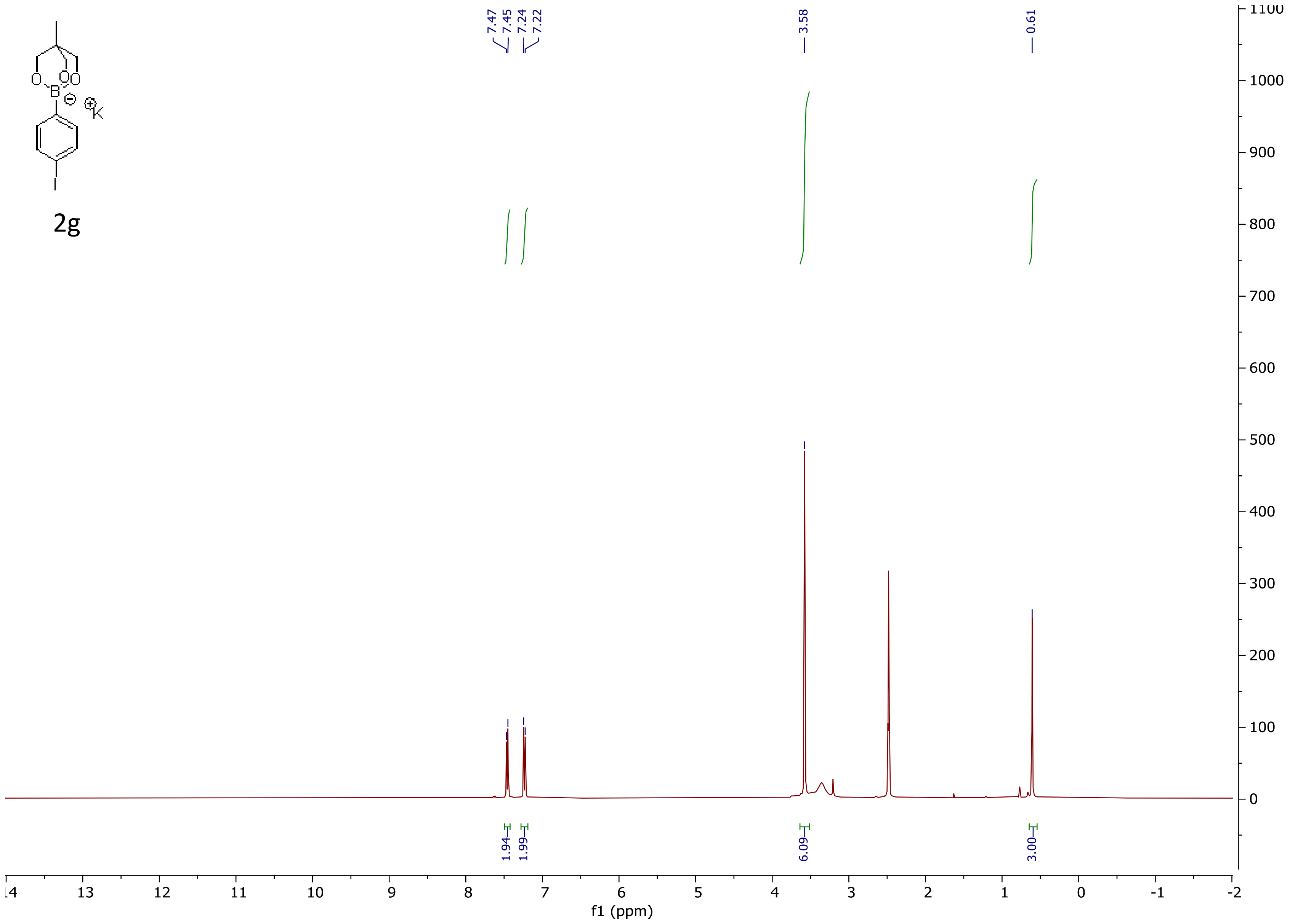
2g
7.47
7.45
7.24
7.22
3.58
0.61
1.94
1.99
6.09
3.00
f1 (ppm)

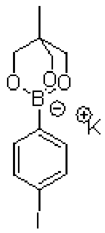

2g

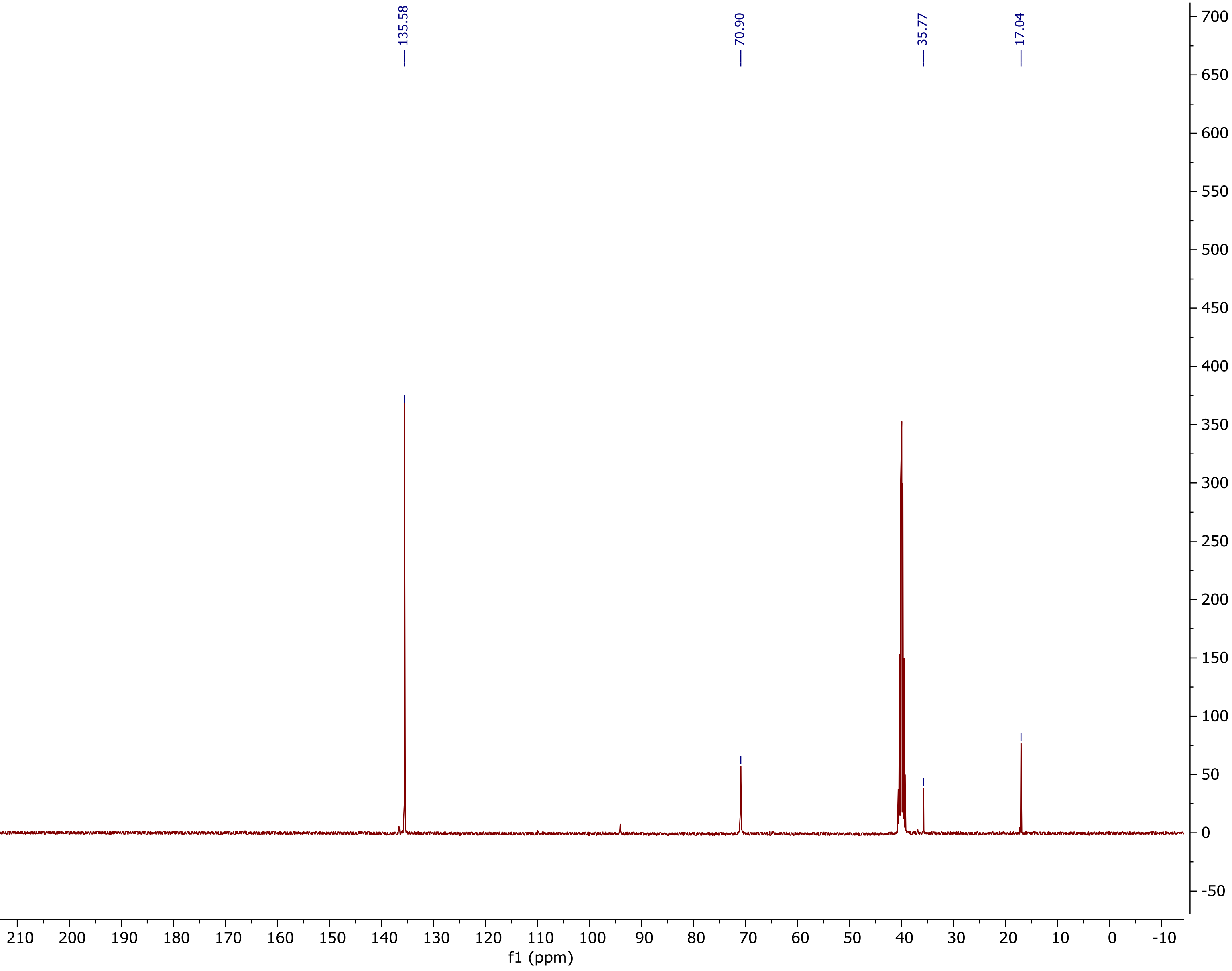

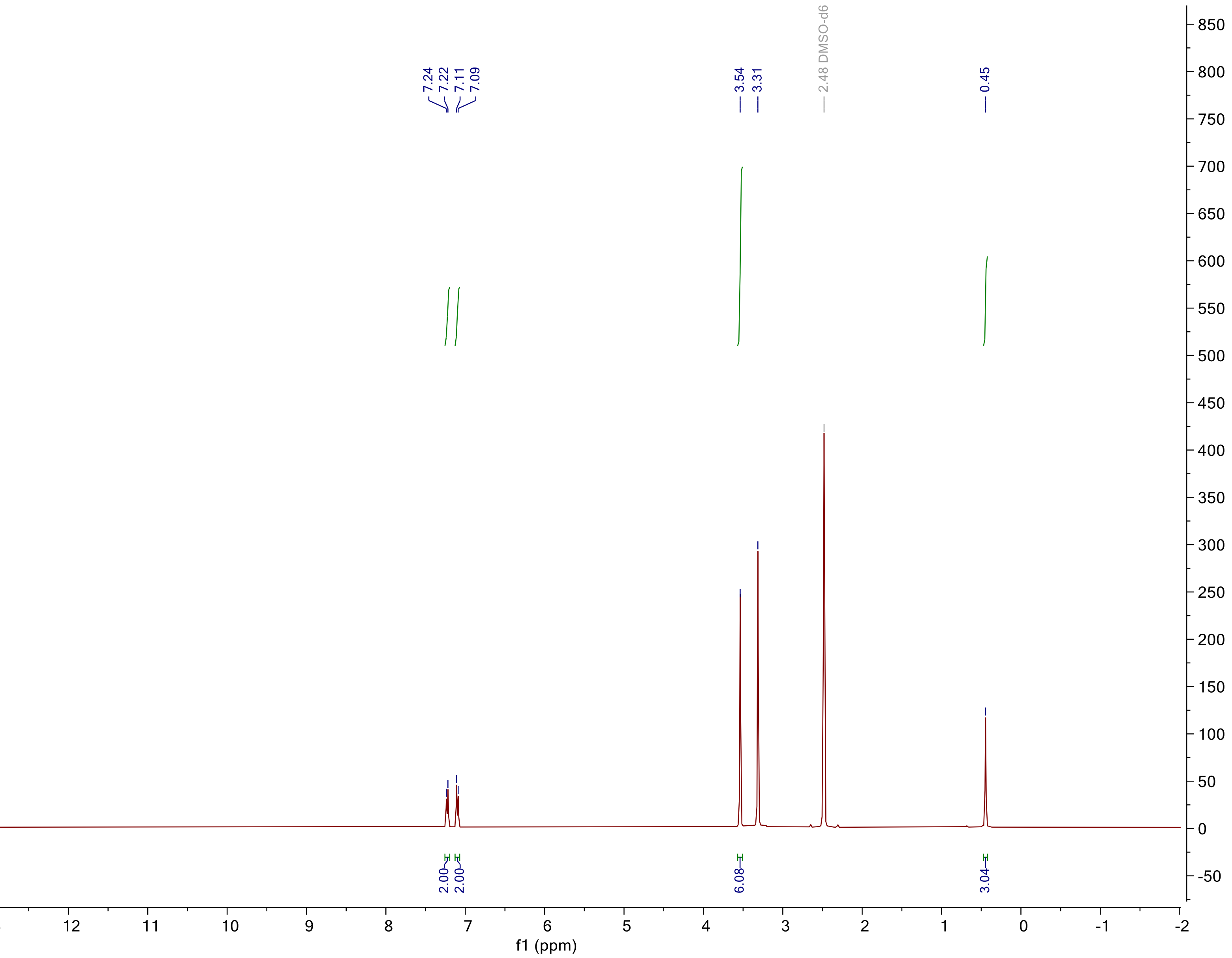
7.24
7.22
7.11
7.09
3.54
3.31
2.48 DMSO-d6
0.45
2.00
2.00
6.08
3.04
f1 (ppm)

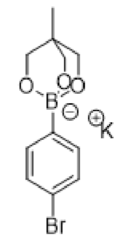
Br
K

2h

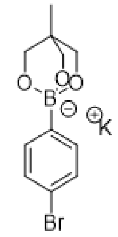
O
B
O
O
K
Br

2h

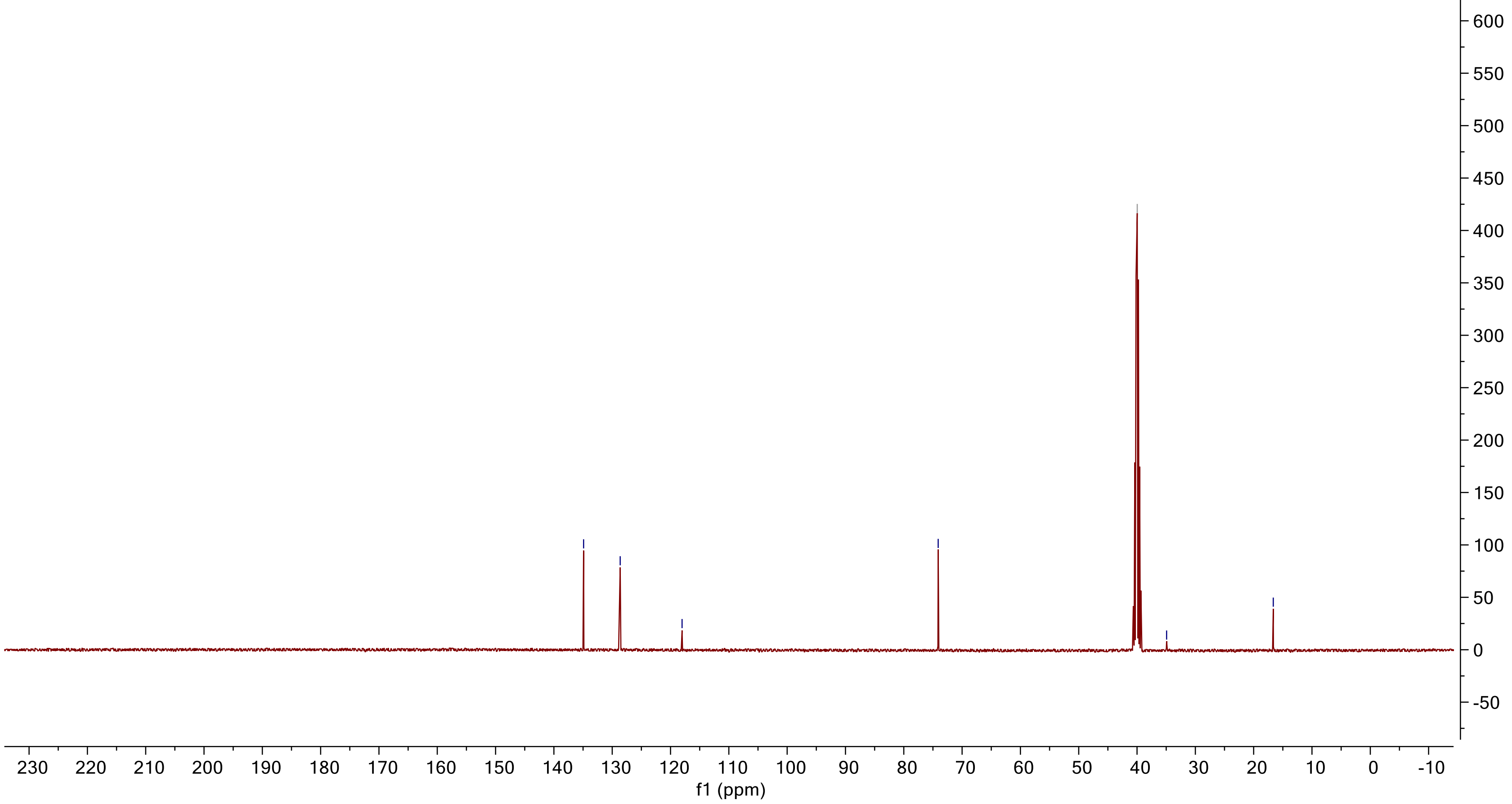
134.91
128.63
118.02
74.10
39.96 DMSO-d6
34.93
16.64
850
800
750
700
650
600
550
500
450
400
350
300
250
200
150
100
50
0
-50
230 220 210 200 190 180 170 160 150 140 130 120 110 100 90 80 70 60 50 40 30 20 10 0 -10
f1 (ppm)

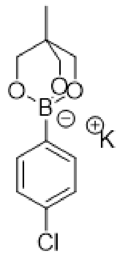

2i

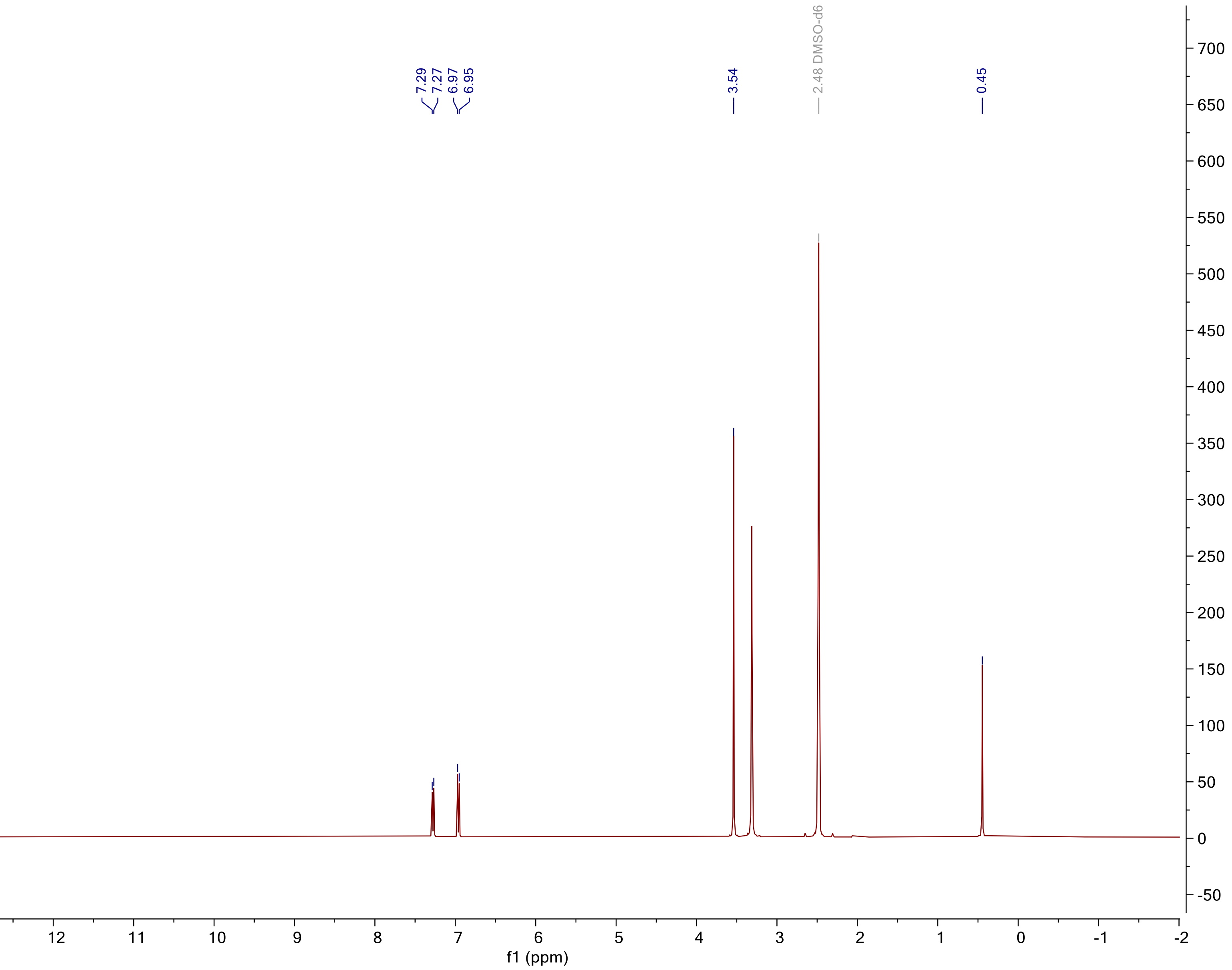

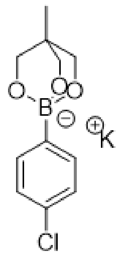

2i

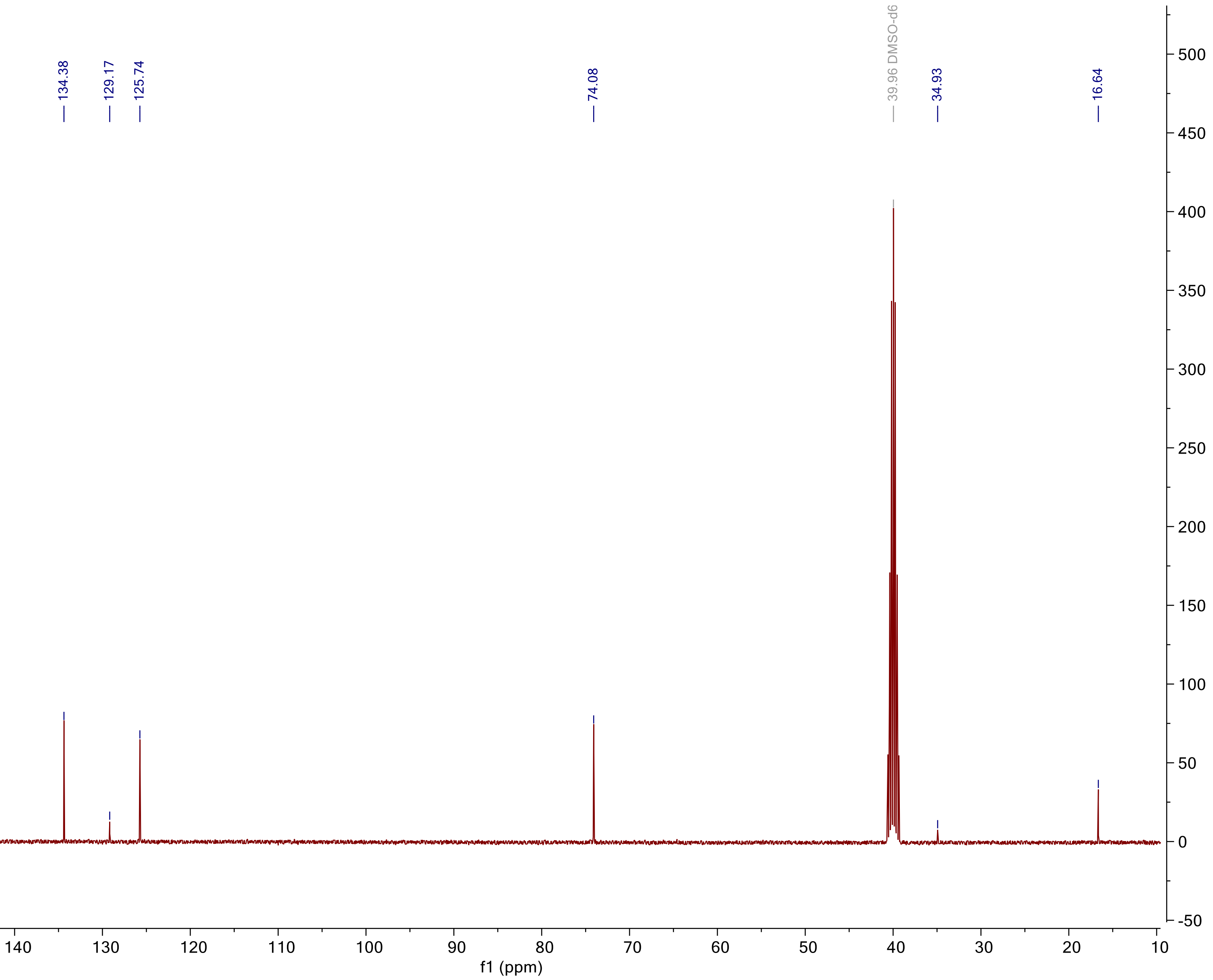

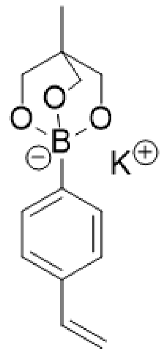
O
O
O
B
K

2j

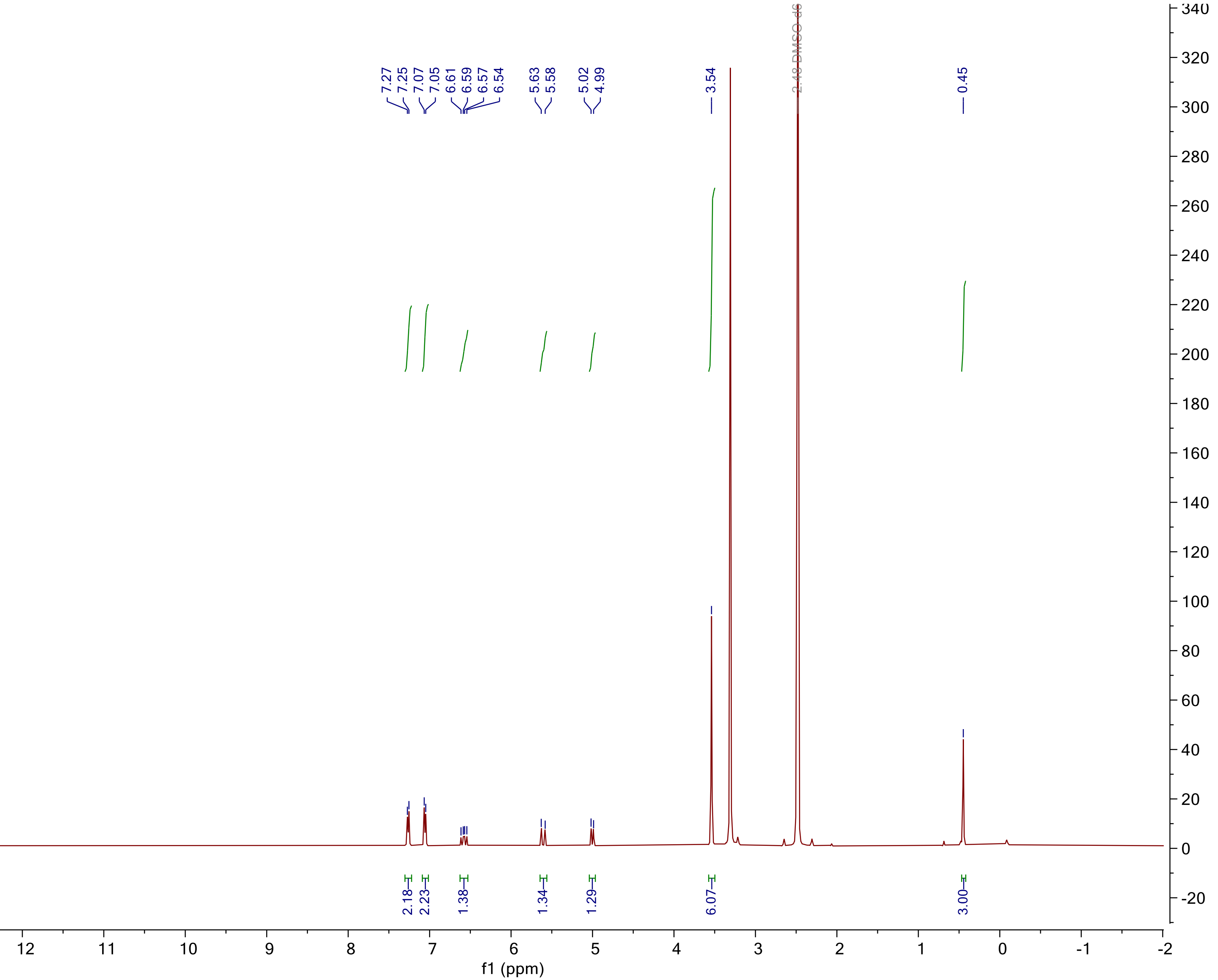
7.27
7.25
7.07
7.05
6.61
6.59
6.57
6.54
5.63
5.58
5.02
4.99
3.54
2.48 DMSO-d6
0.45
2.18
2.23
1.38
1.34
1.29
6.07
3.00
f1 (ppm)

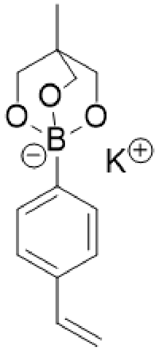

2j

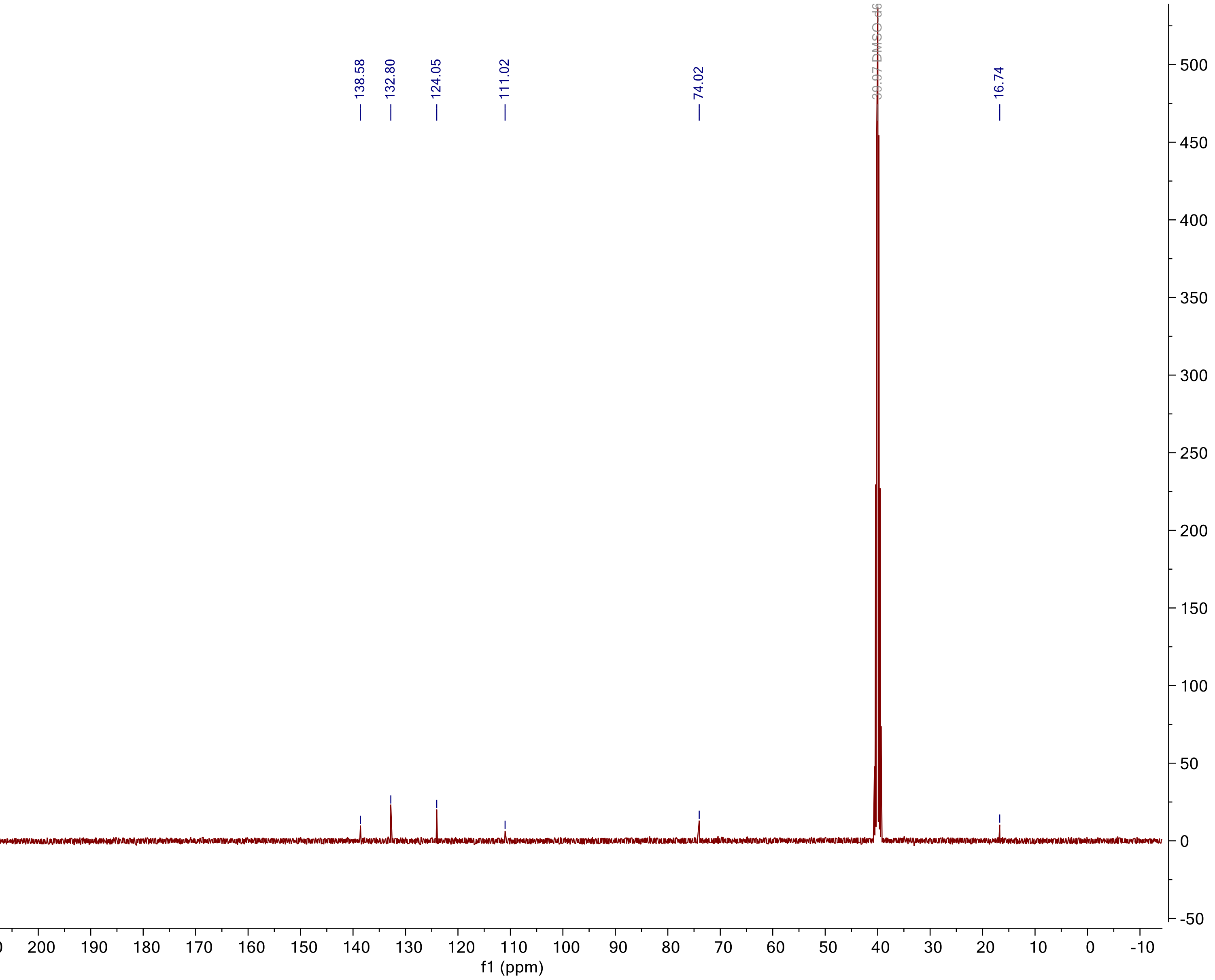

138.58
132.80
124.05
111.02
74.02
39.97 DMSO-d6
16.74
500
450
400
350
300
250
200
150
100
50
0
-50
230 220 210 200 190 180 170 160 150 140 130 120 110 100 90 80 70 60 50 40 30 20 10 0 -10
f1 (ppm)

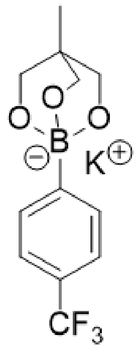
K

2k

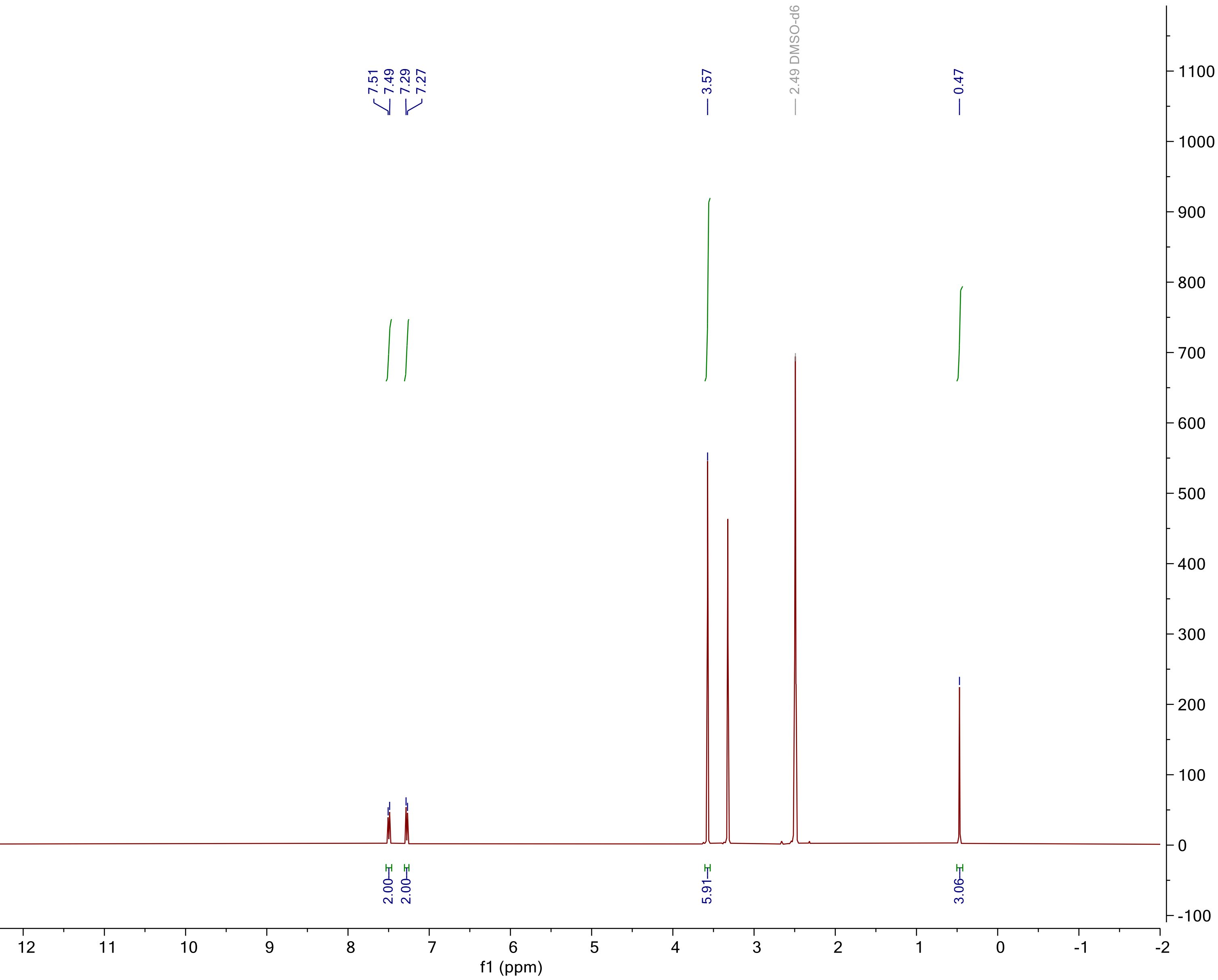
7.51
7.49
7.29
7.27
3.57
2.49 DMSO-d6
0.47
2.00
2.00
5.91
3.06
1100
1000
900
800
700
600
500
400
300
200
100
0
-100
14
13
12
11
10
9
8
7
6
5
4
3
2
1
0
-1
-2
f1 (ppm)

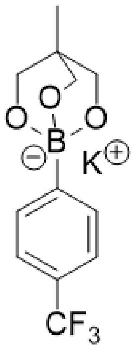

2k

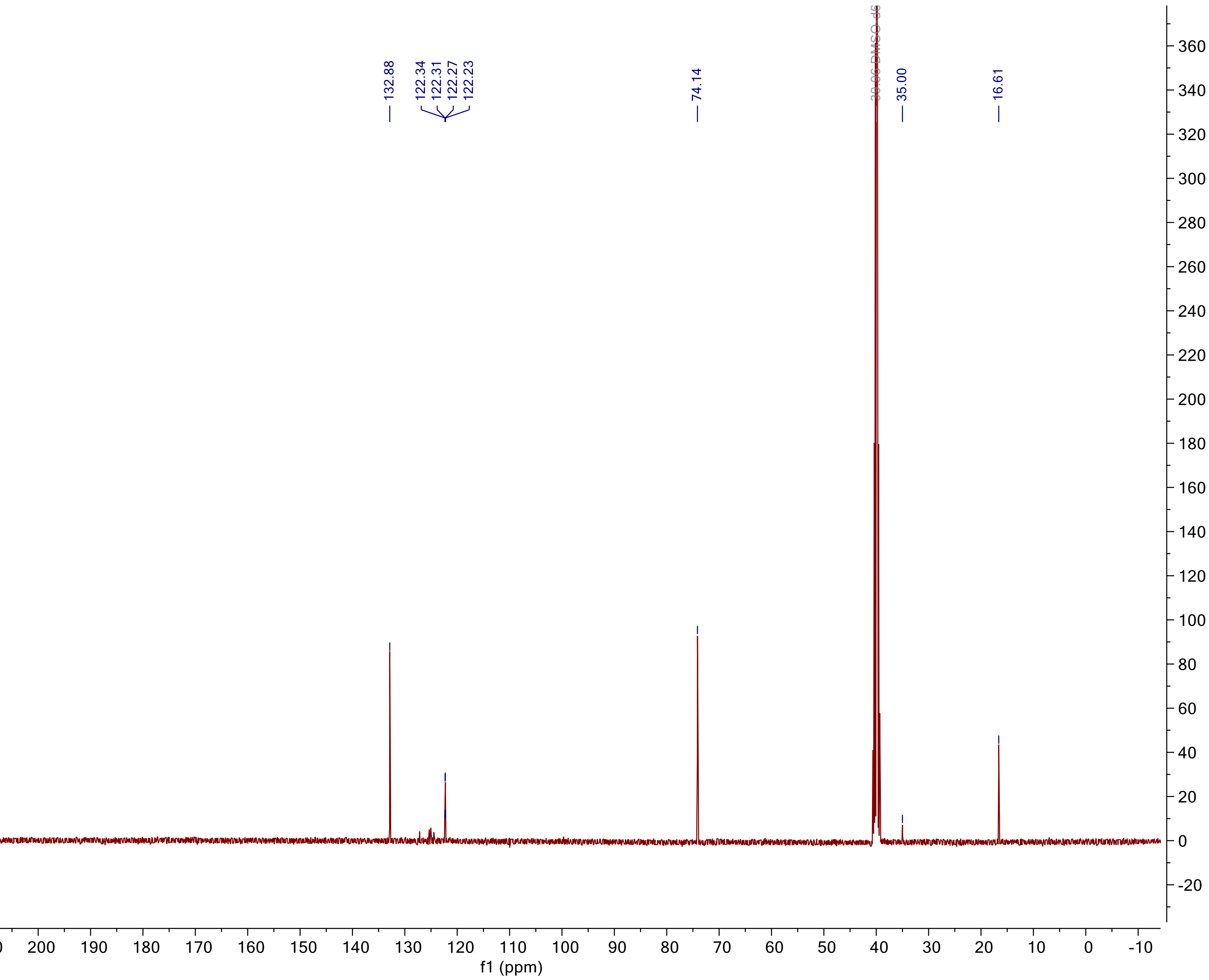

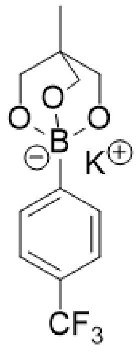

2k

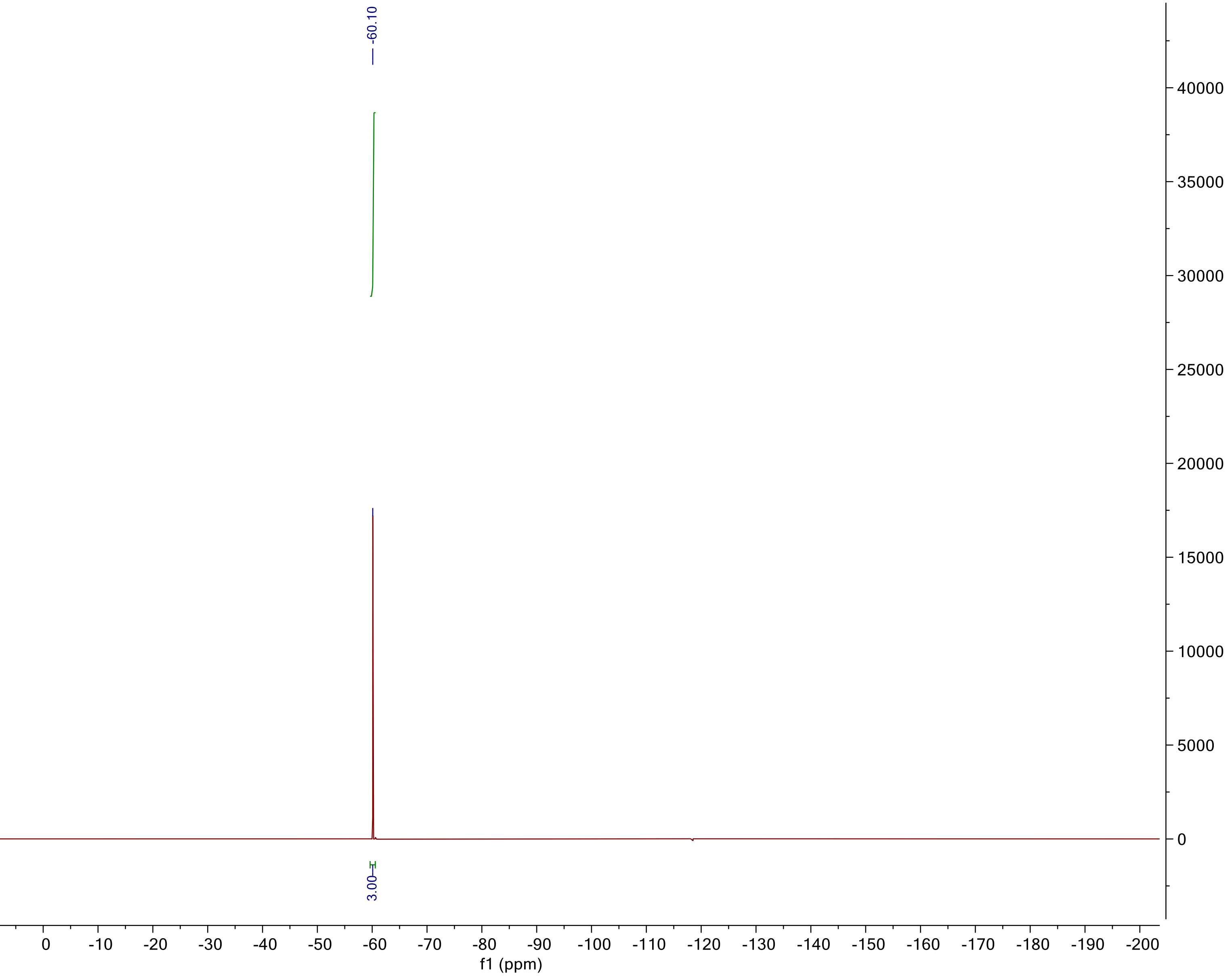

-60.10
3.00
40000
35000
30000
25000
20000
15000
10000
5000
0
30 20 10 0 -10 -20 -30 -40 -50 -60 -70 -80 -90 -100 -110 -120 -130 -140 -150 -160 -170 -180 -190 -200
f1 (ppm)

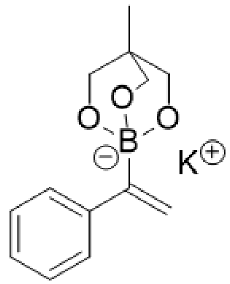

2m

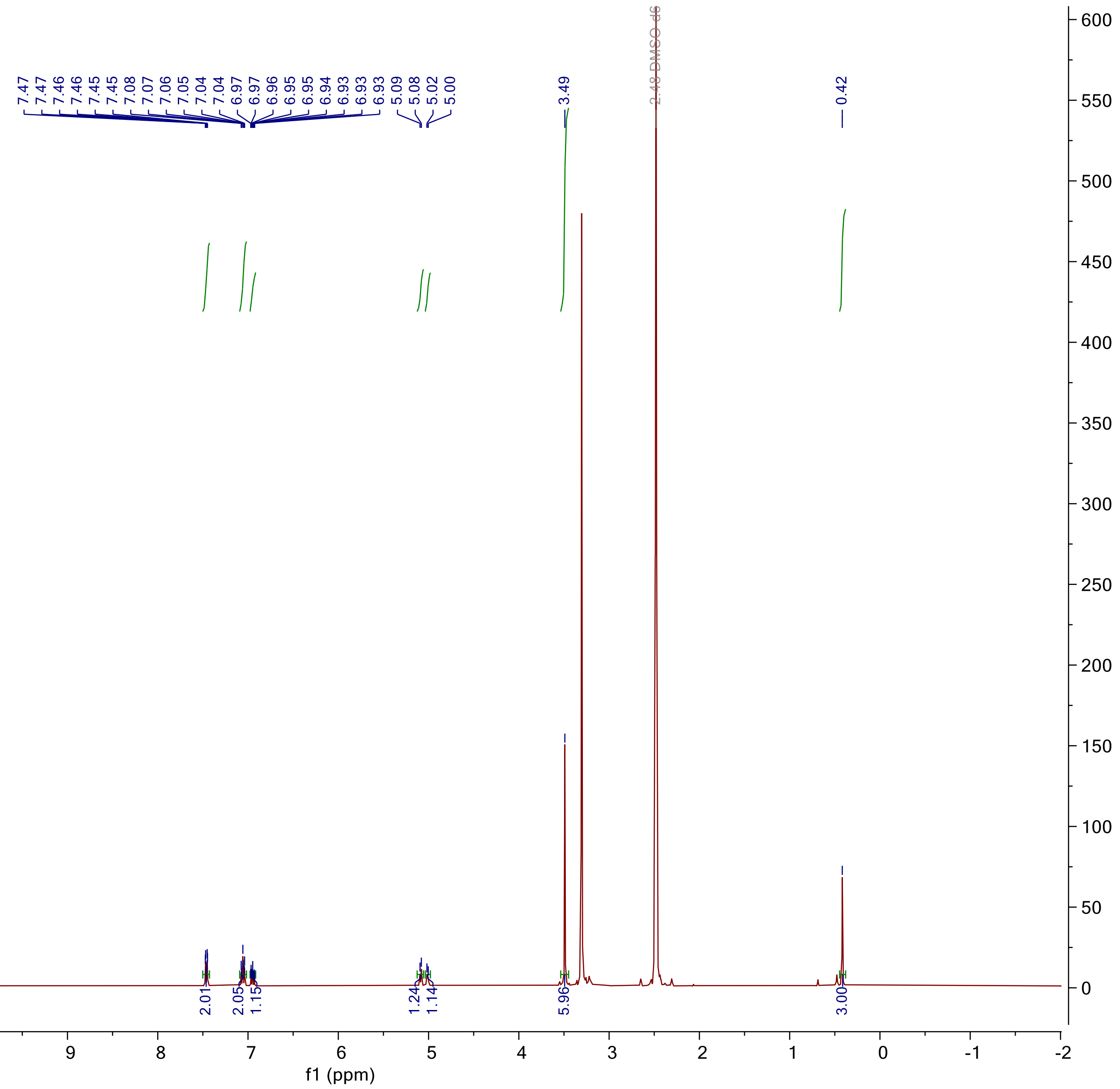
7.47
7.47
7.46
7.45
7.45
7.08
7.07
7.06
7.05
7.04
7.04
6.97
6.97
6.96
6.95
6.95
6.94
6.93
6.93
6.93
5.09
5.08
5.02
5.00
3.49
2.48 DMSO-d6
0.42
2.01
2.05
1.15
1.24
1.14
5.96
3.00
f1 (ppm)

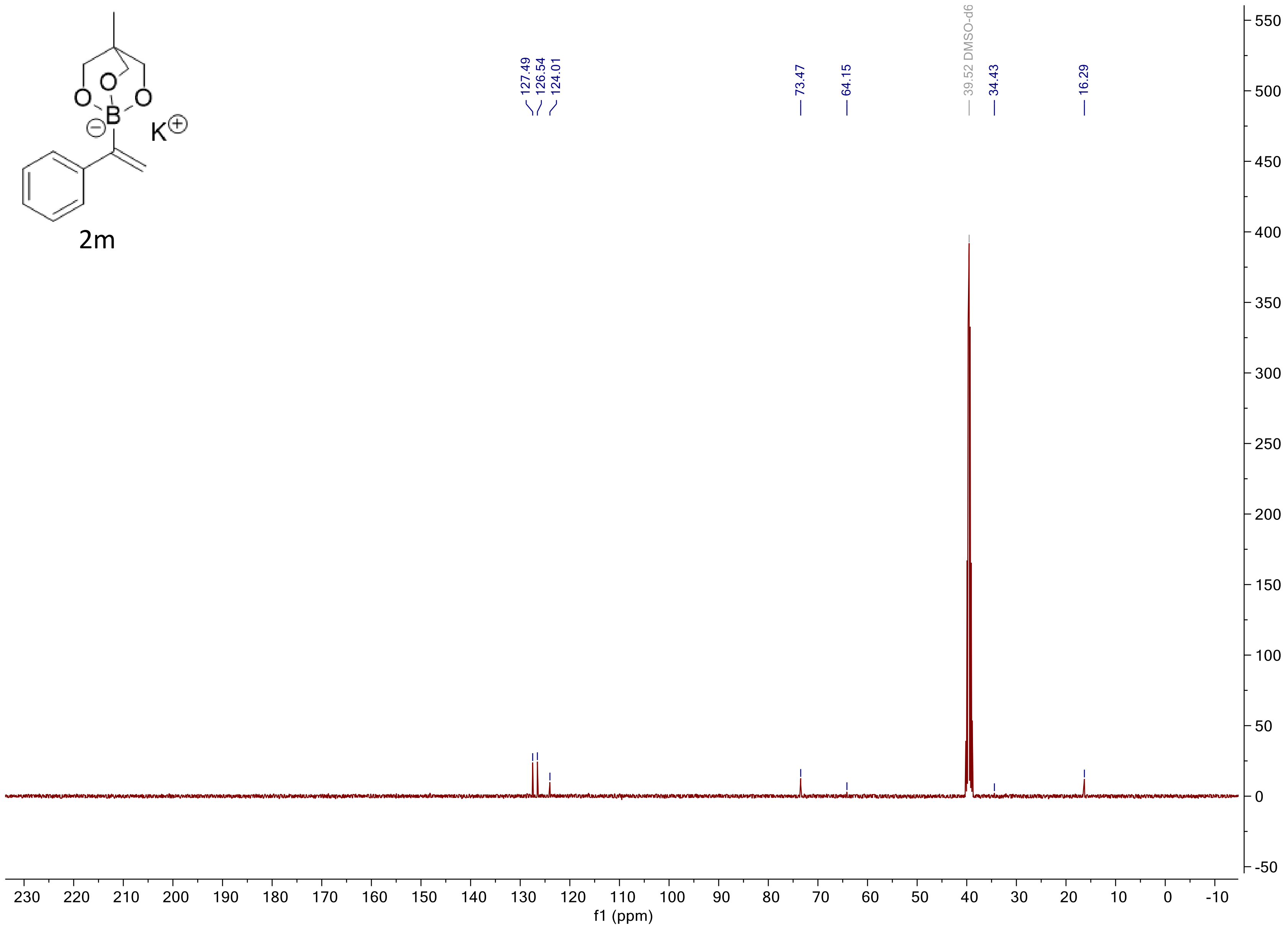

2m
127.49
126.54
124.01
73.47
64.15
39.52 DMSO-d6
34.43
16.29
f1 (ppm)

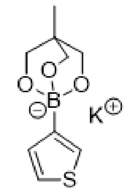

2n

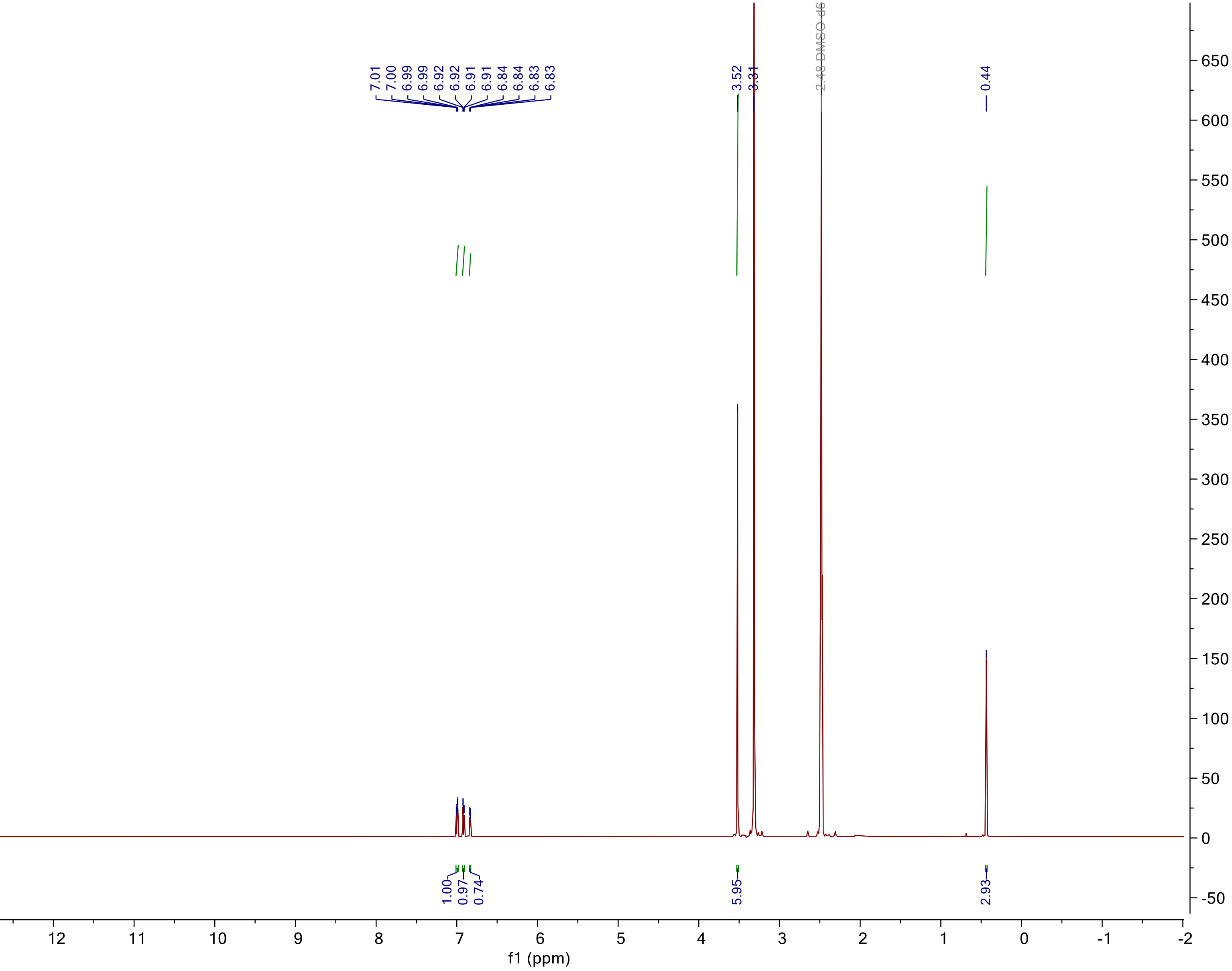

7.01
7.00
6.99
6.99
6.92
6.92
6.91
6.91
6.84
6.84
6.83
6.83
3.52
3.31
2.48 DMSO-d6
0.44
1.00
0.97
0.74
5.95
2.93
f1 (ppm)

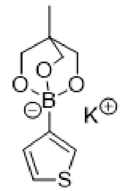

2n

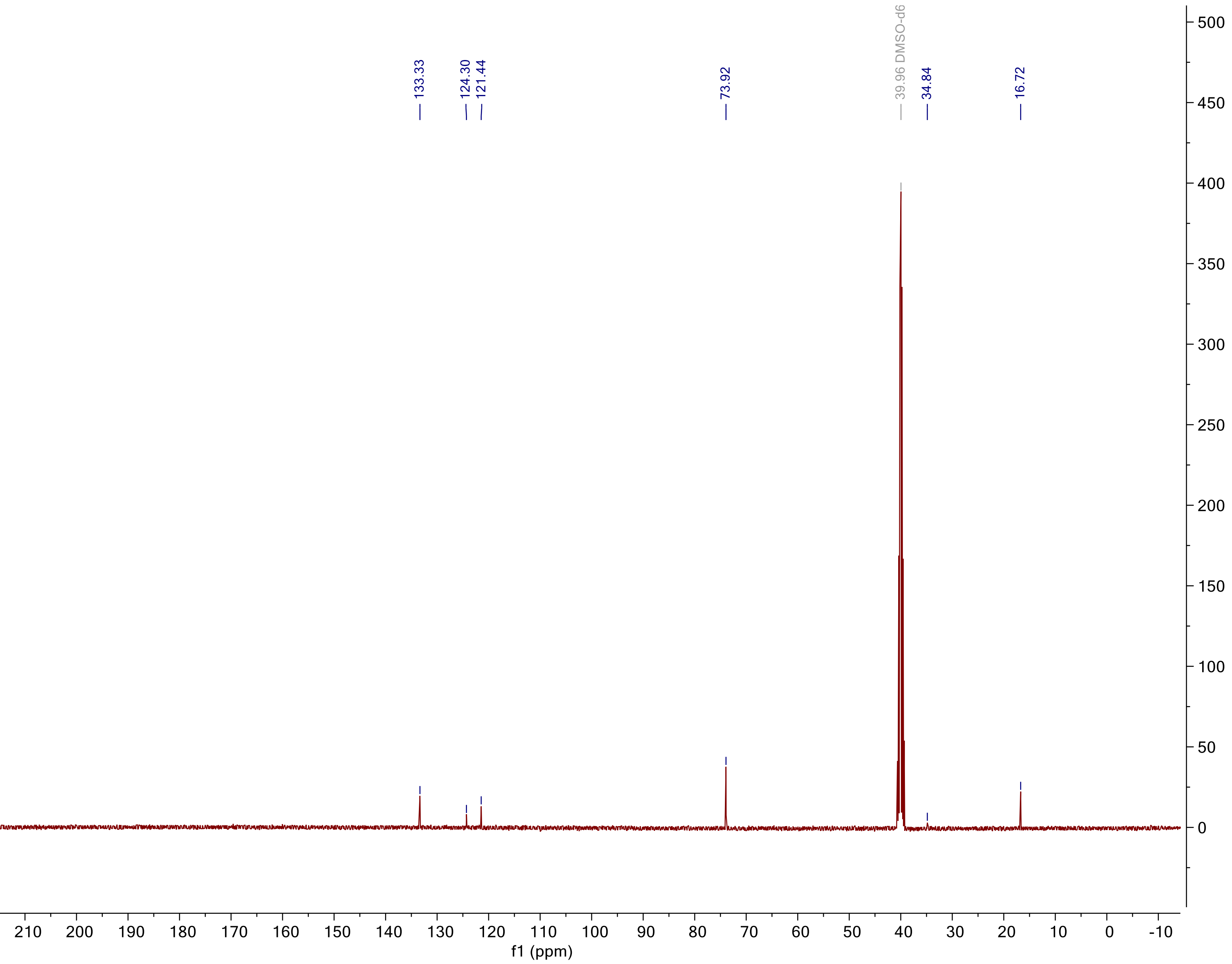
133.33
124.30
121.44
73.92
39.96 DMSO-d6
34.84
16.72
500
450
400
350
300
250
200
150
100
50
0
230 220 210 200 190 180 170 160 150 140 130 120 110 100 90 80 70 60 50 40 30 20 10 0 -10
f1 (ppm)

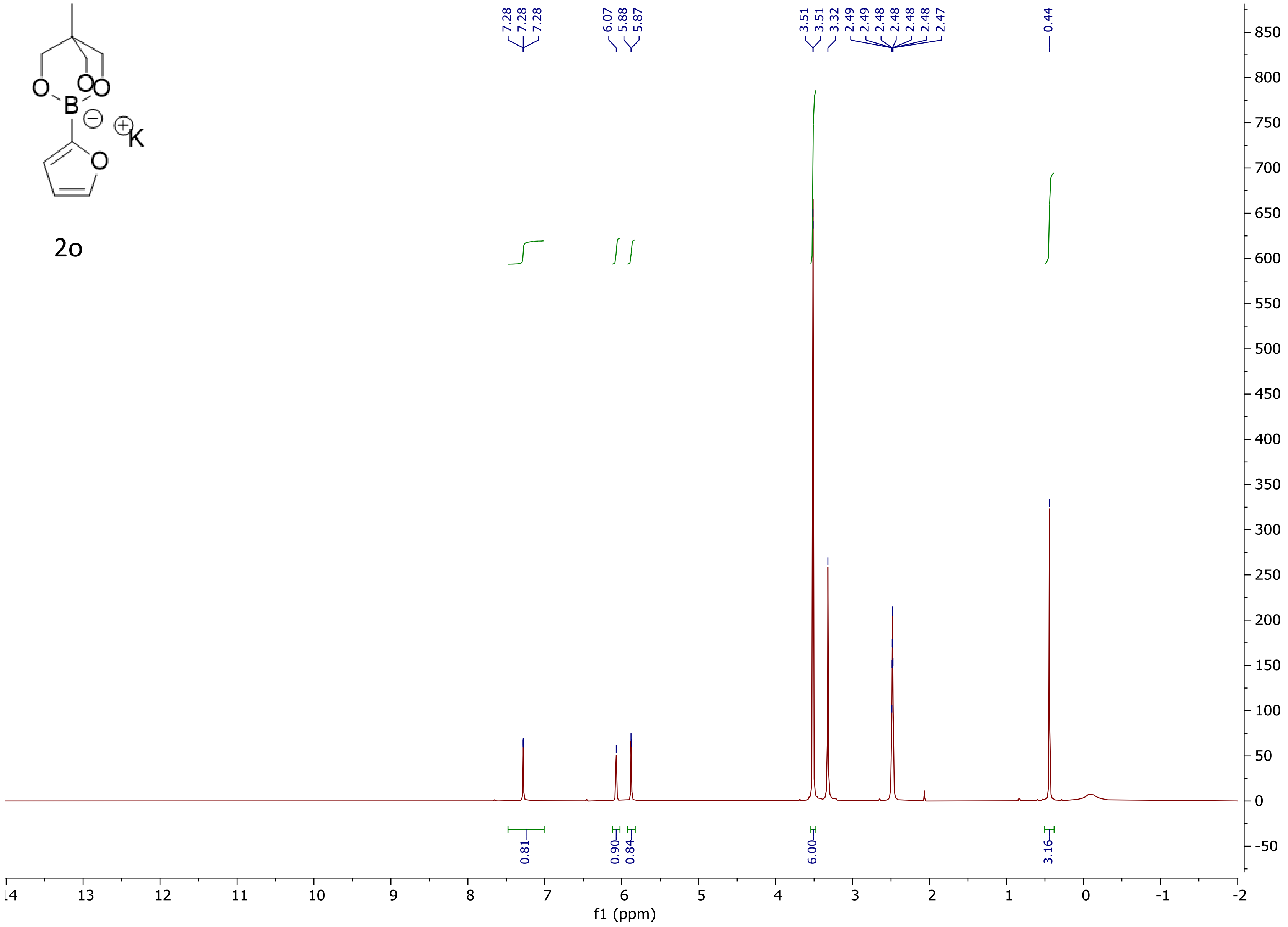
O
B
O
O
K
O
2o
7.28
7.28
7.28
6.07
5.88
5.87
3.51
3.51
3.32
2.49
2.49
2.48
2.48
2.48
2.48
2.47
0.44
0.81
0.90
0.84
6.00
3.16
f1 (ppm)

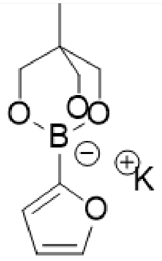

2o

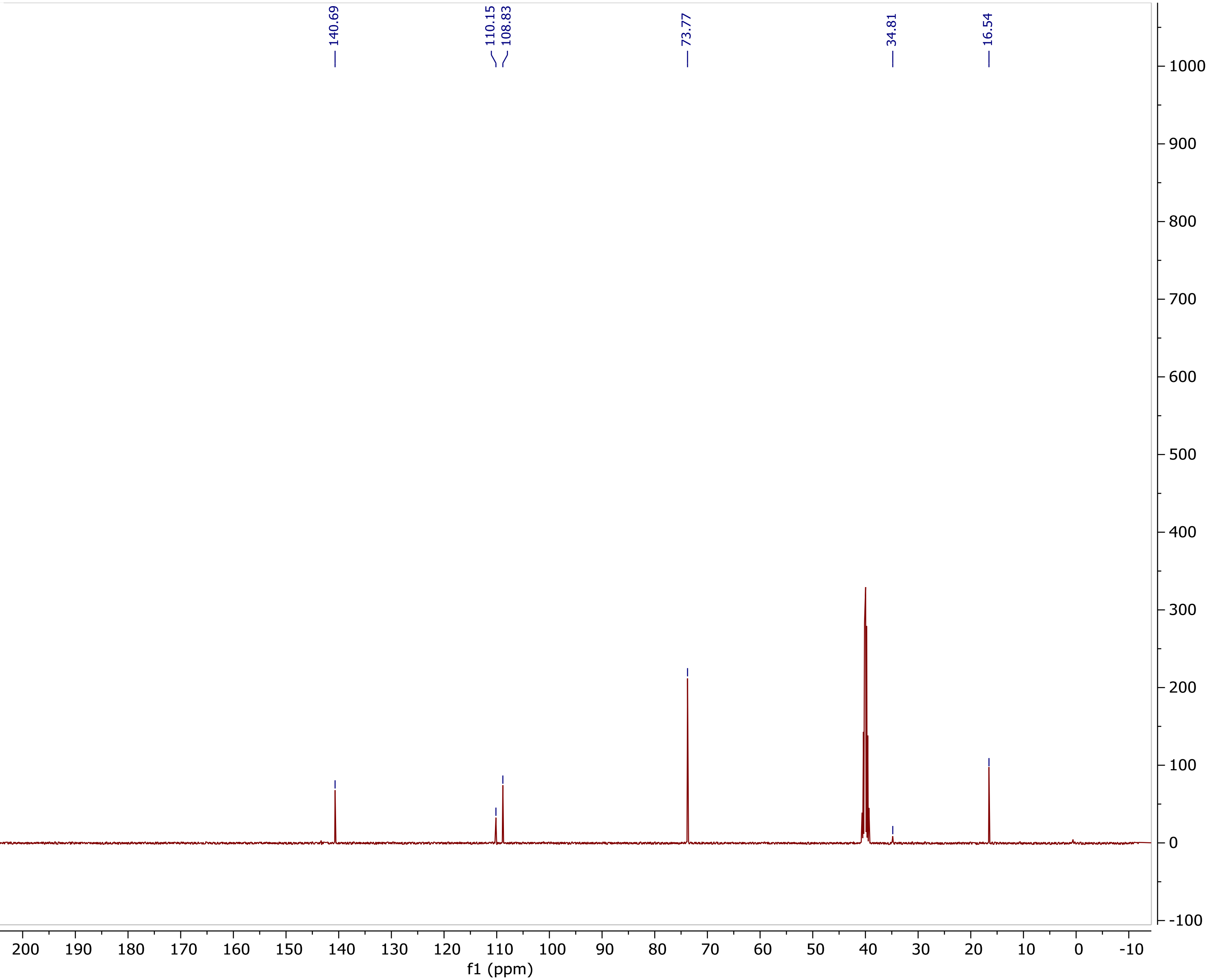

140.69
110.15
108.83
73.77
34.81
16.54
1000
900
800
700
600
500
400
300
200
100
0
-100
230 220 210 200 190 180 170 160 150 140 130 120 110 100 90 80 70 60 50 40 30 20 10 0 -10
f1 (ppm)

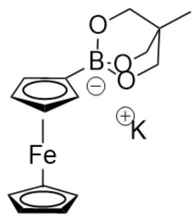

2p

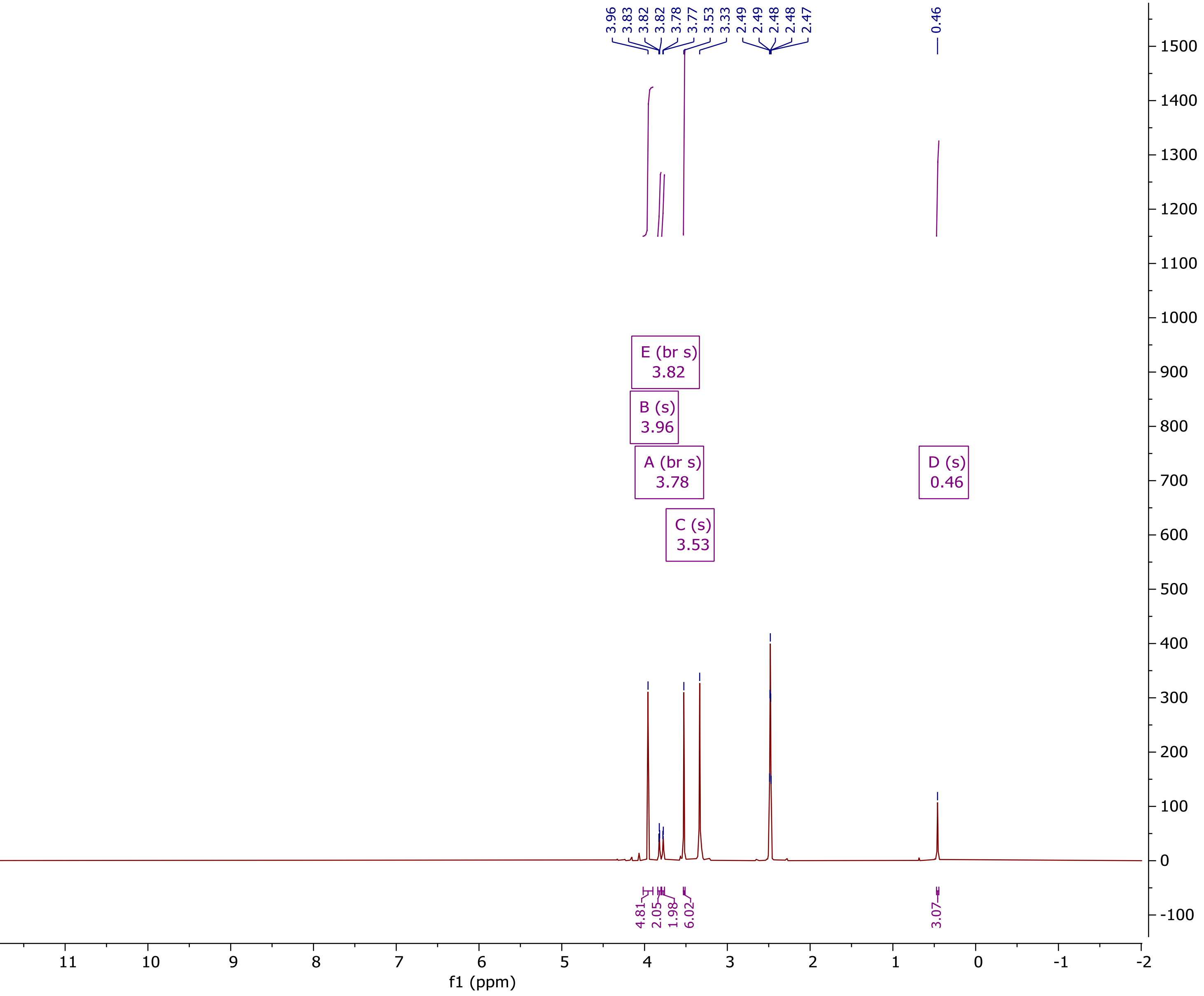
3.96
3.83
3.82
3.82
3.78
3.77
3.53
3.33
2.49
2.49
2.48
2.48
2.47
0.46
E (br s)
3.82
B (s)
3.96
A (br s)
3.78
D (s)
0.46
C (s)
3.53
4.81
2.05
1.98
6.02
3.07
f1 (ppm)

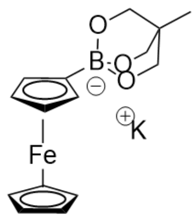

2p

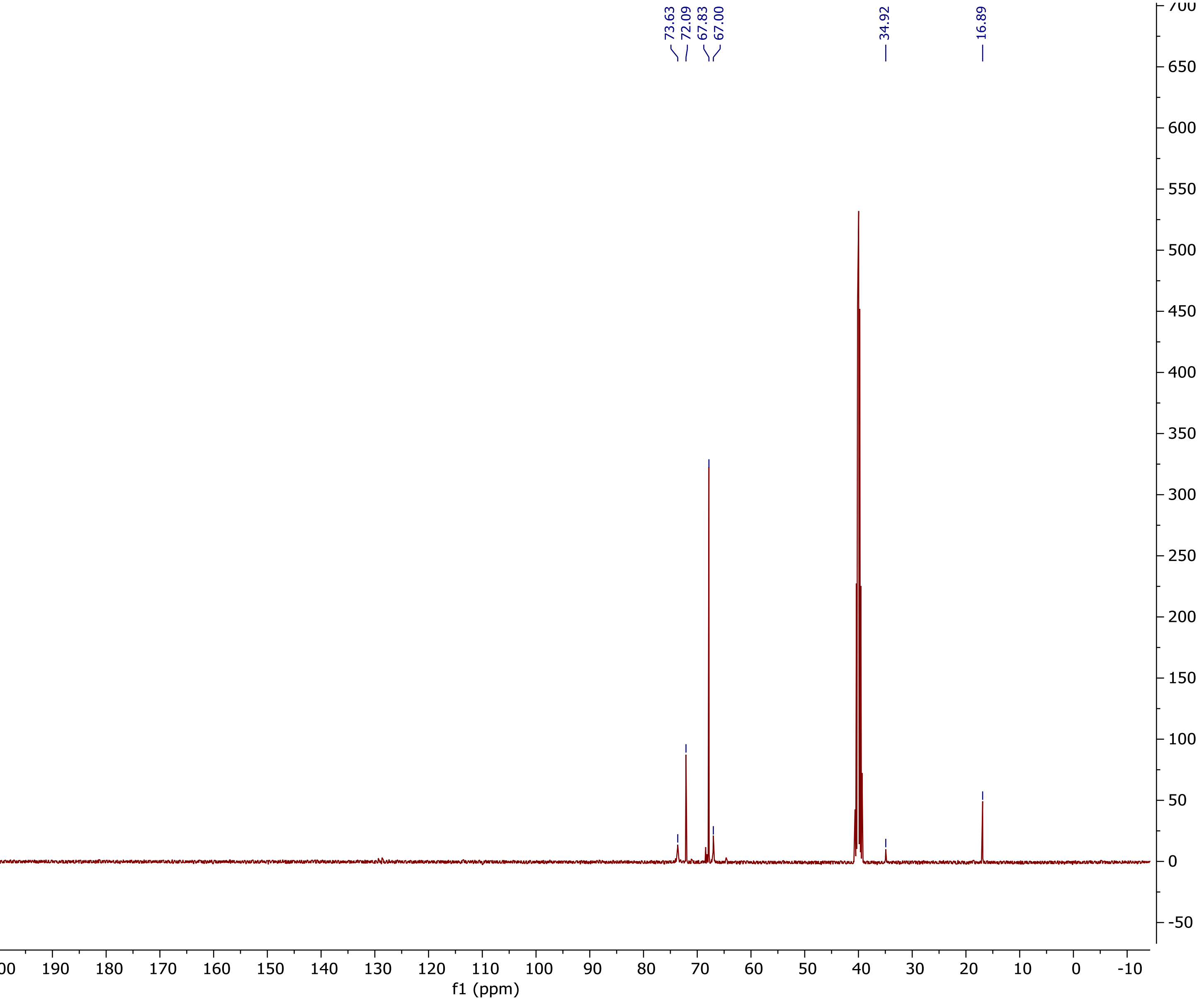

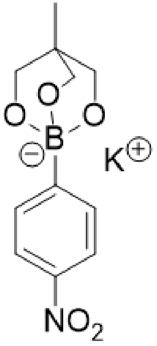
O
O
O
B
K
NO2

2q

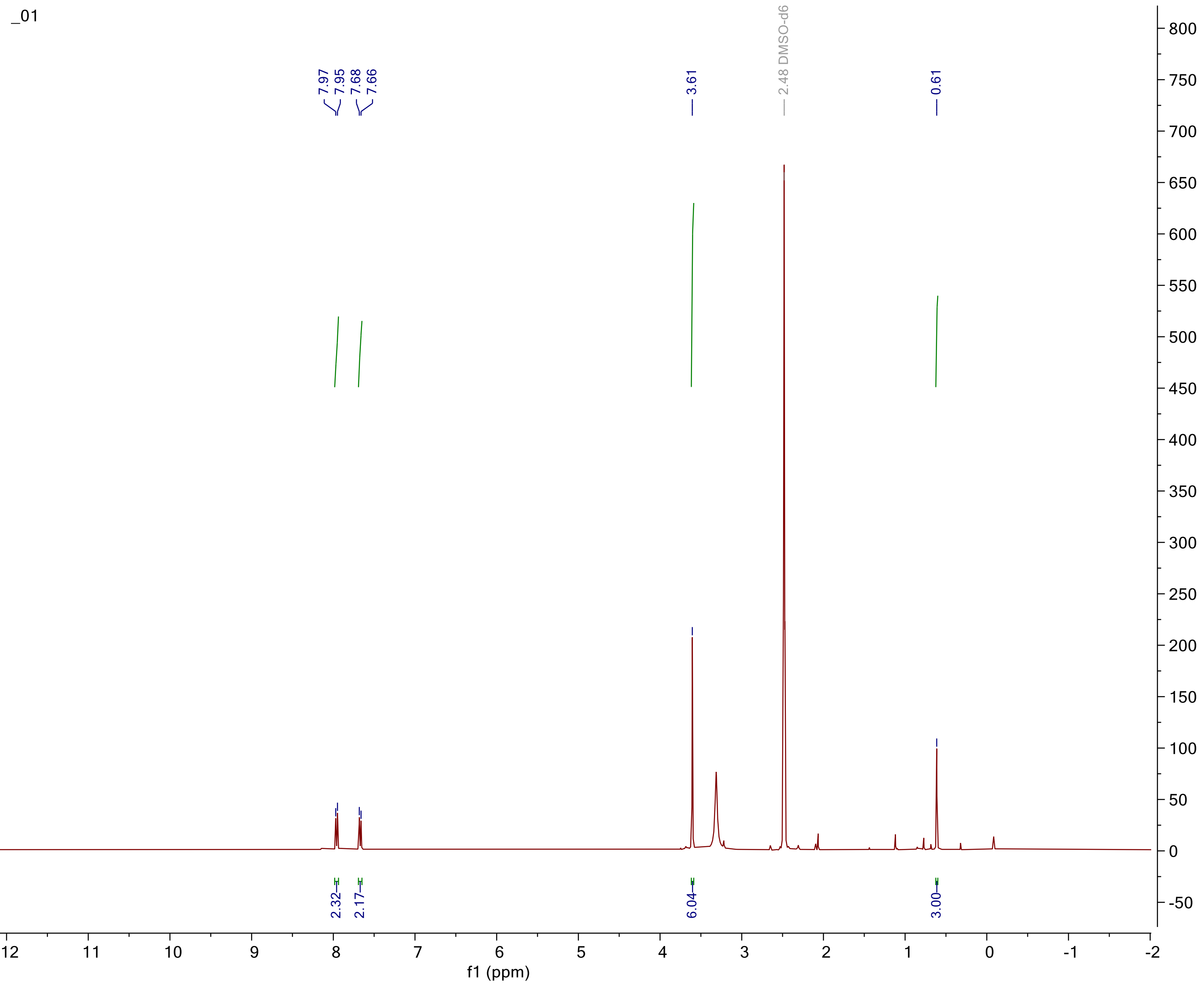
_01
7.97
7.95
7.68
7.66
3.61
2.48 DMSO-d6
0.61
2.32
2.17
6.04
3.00
800
750
700
650
600
550
500
450
400
350
300
250
200
150
100
50
0
-50
14
13
12
11
10
9
8
7
6
5
4
3
2
1
0
-1
-2
f1 (ppm)

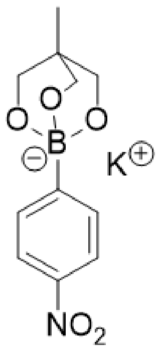
NO2
K⊕

2q

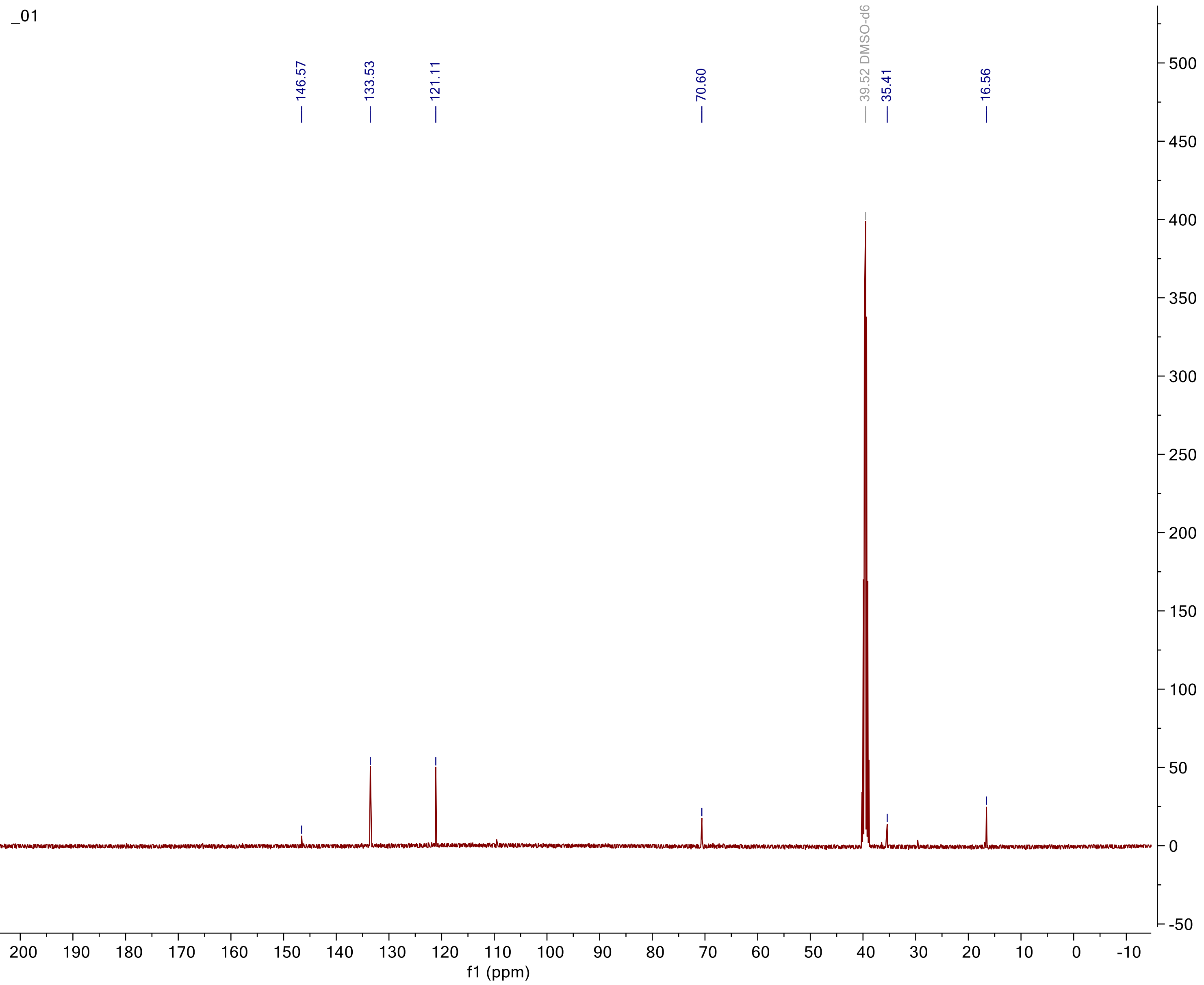
_01
146.57
133.53
121.11
70.60
39.52 DMSO-d6
35.41
16.56
500
450
400
350
300
250
200
150
100
50
0
-50
230
220
210
200
190
180
170
160
150
140
130
120
110
100
90
80
70
60
50
40
30
20
10
0
-10
f1 (ppm)

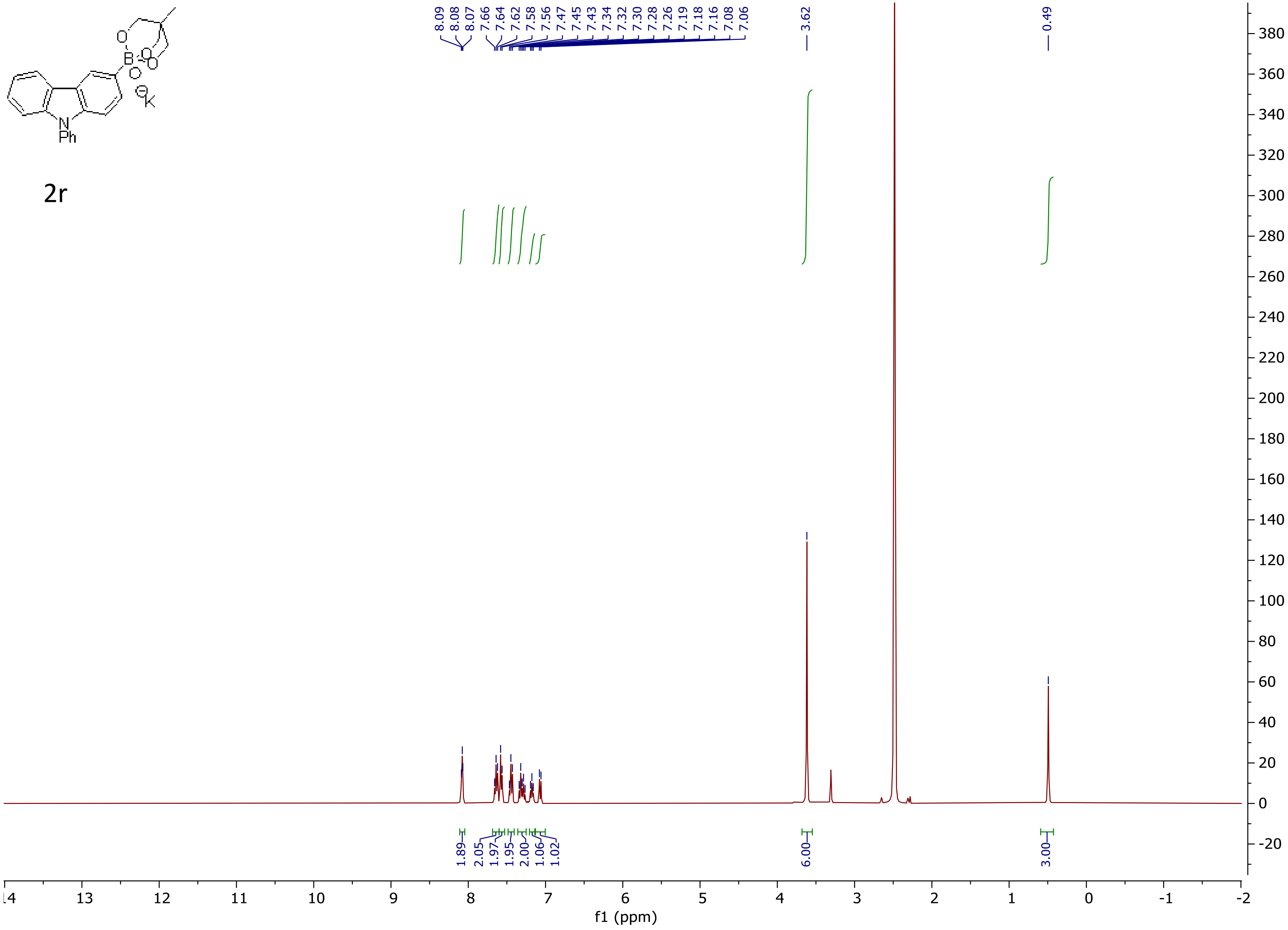
8.09
8.08
8.07
7.66
7.64
7.62
7.58
7.56
7.47
7.45
7.43
7.34
7.32
7.30
7.28
7.26
7.19
7.18
7.16
7.08
7.06
3.62
0.49
1.89
2.05
1.97
1.95
2.00
1.06
1.02
6.00
3.00
f1 (ppm)

2r

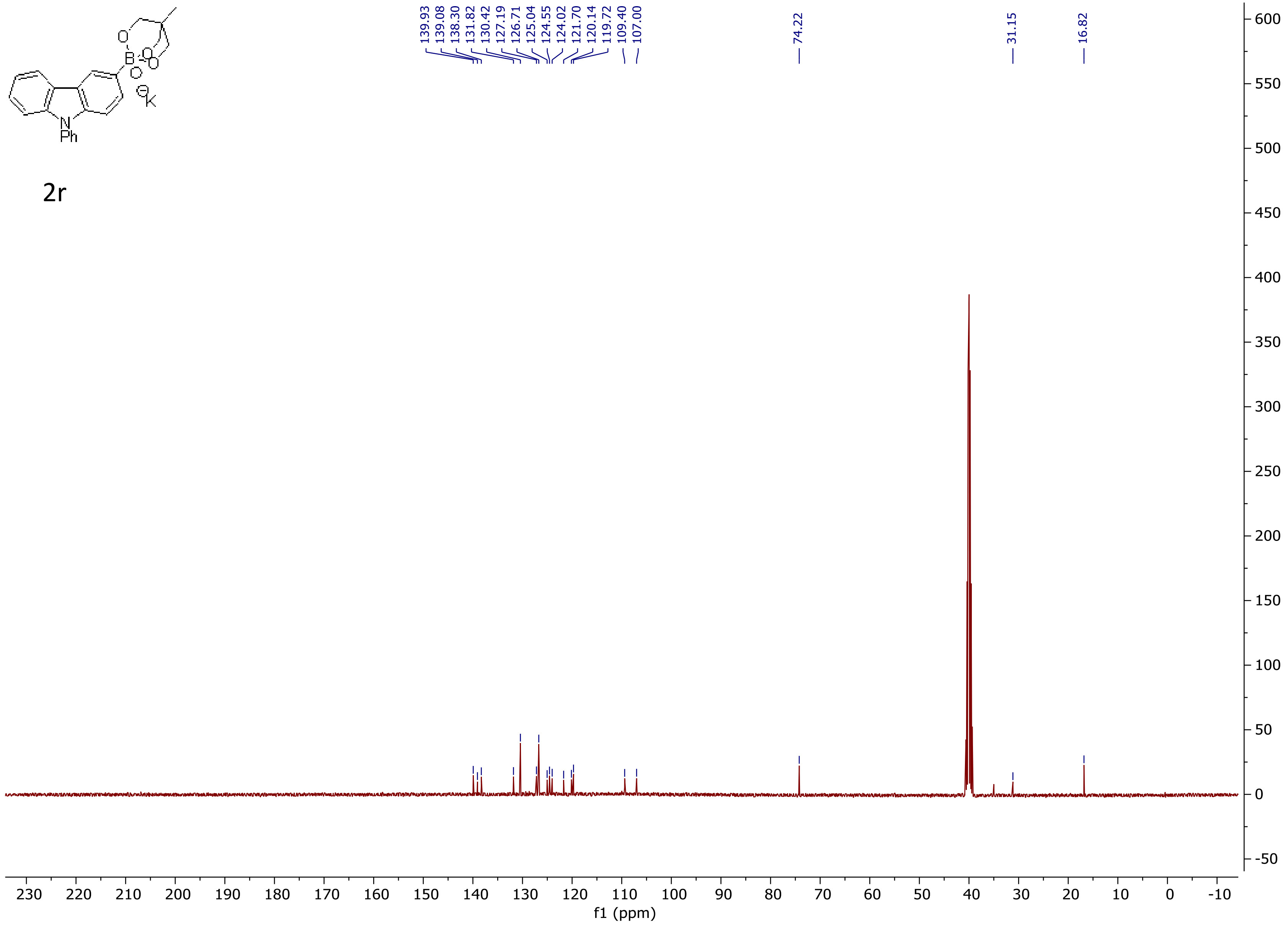

139.93
139.08
138.30
131.82
130.42
127.19
126.71
125.04
124.55
124.02
121.70
120.14
119.72
109.40
107.00
74.22
31.15
16.82
f1 (ppm)

2r

2s

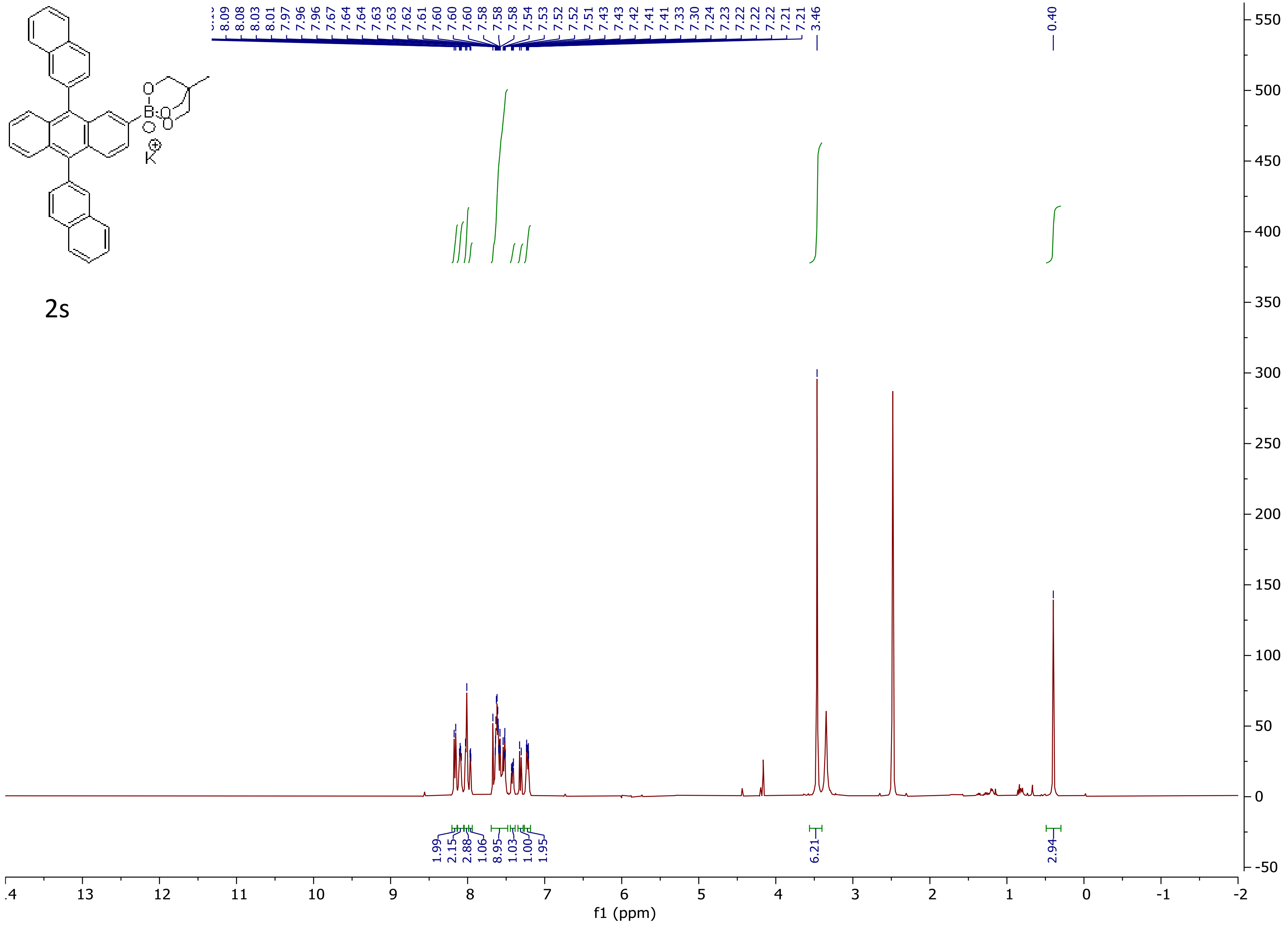

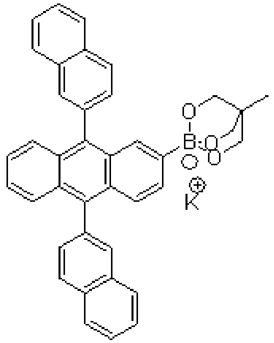

2s

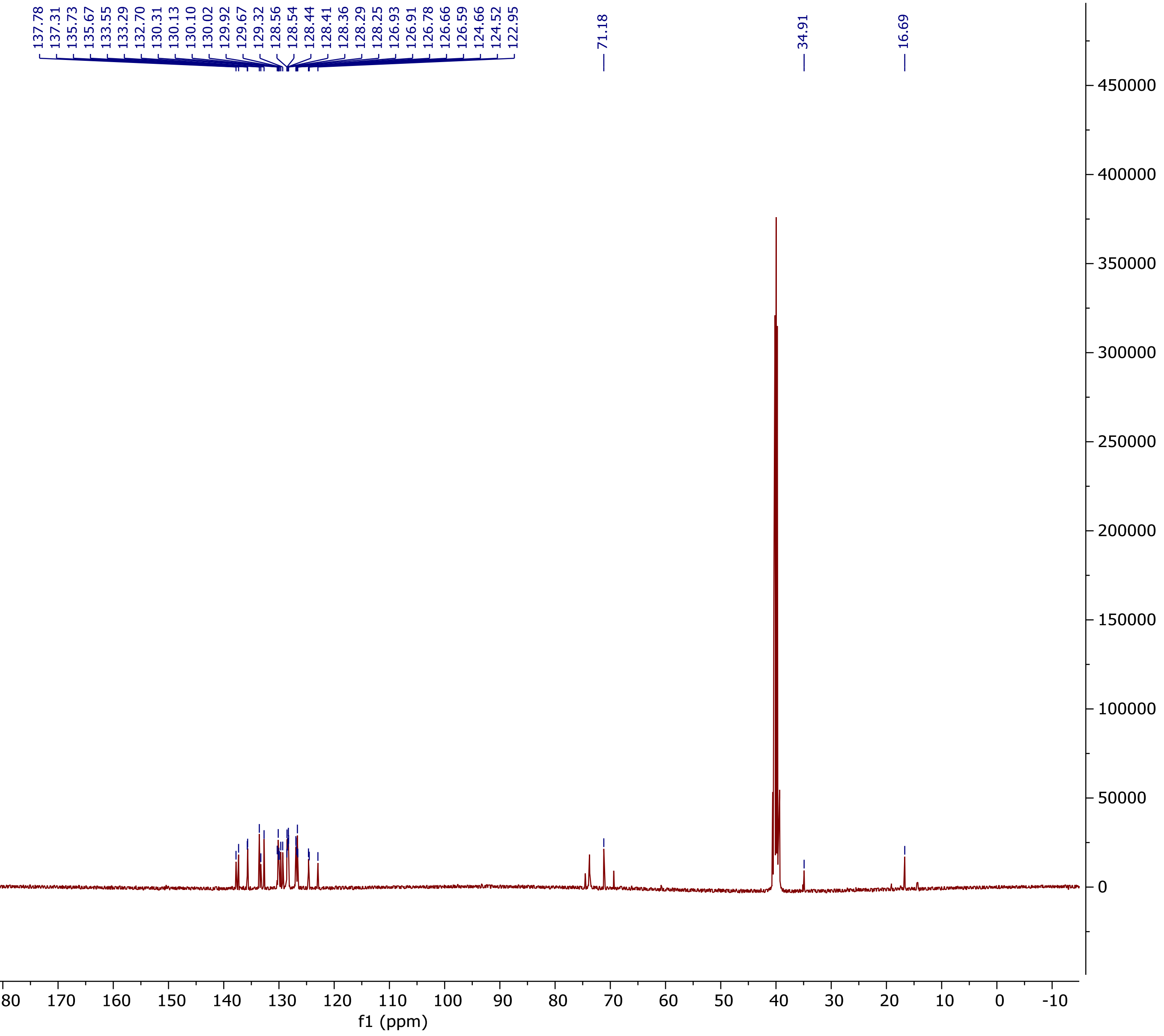
137.78
137.31
135.73
135.67
133.55
133.29
132.70
130.31
130.13
130.10
130.02
129.92
129.67
129.32
128.56
128.54
128.44
128.41
128.36
128.29
128.25
126.93
126.91
126.78
126.66
126.59
124.66
124.52
122.95
71.18
34.91
16.69
f1 (ppm)

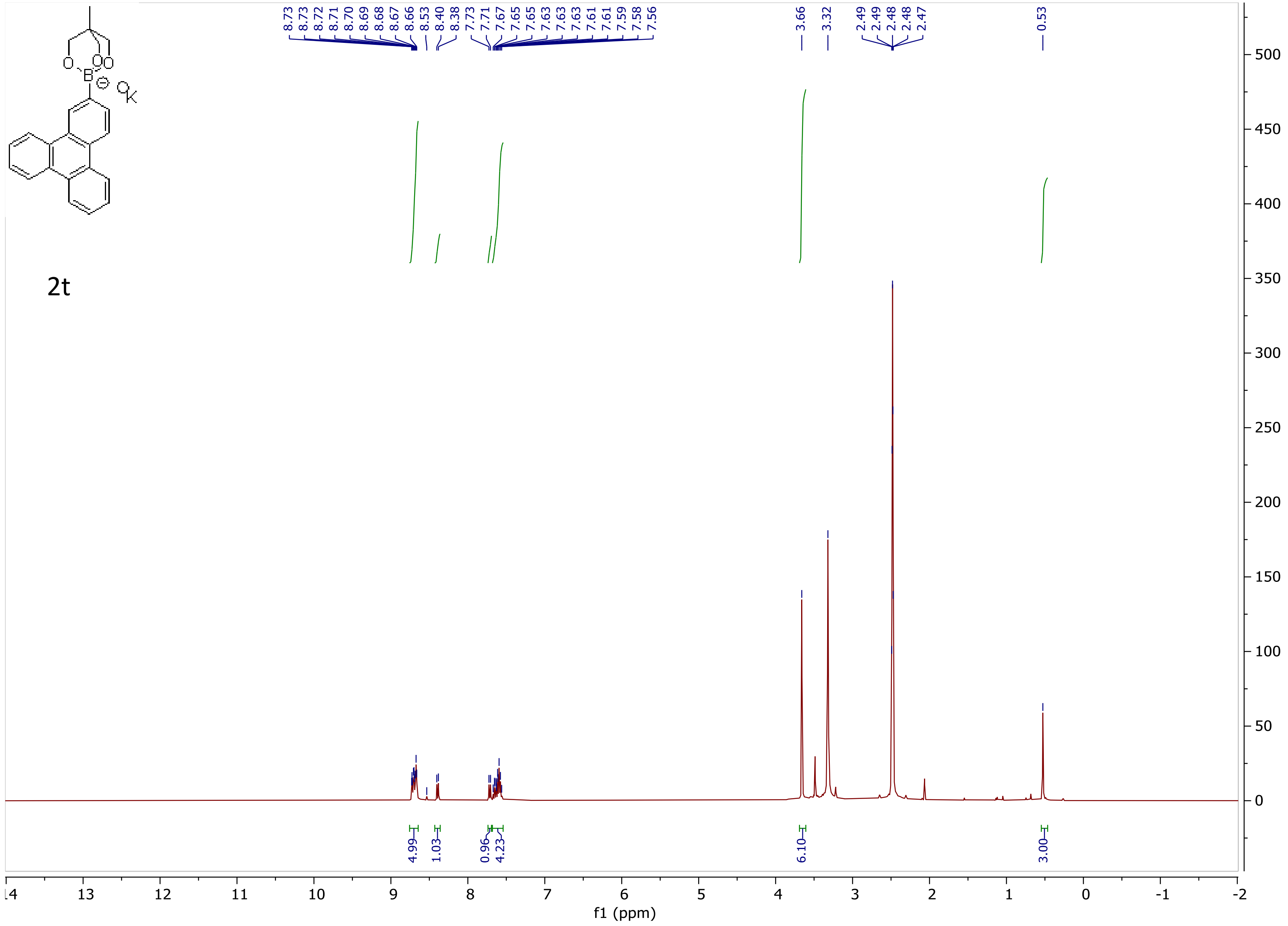
2t
8.73
8.73
8.72
8.71
8.70
8.69
8.68
8.67
8.66
8.53
8.40
8.38
7.73
7.71
7.67
7.65
7.65
7.63
7.63
7.63
7.61
7.61
7.59
7.58
7.56
3.66
3.32
2.49
2.49
2.48
2.48
2.47
0.53
4.99
1.03
0.96
4.23
6.10
3.00
f1 (ppm)

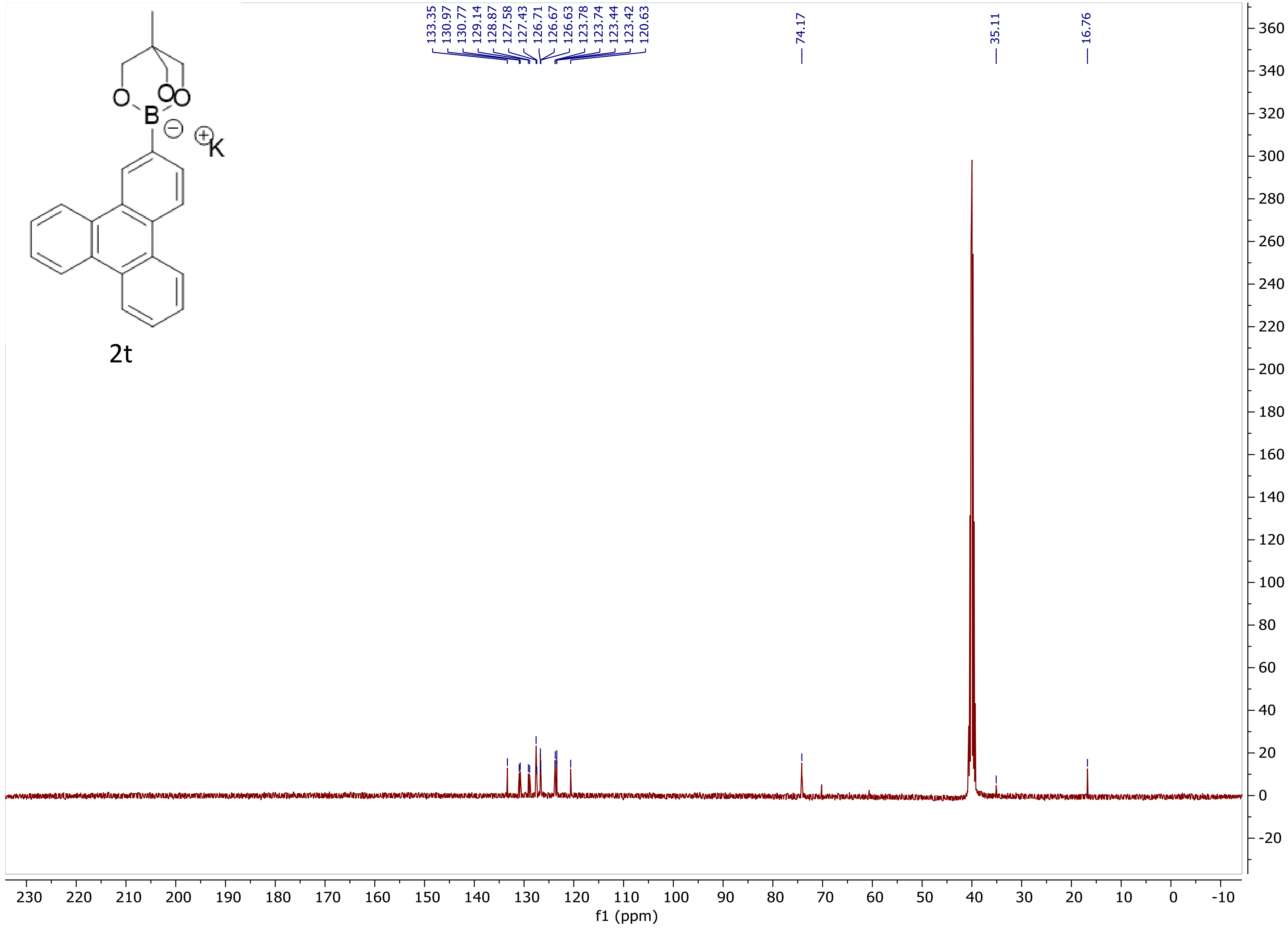

133.35
130.97
130.77
129.14
128.87
127.58
127.43
126.71
126.67
126.63
123.78
123.74
123.44
123.42
120.63
74.17
35.11
16.76
f1 (ppm)

2t

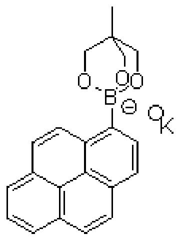

2u

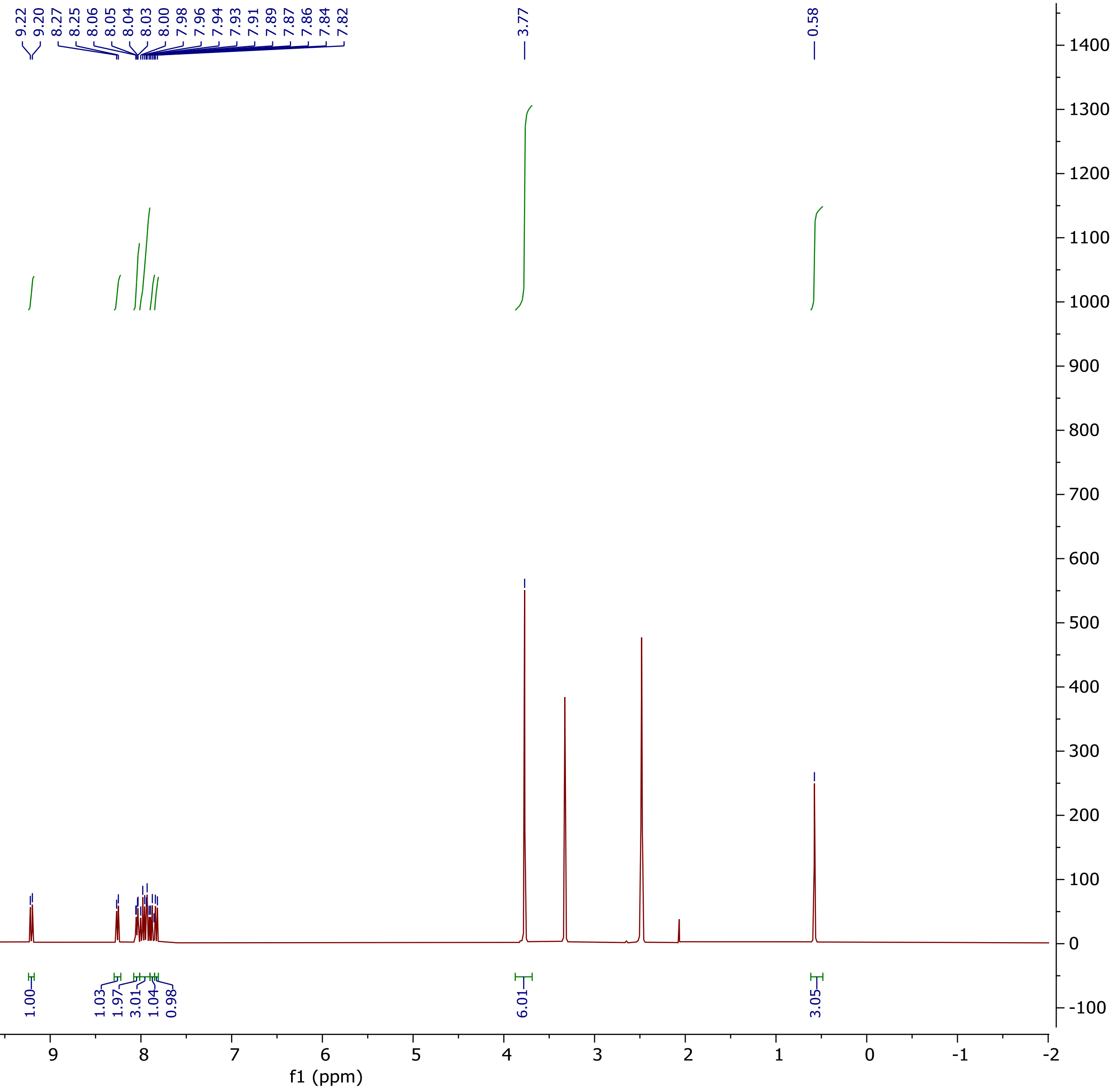

9.22
9.20
8.27
8.25
8.06
8.05
8.04
8.03
8.00
7.98
7.96
7.94
7.93
7.91
7.89
7.87
7.86
7.84
7.82
3.77
0.58
1.00
1.03
1.97
3.01
1.04
0.98
6.01
3.05
f1 (ppm)

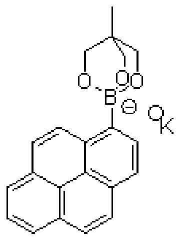

2u

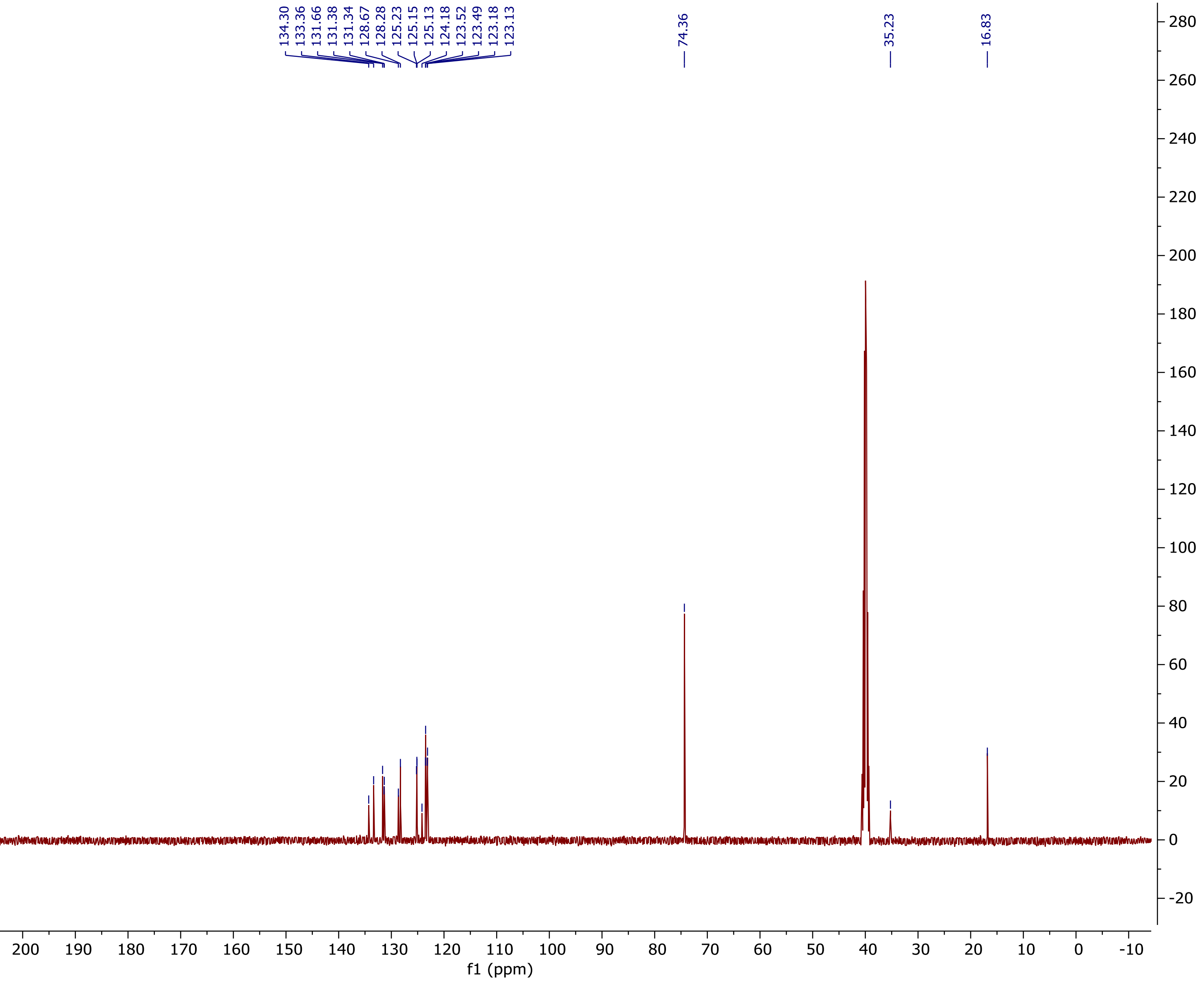
134.30
133.36
131.66
131.38
131.34
128.67
128.28
125.23
125.15
125.13
124.18
123.52
123.49
123.18
123.13
74.36
35.23
16.83
f1 (ppm)

Phosphine/Phosphite Au complexes

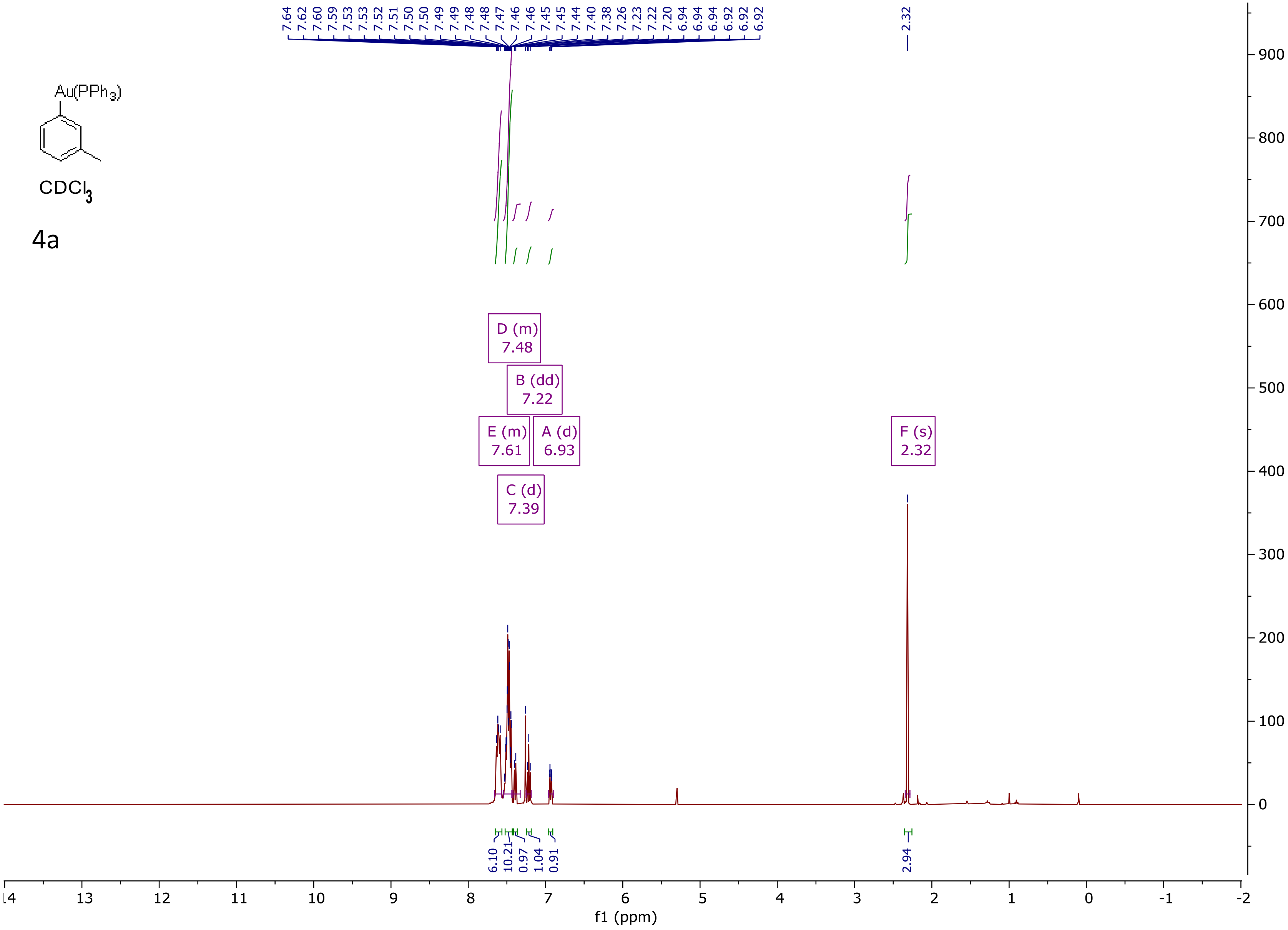

Au(PPh3)
CDCl3
4a
7.64 7.62 7.60 7.59 7.53 7.53 7.52 7.51 7.50 7.50 7.49 7.49 7.48 7.48 7.47 7.46 7.46 7.45 7.45 7.44 7.40 7.38 7.26 7.23 7.22 7.20 6.94 6.94 6.94 6.92 6.92 6.92
2.32
D (m) 7.48
B (dd) 7.22
E (m) 7.61
A (d) 6.93
F (s) 2.32
C (d) 7.39
6.10
10.21
0.97
1.04
0.91
2.94
f1 (ppm)

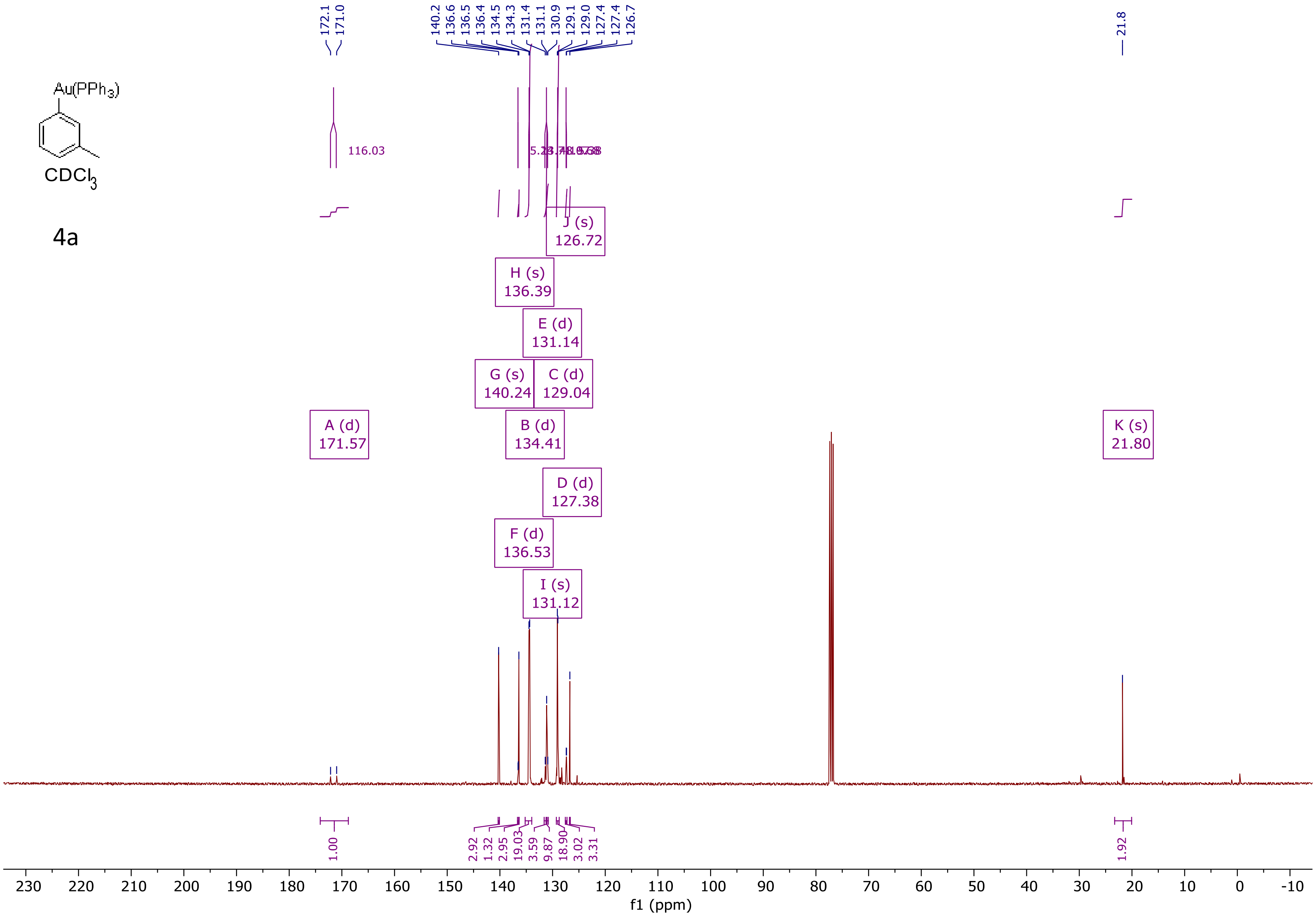
Au(PPh3)
CDCl3
4a
172.1
171.0
140.2
136.6
136.5
136.4
134.5
134.3
131.4
131.1
130.9
129.1
129.0
127.4
127.4
126.7
21.8
116.03
A (d) 171.57
B (d) 134.41
C (d) 129.04
D (d) 127.38
E (d) 131.14
F (d) 136.53
G (s) 140.24
H (s) 136.39
I (s) 131.12
J (s) 126.72
K (s) 21.80
1.00
2.92
1.32
2.95
19.03
3.59
9.87
18.90
3.02
3.31
1.92
f1 (ppm)

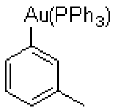

Au(PPh3)

CDCl3

4a

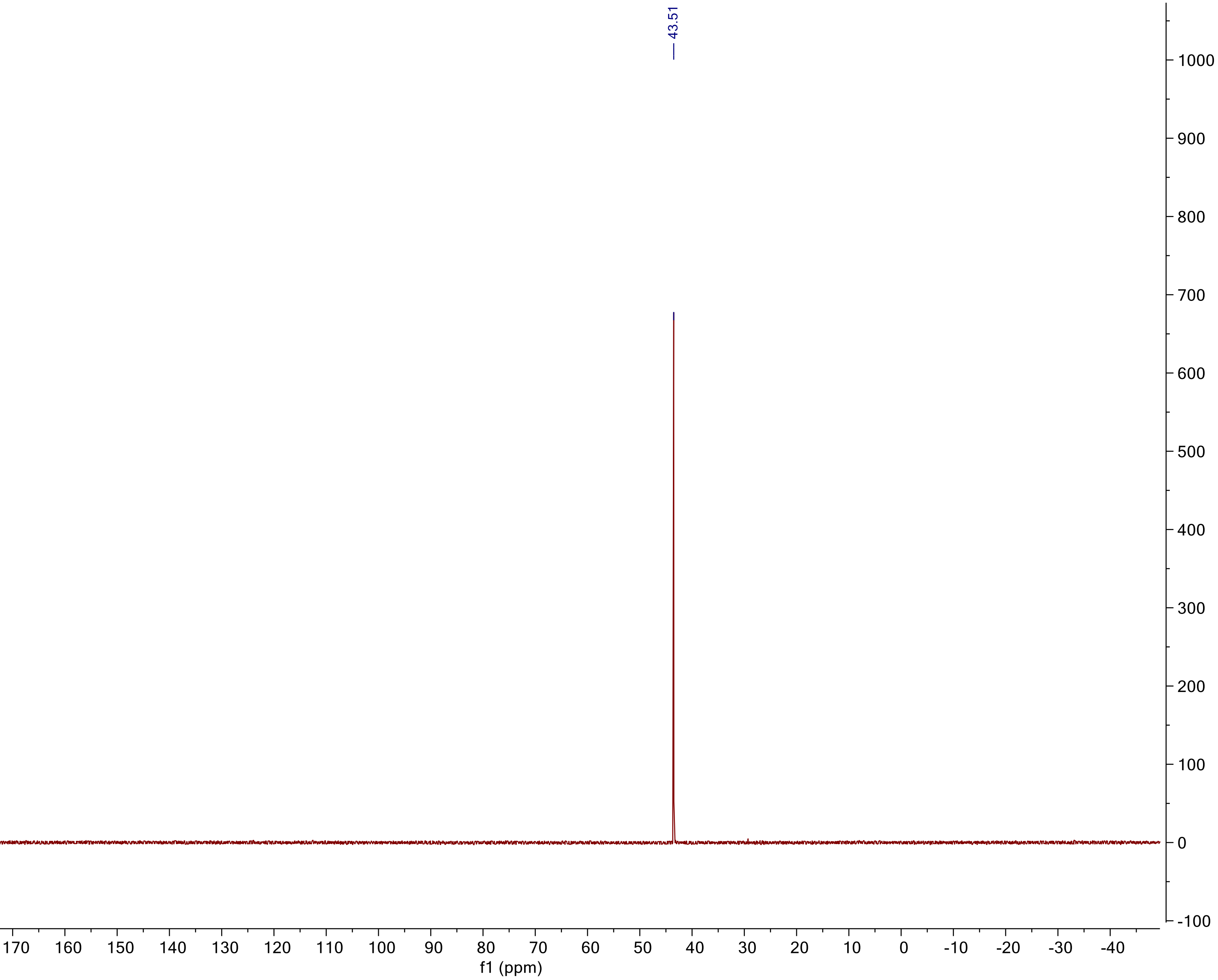

43.51
1000
900
800
700
600
500
400
300
200
100
0
-100
190
180
170
160
150
140
130
120
110
100
90
80
70
60
50
40
30
20
10
0
-10
-20
-30
-40
f1 (ppm)

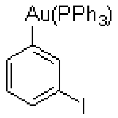
Au(PPh3)
I

4e

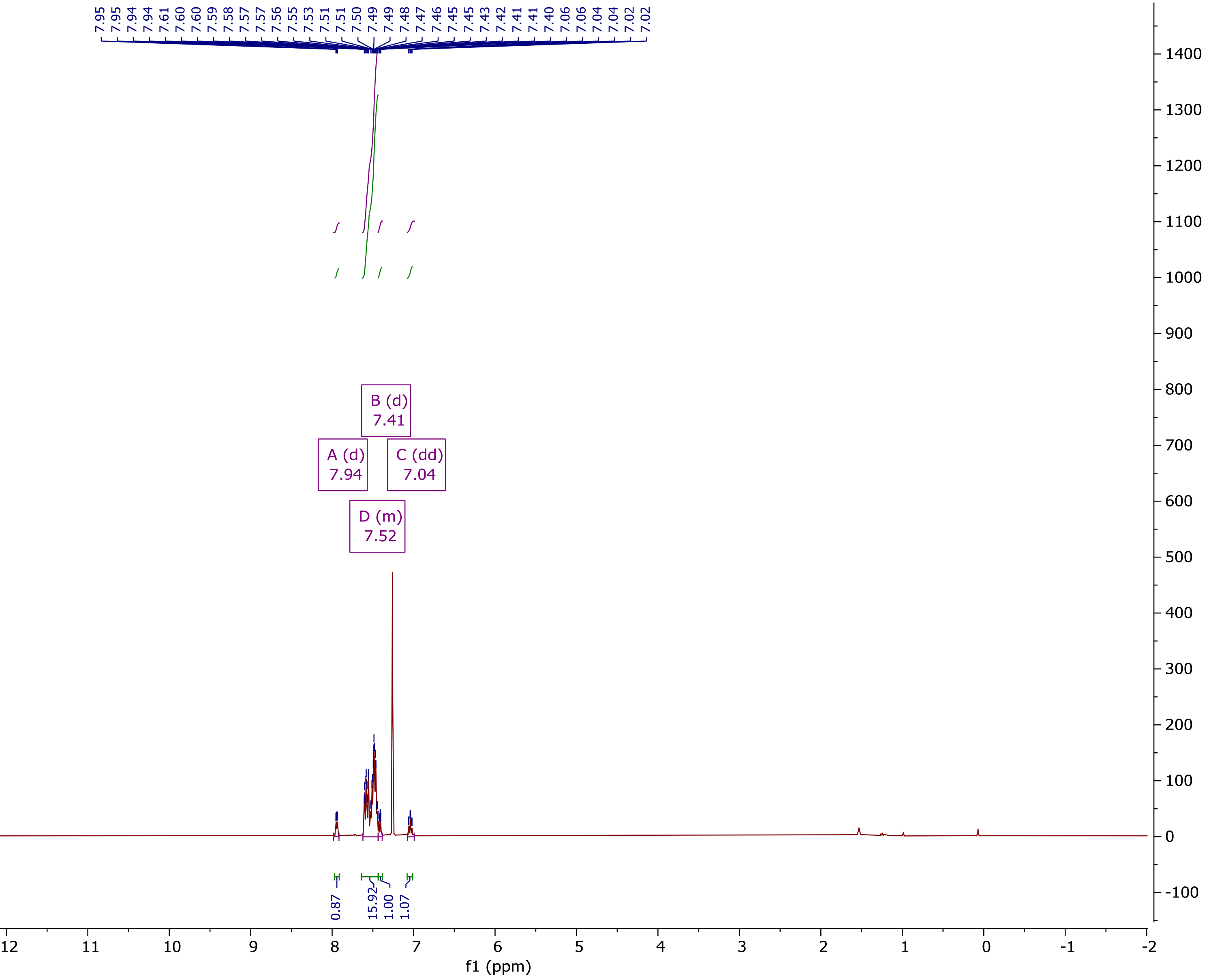
7.95 7.95 7.94 7.94 7.61 7.60 7.59 7.58 7.57 7.57 7.56 7.55 7.53 7.51 7.51 7.50 7.49 7.49 7.49 7.48 7.47 7.46 7.45 7.45 7.43 7.42 7.41 7.41 7.40 7.06 7.06 7.04 7.04 7.02 7.02
B (d)
7.41
A (d)
7.94
C (dd)
7.04
D (m)
7.52
0.87
15.92
1.00
1.07
f1 (ppm)

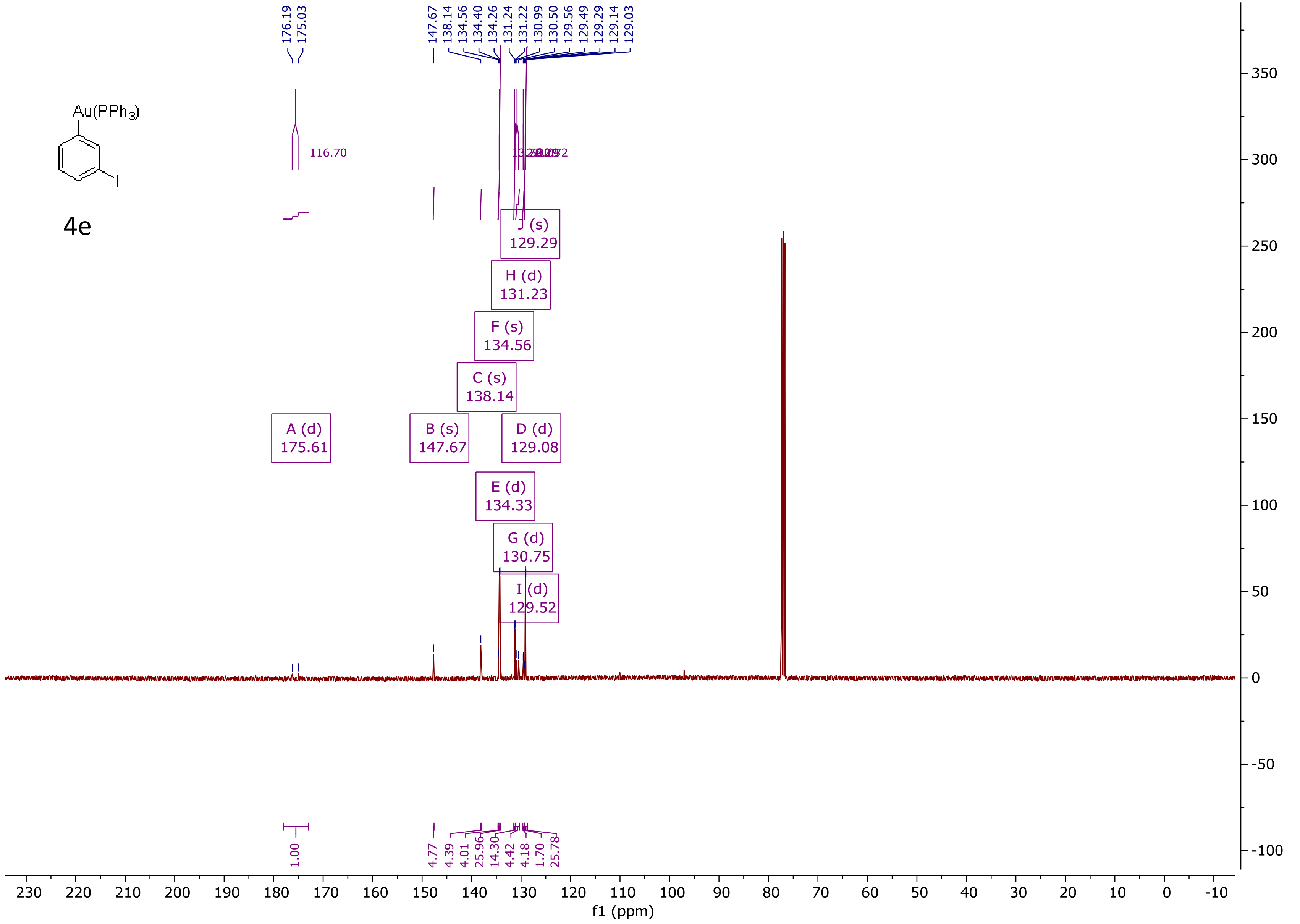
176.19
175.03
147.67
138.14
134.56
134.40
134.26
131.24
131.22
130.99
130.50
129.56
129.49
129.29
129.14
129.03
Au(PPh3)
I
4e
116.70
J (s) 129.29
H (d) 131.23
F (s) 134.56
C (s) 138.14
A (d) 175.61
B (s) 147.67
D (d) 129.08
E (d) 134.33
G (d) 130.75
I (d) 129.52
1.00
4.77
4.39
4.01
25.96
14.30
4.42
4.18
1.70
25.78
f1 (ppm)

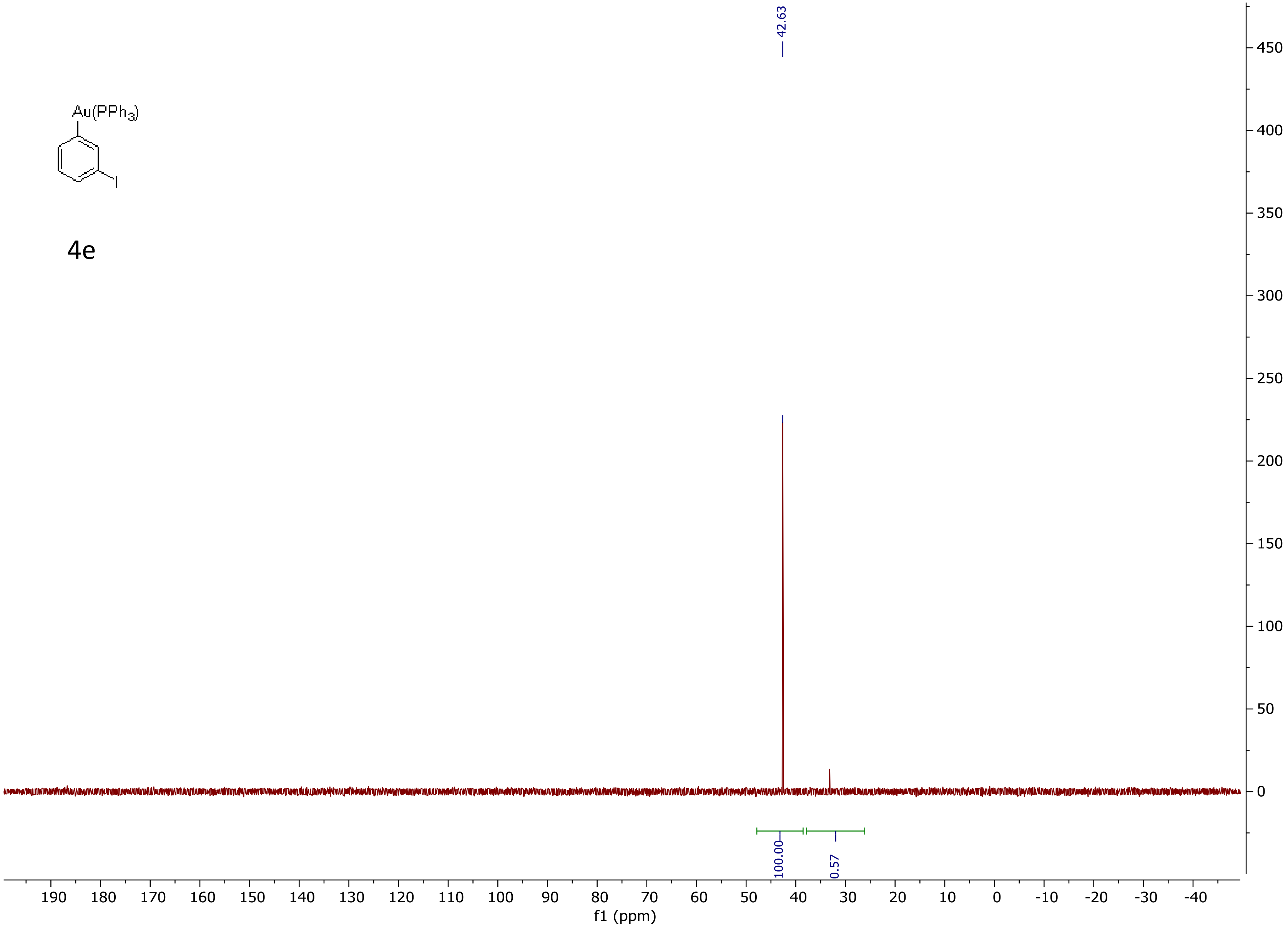

4e

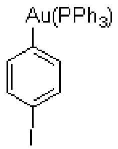

4g

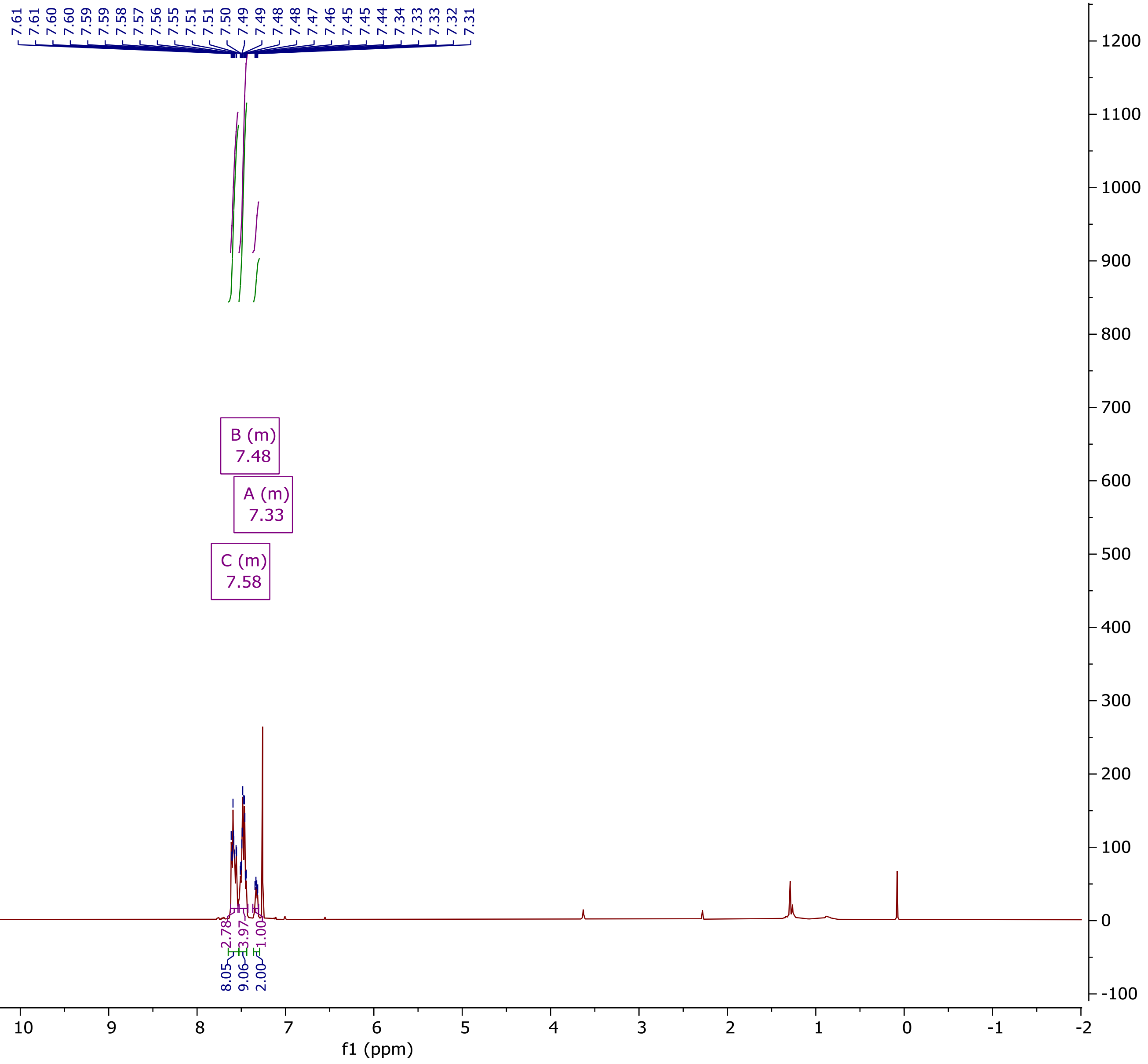

7.61
7.61
7.60
7.60
7.59
7.59
7.58
7.57
7.56
7.55
7.51
7.51
7.50
7.49
7.49
7.48
7.48
7.47
7.46
7.45
7.45
7.44
7.34
7.33
7.33
7.32
7.31
B (m)
7.48
A (m)
7.33
C (m)
7.58
8.05
2.78
9.06
3.97
2.00
1.00
f1 (ppm)

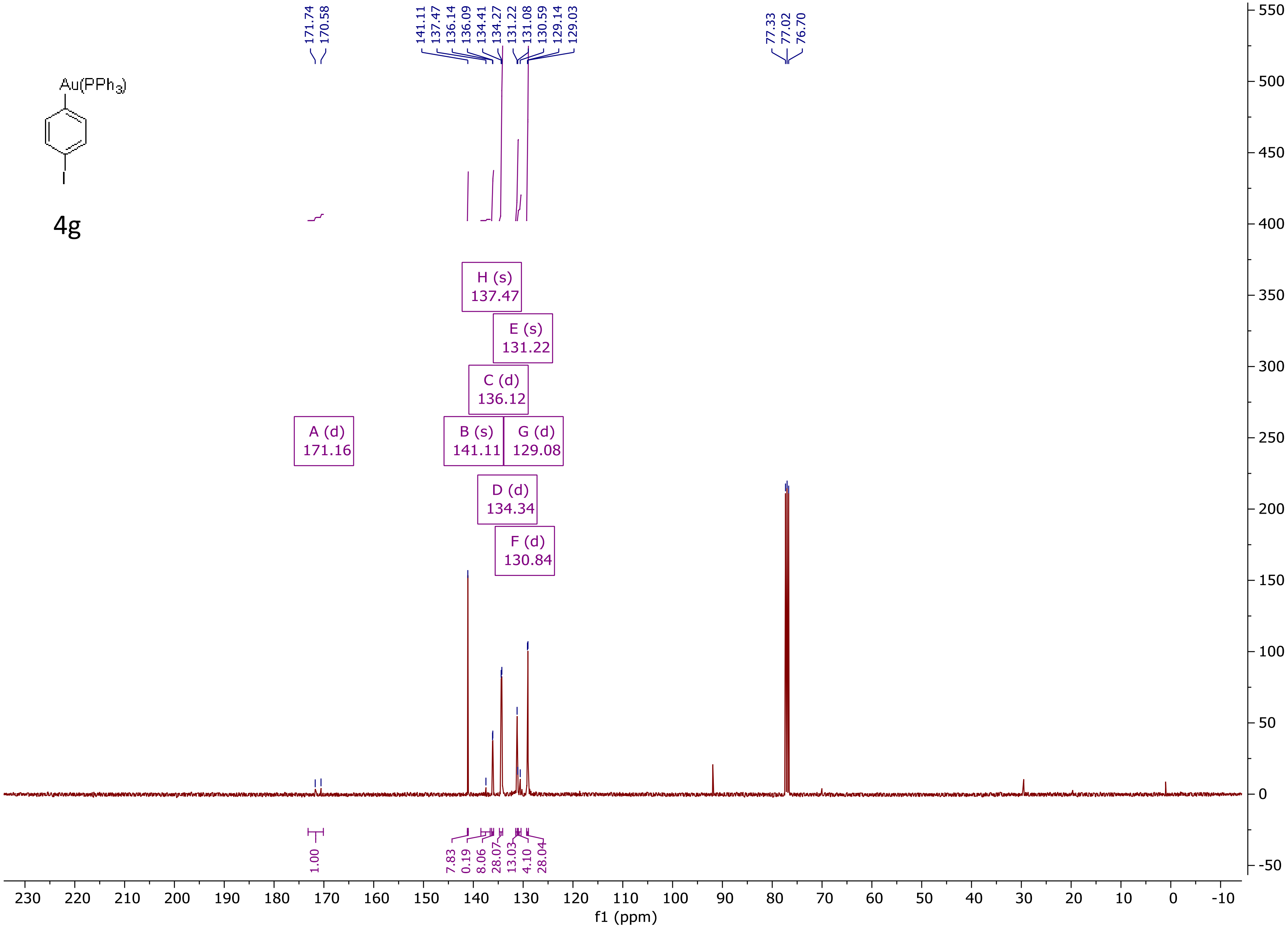
Au(PPh3)
I
4g
171.74
170.58
141.11
137.47
136.14
136.09
134.41
134.27
131.22
131.08
130.59
129.14
129.03
77.33
77.02
76.70
H (s)
137.47
E (s)
131.22
C (d)
136.12
A (d)
171.16
B (s)
141.11
G (d)
129.08
D (d)
134.34
F (d)
130.84
1.00
7.83
0.19
8.06
28.07
13.03
4.10
28.04
f1 (ppm)

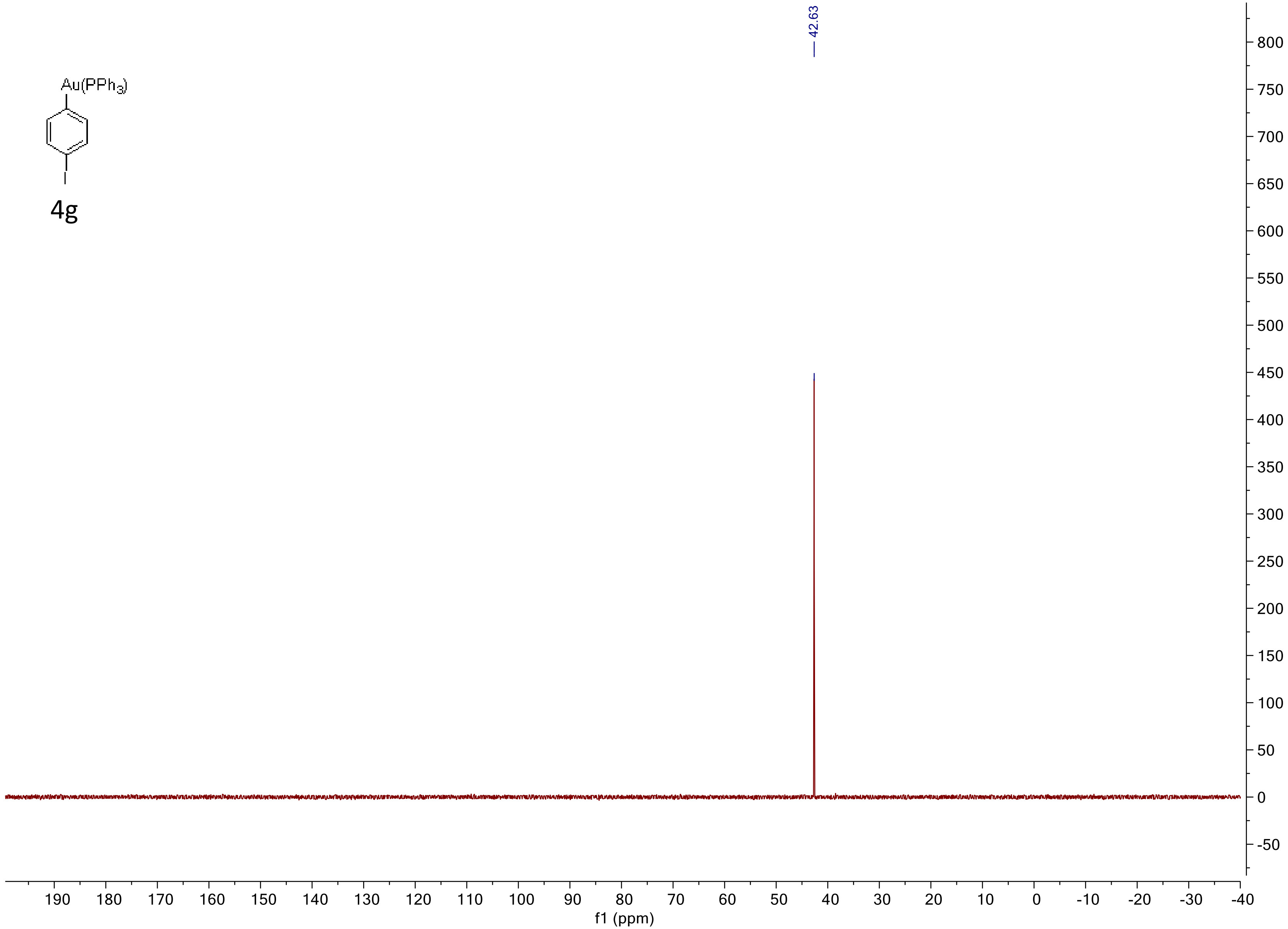
Au(PPh3)
I
4g
42.63
800
750
700
650
600
550
500
450
400
350
300
250
200
150
100
50
0
-50
190
180
170
160
150
140
130
120
110
100
90
80
70
60
50
40
30
20
10
0
-10
-20
-30
-40
f1 (ppm)

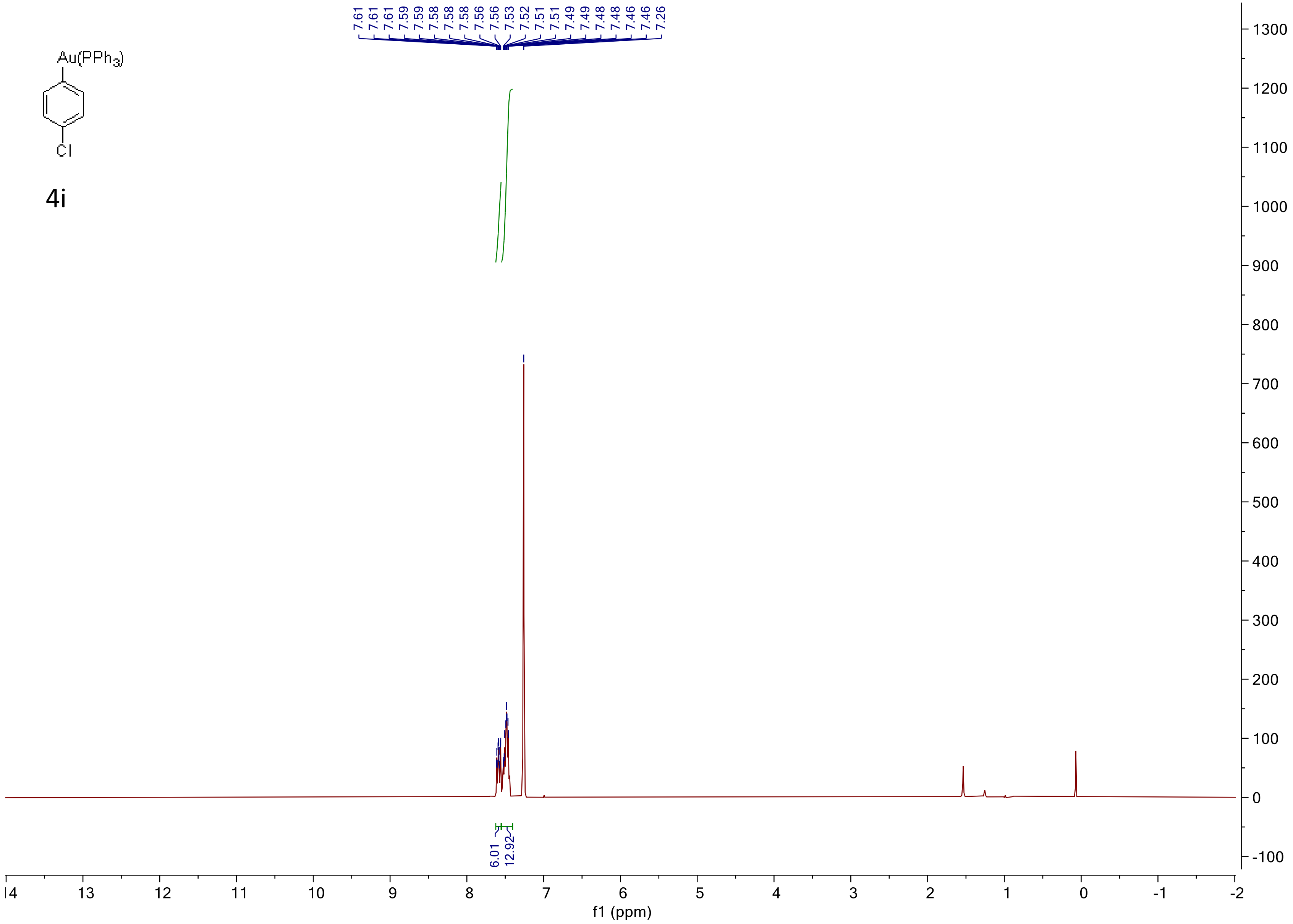
Au(PPh3)
Cl
7.61
7.61
7.61
7.59
7.59
7.58
7.58
7.58
7.56
7.56
7.53
7.52
7.51
7.51
7.49
7.48
7.48
7.46
7.46
7.26
6.01
12.92
f1 (ppm)

4i

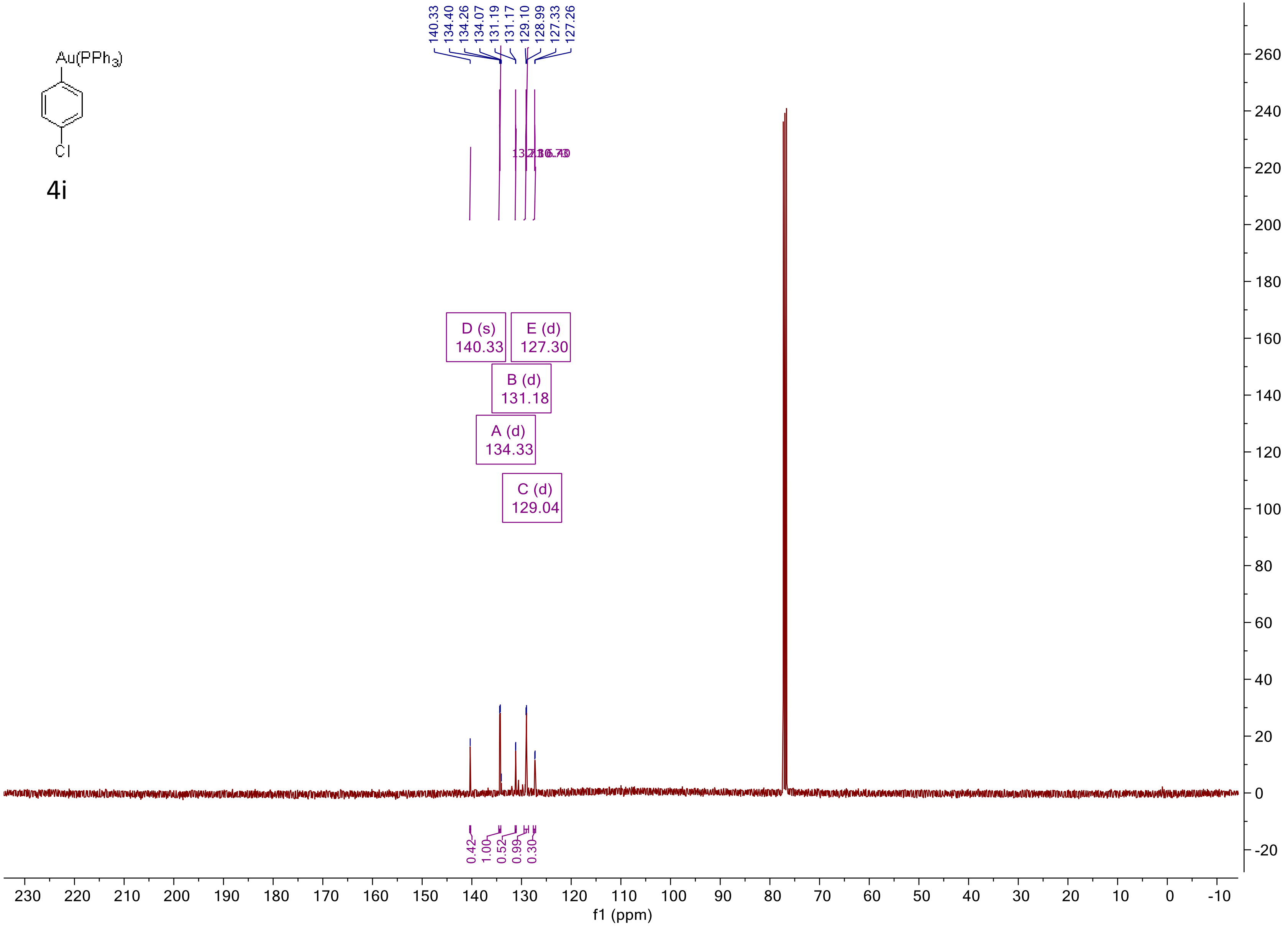
Au(PPh3)
Cl
4i
140.33
134.40
134.26
134.07
131.19
131.17
129.10
128.99
127.33
127.26
D (s)
140.33
E (d)
127.30
B (d)
131.18
A (d)
134.33
C (d)
129.04
0.42
1.00
0.52
0.99
0.30
f1 (ppm)

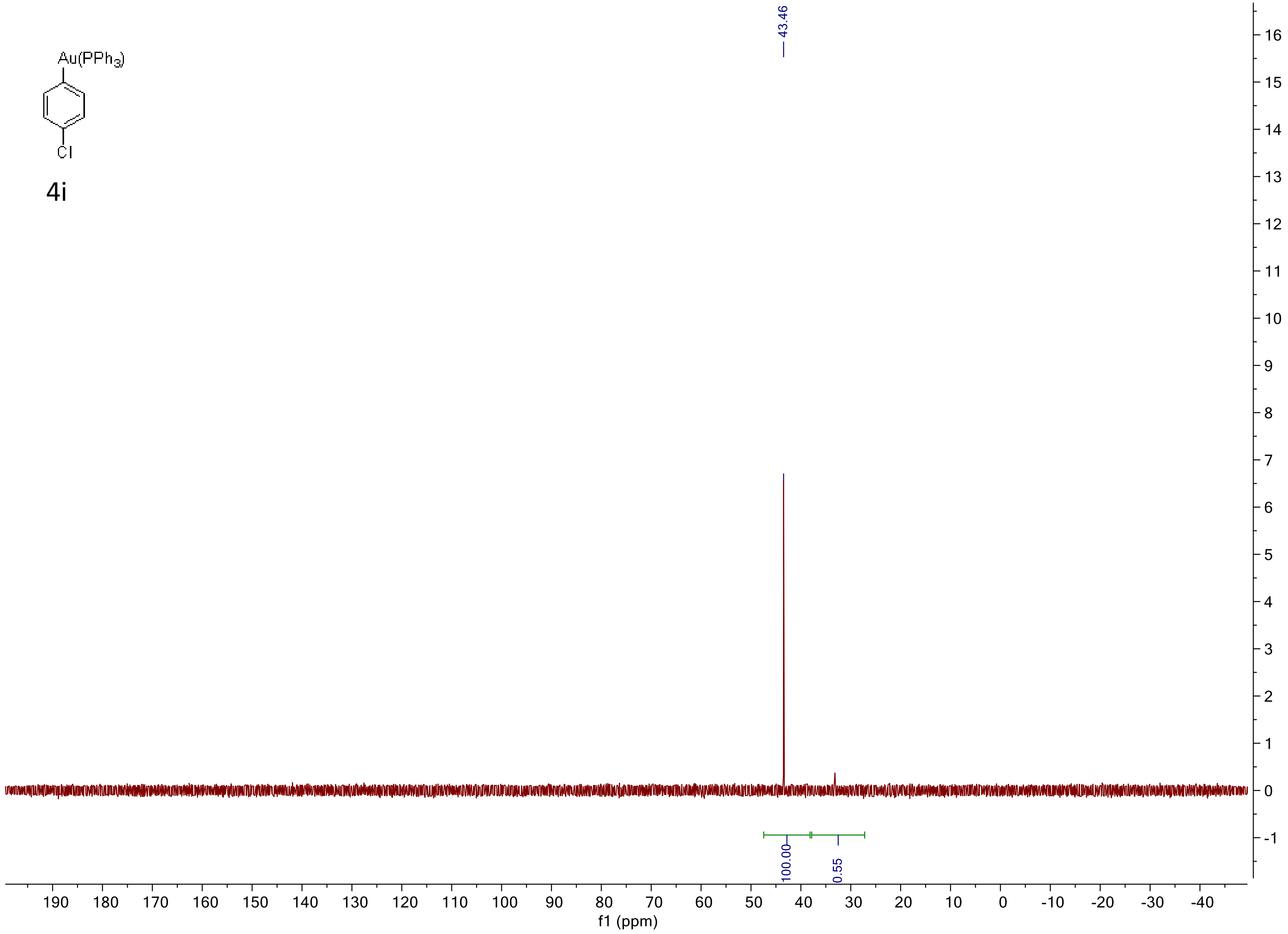

Au(PPh3)
Cl
4i
43.46
100.00
0.55
f1 (ppm)

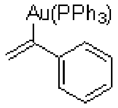
Au(PPh3)

4m

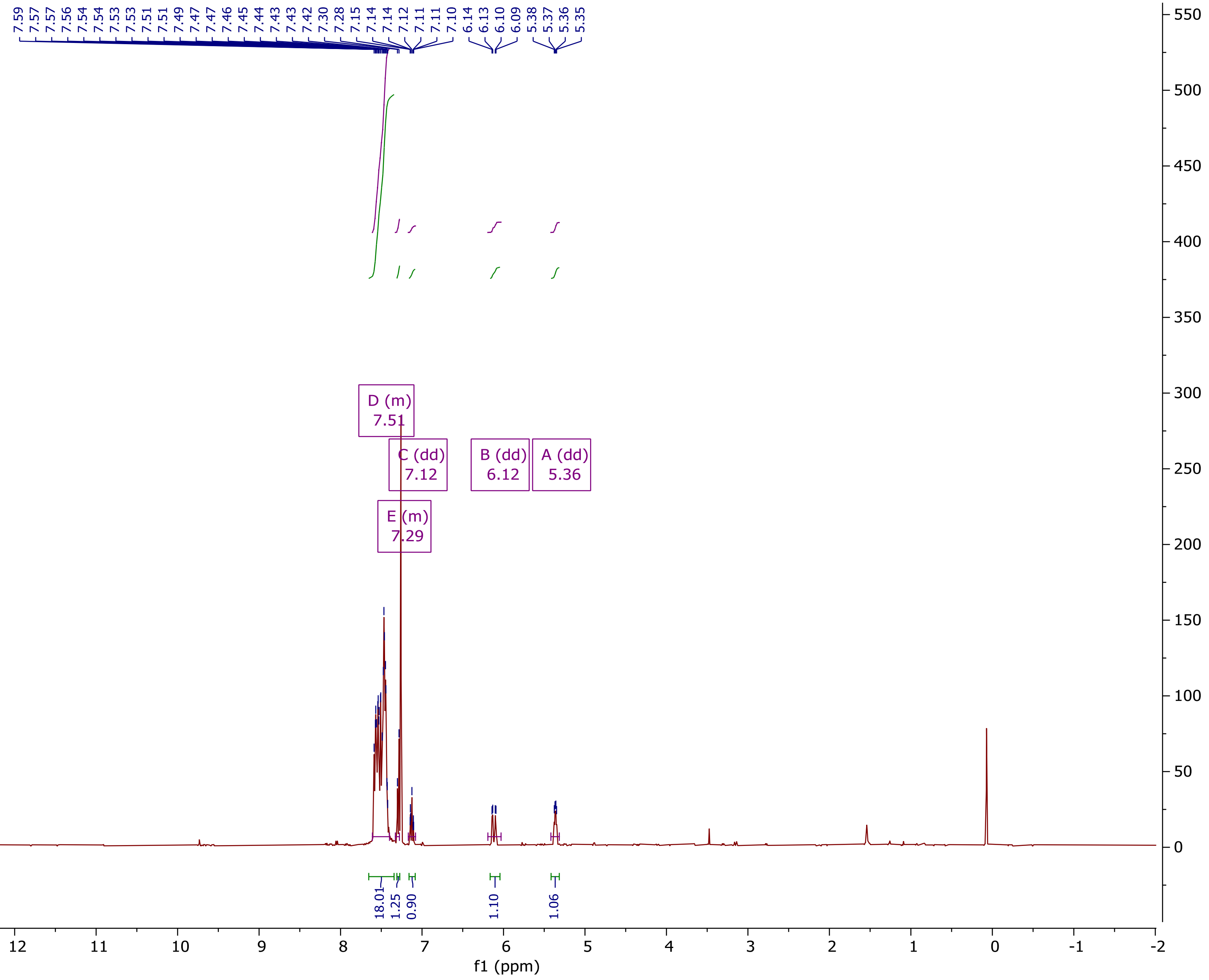
7.59 7.57 7.57 7.56 7.54 7.54 7.53 7.53 7.51 7.51 7.49 7.47 7.47 7.46 7.45 7.44 7.43 7.43 7.42 7.30 7.28 7.15 7.14 7.14 7.12 7.11 7.11 7.10 6.14 6.13 6.10 6.09 5.38 5.37 5.36 5.35
D (m)
7.51
C (dd)
7.12
B (dd)
6.12
A (dd)
5.36
E (m)
7.29
18.01
1.25
0.90
1.10
1.06
f1 (ppm)

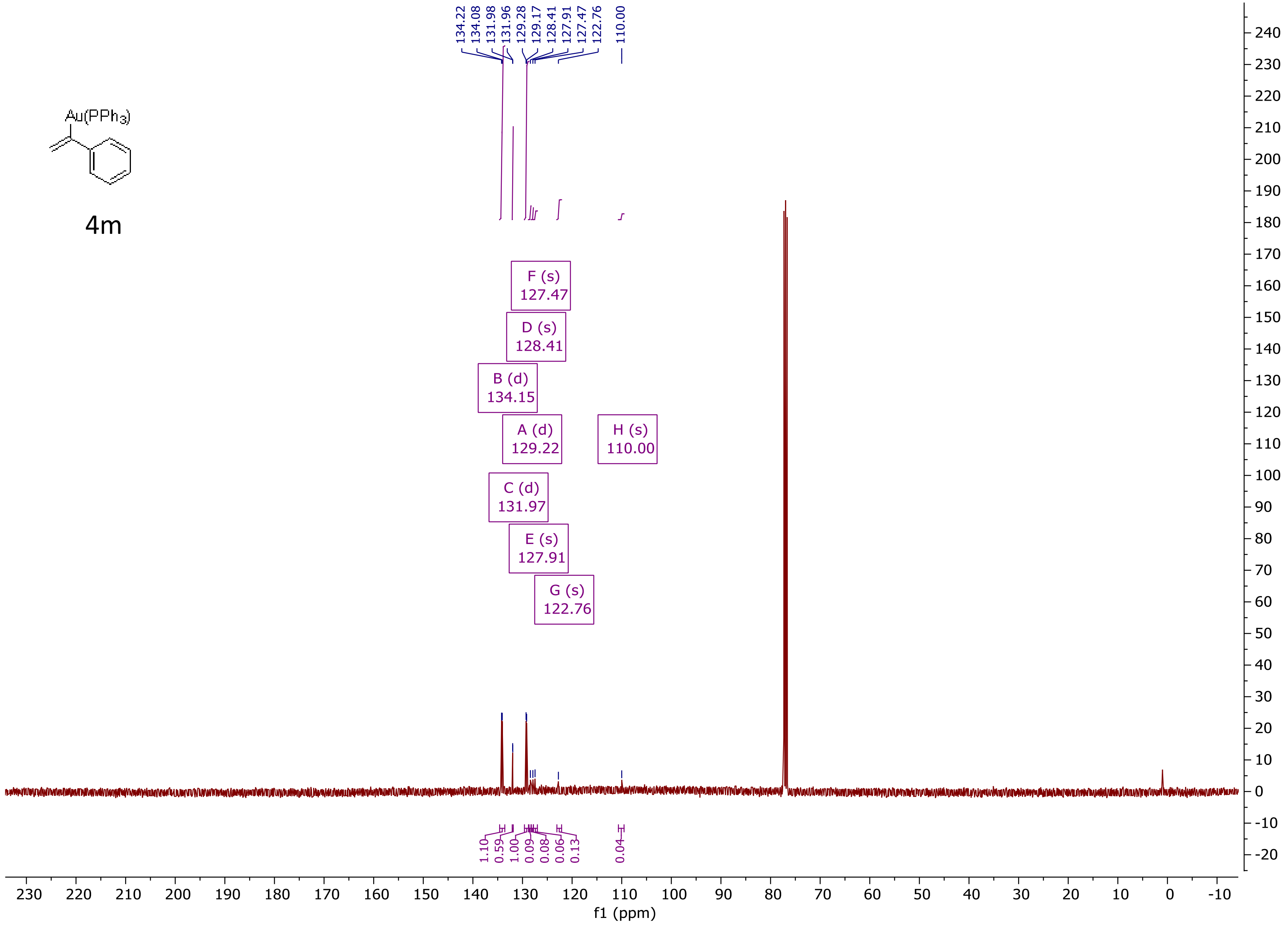
Au(PPh3)
4m
134.22
134.08
131.98
131.96
129.28
129.17
128.41
127.91
127.47
122.76
110.00
F (s) 127.47
D (s) 128.41
B (d) 134.15
A (d) 129.22
H (s) 110.00
C (d) 131.97
E (s) 127.91
G (s) 122.76
1.10
0.59
1.00
0.09
0.08
0.06
0.13
0.04
f1 (ppm)

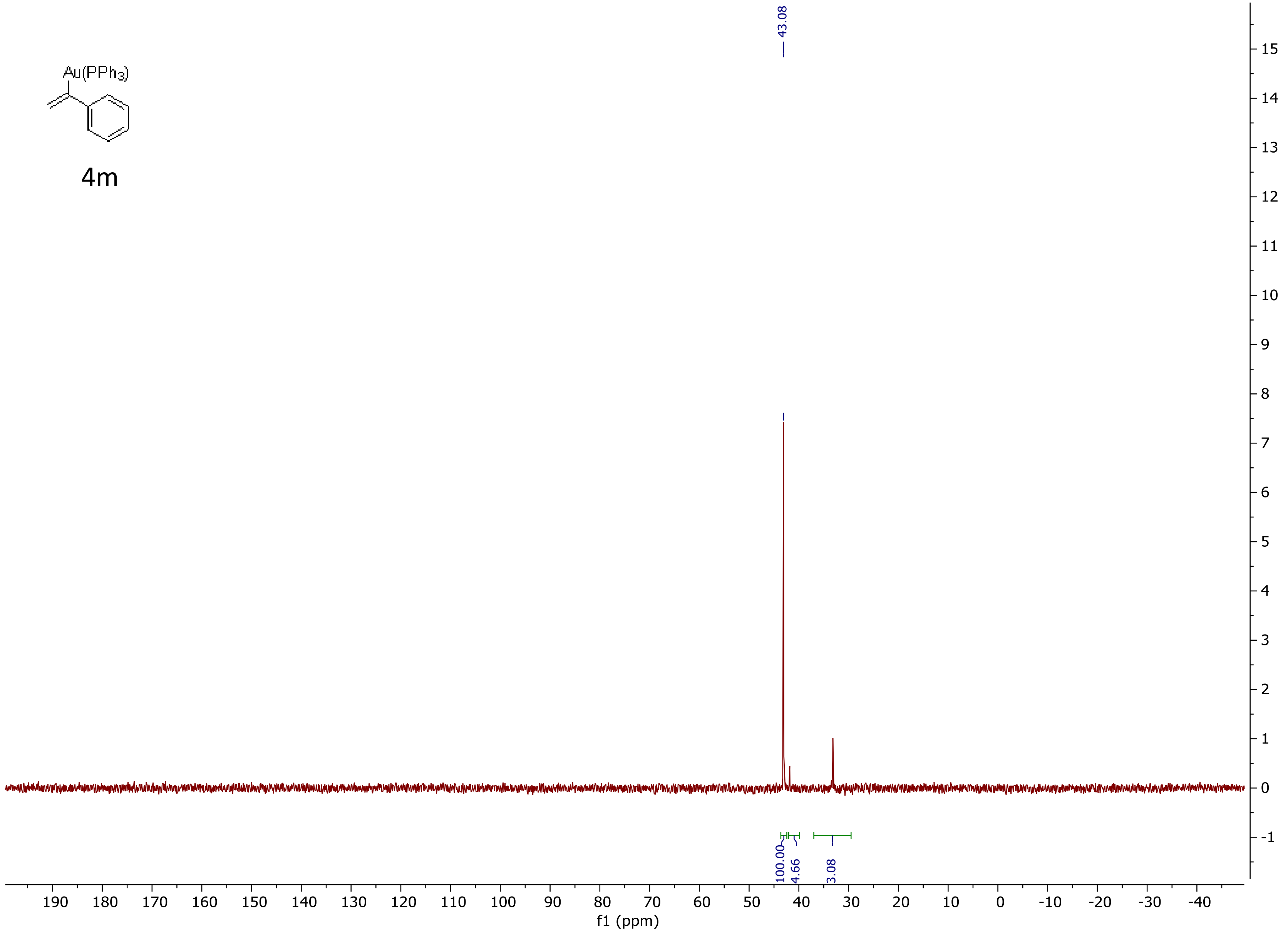

4m

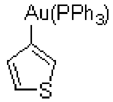

4n

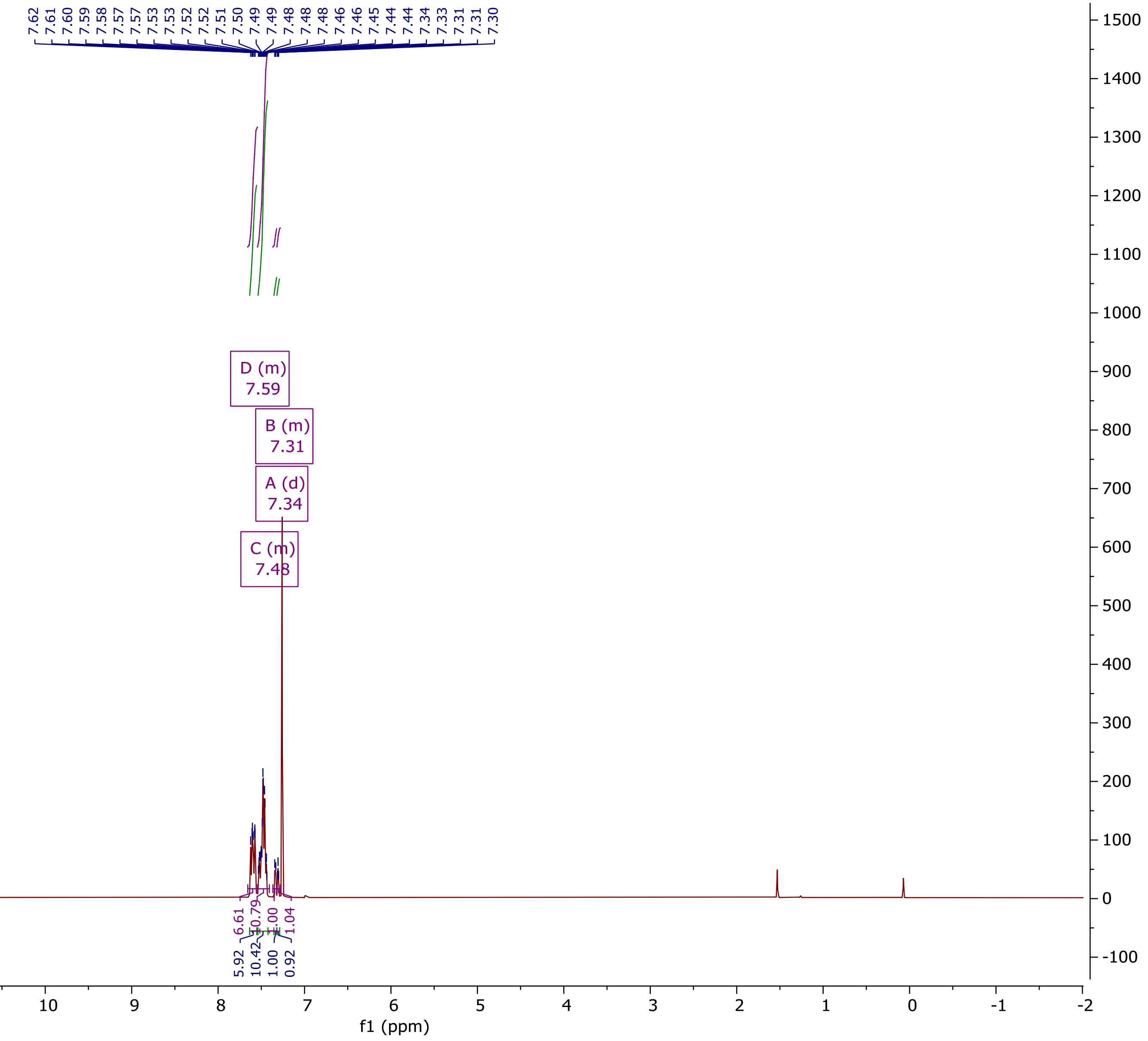

7.62 7.61 7.60 7.59 7.58 7.57 7.57 7.53 7.53 7.52 7.52 7.51 7.50 7.49 7.49 7.49 7.48 7.48 7.48 7.46 7.46 7.45 7.44 7.44 7.34 7.33 7.31 7.31 7.30
D (m) 7.59
B (m) 7.31
A (d) 7.34
C (m) 7.48
6.61 10.79 1.00 1.04
5.92 10.42 1.00 0.92
f1 (ppm)

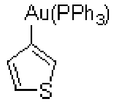
Au(PPh3)
S

4n

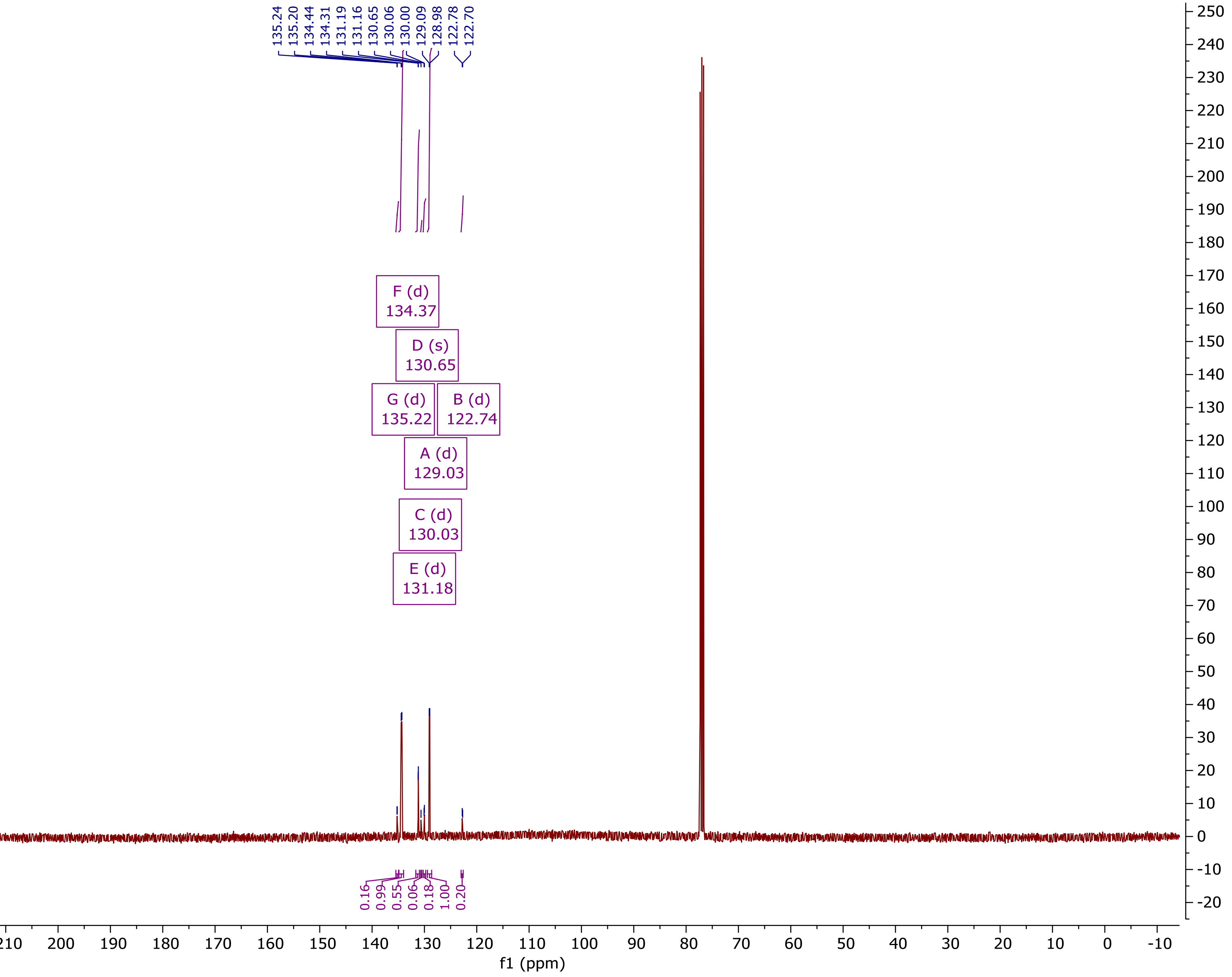
135.24
135.20
134.44
134.31
131.19
131.16
130.65
130.06
130.00
129.09
128.98
122.78
122.70
F (d)
134.37
D (s)
130.65
G (d)
135.22
B (d)
122.74
A (d)
129.03
C (d)
130.03
E (d)
131.18
0.16
0.99
0.55
0.06
0.18
1.00
0.20
f1 (ppm)

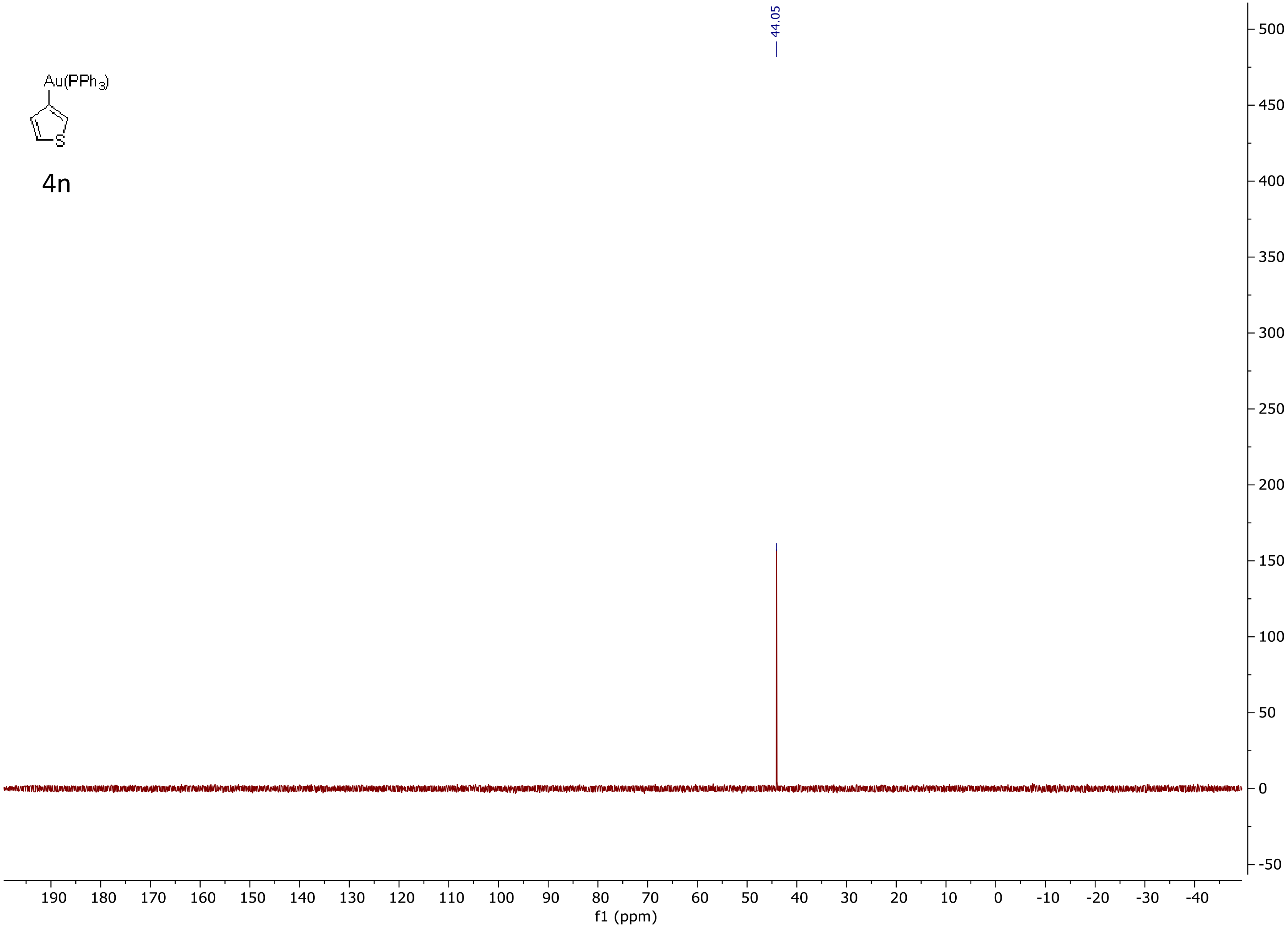

4n

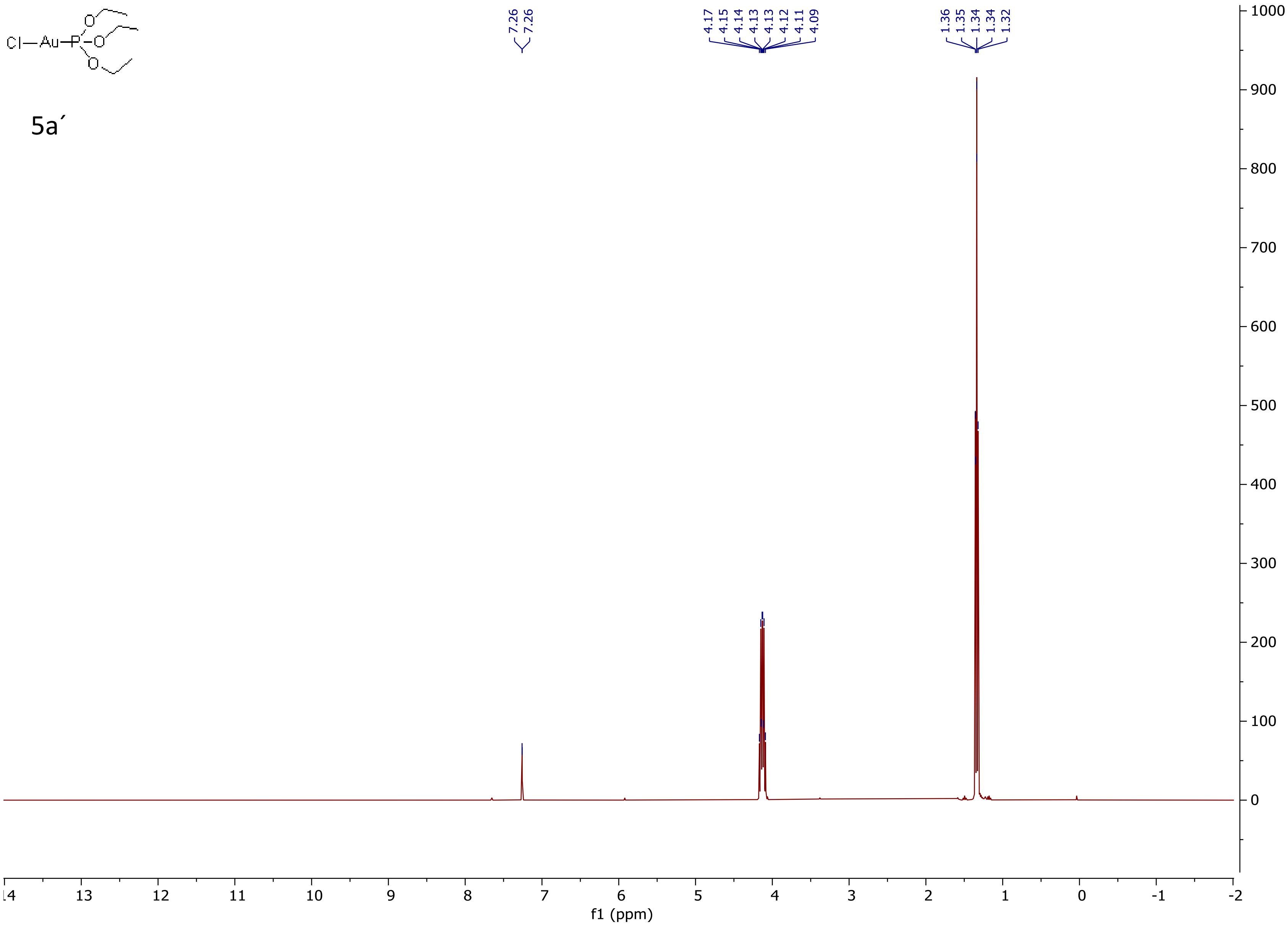
Cl—Au—P—O
5a´
7.26
7.26
4.17
4.15
4.14
4.13
4.13
4.12
4.11
4.09
1.36
1.35
1.34
1.34
1.32
1000
900
800
700
600
500
400
300
200
100
0
14
13
12
11
10
9
8
7
6
5
4
3
2
1
0
-1
-2
f1 (ppm)

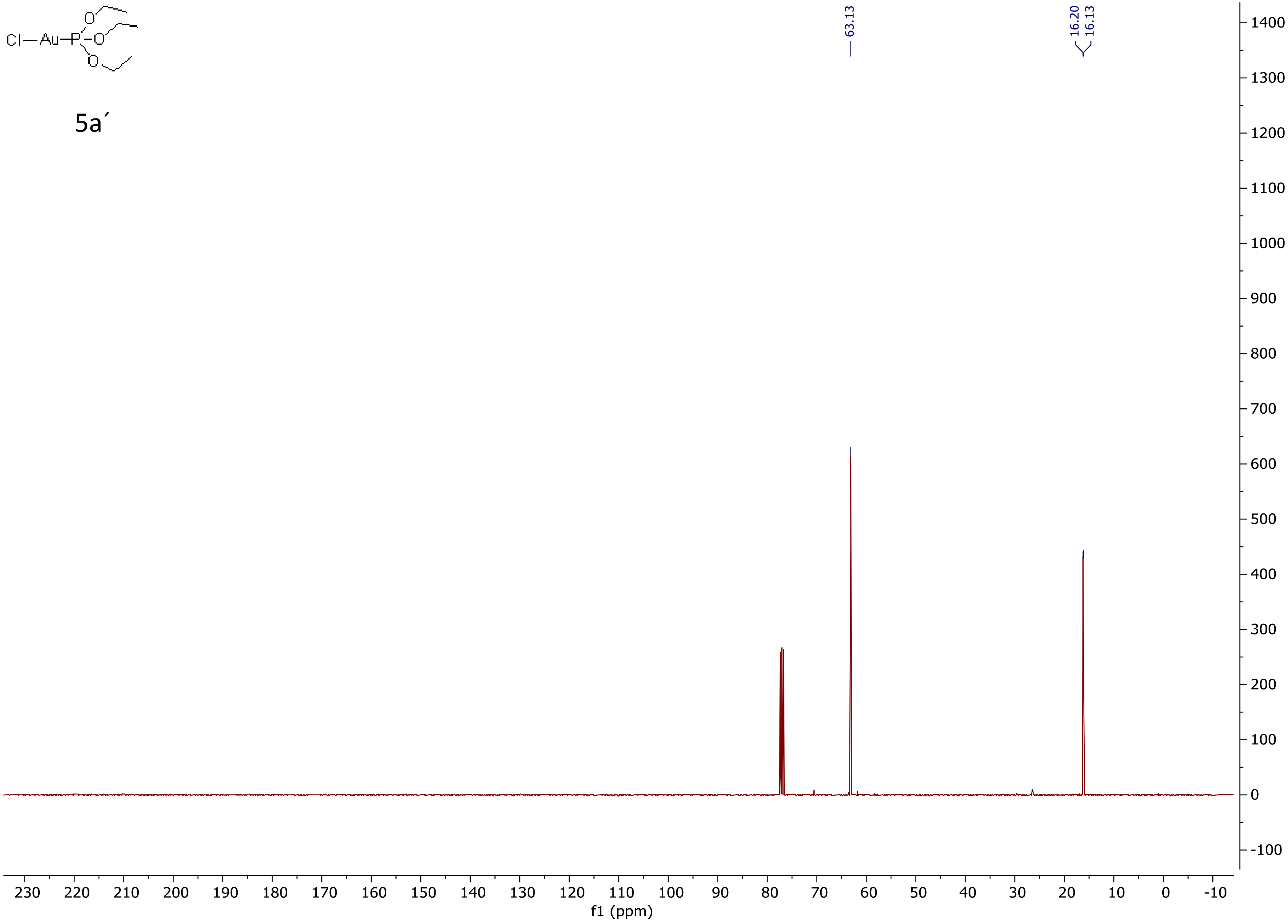
Cl—Au—P—O
O
O
63.13
16.20
16.13
1400
1300
1200
1100
1000
900
800
700
600
500
400
300
200
100
0
-100
230 220 210 200 190 180 170 160 150 140 130 120 110 100 90 80 70 60 50 40 30 20 10 0 -10
f1 (ppm)

5a´

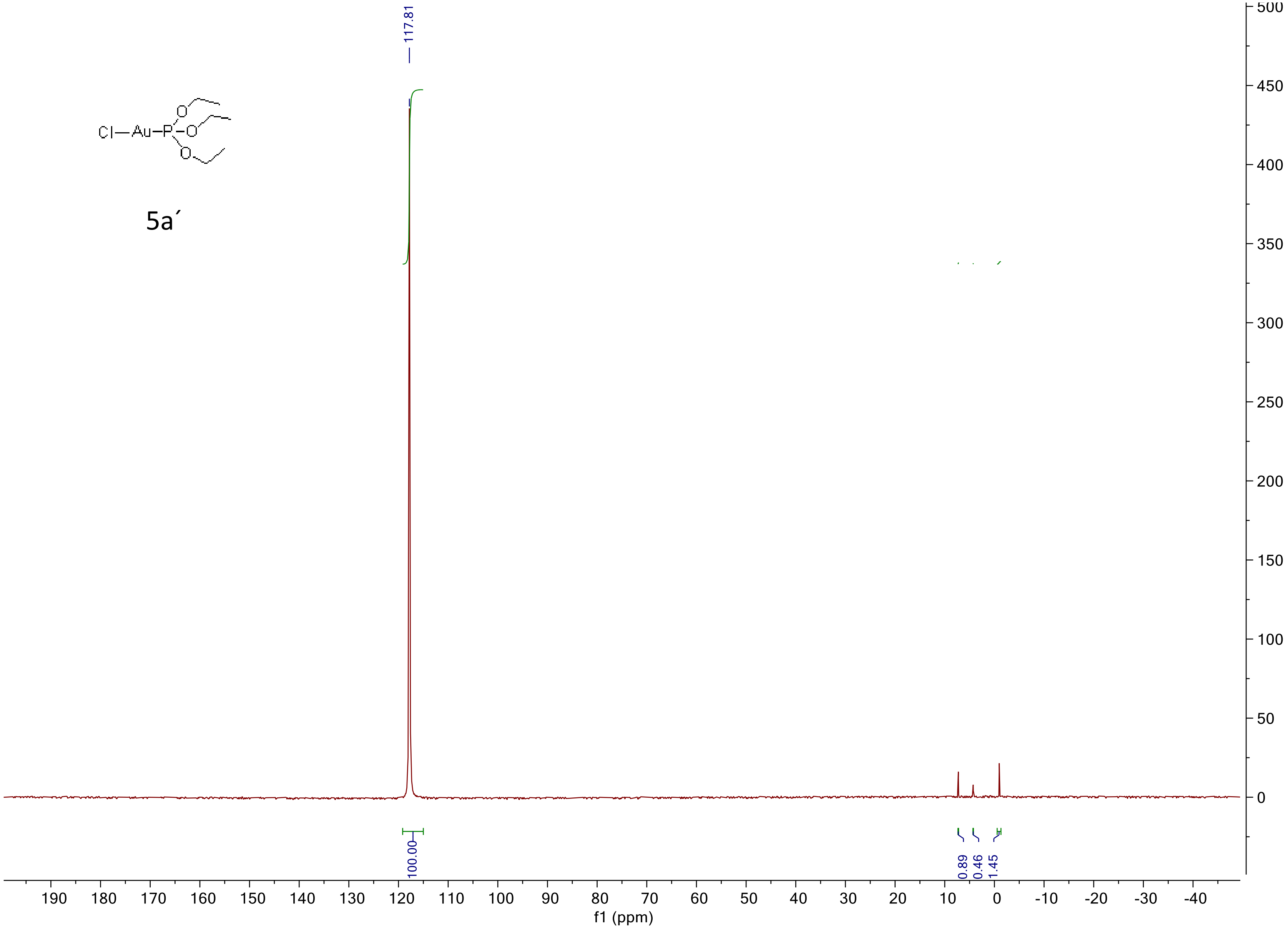
Cl—Au—P
O
O
O
5a´
117.81
100.00
0.89
0.46
1.45
500
450
400
350
300
250
200
150
100
50
0
190 180 170 160 150 140 130 120 110 100 90 80 70 60 50 40 30 20 10 0 -10 -20 -30 -40
f1 (ppm)

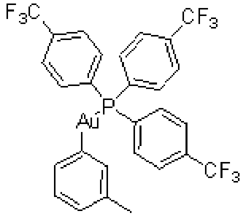

F3C
CF3
CF3
P
Au

5c

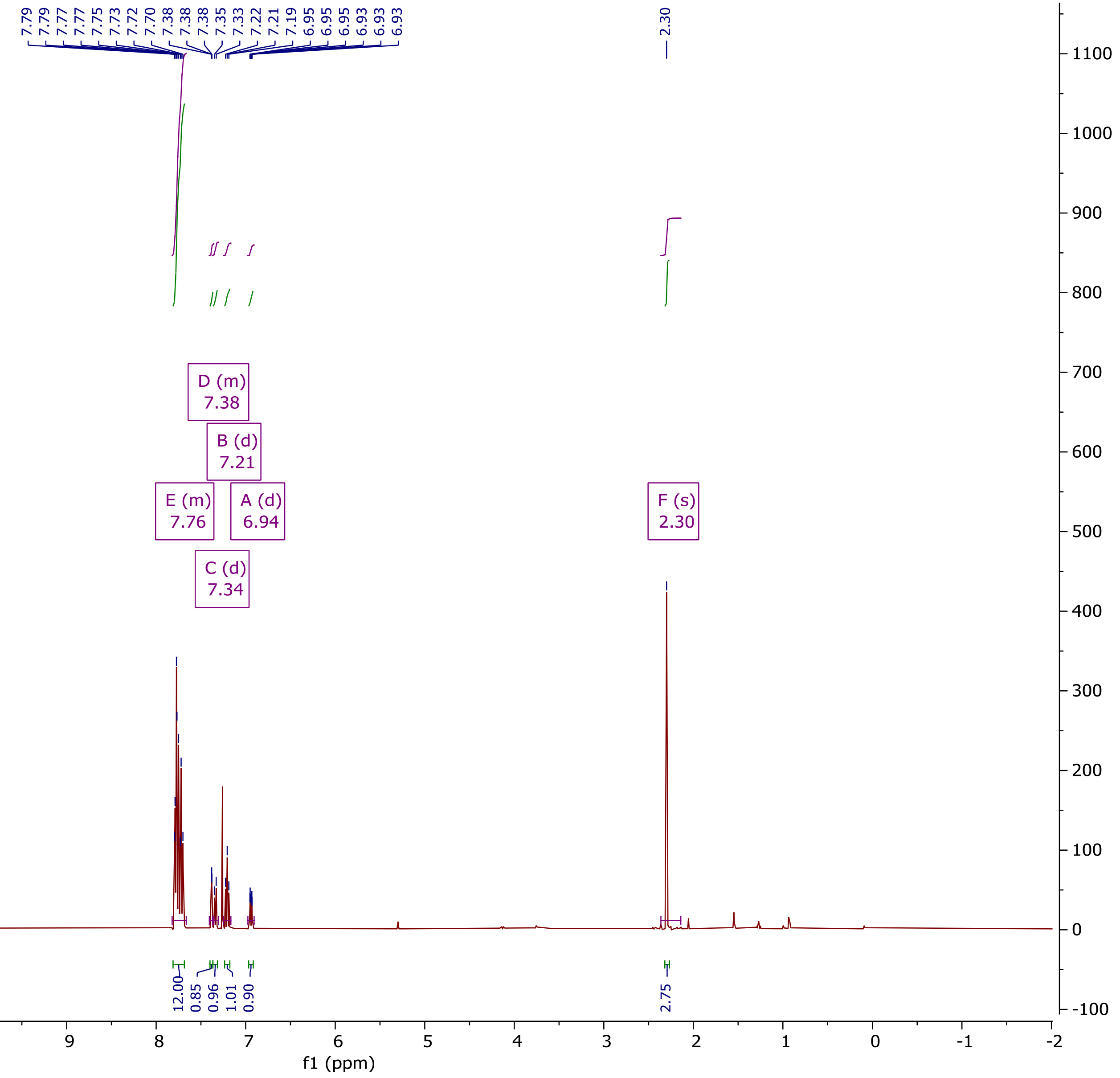

7.79
7.79
7.77
7.77
7.75
7.73
7.72
7.70
7.38
7.38
7.38
7.35
7.33
7.22
7.21
7.19
6.95
6.95
6.95
6.93
6.93
6.93
2.30
D (m)
7.38
B (d)
7.21
E (m)
7.76
A (d)
6.94
F (s)
2.30
C (d)
7.34
12.00
0.85
0.96
1.01
0.90
2.75
f1 (ppm)

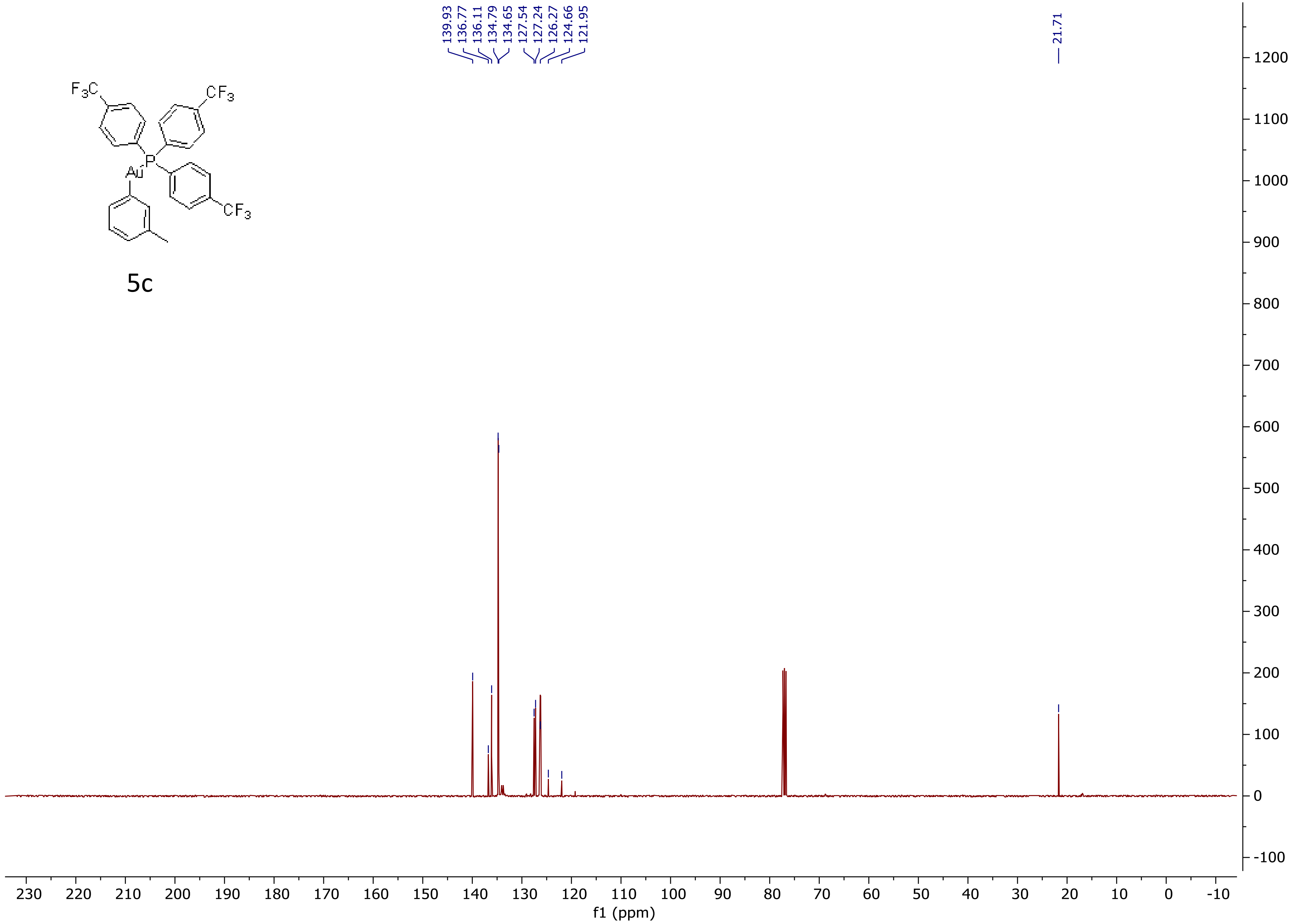
F3C
CF3
P
Au
CF3
139.93
136.77
136.11
134.79
134.65
127.54
127.24
126.27
124.66
121.95
21.71
f1 (ppm)

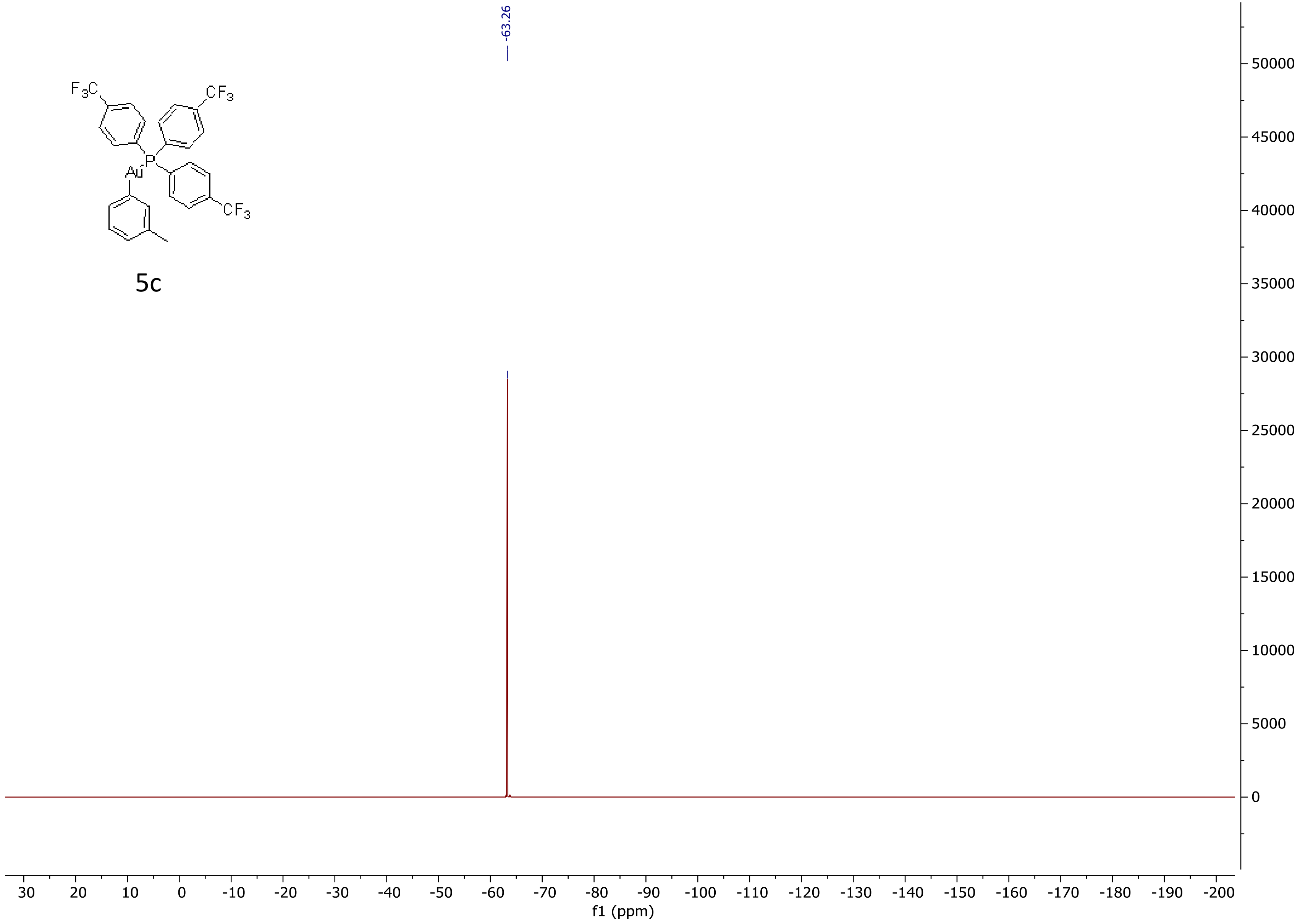
F3C
CF3
CF3
P
Au
5c
-63.26
50000
45000
40000
35000
30000
25000
20000
15000
10000
5000
0
30
20
10
0
-10
-20
-30
-40
-50
-60
-70
-80
-90
-100
-110
-120
-130
-140
-150
-160
-170
-180
-190
-200
f1 (ppm)

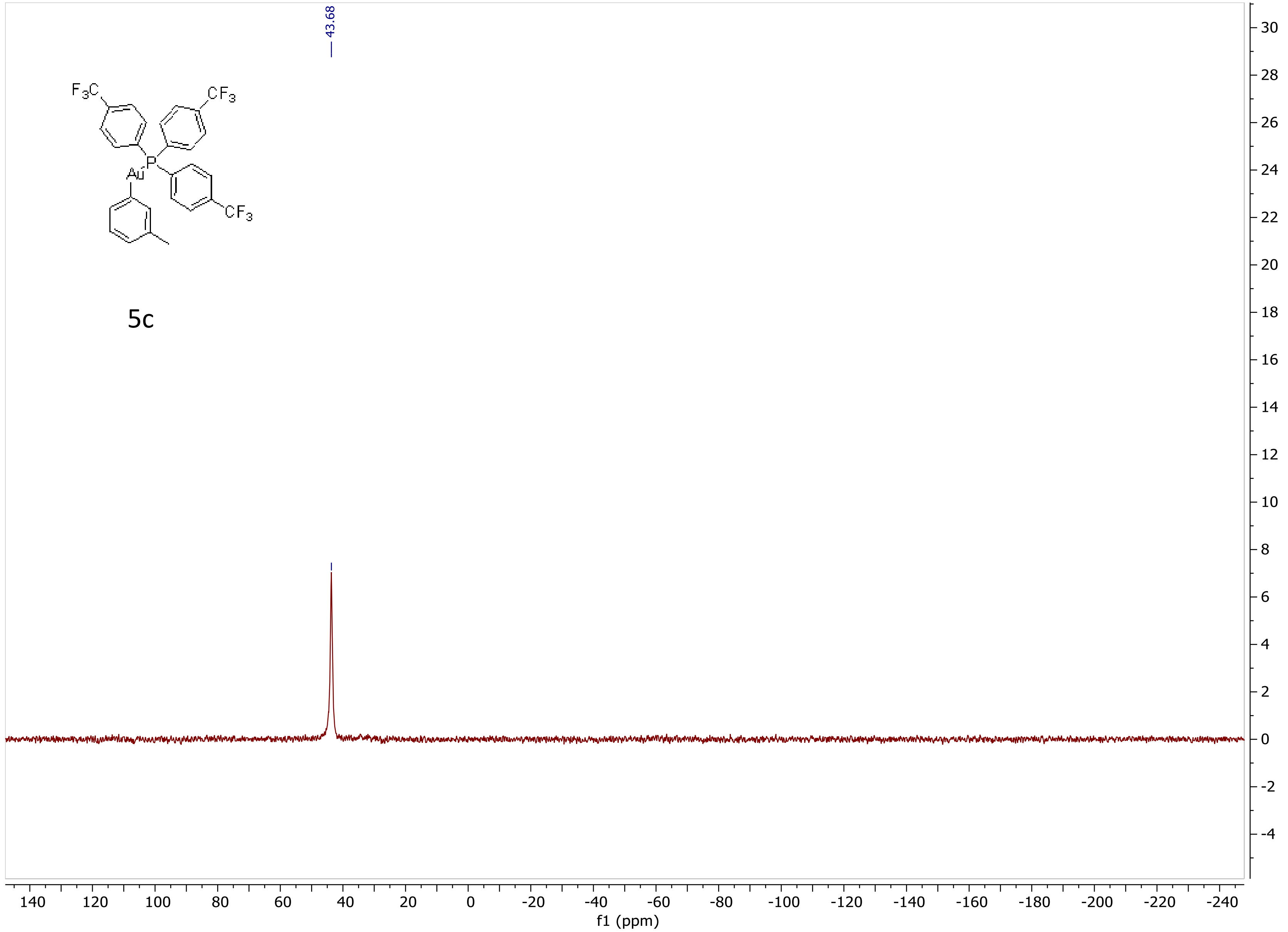
43.68
F3C
CF3
CF3
P
Au
5c
f1 (ppm)

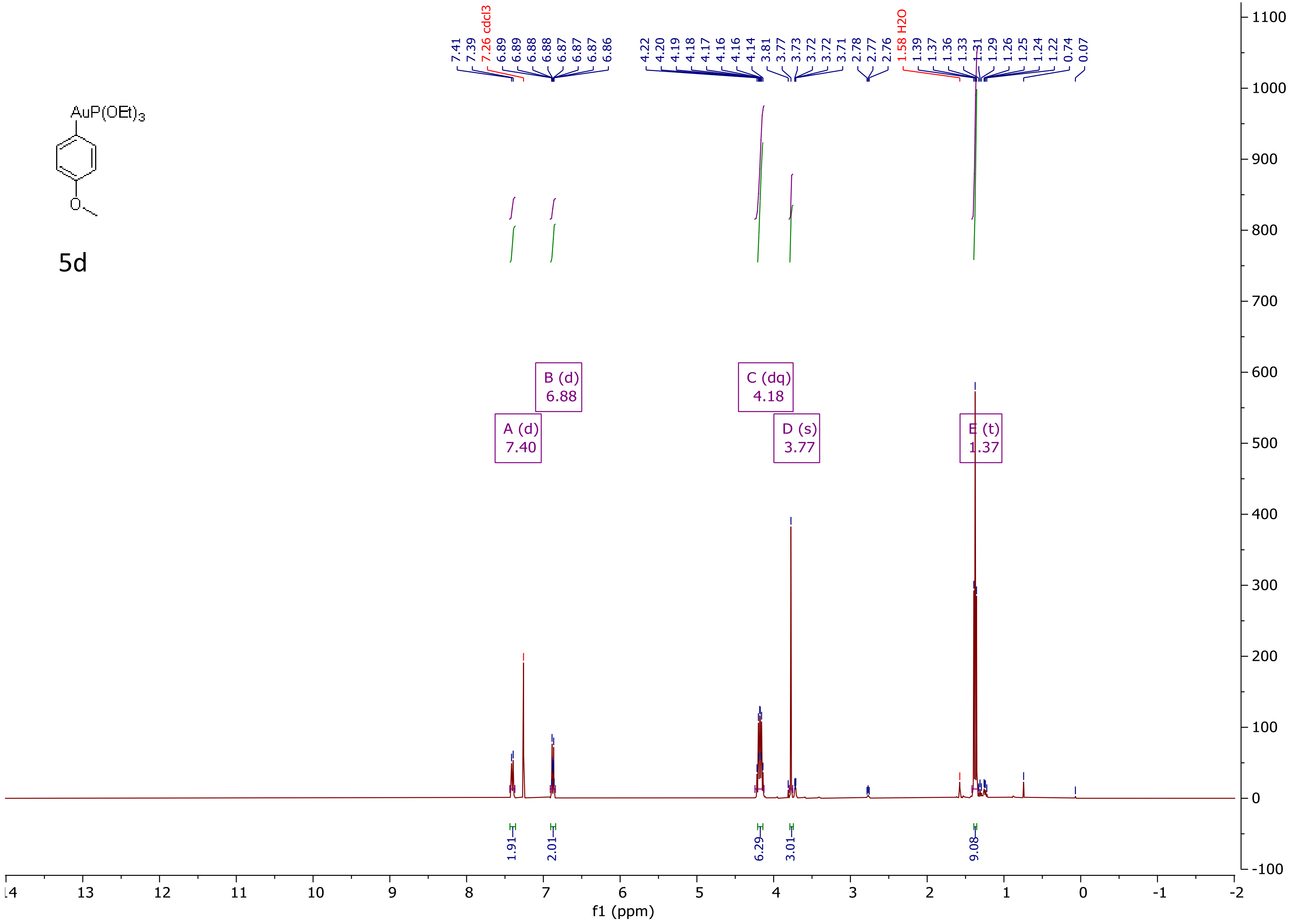

5d

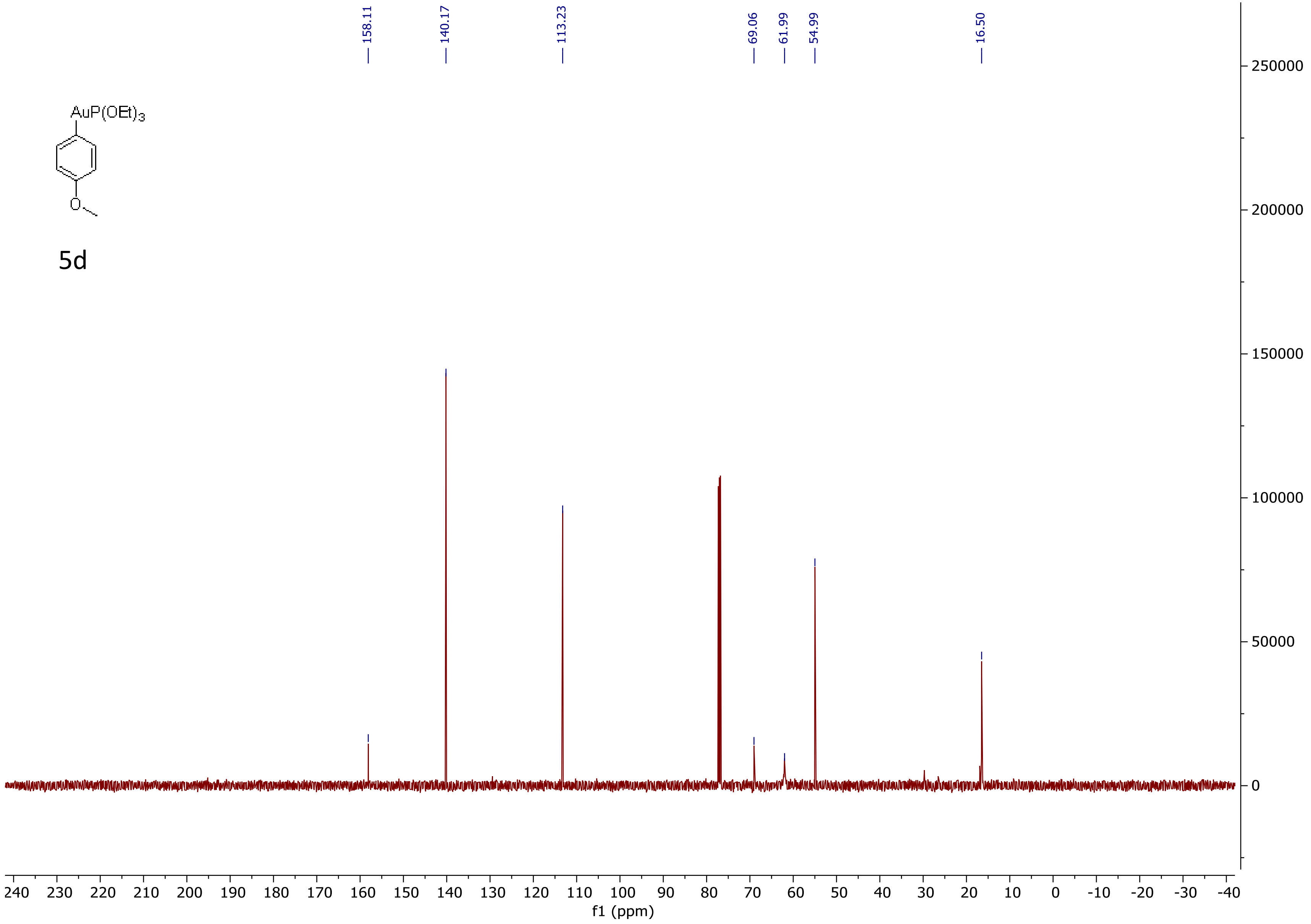
AuP(OEt)3
O
5d
158.11
140.17
113.23
69.06
61.99
54.99
16.50
250000
200000
150000
100000
50000
0
240 230 220 210 200 190 180 170 160 150 140 130 120 110 100 90 80 70 60 50 40 30 20 10 0 -10 -20 -30 -40
f1 (ppm)

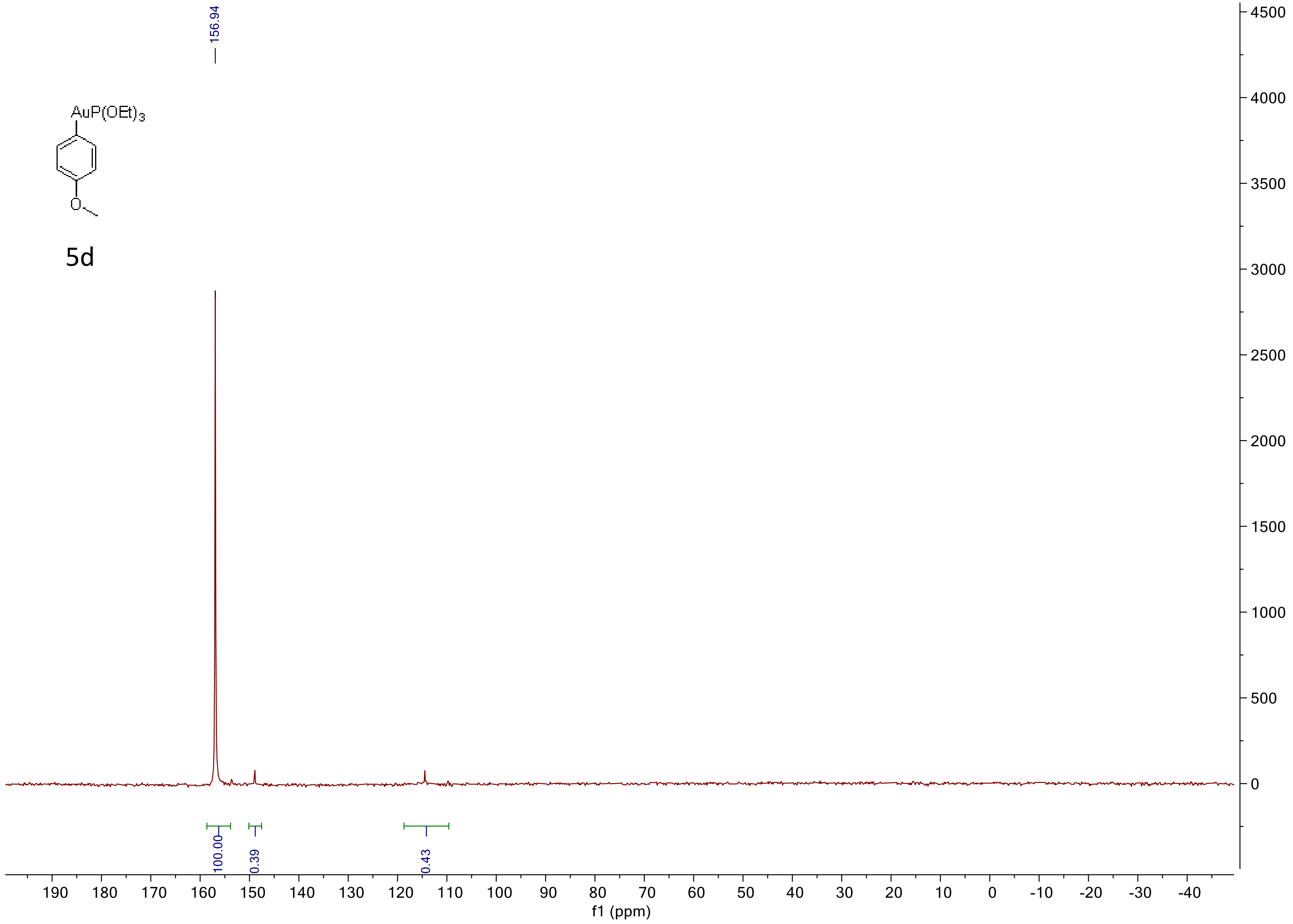
156.94
AuP(OEt)3
100.00
0.39
0.43
4500
4000
3500
3000
2500
2000
1500
1000
500
0
190 180 170 160 150 140 130 120 110 100 90 80 70 60 50 40 30 20 10 0 -10 -20 -30 -40
f1 (ppm)

5d

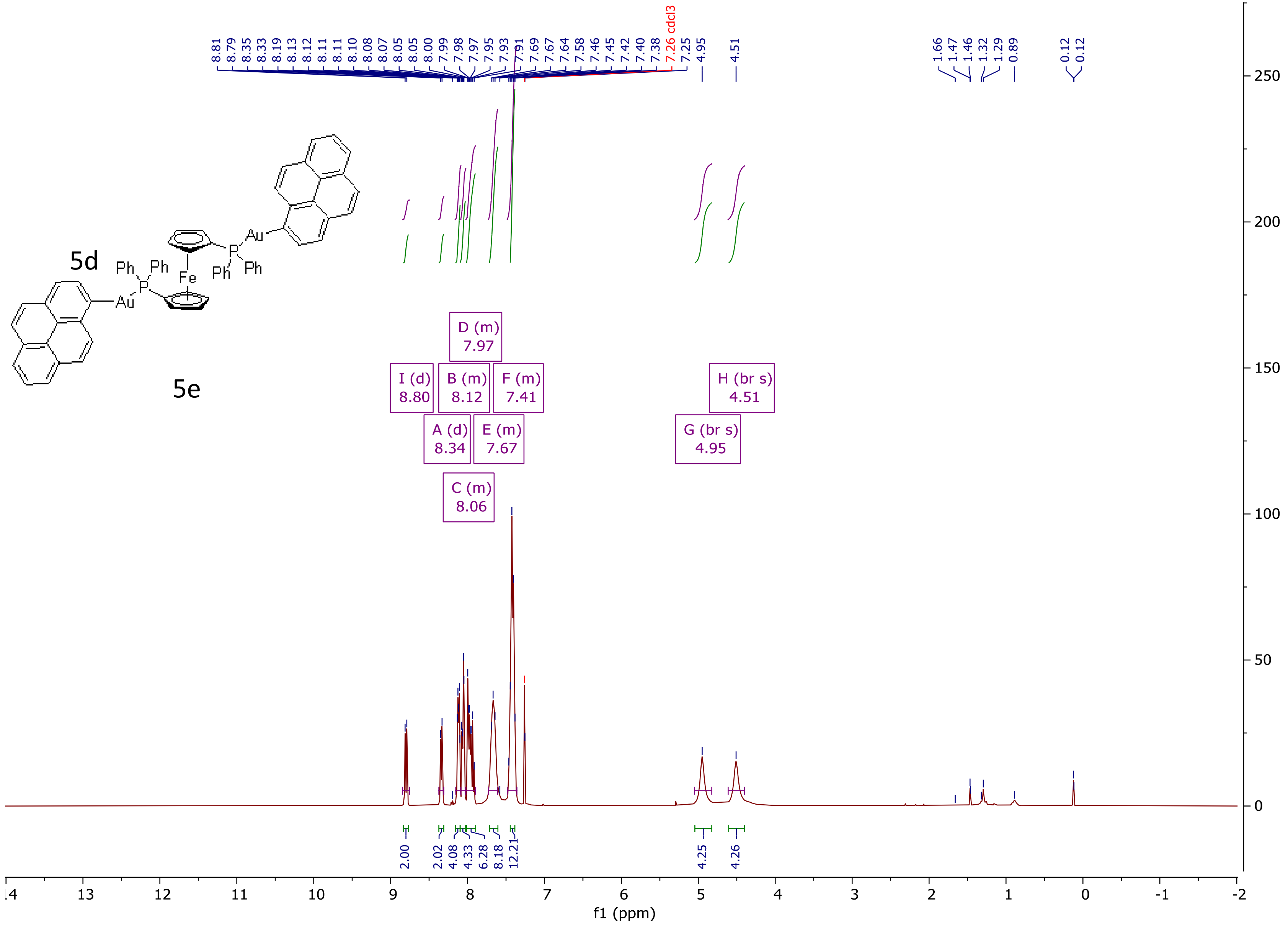
5d
5e
Ph Ph P Au Fe Ph Ph P Au
7.26 cdcl3
8.81 8.79 8.35 8.33 8.19 8.13 8.12 8.11 8.11 8.10 8.08 8.07 8.05 8.05 8.00 7.99 7.98 7.97 7.95 7.93 7.91 7.69 7.67 7.64 7.58 7.46 7.45 7.42 7.40 7.38 7.25 4.95 4.51
1.66 1.47 1.46 1.32 1.29 0.89 0.12 0.12
D (m) 7.97
I (d) 8.80
B (m) 8.12
F (m) 7.41
H (br s) 4.51
A (d) 8.34
E (m) 7.67
G (br s) 4.95
C (m) 8.06
2.00 2.02 4.08 4.33 6.28 8.18 12.21 4.25 4.26
f1 (ppm)

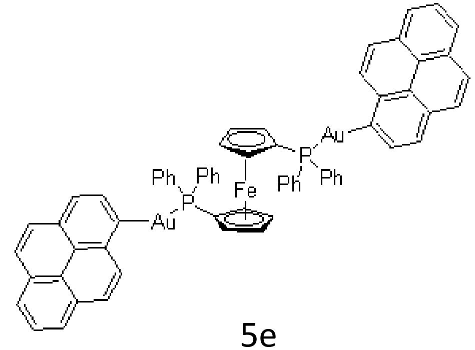

5e

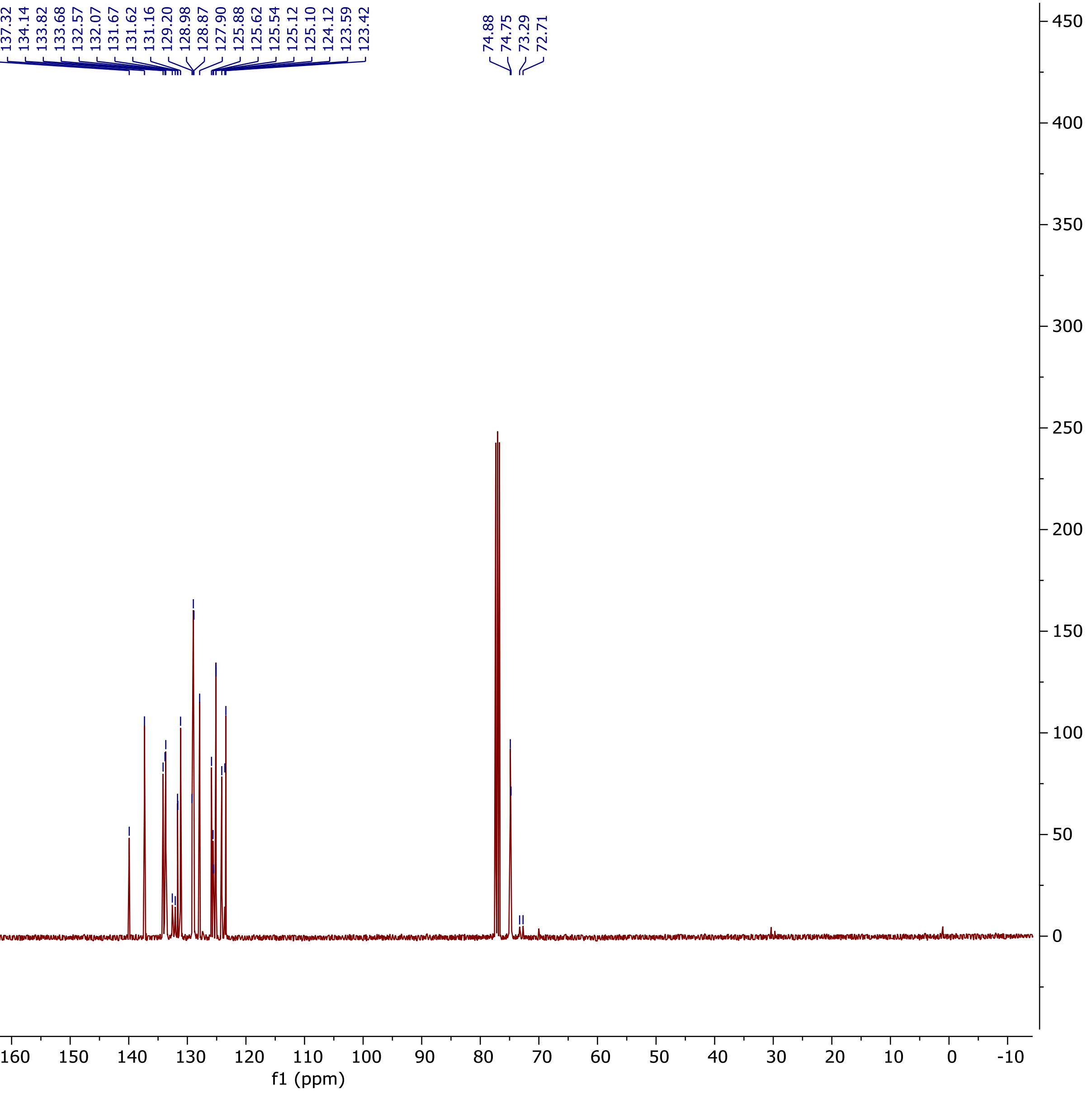
139.92
137.32
134.14
133.82
133.68
132.57
132.07
131.67
131.62
131.16
129.20
128.98
128.87
127.90
125.88
125.62
125.54
125.12
125.10
124.12
123.59
123.42
74.88
74.75
73.29
72.71
f1 (ppm)

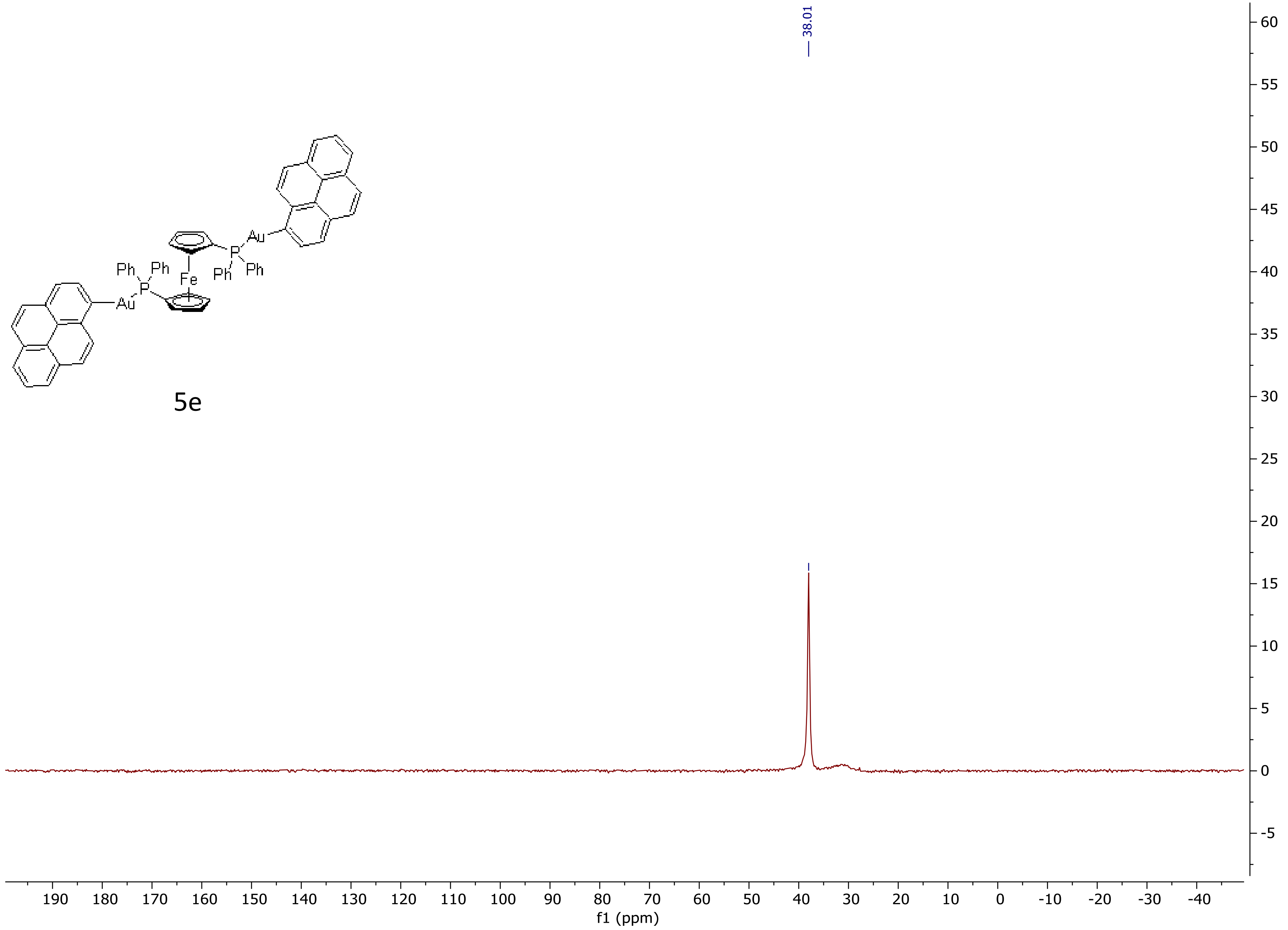

5e

NHC Au complexes

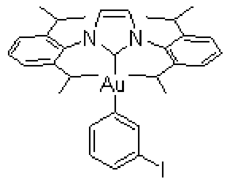

5f

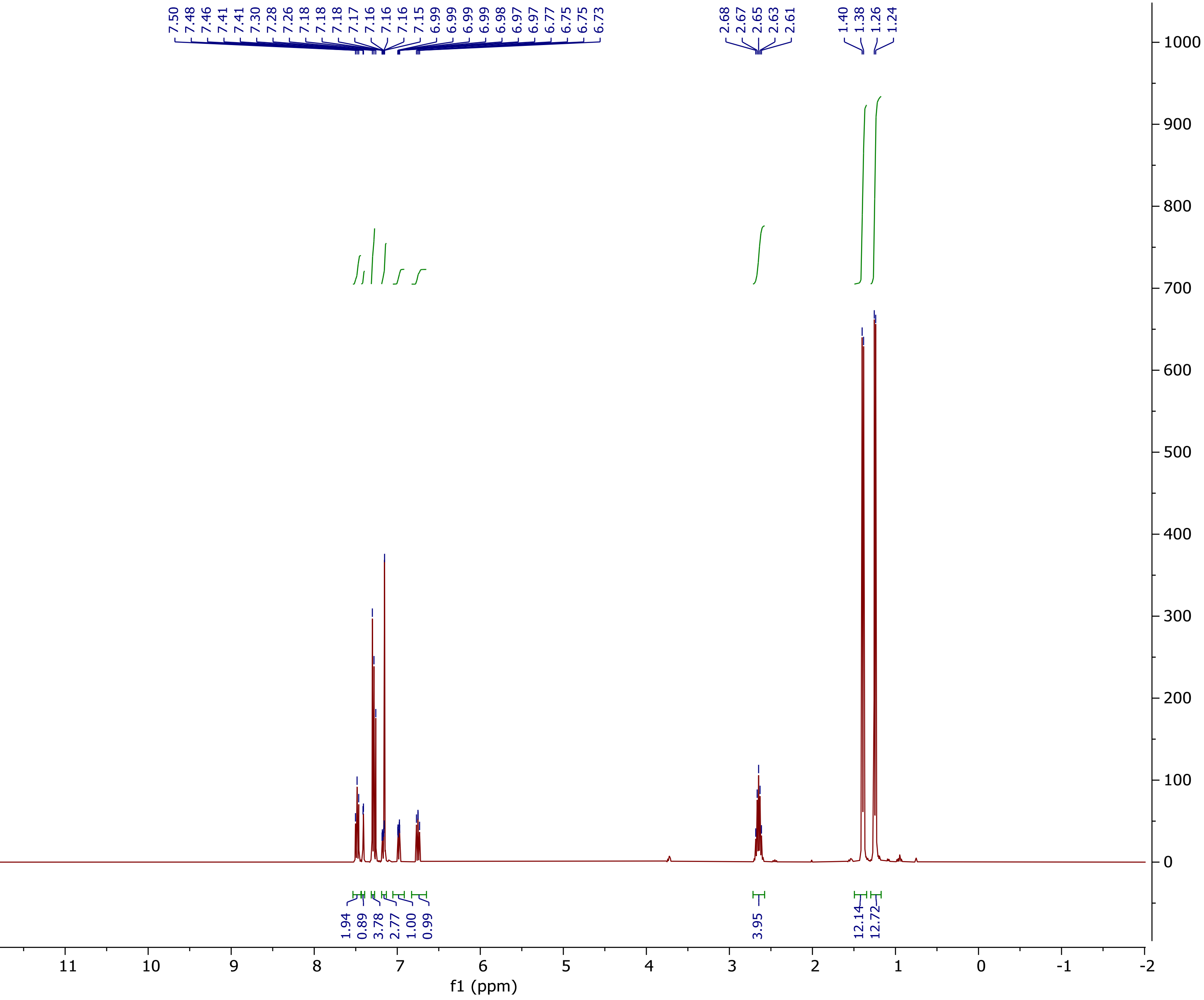

7.50 7.48 7.46 7.41 7.41 7.30 7.28 7.26 7.18 7.18 7.18 7.17 7.16 7.16 7.16 7.15 6.99 6.99 6.99 6.99 6.98 6.97 6.97 6.77 6.75 6.75 6.73
2.68 2.67 2.65 2.63 2.61
1.40 1.38 1.26 1.24
1.94 0.89 3.78 2.77 1.00 0.99
3.95
12.14 12.72
f1 (ppm)

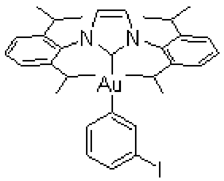
N
N
Au
I

5f

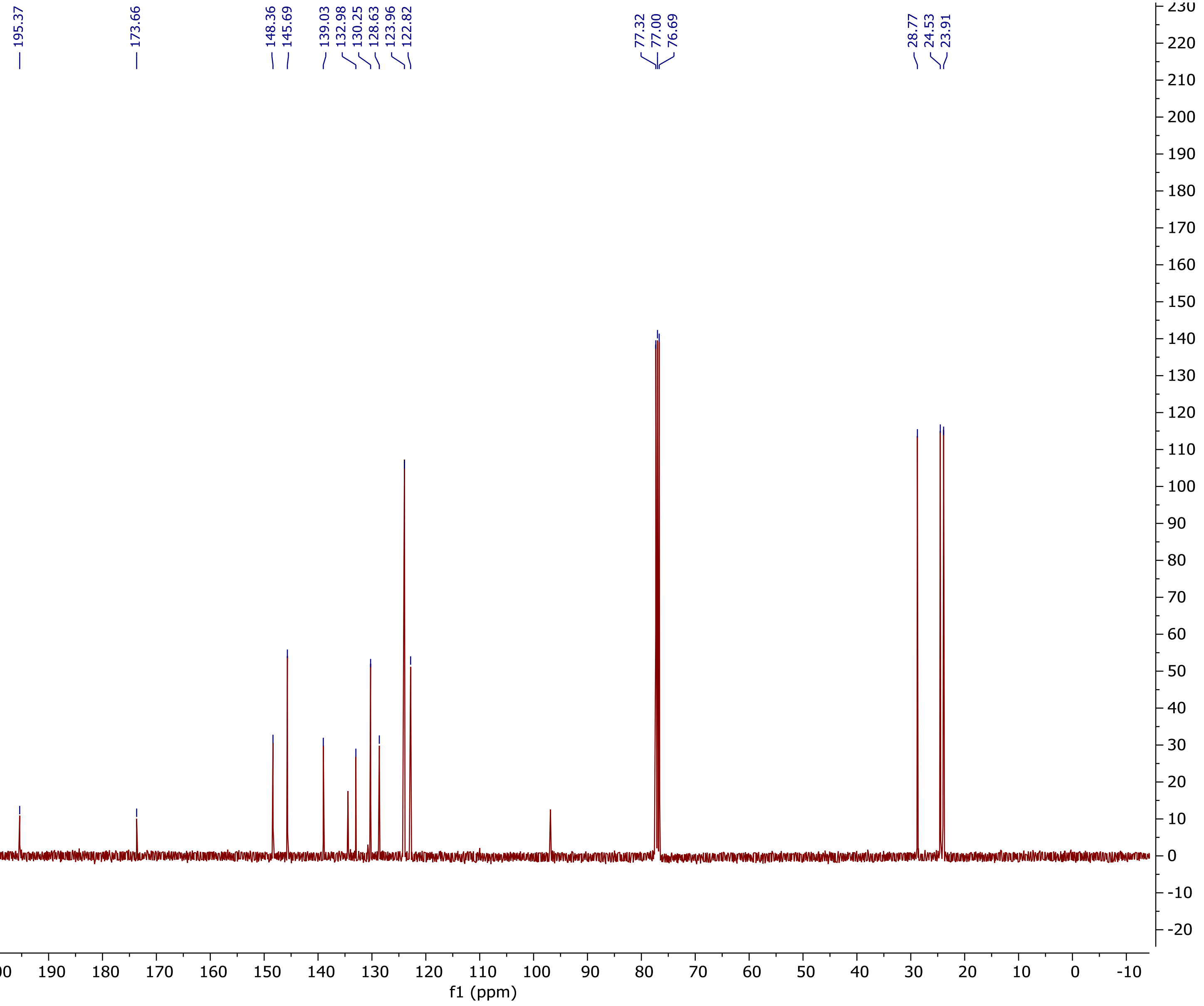
195.37
173.66
148.36
145.69
139.03
132.98
130.25
128.63
123.96
122.82
77.32
77.00
76.69
28.77
24.53
23.91
f1 (ppm)

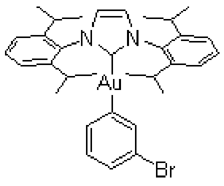

5g

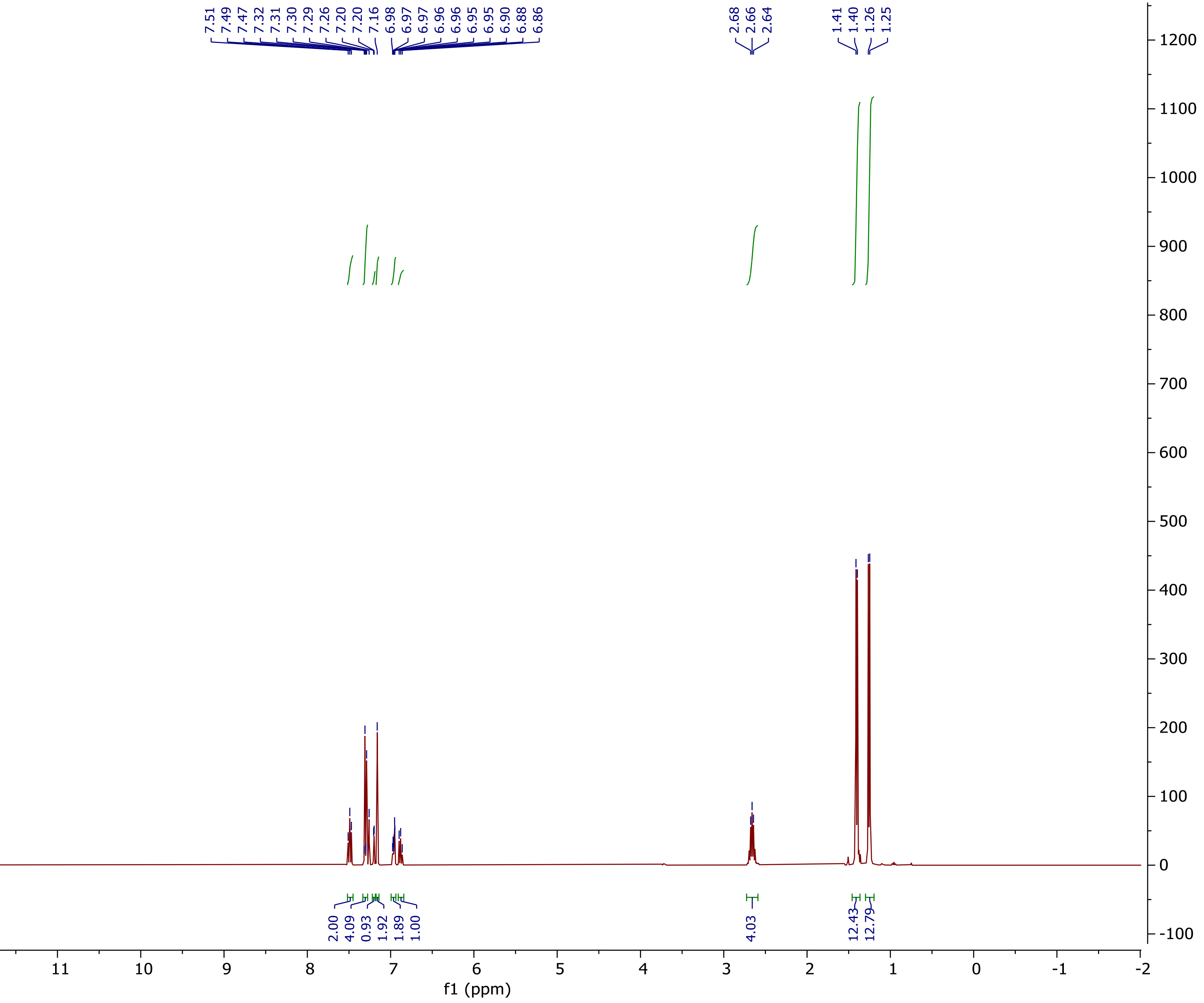
7.51
7.49
7.47
7.32
7.31
7.30
7.29
7.26
7.20
7.20
7.16
6.98
6.97
6.97
6.96
6.96
6.95
6.95
6.90
6.88
6.86
2.68
2.66
2.64
1.41
1.40
1.26
1.25
2.00
4.09
0.93
1.92
1.89
1.00
4.03
12.43
12.79
f1 (ppm)

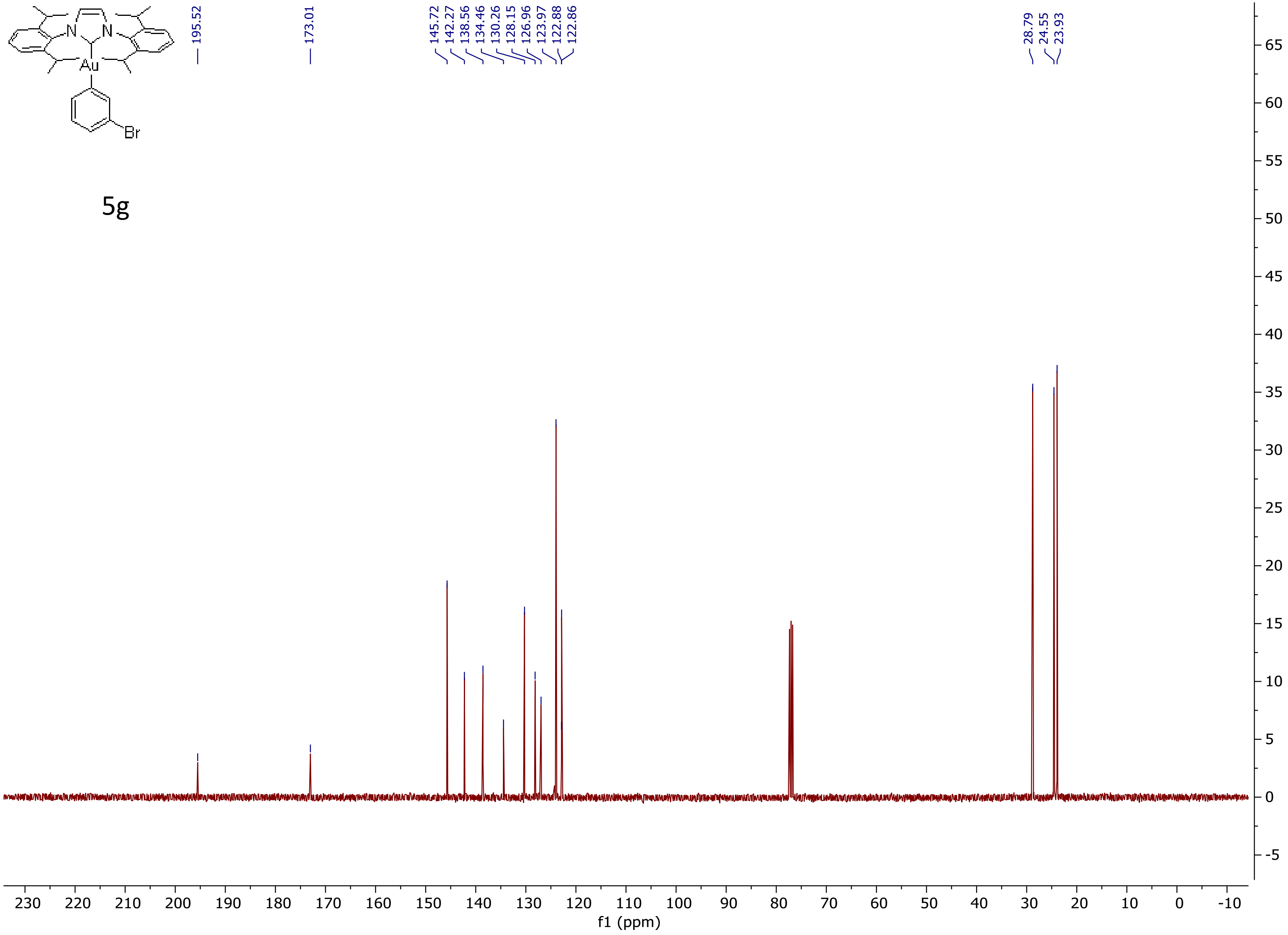
Br
Au
N
N
195.52
173.01
145.72
142.27
138.56
134.46
130.26
128.15
126.96
123.97
122.88
122.86
28.79
24.55
23.93
f1 (ppm)

5g

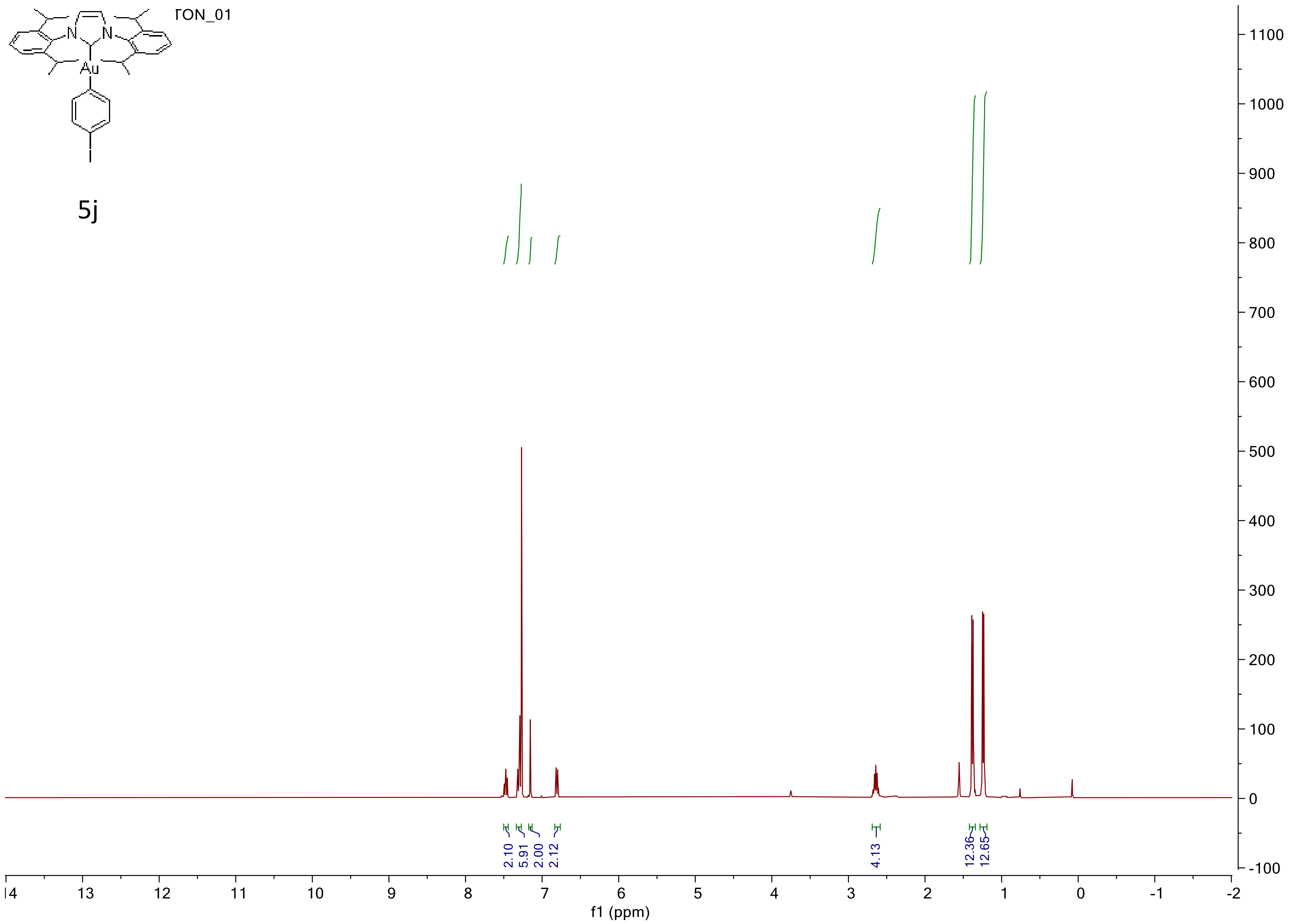

TON_01
Au
N
N
5j
2.10
5.91
2.00
2.12
4.13
12.36
12.65
1100
1000
900
800
700
600
500
400
300
200
100
0
-100
14
13
12
11
10
9
8
7
6
5
4
3
2
1
0
-1
-2
f1 (ppm)

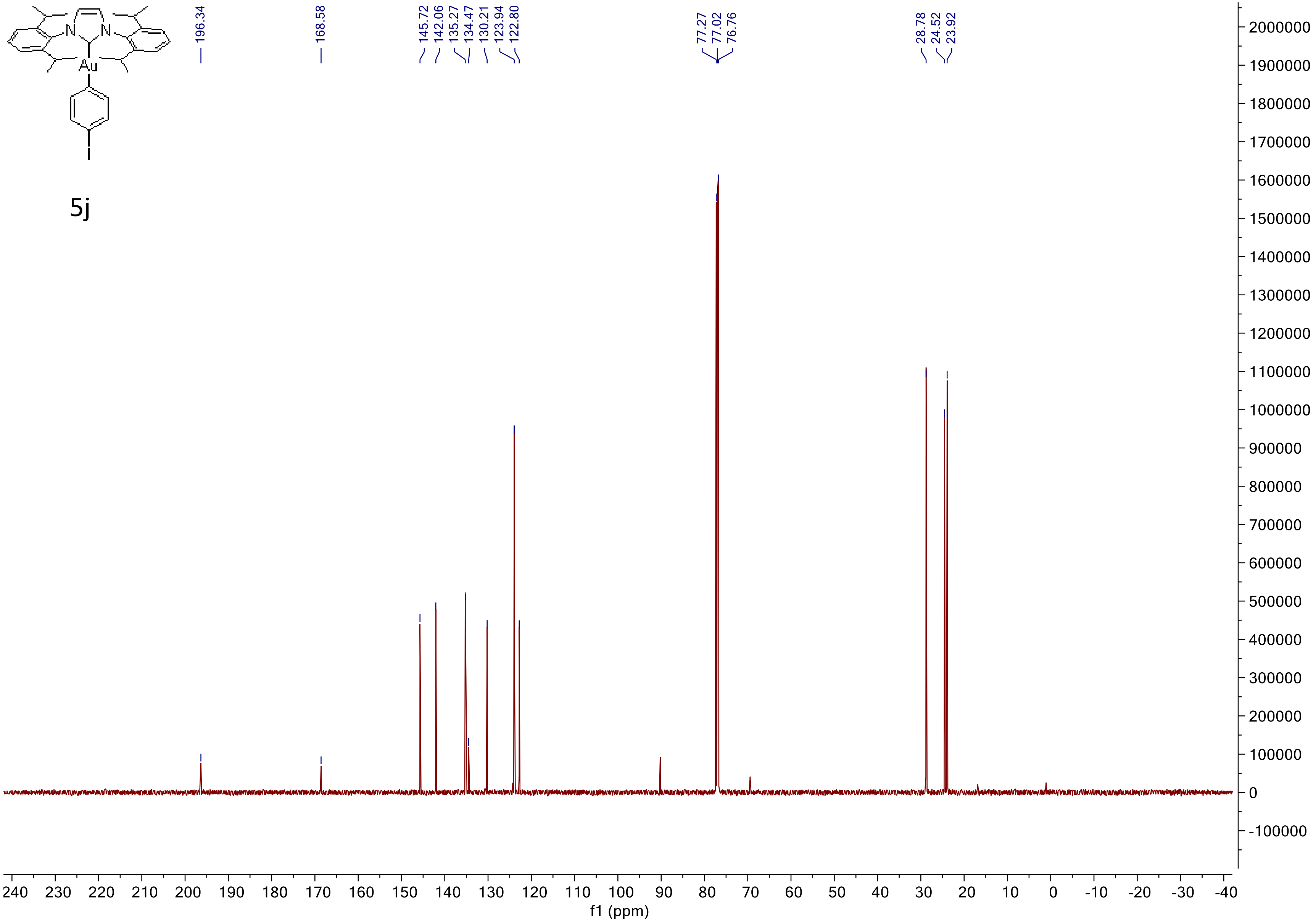
196.34
168.58
145.72
142.06
135.27
134.47
130.21
123.94
122.80
77.27
77.02
76.76
28.78
24.52
23.92
f1 (ppm)

5j

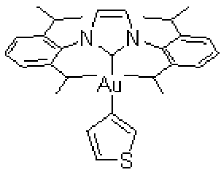

5k

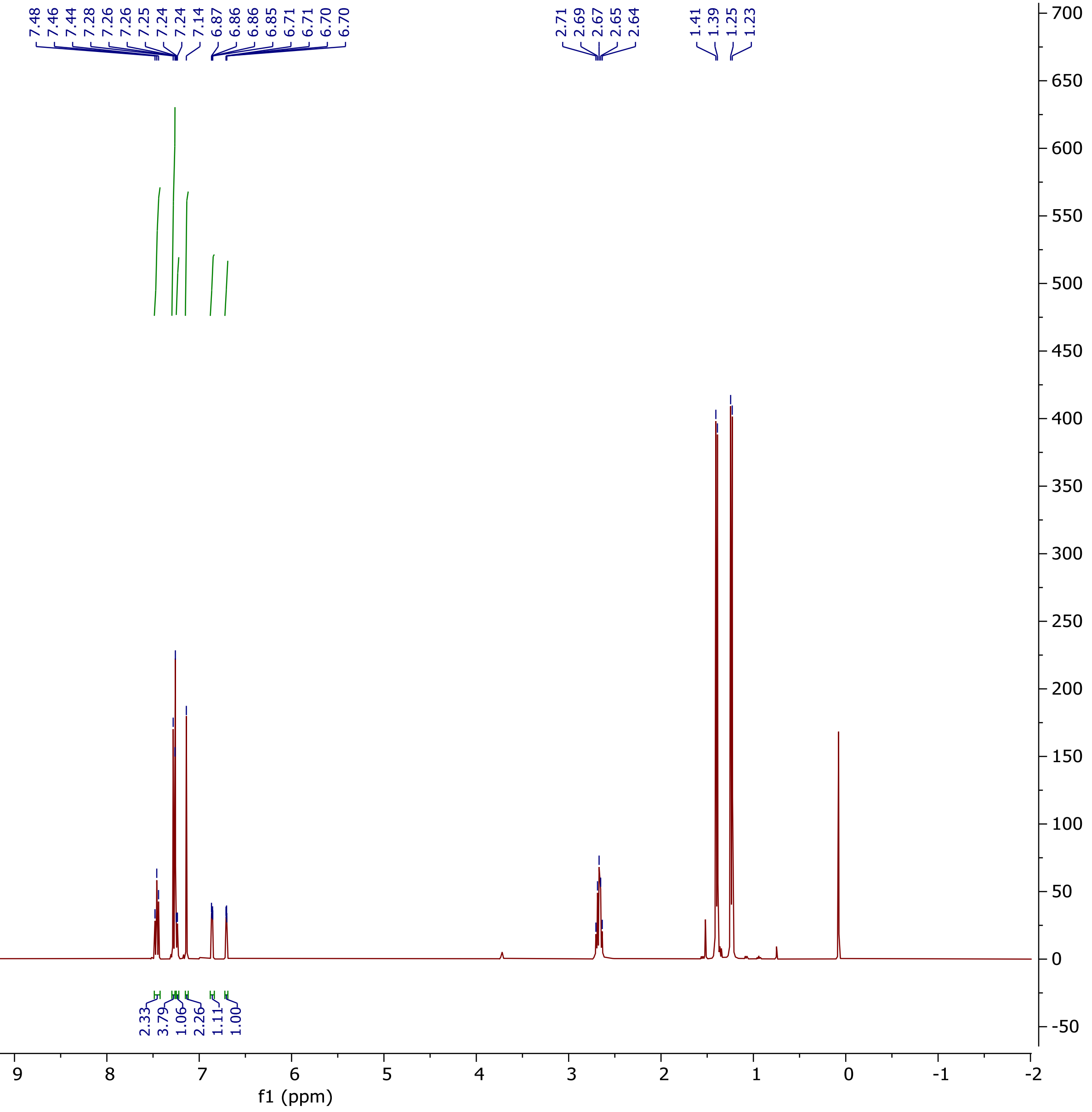

7.48
7.46
7.44
7.28
7.26
7.26
7.25
7.24
7.24
7.14
6.87
6.86
6.86
6.85
6.71
6.71
6.70
6.70
2.71
2.69
2.67
2.65
2.64
1.41
1.39
1.25
1.23
2.33
3.79
1.06
2.26
1.11
1.00
f1 (ppm)

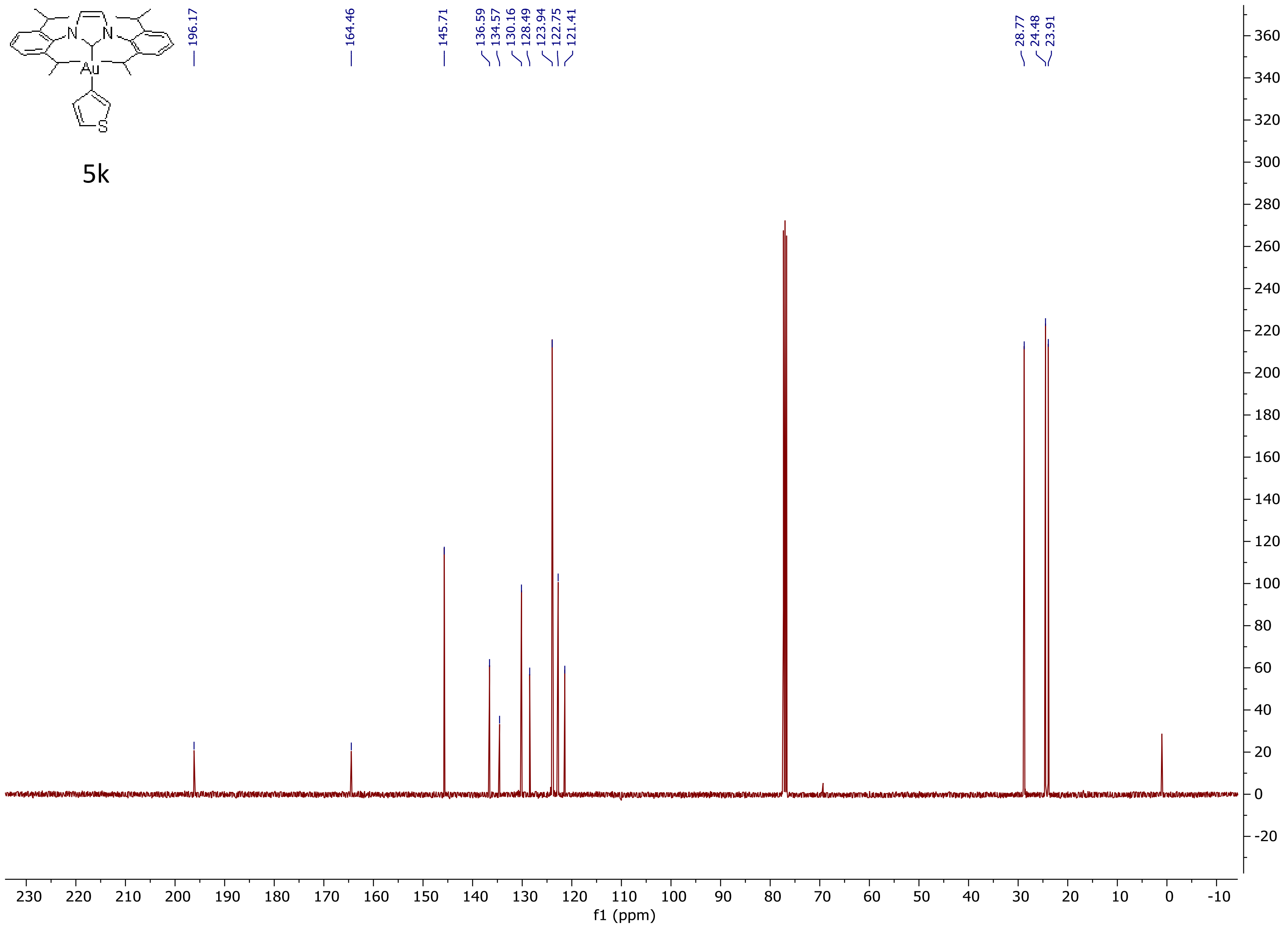

5k
196.17
164.46
145.71
136.59
134.57
130.16
128.49
123.94
122.75
121.41
28.77
24.48
23.91
f1 (ppm)

Polycyclic aromatic hydrocarbon-based Au(I) complexes

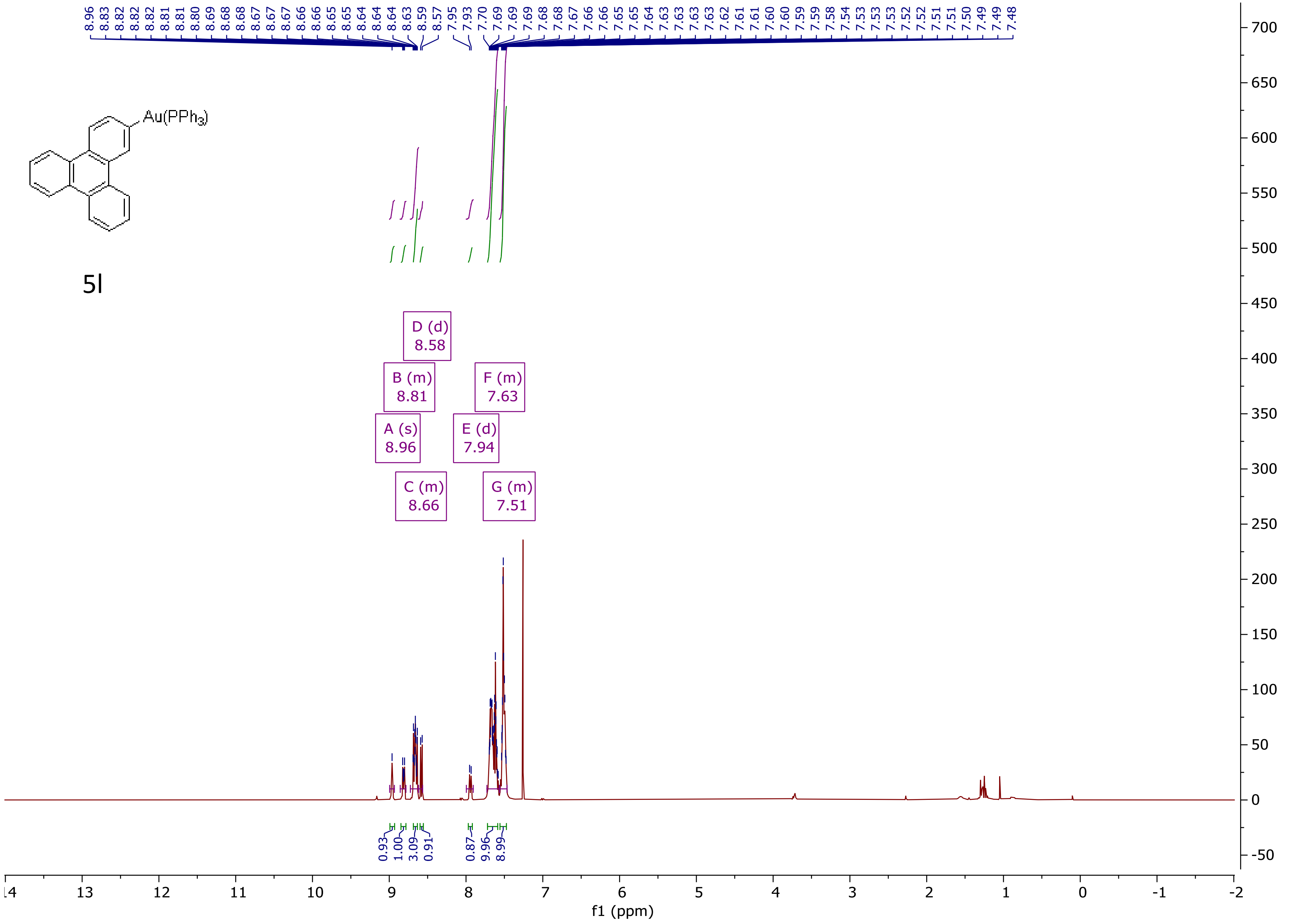
8.96 8.83 8.82 8.82 8.82 8.81 8.81 8.80 8.69 8.68 8.68 8.67 8.67 8.67 8.66 8.66 8.65 8.65 8.64 8.64 8.64 8.63 8.59 8.57 7.95 7.93 7.70 7.69 7.69 7.69 7.68 7.68 7.67 7.66 7.66 7.65 7.65 7.64 7.63 7.63 7.63 7.63 7.62 7.61 7.61 7.60 7.59 7.59 7.58 7.54 7.53 7.53 7.53 7.52 7.52 7.51 7.51 7.50 7.49 7.49 7.48
Au(PPh3)
5l
D (d) 8.58
B (m) 8.81
F (m) 7.63
A (s) 8.96
E (d) 7.94
C (m) 8.66
G (m) 7.51
0.93 1.00 3.09 0.91 0.87 9.96 8.99
f1 (ppm)

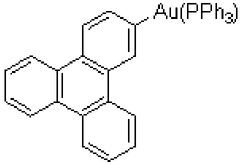
Au(PPh3)

5l

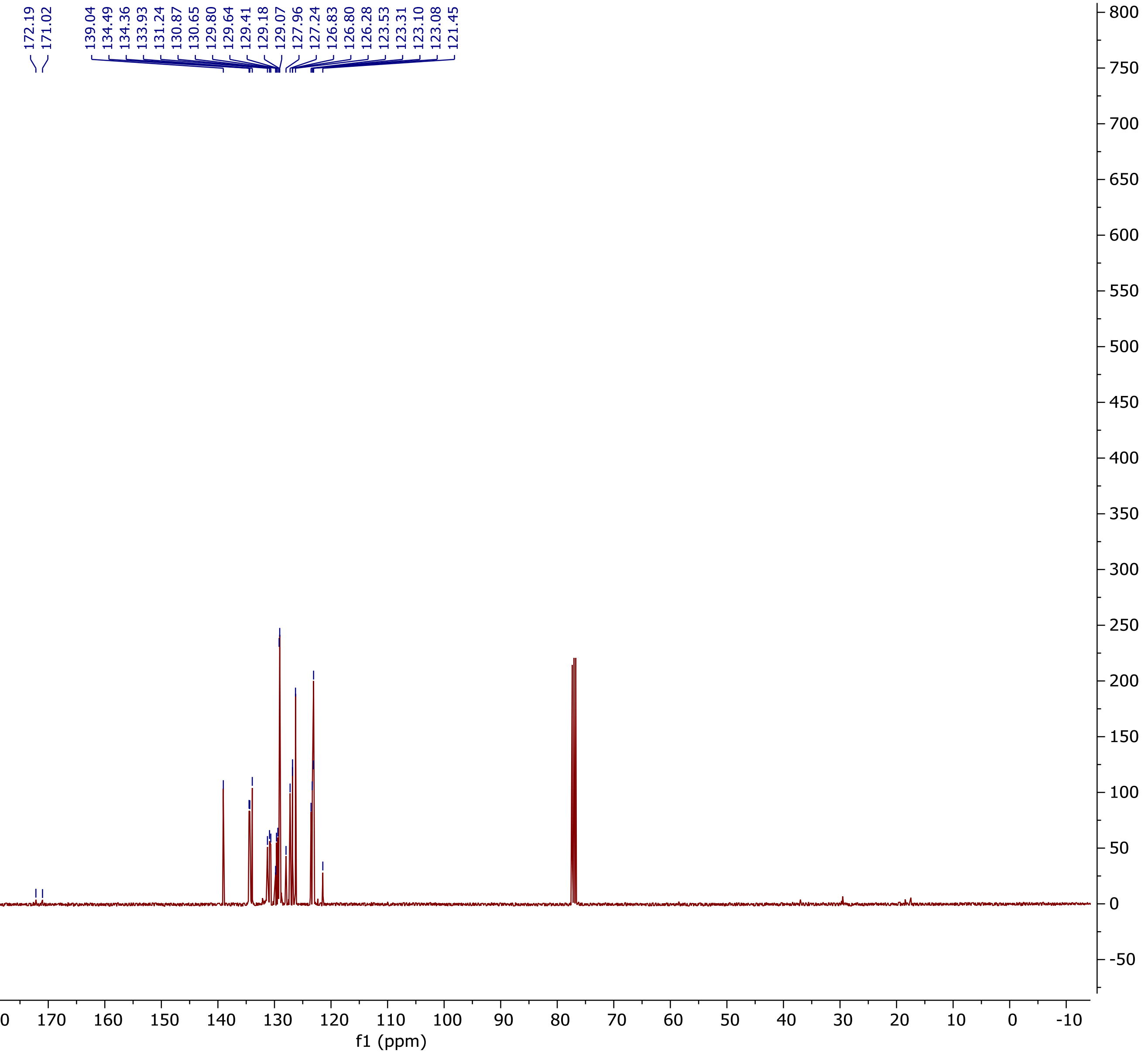
172.19
171.02
139.04
134.49
134.36
133.93
131.24
130.87
130.65
129.80
129.64
129.41
129.18
129.07
127.96
127.24
126.83
126.80
126.28
123.53
123.31
123.10
123.08
121.45
f1 (ppm)
230 220 210 200 190 180 170 160 150 140 130 120 110 100 90 80 70 60 50 40 30 20 10 0 -10
800 750 700 650 600 550 500 450 400 350 300 250 200 150 100 50 0 -50

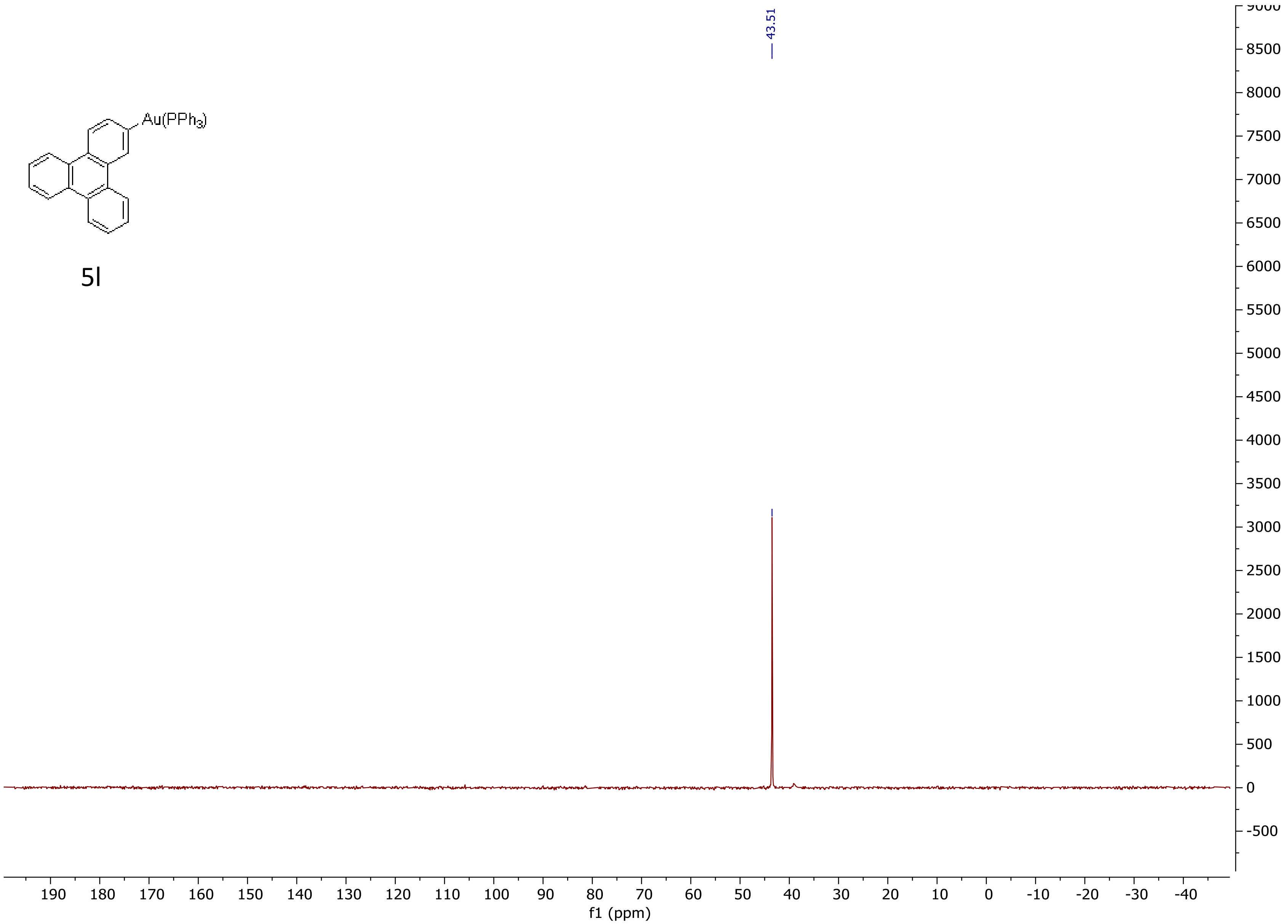
Au(PPh3)
5l
43.51
9000
8500
8000
7500
7000
6500
6000
5500
5000
4500
4000
3500
3000
2500
2000
1500
1000
500
0
-500
190 180 170 160 150 140 130 120 110 100 90 80 70 60 50 40 30 20 10 0 -10 -20 -30 -40
f1 (ppm)

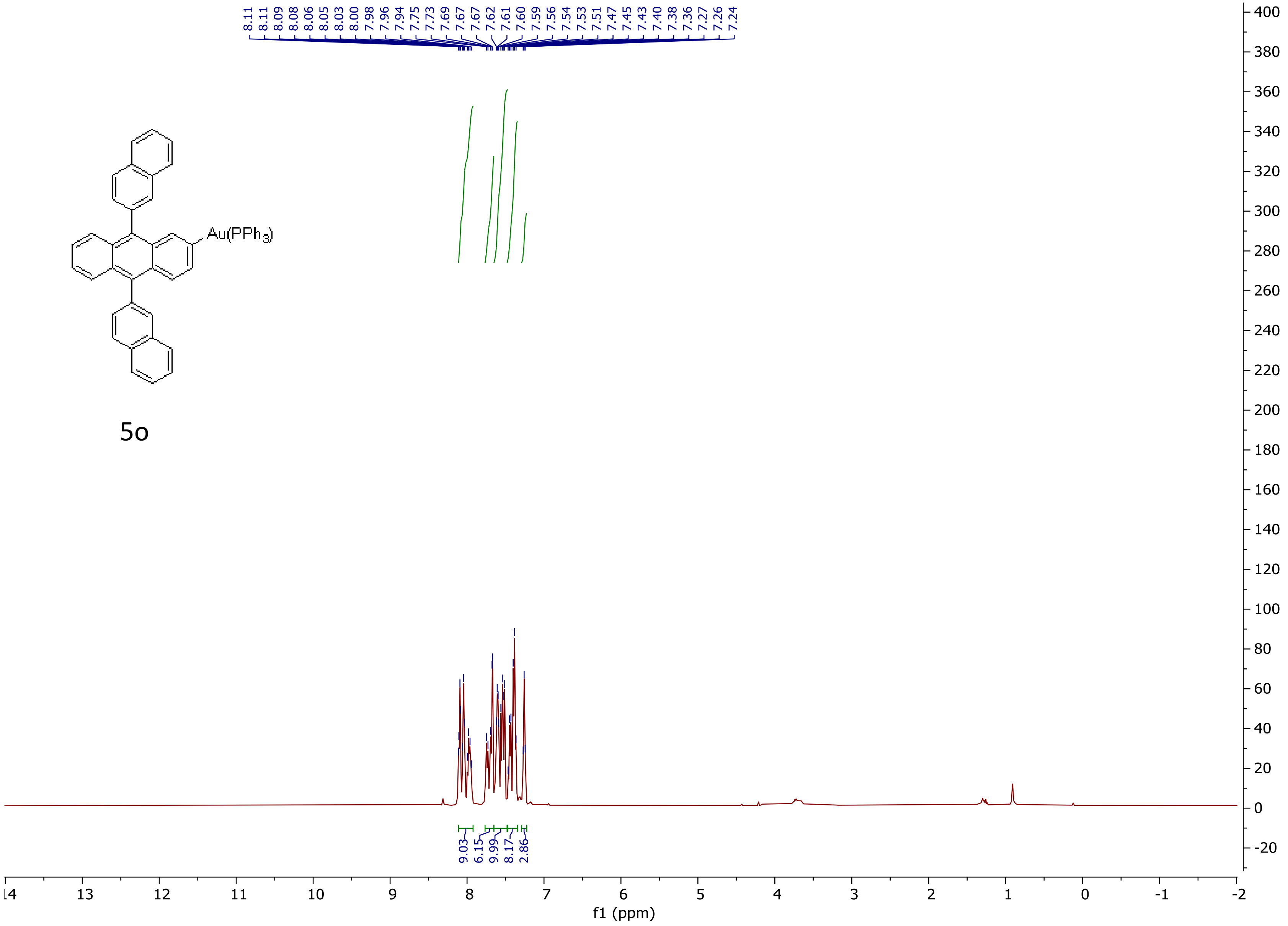
Au(PPh3)
5o
8.11
8.11
8.09
8.08
8.06
8.05
8.03
8.00
7.98
7.96
7.94
7.75
7.73
7.69
7.67
7.67
7.62
7.61
7.60
7.59
7.56
7.54
7.53
7.51
7.47
7.45
7.43
7.40
7.38
7.36
7.27
7.26
7.24
9.03
6.15
9.99
8.17
2.86
f1 (ppm)

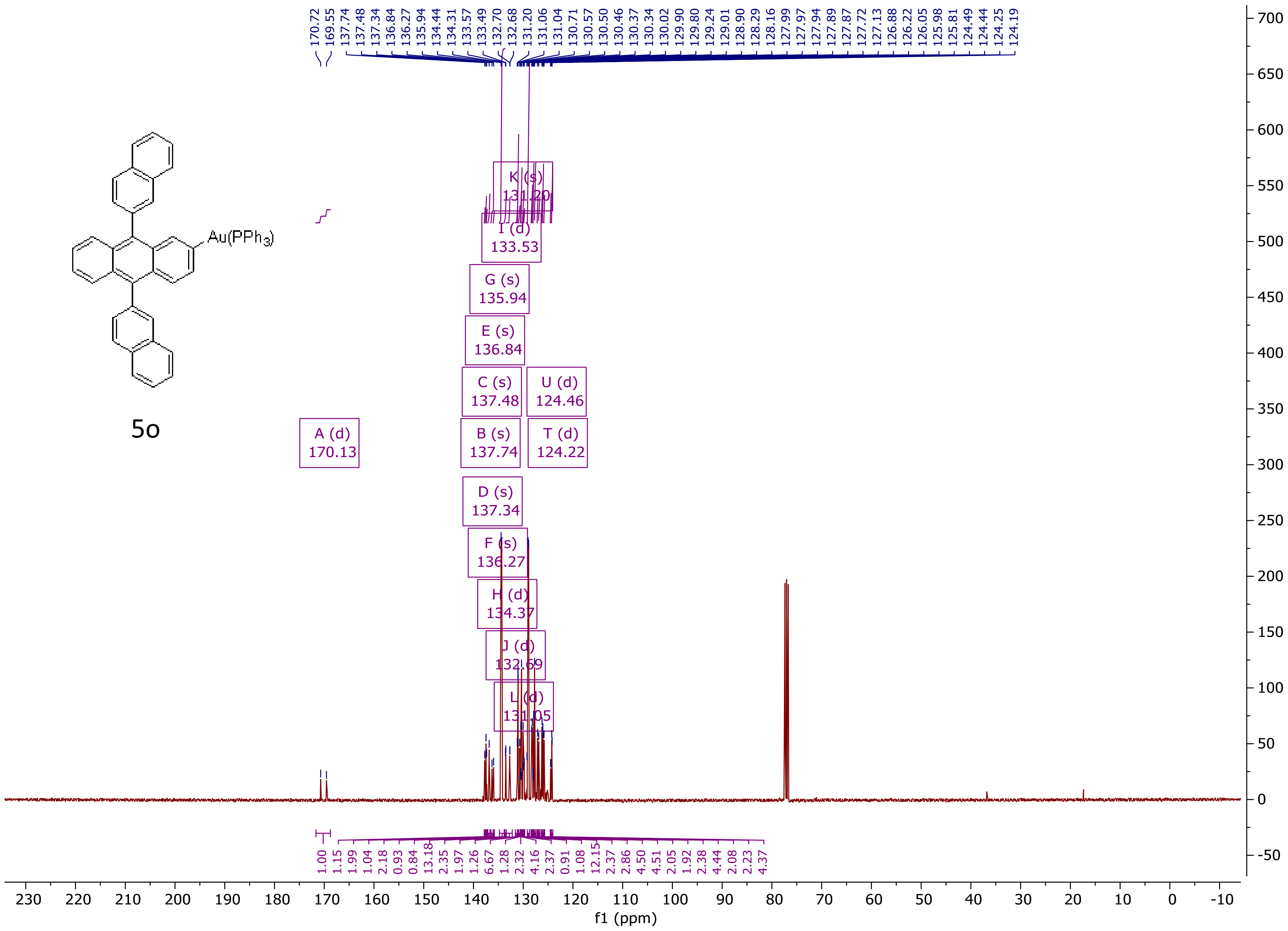
Au(PPh3)
170.72
169.55
137.74
137.48
137.34
136.84
136.27
135.94
134.44
134.31
133.57
133.49
132.70
132.68
131.20
131.06
131.04
130.71
130.57
130.50
130.46
130.37
130.34
130.02
129.90
129.80
129.24
129.01
128.90
128.29
128.16
127.99
127.97
127.94
127.89
127.87
127.72
127.13
126.88
126.22
126.05
125.98
125.81
124.49
124.44
124.25
124.19
A (d) 170.13
B (s) 137.74
C (s) 137.48
D (s) 137.34
E (s) 136.84
F (s) 136.27
G (s) 135.94
H (d) 134.37
I (d) 133.53
J (d) 132.69
K (s) 131.20
L (d) 131.05
T (d) 124.22
U (d) 124.46
1.00
1.15
1.99
1.04
2.18
0.93
0.84
13.18
2.35
1.97
1.26
6.67
1.28
2.32
4.16
2.37
0.91
1.08
12.15
2.37
2.86
4.50
4.51
2.05
1.92
2.38
4.44
2.08
2.23
4.37
f1 (ppm)

5o

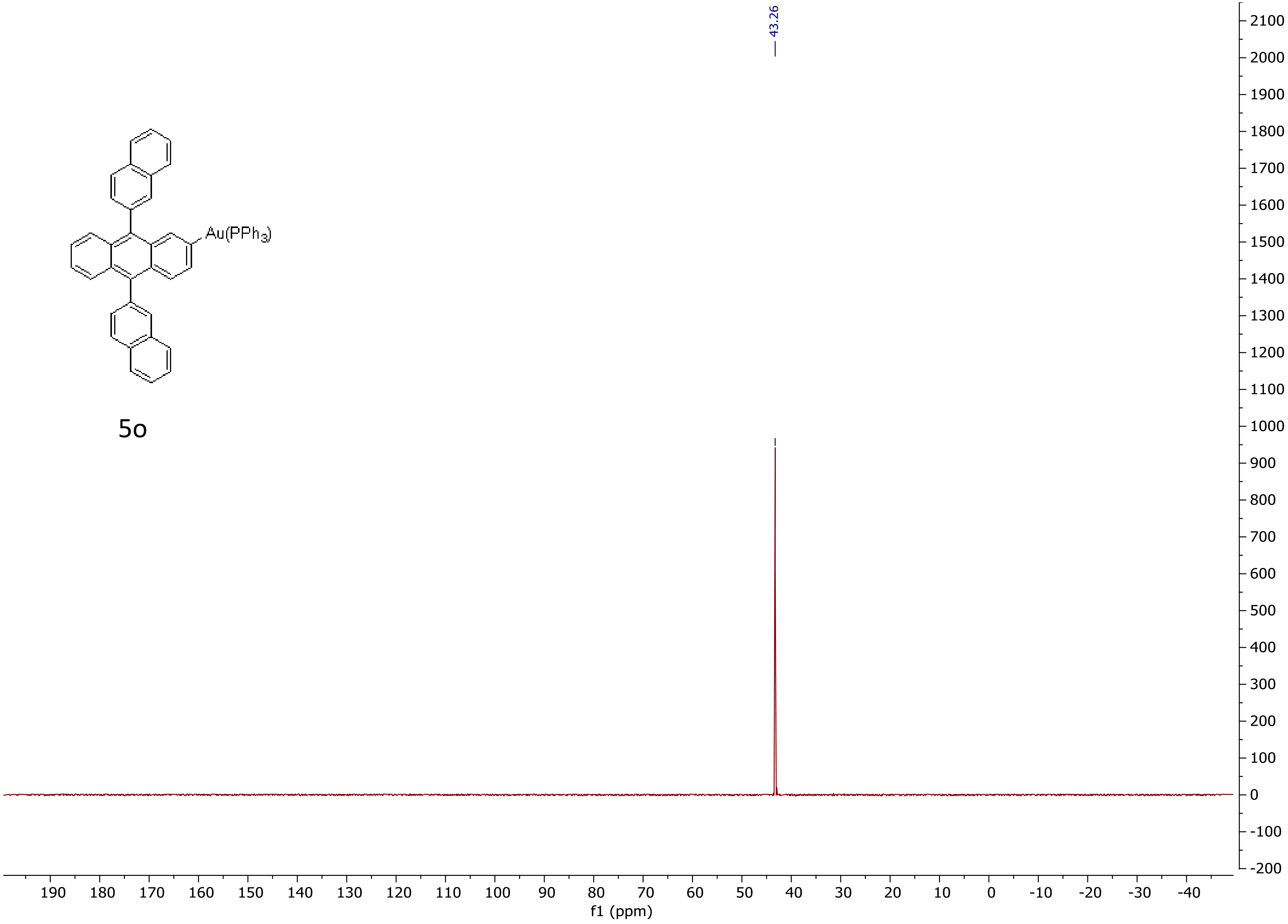
Au(PPh3)
5o
43.26
2100
2000
1900
1800
1700
1600
1500
1400
1300
1200
1100
1000
900
800
700
600
500
400
300
200
100
0
-100
-200
190 180 170 160 150 140 130 120 110 100 90 80 70 60 50 40 30 20 10 0 -10 -20 -30 -40
f1 (ppm)

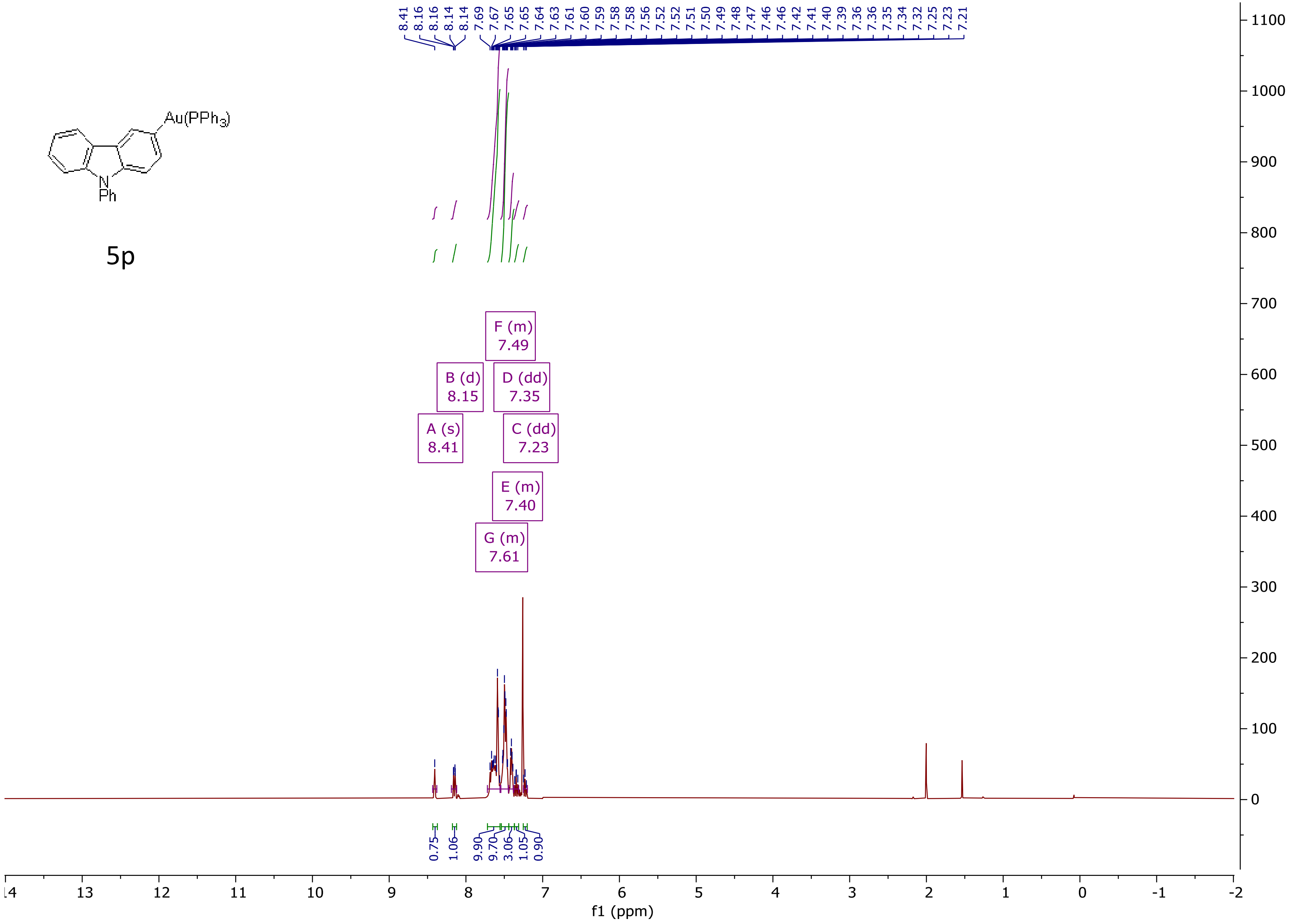
Au(PPh3)
N
Ph
F (m)
7.49
B (d)
8.15
D (dd)
7.35
A (s)
8.41
C (dd)
7.23
E (m)
7.40
G (m)
7.61
f1 (ppm)

5p

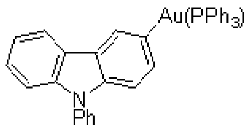
Au(PPh3)
N
Ph

5p

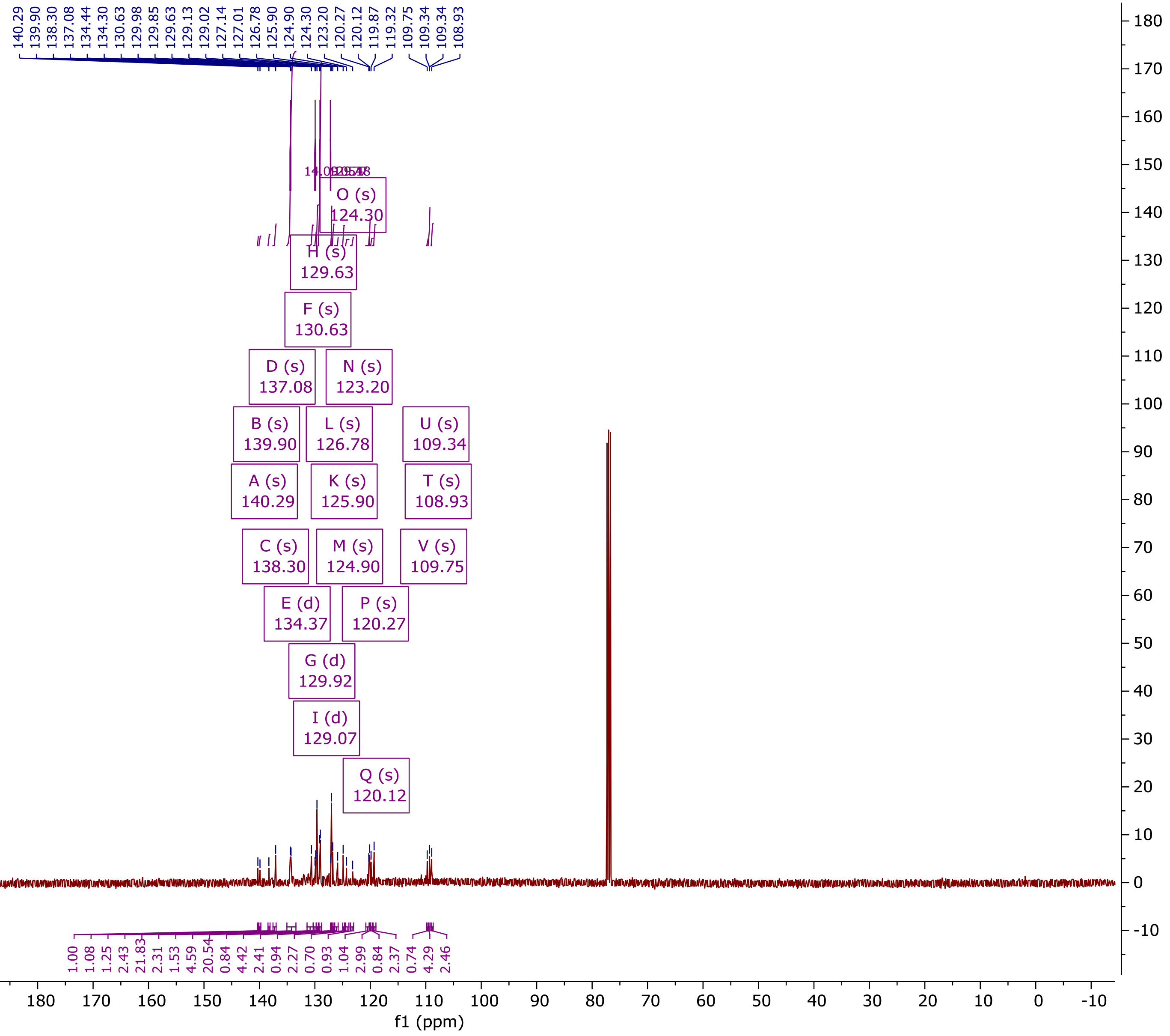
140.29
139.90
138.30
137.08
134.44
134.30
130.63
129.98
129.85
129.63
129.13
129.02
127.14
127.01
126.78
125.90
124.90
124.30
123.20
120.27
120.12
119.87
119.32
109.75
109.34
109.34
108.93
O (s) 124.30
H (s) 129.63
F (s) 130.63
D (s) 137.08
N (s) 123.20
B (s) 139.90
L (s) 126.78
U (s) 109.34
A (s) 140.29
K (s) 125.90
T (s) 108.93
C (s) 138.30
M (s) 124.90
V (s) 109.75
E (d) 134.37
P (s) 120.27
G (d) 129.92
I (d) 129.07
Q (s) 120.12
1.00
1.08
1.25
2.43
21.83
2.31
1.53
4.59
20.54
0.84
4.42
2.41
0.94
2.27
0.70
0.93
1.04
2.99
0.84
2.37
0.74
4.29
2.46
f1 (ppm)
230 220 210 200 190 180 170 160 150 140 130 120 110 100 90 80 70 60 50 40 30 20 10 0 -10
180 170 160 150 140 130 120 110 100 90 80 70 60 50 40 30 20 10 0 -10

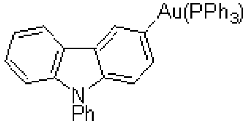

5p

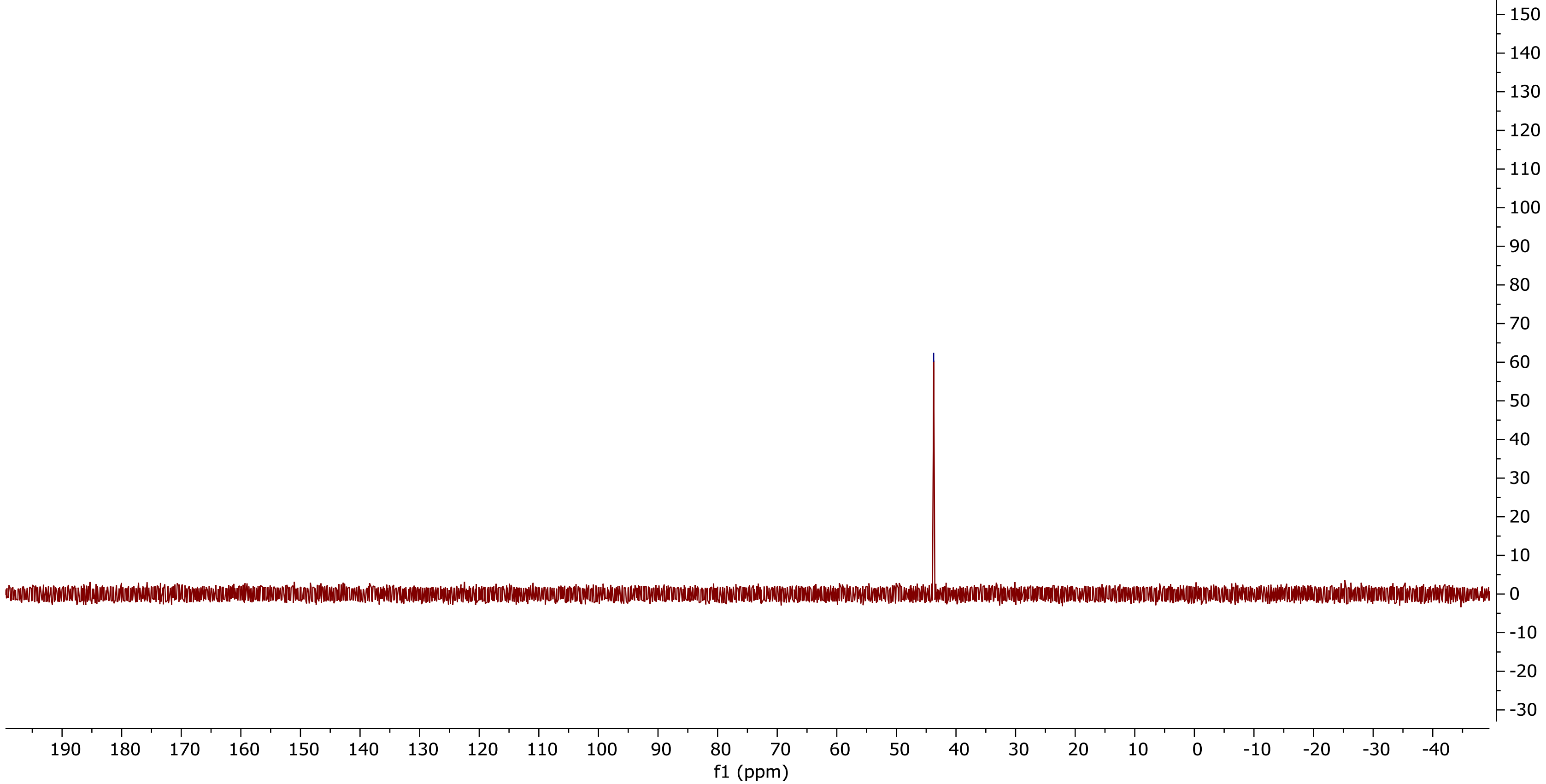

NHC-Bpin Au complexes

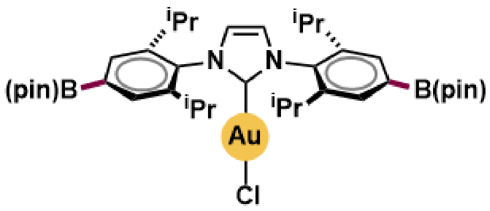
(pin)B
B(pin)
iPr
N
Au
Cl

7

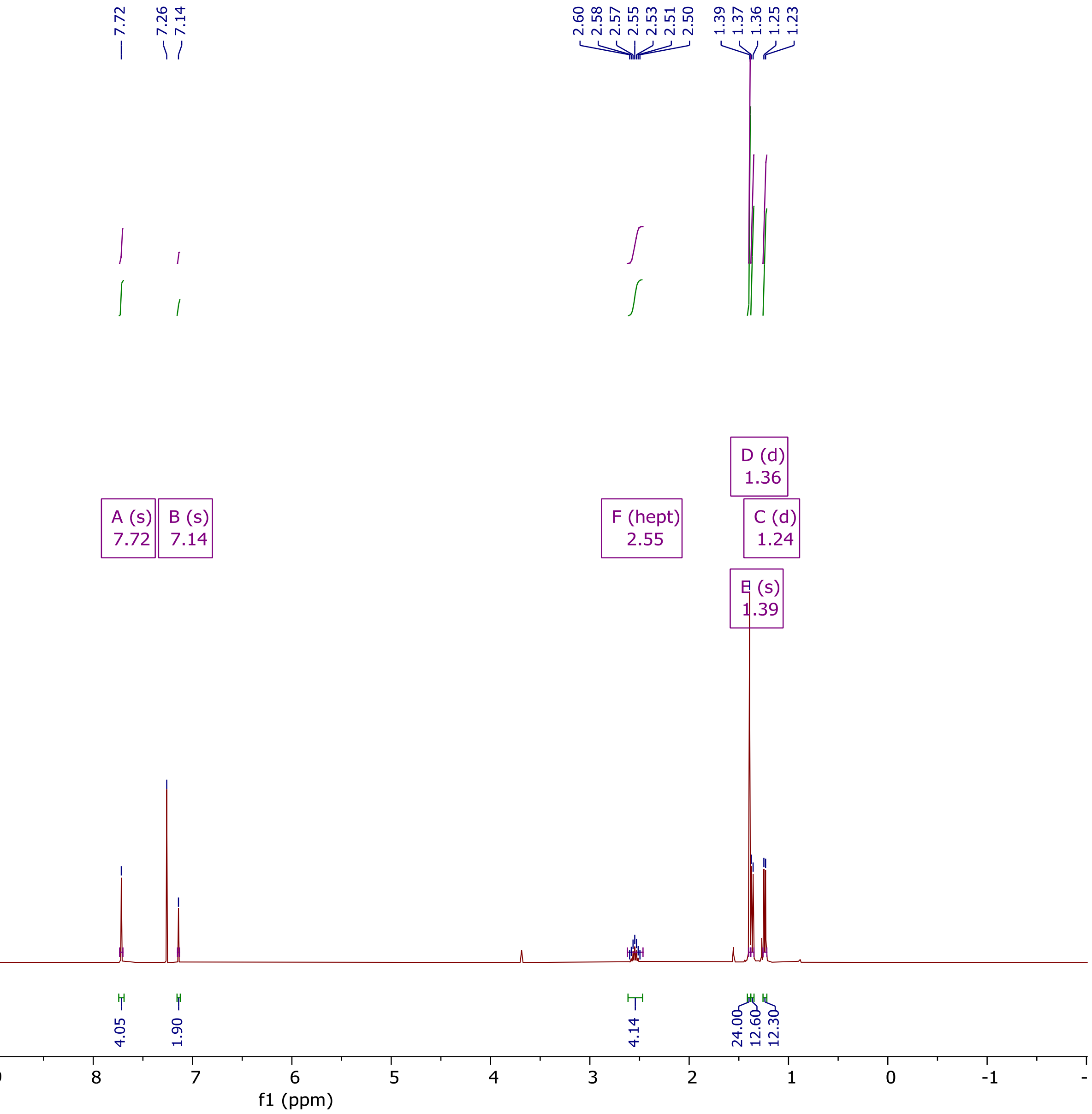
7.72
7.26
7.14
2.60
2.58
2.57
2.55
2.53
2.51
2.50
1.39
1.37
1.36
1.25
1.23
D (d)
1.36
A (s)
7.72
B (s)
7.14
F (hept)
2.55
C (d)
1.24
E (s)
1.39
4.05
1.90
4.14
24.00
12.60
12.30
f1 (ppm)

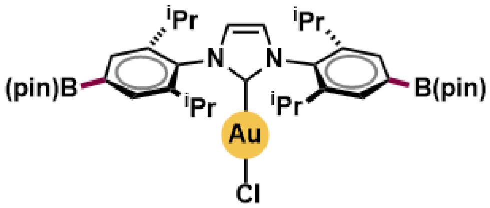
(pin)B
iPr
N
N
B(pin)
Au
Cl

7

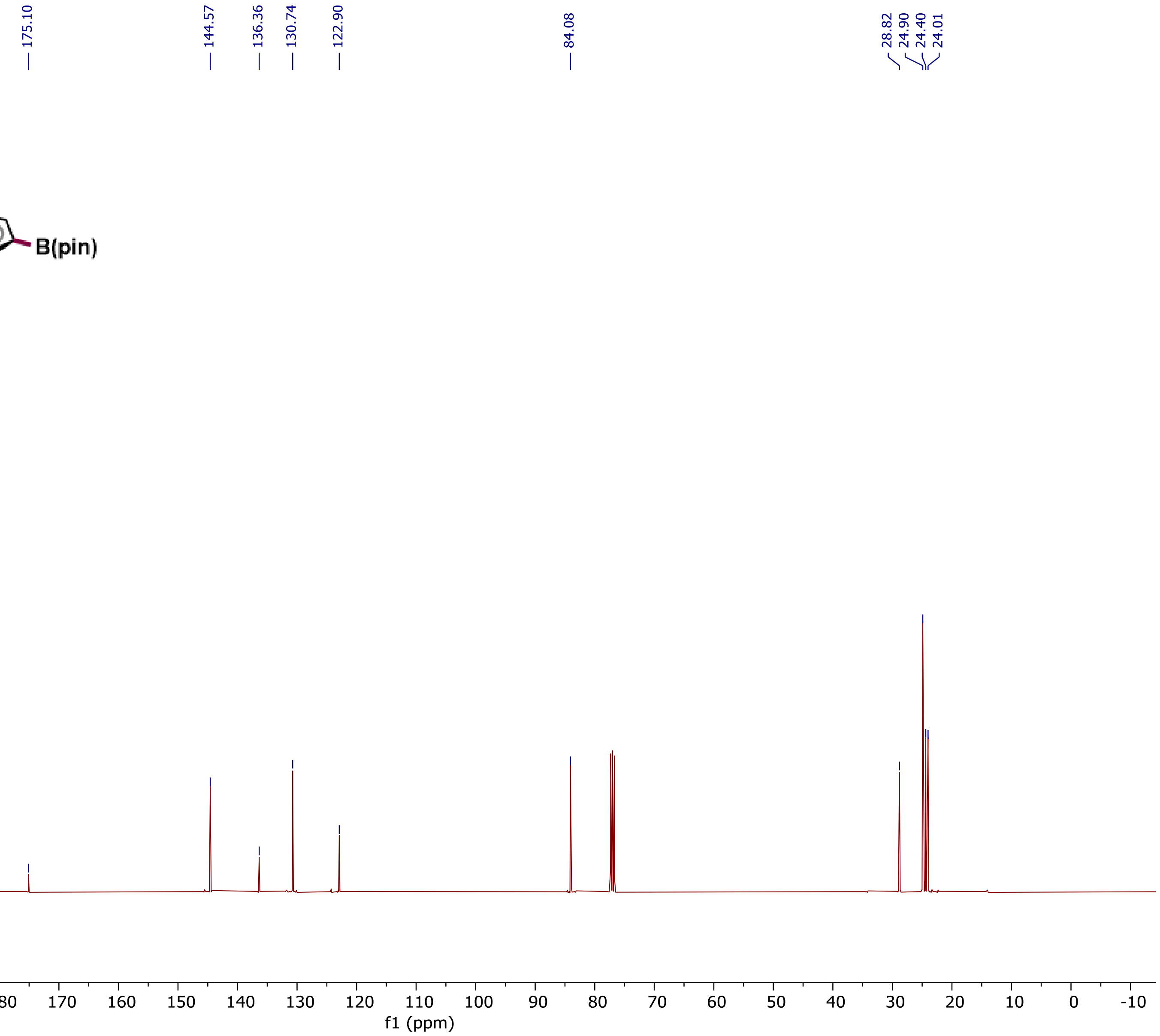
175.10
144.57
136.36
130.74
122.90
84.08
28.82
24.90
24.40
24.01
230 220 210 200 190 180 170 160 150 140 130 120 110 100 90 80 70 60 50 40 30 20 10 0 -10
f1 (ppm)

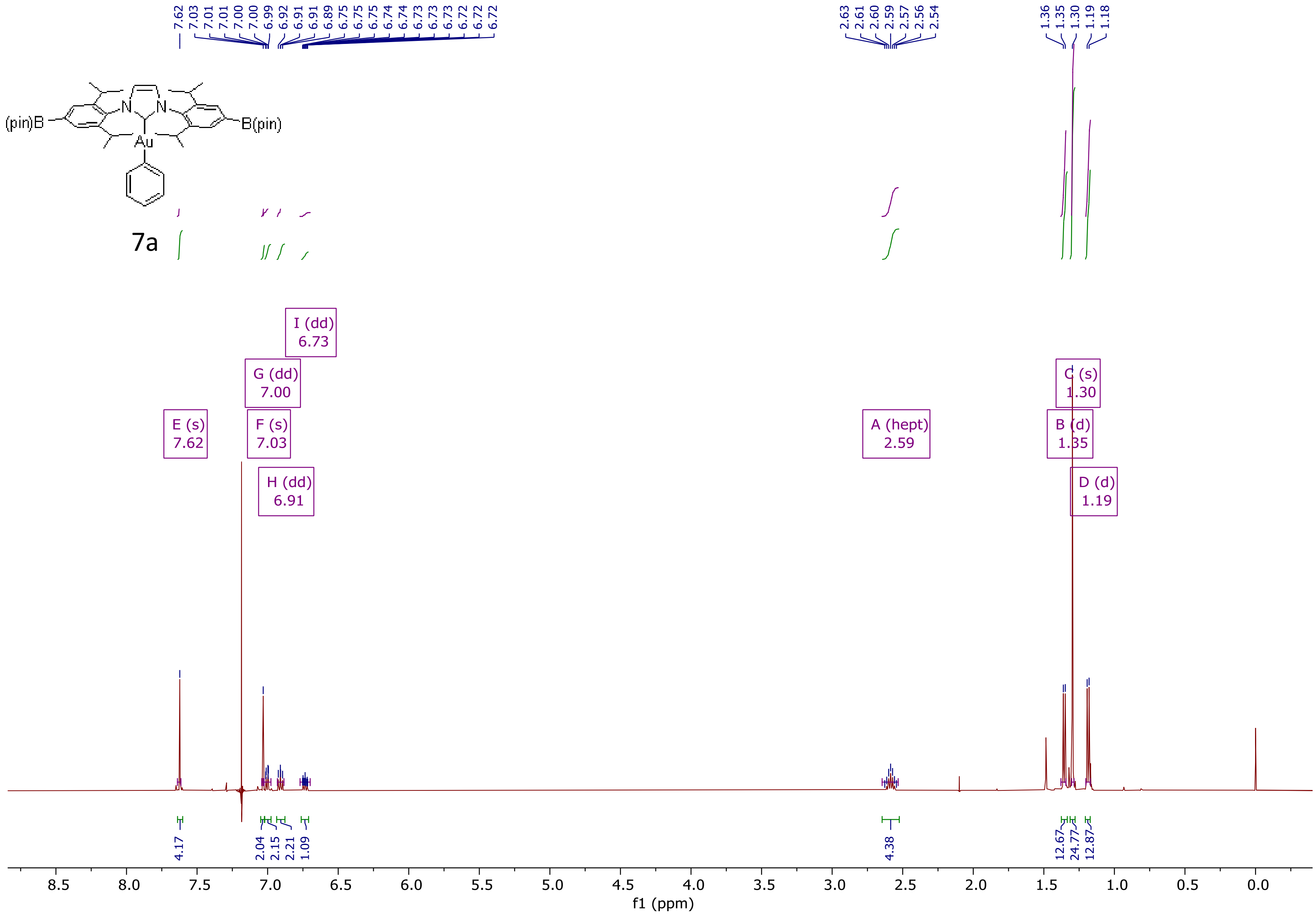

(pin)B
B(pin)
N
N
Au
E (s)
7.62
F (s)
7.03
G (dd)
7.00
H (dd)
6.91
I (dd)
6.73
A (hept)
2.59
B (d)
1.35
C (s)
1.30
D (d)
1.19
4.17
2.04
2.15
2.21
1.09
4.38
12.67
24.77
12.87
f1 (ppm)

7a

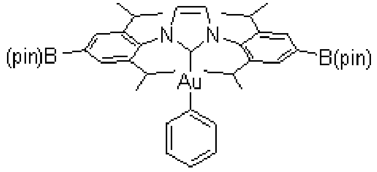
(pin)B
B(pin)
N
N
Au

7a

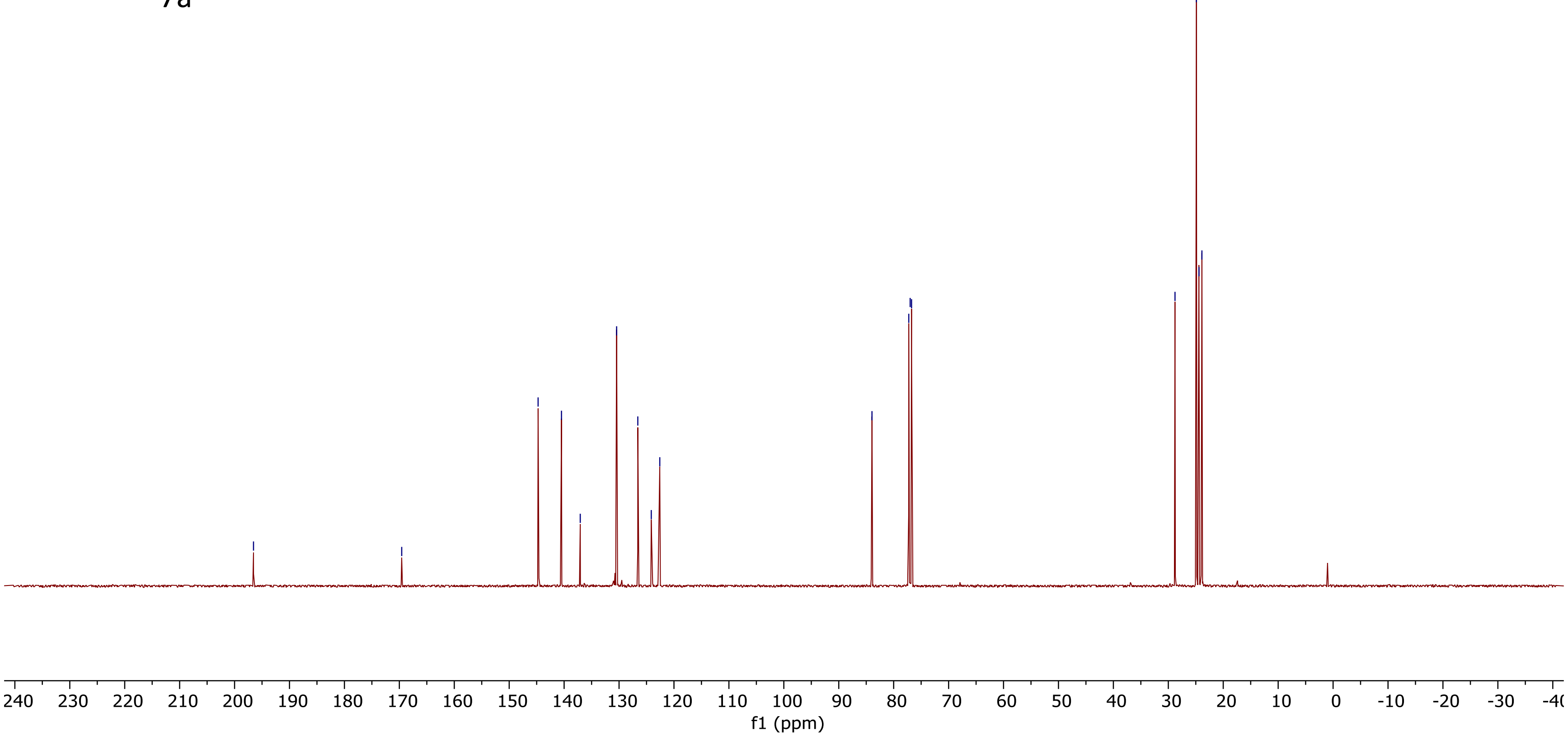
FI-307-01-CDCl3.11.fid
196.55
169.57
144.74
140.48
137.06
130.44
126.59
124.14
122.58
83.95
77.25
77.00
76.75
28.78
24.88
24.43
23.88
240 230 220 210 200 190 180 170 160 150 140 130 120 110 100 90 80 70 60 50 40 30 20 10 0 -10 -20 -30 -40
f1 (ppm)

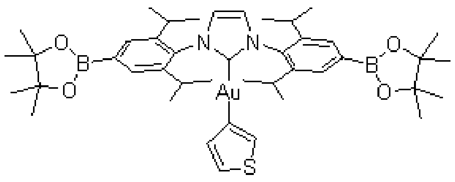

7b

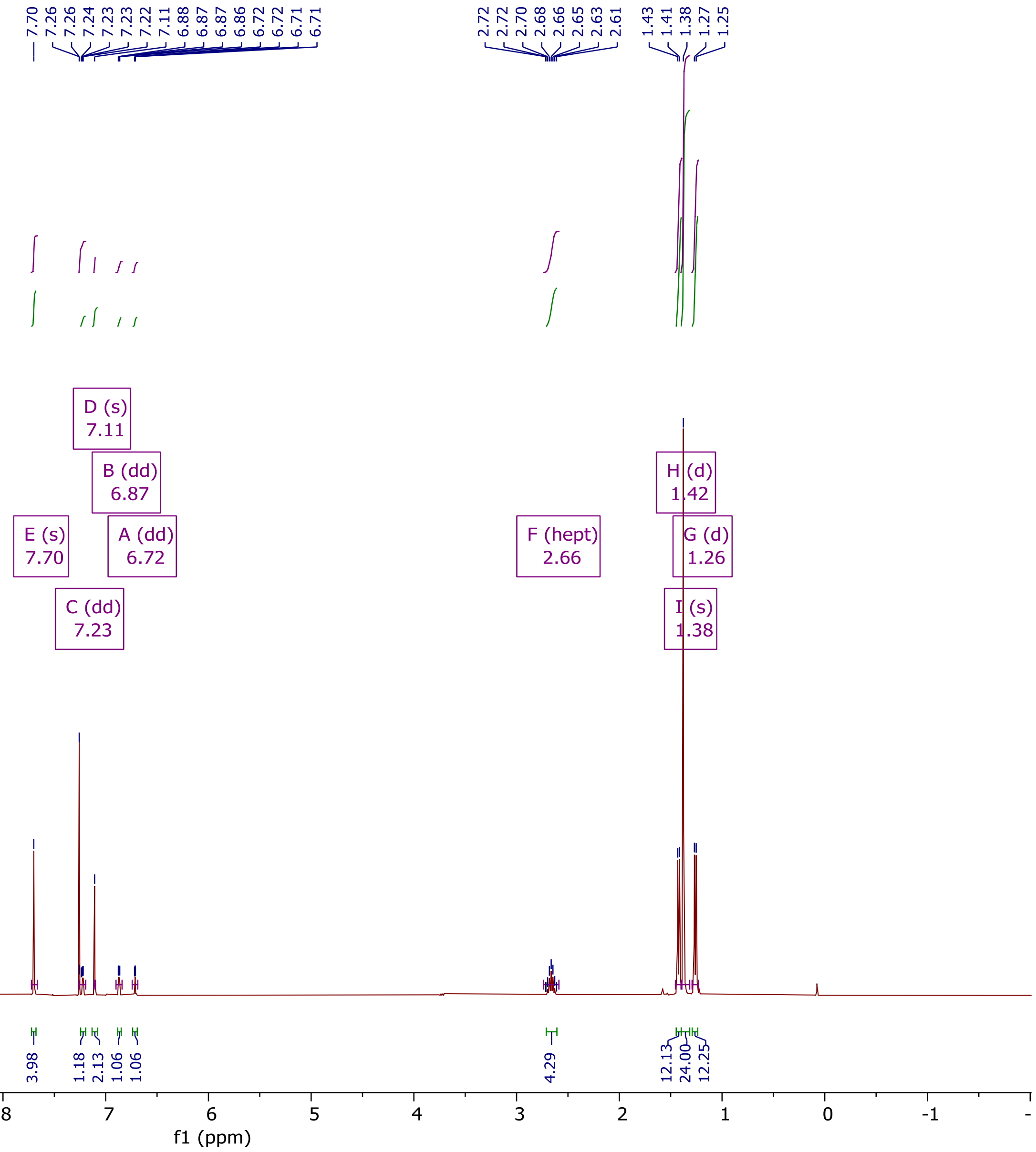
7.70 7.26 7.26 7.24 7.23 7.23 7.22 7.11 6.88 6.87 6.87 6.86 6.72 6.72 6.71 6.71
2.72 2.72 2.70 2.68 2.66 2.65 2.63 2.61
1.43 1.41 1.38 1.27 1.25
D (s)
7.11
B (dd)
6.87
E (s)
7.70
A (dd)
6.72
C (dd)
7.23
F (hept)
2.66
H (d)
1.42
G (d)
1.26
I (s)
1.38
3.98 1.18 2.13 1.06 1.06
4.29
12.13 24.00 12.25
14 13 12 11 10 9 8 7 6 5 4 3 2 1 0 -1
f1 (ppm)

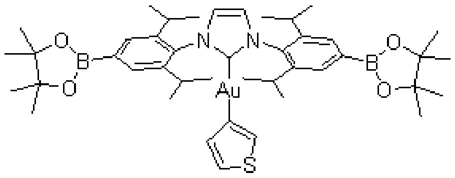

7b

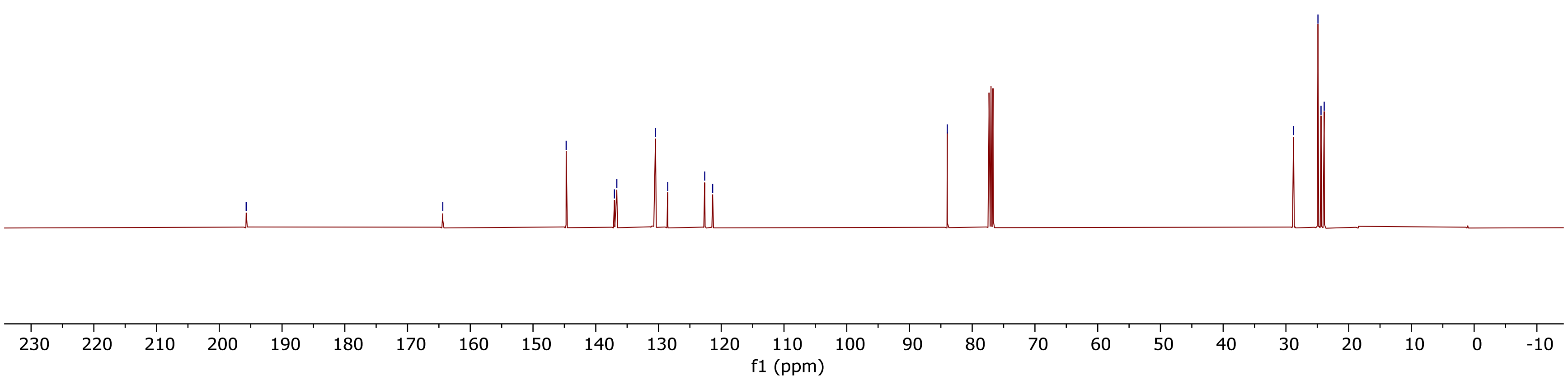
195.72
164.39
144.71
137.02
136.65
130.48
128.53
122.64
121.37
83.97
28.79
24.89
24.41
23.90
230 220 210 200 190 180 170 160 150 140 130 120 110 100 90 80 70 60 50 40 30 20 10 0 -10
f1 (ppm)

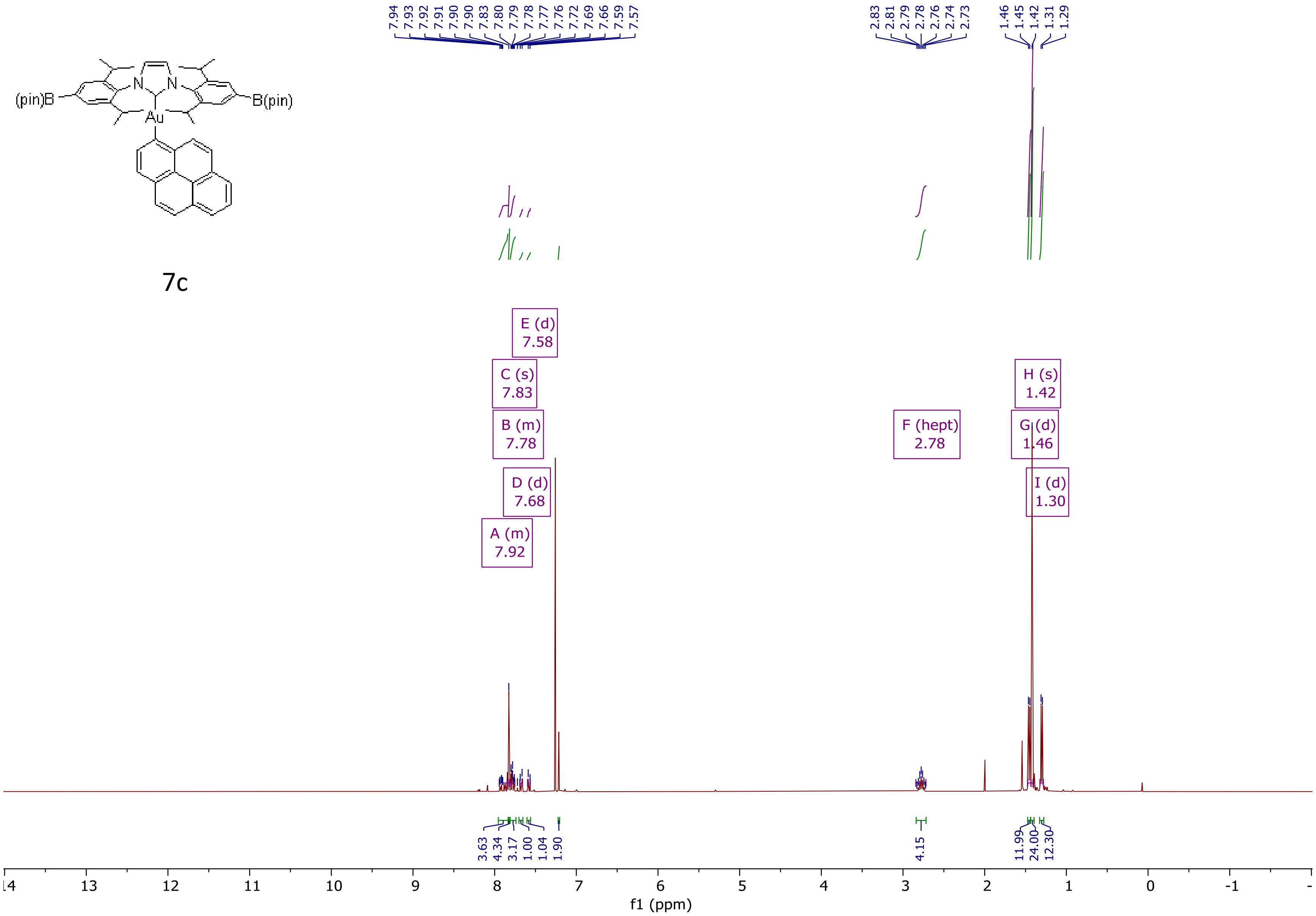
(pin)B
B(pin)
N
N
Au
7.94 7.93 7.92 7.91 7.90 7.90 7.83 7.80 7.79 7.78 7.77 7.76 7.72 7.69 7.66 7.59 7.57
2.83 2.81 2.79 2.78 2.76 2.74 2.74 2.73
1.46 1.45 1.42 1.31 1.29
E (d) 7.58
C (s) 7.83
B (m) 7.78
D (d) 7.68
A (m) 7.92
F (hept) 2.78
H (s) 1.42
G (d) 1.46
I (d) 1.30
3.63 4.34 3.17 1.00 1.04 1.90
4.15
11.99 24.00 12.30
14 13 12 11 10 9 8 7 6 5 4 3 2 1 0 -1
f1 (ppm)

7c

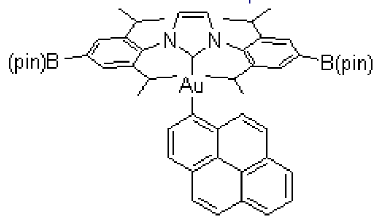
(pin)B
B(pin)
N
N
Au

7c

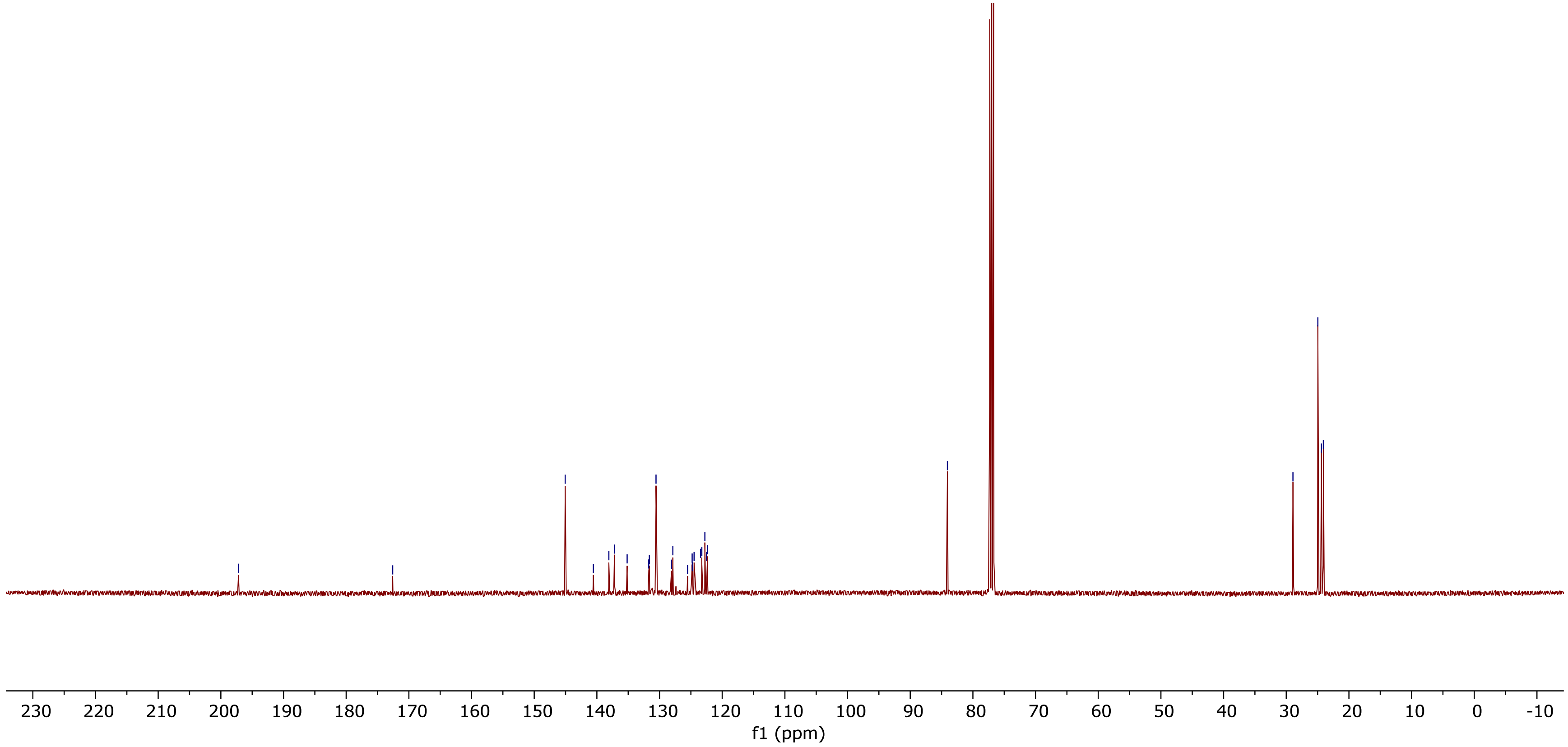
197.18
172.58
145.05
140.56
138.09
137.20
135.17
131.72
131.63
130.56
128.09
127.88
125.52
124.80
124.77
124.49
123.44
123.26
122.77
122.50
122.35
84.06
28.93
24.96
24.39
24.08
230 220 210 200 190 180 170 160 150 140 130 120 110 100 90 80 70 60 50 40 30 20 10 0 -10
f1 (ppm)
